# Theoretical Advances in Polariton Chemistry and Molecular Cavity Quantum Electrodynamics

**DOI:** 10.1021/acs.chemrev.2c00855

**Published:** 2023-08-08

**Authors:** Arkajit Mandal, Michael A.D. Taylor, Braden M. Weight, Eric R. Koessler, Xinyang Li, Pengfei Huo

**Affiliations:** † Department of Chemistry, University of Rochester, 120 Trustee Road, Rochester, New York 14627, United States; ‡ Department of Chemistry, Columbia University, New York, New York 10027, United States; § The Institute of Optics, Hajim School of Engineering, University of Rochester, Rochester, New York 14627, United States; ∥ Department of Physics and Astronomy, University of Rochester, Rochester, New York 14627, United States; ⊥ Theoretical Division, Los Alamos National Laboratory, Los Alamos, New Mexico 87545, United States

## Abstract

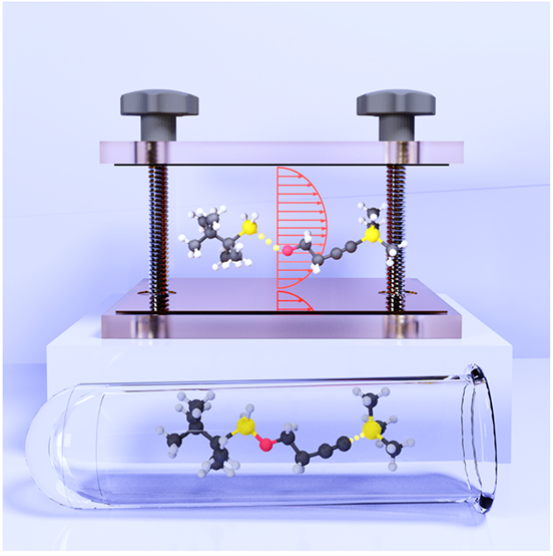

When molecules are coupled to an optical cavity, new light–matter hybrid states, so-called polaritons, are formed due to quantum light–matter interactions. With the experimental demonstrations of modifying chemical reactivities by forming polaritons under strong light–matter interactions, theorists have been encouraged to develop new methods to simulate these systems and discover new strategies to tune and control reactions. This review summarizes some of these exciting theoretical advances in polariton chemistry, in methods ranging from the fundamental framework to computational techniques and applications spanning from photochemistry to vibrational strong coupling. Even though the theory of quantum light–matter interactions goes back to the midtwentieth century, the gaps in the knowledge of molecular quantum electrodynamics (QED) have only recently been filled. We review recent advances made in resolving gauge ambiguities, the correct form of different QED Hamiltonians under different gauges, and their connections to various quantum optics models. Then, we review recently developed ab initio QED approaches which can accurately describe polariton states in a realistic molecule–cavity hybrid system. We then discuss applications using these method advancements. We review advancements in polariton photochemistry where the cavity is made resonant to electronic transitions to control molecular nonadiabatic excited state dynamics and enable new photochemical reactivities. When the cavity resonance is tuned to the molecular vibrations instead, ground-state chemical reaction modifications have been demonstrated experimentally, though its mechanistic principle remains unclear. We present some recent theoretical progress in resolving this mystery. Finally, we review the recent advances in understanding the collective coupling regime between light and matter, where many molecules can collectively couple to a single cavity mode or many cavity modes. We also lay out the current challenges in theory to explain the observed experimental results. We hope that this review will serve as a useful document for anyone who wants to become familiar with the context of polariton chemistry and molecular cavity QED and thus significantly benefit the entire community.

## 1 Introduction

Coupling matter (atoms, molecules, or solid-state materials) to the quantized electromagnetic field inside an optical cavity creates a set of new photon–matter hybrid states, so-called polariton states.^1−3^ These polariton states have delocalized excitations among molecules and the cavity mode, which have been shown to facilitate new chemical reactivities.^1,3,4^ Theoretical investigations play a crucial role in understanding new principles in this emerging field and have suggested interesting reaction mechanisms enabled by cavity quantum electrodynamics (QED).^5−14^

Unlike the traditional coherent control strategies,^15,16^ polariton chemistry does not rely on fragile electronic coherence^15,16^ and is robust to decoherence.^10^ Compared to the classical laser–matter interactions which operate with a large number of photons, cavity QED enables the hybrid system to initiate chemical reactions even without photons initially present in the cavity.^3^ Thus, polariton chemistry provides a new strategy to control chemical reactivity in a general way by tuning the fundamental properties of photons and provides a new paradigm for enabling chemical transformations that can profoundly impact catalysis, energy production, and the field of chemistry at large.

Recent experimental demonstrations^1,3,4,17,18^ of this modification of chemical reactivity, however, are not well understood and in some cases not reproducible.^19,20^ Since these polaritonic systems often require a quantum mechanical description of the photonic modes, existing physical chemistry theories for chemical reactions are no longer directly applicable to these hybrid systems, requiring a more exact QED approach. While the fundamental theories of QED has been known for decades (see Section 2.2), directly translating this knowledge to explain measurements of polariton chemistry remains as a major challenge in both theoretical chemistry and quantum optics. Namely, the mechanism behind the strong coupling of a mesoscopic scale ensemble of molecules to a single optical cavity is still not fully understood (see Section 6). The basic theory for describing the modes in different types of cavity is also briefly discussed in Section 2.6. Additionally, simulating the time-dependent polariton quantum dynamics of the hybrid matter-field systems is often a necessary and essential task, as these polariton photochemical reactions often involve a complex dynamical interplay among the electronic, nuclear, and photonic degrees of freedom (DOFs). However, accurately simulating the polaritonic quantum dynamics remains a challenging task and is beyond the paradigm of traditional photochemistry, which does not include quantized photons, and quantum optics which does not have a well-defined theory to include the influence of nuclear degrees of freedom to describe reactivity nor properly account for molecular structures.^21^ Over the past years, enormous progress has been made to address this interdisciplinary challenge. We have witnessed how electronic structure theory (Section 3), nonadiabatic quantum dynamics (Section 4), and statistical mechanics (rate constant theory, in particular, Section 5) have actively participated in this exciting field in the past few years.

Polariton chemistry has become a fast-growing community, with exciting progress occurring daily. We feel this is the right time to review this exciting progress and encourage more people from both chemistry and quantum optics to continuously contribute to this ever-growing field. In this review, we will focus our discussion on the extensive *theoretical advances* in polariton chemistry. As such, we will not review, in detail, many experimental works, except briefly mention them in Section 4 and Section 5. For those readers interested in the details of the experimental works, there are a number of excellent reviews available that discuss the experimental progress and challenges of polariton chemistry.^1,2,22−24^ We hope that this review will serve as a useful tool for anyone who wants to become familiar with the recent theoretical advances in polariton chemistry and molecular cavity QED and will significantly benefit the entire community.

### 1.1 Jaynes-Cummings Model in Cavity QED

In quantum optics, atoms/molecules (modeled as two-level systems) coupled to a single mode in an optical cavity are a well-studied subject. This study has led to well-known model Hamiltonians, such as the Jaynes-Cummings model^25^ and the Tavis-Cummings model.^26^ Since these two models are also widely used in recent investigations of polariton chemistry, here we briefly discuss them and the intuitive insights they provide. We would like to emphasize that the JC and TC models are good qualitative pictures, but their accuracy becomes questionable at best for realistic molecular systems (see Section 2).

**Figure 1:**
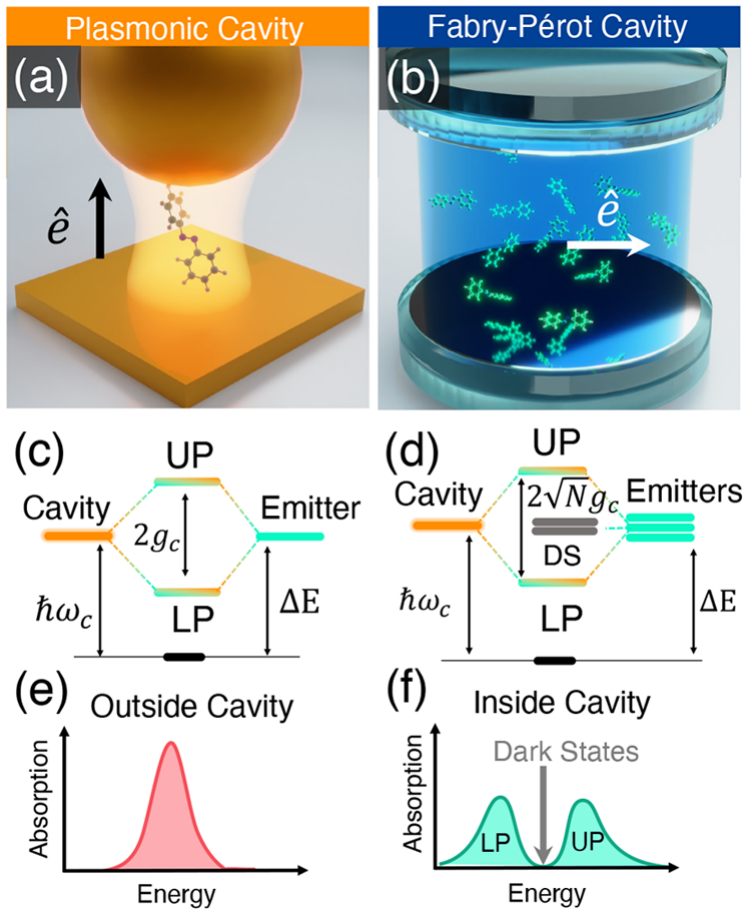
Schematic illustrations of commonly used optical cavities in molecular polariton research. (a) Plasmonic cavity: A single molecule coupled to a plasmonic field. (b) Fabry–Pérot cavity: An ensemble of molecules coupled to a quantized vacuum radiation field. In both panels, the arrows and **ê** indicate the dominant cavity field polarization directions that matter couple to. Panels (c) and (d) depict the polariton spectrum for a single molecule coupled to cavity (depicted in panel a) and *N* molecules collectively coupled to a cavity (depicted in panel b). (e) Molecular absorption, and (f) the Polariton absorption. For a single molecule case, there is no dark state (see panel c), but for the *N*-molecule collective coupling case, one can observe the dark states due to their nearly zero transition dipole.

We consider a single emitter with two electronic states |*g*⟩ and |*e*⟩ with the following matter Hamiltonian
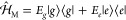
1where *E*_*g*_ and *E*_*e*_ are the ground and excited state energy. The well-known Jaynes-Cummings (JC) Model^25^ is used to describe the single emitter-cavity hybrid systems and has the following form
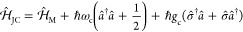
2where σ^†^ = |*e*⟩⟨*g*| and σ = |*g*⟩⟨*e*| are the creation and annihilation operators for the molecular excitation, respectively, and *â*^†^ and *â* are raising and lowering operators of the cavity field, respectively, with the cavity frequency ω_c_. The term  describes the cavity field (under the single mode approximation); its eigenstate |*n*⟩ describes the number of photons inside the empty cavity (without the presence of the emitter), where *n*=⟨*n*|*â*^†^*â*|*n*⟩. Lastly, *g*_c_ is the coupling strength between the matter and the cavity field, which is often expressed as
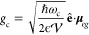
3where **μ**_*eg*_ is the transition dipole vector between the |*g*⟩ and |*e*⟩ states, **ê** is the cavity field polarization direction (with the hat indicating its status as a unit vector), ϵ is the permittivity inside the cavity (for vacuum, ϵ = ϵ_0_), and  is the effective cavity quantization volume. A rigorous derivation of the JC model Hamiltonian from the minimal coupling Hamiltonian (eq 35) can be found in Section 2.5. Experimentally, such single emitter-cavity strongly coupled systems can be realized in plasmonic cavity setups^27^ as shown schematically in Figure 1a. This Jaynes-Cummings Hamiltonian is used ubiquitously across the field of quantum optics, from quantum computing^28^ applications to fundamental physics experiments.^29,30^

The eigenstates of the JC Hamiltonian can be obtained analytically, using a convenient basis of matter and photon states, |*g*⟩⊗|*n*⟩ ≡|*g*,*n*⟩ and |*e*⟩⊗|*n*⟩ ≡|*e*,*n*⟩ for *n* = 0, 1,···. The polariton ground state of the hybrid system is |*g*,0⟩, and the *n*_th_ excited upper polariton state (|+,*n*⟩) and the *n*_th_ excited lower polariton state (|–,*n*⟩) are
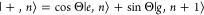
4a
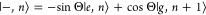
4bwhere  is the mixing angle, and Δ*E* = *E*_*e*_ – *E*_*g*_ is the energy difference between the ground and excited states. The eigenenergies of the polariton states are
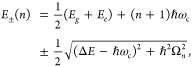
5where  is the *n*_th_ Rabi frequency (which is the Rabi splitting under the resonant condition when Δ*E* – *ℏω*_c_ = 0). Note that when the light–matter detuning (Δ*E* – *ℏω*_c_) is zero, , and *E*_±_ (*n*) = *E*_*g*_ + *ℏω*_c_ (*n* + 3/2) ± Ω_*n*_/2. This is the resonance case, which is schematically depicted in Figure 1c for the *n* = 0 case. A full diagram of JC polariton eigenstates with all *n* is commonly referred to as the Jaynes-Cummings ladder (e.g., see Figure 1 in ref (21)). In the JC model, the difference in energy between the upper and lower polariton states is called the “Rabi splitting”
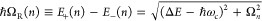
6

For a resonant light–matter coupling, Δ*E* – *ℏω*_c_ = 0, , which scales linearly with the coupling strength *g*_c_ and the square root of the “photon number” *n*, providing a simple and intuitive way to consider how a system changes as a function of coupling strength. Figure 1c depicts the situation for *n* = 0.

The JC Hamiltonian in eq 2 and its eigenenergies (eq 5) correspond to the ideal cavity situation where the cavity photon loss and the matter de-excitation process (e.g., due to the nonradiative decay) are not considered. In a realistic experimental setup, the cavity photon only has a finite lifetime before it leaks outside the cavity. The condition to achieve strong coupling, (i.e., where one can observe the Rabi splitting in absorption spectra) depends on the relation between the excitation lifetimes in the cavity and the coupling strength *g*_c_.

One can phenomenologically introduce different sources of dissipation that lead to a spectroscopic broadening of the light–matter eigenspectrum. Let us denote the loss rate for the cavity photon as κ, and the decay rate of the matter excitation as γ (see Figure 14a for a schematic illustration). For the Markovian dissipation at zero temperature, the cavity–matter density matrix for the JC model is given with the quantum Liouville equation  where  is the dissipative part based on the Lindblad jump operator *â* with a similar expression with the matter DOFs for . For the JC model, the approximate evolution of the density matrix under such dissipation can be captured by defining an effective Hamiltonian
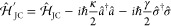
7such that  when ignoring the 2*âρ̂â*^†^ and 2*σ̂ρ̂σ̂*^†^ terms in  and . Similar to the JC model Hamiltonian, when including dissipation,  is block-diagonalized within the {|*e*, *n*⟩, |*g*, *n* + 1⟩} subspace and the matrix elements of  within this subspace are written as
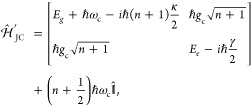
8where  is the identity operator (in this electronic–photonic subspace). The complex eigenvalues of  are obtained by diagonalizing the above 2 × 2 matrix as,^31−35^
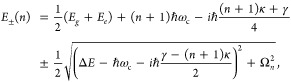
9where the real parts of *E*_±_(*n*) are energies of the states |±,*n*⟩ and the imaginary parts yield their broadening. In resonance, when *E*_*g*_ + *ℏω*_c_ = *E*_*e*_, the Rabi-splitting is . Thus, to observe the Rabi-Splitting at *n* = 0, we require Ω_*n*_ ≫ κ or γ which defines the strong coupling regime.

To get an intuitive understanding of the cavity-modified photochemistry, consider the Hamiltonian in the |*e*, 0⟩ (the molecule in the excited state with 0 photons in the cavity) and |*g*, 1⟩ (the molecule in the ground state with 1 photon in the cavity) subspace. This is the most common treatment of the Jaynes-Cummings Hamiltonian, and further details on it can be seen in refs (6, 36). The polariton Hamiltonian within this subspace is expressed as follows
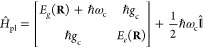
10where  is the zero point energy of the quantized photon mode inside the cavity. Here we have made the replacement *E*_*g*__/*e*_ → *E*_*g*__/*e*_(**R**) such that the ground and excited state potential energies depend on molecular nuclear configuration **R**, that is *E*_*g*_(**R**) and *E*_*e*_(**R**) are the molecular potential energy surfaces (PES).

The polariton potential energy surfaces can be obtained by diagonalizing 2 × 2 matrix given in eq 10 and are given as
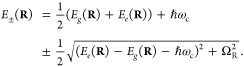
11

These light–matter hybrid PESs *E*_±_(**R**), so-called polaritonic PESs, adapt their curvature from both the ground and the excited state PESs and depend on the light–matter coupling strength *ℏg*_c_ and the cavity photon frequency *ℏω*_c_. Therefore, the excited state potential energy landscape, and consequently the photochemistry of the cavity-molecule system, is modified with *ℏg*_c_ and *ℏω*_c_ acting as tuning knobs to control the molecular excited state dynamics. Note that within the approximated JC model, the |*g*, 0⟩ state has the PES  which is the same as the molecular ground state |*g*⟩ other than the irrelevant zero-point energy shift of . This change of PES landscape is the central idea of polariton photochemistry (in the single molecule coupled to a single radiation mode limit) which will be discussed in detail in Sections 3 and 4. Details of the rigorous light–matter Hamiltonian, as well as various approximate ones (such as the JC model), and their applicability are discussed in Section 2.

### 1.2 Tavis-Cummings Model and Collective Light–Matter Coupling

Most of the recent molecular cavity QED experiments,^3,34,37−40^ however, use the setup illustrated in Figure 1b, where many molecules are collectively coupled to the quantized electromagnetic field inside a Fabry–Pérot optical cavity (formed by reflecting mirrors). To describe this collective regime of light–matter coupling, the Tavis-Cummings (TC) model Hamiltonian^26^ is used as an analog to the JC Hamiltonian with many molecules. This model is under the same level of approximation (mainly the rotating wave approximation) as the JC model but with many molecules, taking the following form
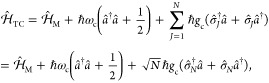
12where *J* is the index of the two-level atoms/molecules in the cavity (and there are a total of *N* of them effectively coupled to the cavity), with corresponding exciton creation operator, σ̂_*J*_^†^=|*e*_*J*_⟩⟨*g*_*J*_|, and annihilation operator, *σ̂*_*J*_ = |*g*_*J*_⟩⟨*e*_*J*_|. Further, due to the model’s symmetry, one can introduce the collective excitation operator  and collective de-excitation operator . Similar to the JC model, the TC model also has analytical solutions to its eigenstates and eigenenergies in the first excitation subspace. The total ground state is |*G*, 0⟩ = |*g*_1_⟩ ⊗···|*g*_*J*_⟩··· ⊗|*g*_*N*_⟩ ⊗|0⟩, the photon dressed ground state is |*G*, 1⟩, where all the emitters are in the ground state with one photon in the cavity, and the state where the all the molecules are in the ground state except for the *J*_th_ molecule in the excited state is |*E*_*J*_, 0⟩ = |*g*_1_⟩ ⊗···|*e*_*J*_⟩··· ⊗|*g*_*N*_⟩ ⊗|0⟩. In the single excitation manifold, the collective “bright state” of the matter is
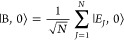
13which will explicitly couple to the |*G*, 1⟩ state, resulting in the polariton states | ± ⟩ (which have nonzero transition dipoles from the |*G*, 0⟩ states) as follows
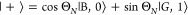
14a
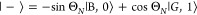
14bwhere  is the mixing angle under the collective coupling regime, and Δ*E* = *E*_*e*_ – *E*_*g*_ is the energy difference between the bright state |*B*, 0⟩ (as well as the singly excited manifold) and ground state |*G*, 0⟩. Through the collective coupling to the cavity, the polariton states are delocalized across all *N* molecules in the cavity and should be viewed as mesoscopic quantum states that involve *N* molecules and a single cavity mode. When *N* = 1 (single molecule), the |±⟩ states in eq 14 reduces back to the |±,0⟩ states of the JC model in eq 4.

The eigenenergies of the upper and lower polariton states also differ from the single-molecule picture because their Rabi splitting now scales with  as
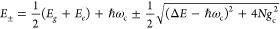
15where the collective Rabi splitting is defined as
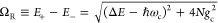
16which scales as . Figure 1d shows a schematic of an energy level diagram for this system at the resonance condition (when Δ*E* – *ℏω*_c_ = 0), and the Rabi splitting is written as
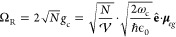
17

This is a typical example of the collective effect, demonstrating how many molecules collectively coupled to the cavity can enhance the effective coupling strength by , or collectively enhance the Rabi splitting with the concentration  of the molecules inside the cavity.^39,41,42^

The rest of the *N* – 1 eigenstates (in the single excitation manifold) of the TC model are referred to as the “dark” states^9,40,43^ (labeled by α) which are expressed as follows
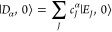
18where ∑_*J*_*c*_*J*_^α^ = 0 for all dark states α. These states are superpositions of the *N* matter states {|*E*_*J*_, 0⟩}, and thus are also delocalized across *N* molecules (one should note their difference compared to the individual localized excited state |*E*_*J*_, 0⟩). Energetically, they are the same as the original single-molecule excitation, *E*_*e*_, and are depicted as the gray states in Figure 1d. These dark states do not mix with the photon-dressed state |*G*, 1⟩ and do not contain any photonic excitation component under the TC model consideration. These dark states are also optically dark from the ground state |*G*, 0⟩ due to the net zero transition dipole ⟨*D*_*α*_,0|∑_*J*_*μ̂*^*J*^ |*G*,0⟩ = *μ*_*eg*_ ∑_*J*_C_*J*_^α^ = 0 if we assume ⟨*e*|**μ̂*^*J*^* |*g*⟩ = *μ*_*eg*_ for all *J* ∈ [1, *N*] emitters. Optically, one will see no significant absorption in between two polariton absorption peaks (when ignoring disorder). It should be noted that this treatment of the “dark” states is only valid in the Tavis-Cummings level of theory (when considering identical, noninteracting emitters without any disorder). In real molecular systems, there are dynamical fluctuations in the molecules due to phonon fluctuations (electron–nuclear interactions) such that even states that are “dark” in the TC level of theory have some nonzero photonic character^40^ (See Section 2.6 and Section 6 for more information on when the TC picture breaks down and recent theoretical advances in modeling collective systems). Considering intermolecular interactions will also break the degeneracy of the dark states, as shown in ref (44). In some of the recent molecular polariton experiments, it has been theoretically estimated (assuming a simple TC model Hamiltonian) that there are *N* ∼ 10^6^ – 10^11^ organic molecules effectively coupled to the cavity mode.^45,46^ For CdSe nanoplatelets coupled to a Fabry–Pérot cavity,^40^ it was estimated that there are *N* ∼ 10^3^ emitters per cavity mode.

### 1.3 Theoretical Challenges

In quantum optics, coupling strengths can be classified as weak, strong, ultrastrong, and deep strong.^47^ The classification between weak and strong is governed by the relationship between the coupling strength, , and the loss rate (whether cavity or molecule energy loss), γ. The coupling is considered weak for  and strong for . The classification between ultrastrong and deep strong, however, depends on the ratio *g*_c_/ω_c_, with the ultrastrong regime being , and the deep strong regime being .

While the Jaynes-Cummings and Tavis-Cummings models provide valuable, intuitive insights into how light couples with matter inside optical cavities, these models are subject to many approximations: the rotating wave approximation, the dipole approximation, the two-level approximation, and also the absence of permanent dipole and dipole self-energy. As coupling strengths increase, these approximations begin to break down,^47,48^ and more rigorous Hamiltonians should be used (such as those discussed in Section 2). In the ultrastrong and deep coupling regimes, the JC and TC models fail to accurately capture the results of more rigorous methods.

The necessity of using more rigorous models is substantiated by the recent progress of experimentation in recent years. For example, the Ebbesen group in ref (49) achieved ultrastrong light–matter coupling in a Fabry–Pérot cavity with an effective . Additionally, for single molecules in plasmonic cavities, the Baumberg group in ref (27) demonstrates strong coupling that was nearly in the ultrastrong regime. These seminal experiments cannot be accurately described with the simple JC and TC models. In this manner, there has been a significant push in recent years to advance the theoretical understanding and simulations for these systems to explain current experiments and predict future ones.^50^

### 1.4 Outline of the Review

This review summarizes recent theoretical advances in polariton chemistry, and it is organized as follows. Section 2 discusses the fundamental theoretical framework behind light–matter interactions. Starting from the most rigorous Hamiltonian, it discusses how and when to perform various approximations to reduce the computational complexity while keeping the relevant physics. Section 3 discusses how to apply the fundamental framework of the previous section to realistic systems with ab initio electronic structure methods. This section reviews different methods of marrying electronic structure methods to these hybrid light–matter systems to model complicated polariton systems. Section 4 applies the methods of Sections 2 and 3 to photochemistry, showing how simple chemical reactions such as photoisomerization or charge transfer reactions can be altered by strongly coupling electronic transitions to a cavity. Section 5, similarly, summarizes recent progress in understanding vibrational strong coupling (VSC), where the nuclear vibrational states are strongly coupled to the cavity, leading to changes of the ground state chemical kinetics. This section further shows how the fundamentals of statistical mechanics like rate constant theory can be used to understand these reactions. Section 6 goes on to present recent theoretical explanations of experiments in the collective coupling regime, a regime that is largely mysterious since direct modeling of experimentally relevant numbers of molecules is typically impossible, and simple models like the TC model break down for experimentally realizable coupling strengths. This section also discusses various recent theoretical hypotheses to explain the experimentally observed suppression or enhancement of the reaction rate constant under the collective vibrational strong coupling regime.

## 2 Fundamental Theory of Light–Matter Interactions

While the Jaynes-Cummings and Tavis-Cummings models discussed in the Introduction provide an intuitive understanding of light–matter interactions, these simplified models break down for many systems that cannot be thought of as two-level systems or have permanent dipole.^51^ For most molecular systems, a more rigorous framework is needed to provide even qualitatively accurate results. With this in mind, this section discusses the various theoretical representations that go beyond simple quantum optics models like the Jaynes-Cummings model.

Going beyond the framework discussed in the Introduction, this section outlines the fundamental theory of cavity QED. Section 2.1 starts off by reviewing the formulation of molecular Hamiltonians. Section 2.2 similarly reviews quantum electrodynamics (QED). Section 2.3 discusses the most common cavity QED Hamiltonians as they are represented in the full Hilbert space. Section 2.4 then goes on to show recent advances and controversies on how to accurately represent these QED Hamiltonians in a truncated Hilbert space. Section 2.6 discusses a further extension of the typical QED Hamiltonians to models which include many molecules and many photonic modes in a single cavity.

We also recommend to readers the following resources for further reading: ref (47) provides an excellent review on different coupling regimes of light–matter interactions, including the ultrastrong and deep-strong couplings; ref (52) provides an extensive discussion on gauge ambiguities in a broader perspective; ref (53) provides a thorough review on recent progress in molecular cavity QED; refs (54−58) provide fundamental discussions on QED and cavity QED; and lastly, refs (57 and 59) provide an excellent introduction to quantum optics.

### 2.1 A Review of Molecular Hamiltonians

Here, we briefly review some basic knowledge of the molecular Hamiltonian, which will be useful for our discussions of molecular cavity QED. We begin by defining the matter Hamiltonian as follows
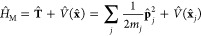
19where *j* is the index of the *j*_th_ charged particle (including all electrons and nuclei), with the corresponding mass, *m*_*j*_, and *canonical* momentum, **p̂**_*j*_ = -*iℏ*∇_*j*_. We denote electronic coordinate with **r̂**, and nuclear coordinate with **R̂**, and use **x̂**_*j*_∈{**r**_*j*_,**R**_*j*_} to represent either the electron or nucleus, with **x̂** being the coordinate operator for all charged particles. Further, **T̂** = **T̂**_**R**_ + **T̂**_**r**_ is the kinetic energy operator for all charged particles, where **T̂**_**R**_ and **T̂**_**r**_ represent the kinetic energy operator for nuclei and for electrons, respectively. Further, *V̂***(x̂)** is the potential operator that describes the Coulombic interactions among the electrons and nuclei. The electronic Hamiltonian is often defined as
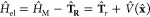
20which includes the kinetic energy of electrons, electron–electron interactions, electron–nuclear interactions, and nuclear–nuclear interactions. The essential task of the electronic structure community is focused on solving the eigenstates of *Ĥ*_el_ at a particular nuclear configuration **R** as follows
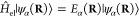
21where *E*_*α*_(**R**) is commonly referred to as the α_th_ potential energy surface (PES) or adiabatic energy, and |*ψ*_*α*_(**R**)⟩ is commonly referred to as the α_th_ adiabatic electronic state.

In the adiabatic electronic basis {|*ψ*_*α*_(**R**)⟩}, the matter Hamiltonian can be expressed as^60,61^
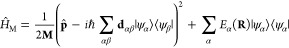
22where **P̂** is the nuclear momentum operator, **M** is the tensor of nuclear masses, and we have used the shorthand notation |*ψ*_*α*_⟩ ≡|*ψ*_*α*_(**R**)⟩, and **d**_*αβ*_ is the derivative coupling expressed as
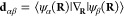
23

Note that the above equation is equivalent^60,61^ to the commonly used form of the vibronic Hamiltonian
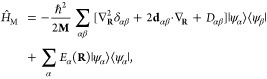
where *D*_*αβ*_ = ⟨*ψ*_*α*_ (**R**)|∇_*R*_^2^ |*ψ*_*β*_ (**R**)⟩ is the second derivative coupling. A simple proof can be found in ref (51).

Later, we will see that the dipole operator plays an important role in describing light–matter interactions, so let us spend a bit of time to discuss the molecular dipole operator. The total dipole operator of the entire molecule is
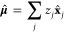
24where *z*_*j*_ is the charge for the *j*_th_ charged particle. The matrix elements of the total dipole operators can be obtained using the adiabatic states as
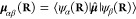
25

For α ≠ β, **μ**_*αβ*_ (**R**) is referred to as the transition dipole between state |*ψ*_*α*_⟩ and |*ψ*_*β*_⟩, while **μ**_*αα*_ (**R**) is commonly referred to as the permanent dipole for state |*ψ*_*α*_⟩.

It is often difficult to get accurate electronic states for highly excited adiabatic states. It is thus ideal to consider a Hilbert subspace of the electronic Hamiltonian. Considering a finite subset of electronic states {|*ψ*_*α*_⟩} (see eq 21) where there is a total of  matter states, one can define the following projection operator
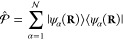
26which defines the truncation of the full electronic Hilbert space  which has an infinite basis, to a subspace  that contains a total of  states, where  is the identity operator in the electronic Hilbert subspace (the subspace containing all of the electron DOF) and  is the subspace being projected out.

Using the projection operator, one can define the projected matter Hamiltonian (or the truncated matter Hamiltonian) as follows
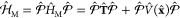
27

Throughout this review, we use calligraphic symbols (such as ) to indicate operators in the truncated Hilbert space, which we have already started in eq 1 of the Introduction.

One can also explicitly write the dipole operator in the truncated Hilbert space as follows
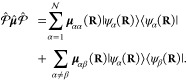
28

In the *same* truncated electronic subspace as defined by  (eq 26), we can diagonalize the dipole matrix in eq 28 to obtain
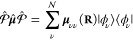
29where |*ϕ*_*ν*_⟩ is the eigenstate of the projected dipole operator  with
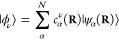
30and *c*_α_^ν^ (**R**) = ⟨*ψ*_*α*_ (**R**)|*ϕ*_*ν*_⟩. An example of the dipoles for LiF is provided in Figure 6(b).

The projection operator in eq 26 can also be expressed as
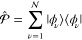
31which is simply a unitary transform of eq 26 (from the |*ψ*_*α*_(**R**)⟩-representation to the |*ϕ*_*ν*_⟩-representation).

In the literature, the eigenstates of , {|*ϕ*_*ν*_⟩}, are referred to as the Mulliken-Hush (MH) diabatic states,^62−66^ which are commonly used as approximate *diabatic* states that are defined based on their characters. They are approximate diabatic states in the sense that
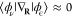
32hence, we drop the **R**-dependence in |*ϕ*_*ν*_⟩. Constructing rigorous diabatic states (where the derivative coupling is rigorously zero for all possible nuclear configurations) in a finite set of electronic Hilbert spaces is generally impossible, except for diatomic molecules. Recent theoretical progress on diabatization can be found in refs (67−69).

In the electronic subspace defined within the MH diabatic subspace using  (eq 31), *Ĥ*_el_ (eq 20) has off-diagonal (or “diabatic”) coupling terms
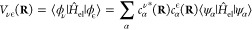
33

We can explicitly express the matter state projected
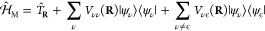
34

This is also the molecular Hamiltonian for any diabatic representation.

### 2.2 A Review of Quantum Electrodynamics

We provide a quick review of quantum electrodynamics (QED).^48,53^ We begin by writing the electric field as **Ê**(**r**) = **Ê**_∥_(**r**) + **Ê**_⊥_(**r**), with its longitudinal part **Ê**_∥_(**r**) that is curl free (irrotational), ∇ × **Ê**_∥_(**r**) = 0, and the transverse part, **Ê**_⊥_(**r**), that is divergence-free (solenoidal), ∇· **Ê**_⊥_(**r**) = 0. The magnetic field is purely transverse **B̂** (**r**) = **B̂**_⊥_ (**r**), because it is divergence-free ∇· **B̂** (**r**) = 0. These fields have spatial dependence, with spatial coordinate **r** (not to be confused with the electronic coordinate operator, **r̂**).

In the context of cavity QED, most simulations are performed in one of two gauges, either the Coulomb gauge^54^ or the dipole gauge (or equivalently the Poincare/multipolar gauge under the dipole approximation^54^),^5,13,70^ where the term “gauge” refers to the specific representation of the vector potential **Â**. Expressing **Â** = **Â**_∥_ + **Â**_⊥_, with its longitudinal part **Â**_∥_ that is curl free ∇ × **Â**_∥_ = 0, and the transverse part **Â**_⊥_ that is divergence-free ∇· **A**_⊥_ = 0. In principle, one can do gauge transformations that change the longitudinal part **Â**_∥_, because the physically observed quantities will not change, (e.g., the magnetic field, since **B̂** = ∇ × **Â** = ∇ × **Â**_⊥_). One often refers to fixing a gauge by choosing the value of ∇ **Â**, and the gauge transformation as a unitary transformation that is effectively adding an additional ∇χ component to **Â**_∥_, which is purely longitudinal because when χ is a scalar function in space, and ∇χ is curl-free (∇ × ∇χ = 0).

When deriving QED from first-principles, one often uses the minimal coupling Hamiltonian in the Coulomb gauge^71^ (see eq 45). From there, the electric-dipole Hamiltonian can be found via a gauge transformation. The commonly used Pauli-Fierz (PF) QED Hamiltonian^48,53,72^ (see eq 56) in recent studies of polariton chemistry can be obtained by applying another gauge transformation on the electric-dipole Hamiltonian. We will further discuss the consequence of matter state truncation on gauge invariance, the connection with the commonly used quantum optics model Hamiltonians, and when they will break down in molecular QED.

When fixing a specific gauge, one defines the gauge-dependent vector and scalar potentials for the electromagnetic field. By choosing the Coulomb Gauge (i.e., by enforcing ∇ · **A** = 0) which makes the vector potential purely transverse, **Â** = **Â**_⊥_, the Hamiltonian of point charge particles (including both electrons and nuclei) interacting with the electromagnetic field can be written as follows^54^
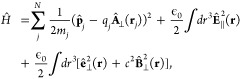
35where the sum includes *both* the nuclear and electronic DOFs, **r**_*j*_ and **p**_*j*_ are the position and momentum of the charged particle *j*, with the charge *q*_*j*_ and mass *m*_*j*_. Further, **A**_⊥_(**r**), **E**_⊥_(**r**), and **B**_⊥_(**r**) are the transverse vector potential, electric field, and magnetic field, respectively. The energy associated with **E**_∥_(**r**) (the second term in eq 35) is given by
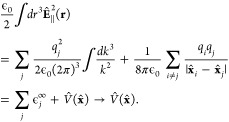
36

Here, the first term ∑_*j*_ ϵ_*j*_^∞^ in the third line of eq 36 is a time-independent infinite quantity that is referred to as the self-energy (not to be confused with the dipole self-energy), which can be regarded as a shift of the zero-point energy^57^ and is dropped in the last line of the above equation. It should be noted that eq 36 is for the free space situation; when a system is placed inside an optical cavity, the integral over *k* in eq 36 should be replaced by a discrete sum due to the cavity’s spacial confinement.^57^ Nevertheless, one often ignores these {ϵ_*j*_^∞^} terms as they only contribute to the zero-point energy of the field. In short, the Coulomb potential *V*_coul_ (**x̂**) ≡ *V*(**x̂**) emerges from the longitudinal electric field. The last term in eq 35 is the energy associated with the transverse fields **Ê**_⊥_(**r**) and **B̂**_⊥_(**r**). The general expressions for **Â**_⊥_(**r**), **Ê**_⊥_(**r**), and **B̂**_⊥_(**r**) are^54^
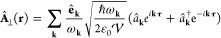
37a
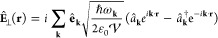
37b
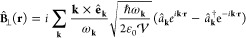
37cwhere *â*_**k**_^†^ and *â*_**k**_ are the raising and lowering operator of the mode that has a wavevector of **k** ≡ (*k*_*x*_, *k*_*y*_, *k*_*z*_), and they satisfy the canonical commutation relation^54^
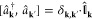
38*â*_k_^†^ and *â*_**k**_ are the creation and annihilation operators of the photon, respectively, δ_**k**,**k**′_ is the Kronecker delta, and the frequency of mode **k** is *ω*_*j*_ = *c*| **k**|. Here **k** = | **k**| **k̂** aligns in the direction of the unit vector **k̂** and **ê**_k⊥_**k̂** is the polarization unit vector for **Ê**⊥(**r**) and **Â**_⊥_ (**r**). The polarization of the photonic field can be written as a linear combination of the transverse electric (TE) polarization, **ê**_**k**_,_TE_, and the transverse magnetic (TM) polarization, **ê**_**k**_,_TM_, in relation to a given interface (where TE and TM must be defined relative to a surface) and propagation direction (defined by *k*). The TE mode’s polarization, **ê**_**k**_,_TE_, is defined as being perpendicular to the propagation direction and parallel to the interface defining the polarization coordinate system. The TM mode’s polarization, **ê**_**k**_,_TM_, is defined as being perpendicular to both the propagation direction and the TE polarization. For a given polarization, **ê**_**k**_, the transverse electric field is along **ê**_**k**_ and the magnetic field is along the **k̂** × **ê**_**k**_ direction. For example, for the TM mode, the transverse electric field polarization is along **ê**_**k**_,_TE_ and the transverse magnetic field polarization is along -**ê**_**k**_,_TM_. It should be noted that the quantization scheme in eq 37a is general but not unique (such as the sine mode functions used in ref^73, 74^.); however, it is a very common framework and is particularly salient for the purposes of this review.

When considering a planar Fabry-Pérot (FP) microcavity, **Â**_⊥_(**r**), **Ê**_⊥_(**r**), and **B̂**_⊥_(**r**) satisfy the boundary conditions and thus the wavevector **k** becomes quantized.^54,57^ For cavity mirrors imposing a boundary condition along *z* direction (see Figure 4), the *z* component of the wavevector  with *n* = 1, 2, 3··· as a positive integer. Note that *k*_*x*_ and *k*_*y*_ still remain quasi-continuous variables. These are discussed in details in Section 2.6.

Using the above expressions, the energy of the transverse fields, i.e., the last term in eq 35 is quantized as follows
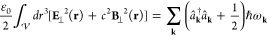
39where the spatial integral *dr*(3) is done within the effective quantized volume  of the cavity. Thus, eq 35 is quantized as
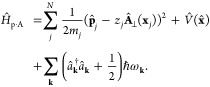
40

This is commonly referred to as the “p·A” or the minimal coupling QED Hamiltonian, in the sense that the light and matter coupling is only carried through the matter momentum and the vector potential of the field. The minimal coupling structure in eq 45 comes naturally due to the local *U*(1) symmetry of the EM field, which is an Abelian gauge field.

Assuming that the size of the molecular system is much smaller than the length of the cavity in the quantized direction, which is commonly referred to as the *long wavelength approximation*, the transverse fields can be treated as spatially uniform, i.e., *e*^*i*^^**k**· **r**^ ≈ 1, such that
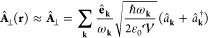
41

### 2.3 Cavity QED Hamiltonians

In cavity QED, one often considers only a *single mode* of the radiation field along the **ê** direction. This is commonly referred to as the single mode approximation in cavity QED, with the frequency ω_c_ = *πc*/*L* (*c* is the speed of the light, and ω_c_ represents the single mode frequency of the cavity), and the corresponding photonic creation and annihilation operators *â*^†^ and *â* (where we have dropped the label of **k** for a single mode.) While it is convenient to discuss and learn cavity QED under this approximation, real experiments have many modes present inside the cavity, so this approximation may not hold when considering realistic systems. With that in mind, we first introduce the formalism of cavity QED for a single photonic mode and then we generalize this for many modes in Section 2.6. Additionally, progress has been made on resolving ambiguities of mode truncation between different gauges.^75^

The single mode cavity photon field Hamiltonian, which is eq 39 under the single mode assumption, is then expressed as
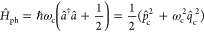
42where
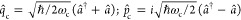
43are the photonic coordinate and momentum operators, respectively.

Under the single mode approximation, the vector potential (under the long wavelength approximation) in eq 41 can be expressed as
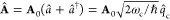
44where  is the vector field for a cavity. Note that we have also dropped the “⊥” symbol for the vector potential because it is purely transverse.

#### 2.3.1 The Minimal Coupling Hamiltonian

Under the long wavelength and single mode approximation, the “p·A” minimal coupling QED Hamiltonian (in the *Coulomb* gauge) in eq 40 is expressed as
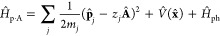
45where **p̂**_*j*_ = −*i*ℏ∇_*j*_ is the *canonical* momentum operator. Upon a gauge transformation
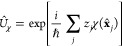
46where χ is a scalar function of position, and the gauge transformed p·A Hamiltonian is *Ĥ*_χ_= *Û*_χ_*Ĥ*_C_*Û*_χ_^†^, or more explicitly, expressed as follows
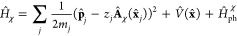
47where **Â**_χ_ (**x**_*j*_) = **Â** + ∇_*j*_ χ(**x̂**_*j*_) is the gauge transformed vector potential that provides the *same physical field*, because ∇_*j*_ × ∇_*j*_ χ(x **^**_*j*_) = 0.

We further introduce the Power-Zienau-Woolley (PZW) gauge transformation operator^54,76,77^ as
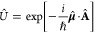
48or equivalently, with the following expressions
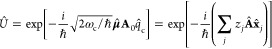


Recall that a momentum boost operator^53,78,79^ displaces *p̂* by the amount of *p*_0_, such that *Û*_p_*Ô*(*p̂*)*Û*_p_^†^ = *Ô* (*p̂* + *p*_0_). Hence, *Û* is a momentum boost operator for both the photonic momentum *p̂*_c_ by the amount of , as well as for the matter momentum **p̂**_*j*_ by the amount of z_*j*_**Â**. The PZW gauge operator (eq 48) is a special case of *Û*_*χ*_, such that χ = −**x̂**_*j*_ · , where **χ** now also explicitly dependents on **Â** (as appose to a pure function of matter coordinates). More detailed discussion related to the PZW gauge transformation can be found in section D_IV_.1 of ref (54) as well as the original paper by Woolley.^77^

Using *Û*^†^ to boost the matter momentum, one can re-express *Ĥ*_p·A_ in eq 45 as
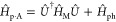
49hence *Ĥ*_p·A_ can be obtained^80^ by a momentum boost with the amount of -z_*j*_**Â** for **p̂**_*j*_, then adding *H***^**_*ph*_. This result was first introduced in ref (76). This expression is general even beyond the long-wavelength approximation.

#### 2.3.2 The Dipole Gauge Hamiltonian

The QED Hamiltonian in the electric-dipole “d ·E” form^76,77,81^ (or so-called *dipole* gauge) can be obtained by performing the PZW transformation on *Ĥ*_p·A_ as follows
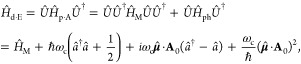
50where we have used eq 49 to express *Ĥ*_p·A_, and the last three terms of the above equation are the results of *ÛĤ*_*ph*_*U ^*^†^.

Using *q̂*_c_ and *p̂*_c_ (as defined in eq 43), one can equivalently express eq 50 as
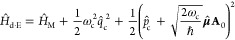
51

This can also be understood as the PZW operator boosting the photonic momentum *p̂*_c_ by .

The “d·E” Hamiltonian can also be viewed as effectively using the Poincaré gauge,^54^ where the vector potential under the Coulomb gauge upon PZW transformation gives the new vector potential **A**′_∥_(**r**) = −∇∫_0_^1^*du***r**·**A**_⊥_ (*u***r**) and **A′**_⊥_(**r**) = **A**_⊥_(**r**). Note that in this new gauge, the vector potential is *no longer purely transverse*.^54^ This choice of the vector potential^54^ makes **r**·**A**′(**r**) = 0. Thus, the Poincaré gauge enforces the vector potential to be perpendicular to the **r** vector everywhere (where the radial component of the vector potential is forced to be zero). The “d·E” Hamiltonian is often referred to as the dipole gauge^80^ (where beyond the dipole approximation should be referred to as the multipolar gauge^54^) or the length-gauge^53^ due to **μ̂** linearly depending on position.

The last term in eq 50 is commonly referred to as the dipole self-energy (DSE)^54^
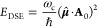
52which can be intuitively understood as the matter dipole polarizing the cavity field, and then the polarized cavity field acting back on the matter dipole, causing additional energy. Note that the DSE is different than the quadratic terms *z*_*j*_^2^**Â**^2^/2*m*_*j*_ in *Ĥ*_p·A_ (eq 45), which is commonly referred to as the **Â**^2^ term or diamagnetic term. Mathematically, the PZW gauge transformation operator shifts away (along the matter momentum direction) the **Â**^2^ terms in the p·A Hamiltonian, and causes a new shift (along the photonic momentum direction) that results in the DSE term in *H***^**_d·E_. Thus, the DSE is an essential component to make sure that *H***^**_d·E_ (eq 50) and *H***^**_p·A_ (eq 45) are gauge invariant. However, for small coupling strengths (*g*_c_ ≪ ω_c_ or (*E*_*e*_ – *E*_*g*_)/*ℏ*), ignoring the DSE term will not cause significant numerical errors, but will break the gauge invariance.^82^ This result depends on the proper truncation of modes such that when the Hamiltonian is truncated to a given set of modes, the DSE contribution only comes from those modes.^75^ For this section, we are only concerned with Hamiltonians under the single-mode approximation. The many modes scenario and the corresponding DSE expressions can be found in Section 2.6 and eq 101.

In the strong and ultrastrong coupling regimes, ignoring the DSE can cause an unstable ground state, especially under the long-wavelength approximation.^48^ As discussed at length in ref (48), the loss of the DSE term causes the ground state to be unbounded from below. Additionally, without the DSE term, the Maxwell equations in matter are no longer satisfied.^48^ In this manner, it is essential to include the DSE term in the strong and ultrastrong coupling regimes to accurately capture the physics of the system.

Under the classical limit, the d·E Hamiltonian can be obtained by applying the classical version of *Û* (eq 48), which is the Göppert-Mayer gauge transformation, on the classical p·A Hamiltonian in eq 35. The details can be found in ref (56) (page 73) or ref (57) (page 53). Interestingly, the classical version of the d·E Hamiltonian does not contain the dipole self-energy term. This is because the DSE arises as a consequence of the quantum commutation relation among field operators.^56^ In the semiclassical picture, the electric and magnetic fields are time-dependent potentials that commute with the semiclassical PZW operator
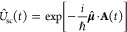
53

This *Û*_sc_ commutes with the electromagnetic fields, causing no boost of the photonic DOFs. By using the time-dependent Schrödinger equation, the linear d·E term forms due to the time dependence of *Û*_sc_ (*t*); however, since [*Û*_sc_ (*t*),**A** (*t*)] = 0, there is no DSE term in the semiclassical picture for the light–matter interactions. This has also been extensively discussed in ref (56) with a derivation for this semiclassical case starting on page 73. Additionaly, on page 231 of ref (56), Milonni states, “A difference between the classical or semiclassical derivations of the electric dipole form of the Hamiltonian from the minimal coupling form, compared to the approach here where the field is quantized, is worth noting: the [self-polarization] term···does not appear in classical or semi-classical derivations. It does not appear for the simple reason that the commutation relation between the vector potential and the transverse electric field is responsible for the appearance of [the self-polarization term]···If the field is not quantized, there is no such (non-vanishing) field commutator because the field variables are then c-numbers, not operators.”^56^

#### 2.3.3 The Pauli-Fierz QED Hamiltonian

The widely used Pauli-Fierz (PF) QED Hamiltonian (in the dipole gauge)^48,53,72^ in recent studies of polariton chemistry can be obtained by applying another unitary operator *Û*_0_ on *Ĥ*_d·E_. This unitary transformation is expressed as
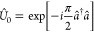
54

Note that *Û*_0_*â*^†^*âÛ*_0_^†^ = *â*^†^*â*, *Û*_0_*âÛ*_0_^†^*iâ*, and *Û*_0_*â*^†^*Û*_0_^†^ = *iâ*†. The PF Hamiltonian is related to *Ĥ*_d·E_ as follows
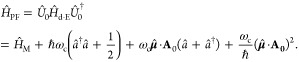
55

The PF Hamiltonian in eq 55 has the advantage of being a purely real Hamiltonian (under the long wavelength approximation).

Using the *q̂*_c_ and *p̂*_c_ operators (defined in eq 43), the PF Hamiltonian is expressed as
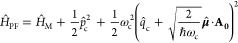
56

By comparing the above equation with eq 51, one can clearly see that the role of *Û*_0_ is to swap *p̂*_c_ with *q̂*_c_. In eq 56, *q*_c_ is displaced by . Note that another commonly used form of *Ĥ*_PE_ is with the negative sign of the photonic coordinate displacement
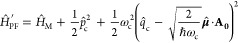
57which is the result of applying *Û*_0_’ = exp[-*i*π*â*^†^*â*] unitary transformation on *Ĥ*_PF_, with *Ĥ*_PF_′ = *Û*_0_′ *Ĥ*_PF_*Û*_0_′^†^. The role of *Û*_0_′ is causing a π phase shift for the photonic DOF and flip the sign of the *q̂*_c_ displacement from a positive one in *Ĥ*_PF_ to a negative one in *Ĥ*_PF′_.

From the form in eq 56, the photonic DOF can be viewed^53,72^ and computationally treated^83,84^ as an additional “nuclear coordinate”.^83,85,86^ This will be discussed further in Sec. 4.1.

#### 2.3.4 Consistency upon Gauge Transformation

We emphasize that both the *operators* as well as the *wave functions* should be *gauge transformed* through *Û*, in order to have a gauge invariant expectation value.^87^ This means that
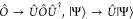
58such that the expectation value of any observable is invariant under any gauge
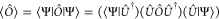
59

Even though this is a basic fact in quantum mechanics, historically, it has been overlooked in the quantum optics community,^87^ and has been extensively discussed in standard text books (e*.*g., see page 146 of ref (59)).

The argument in eq 59 should also apply to the photon number operator, which means that it should also be gauge transformed in order to provide a physical result. Under the Coulomb gauge, it is defined as
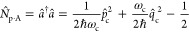
60

Under the dipole gauge, it should be
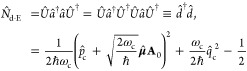
61where  =  =  =  + . For the PF Hamiltonian, the photon number operator should be
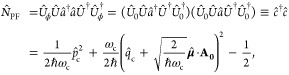
62where the corresponding gauge transformed raising operator becomes
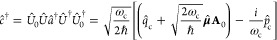
63and the physical number operator is
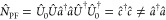
64

This has been pointed out extensively in recently works in refs (82) and (51). Using the incorrect expression *â*^†^*â* under the dipole gauge will *overestimate* the actual photon number,^51^ causing inaccurate and misleading results.

### 2.4 Hamiltonians in Truncated Hilbert Spaces

Investigating cavity QED dynamics often requires a truncation of electronic states applied to the QED Hamiltonians.^80,88^ This is because these matter electronic states are often difficult to obtain, and in a lower energy regime, one can project the QED Hamiltonian to a few physically relevant electronic states without losing significant accuracy. Consider a finite subset of electronic states {|α⟩} where there is a total of  matter states, eq 26 can be rewritten to define the projection operator
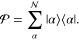
65

To make the discussion more general, the state |α⟩ is not necessarily the adiabatic state used in eq 26. As discussed in Section 2.1,  defines the truncation of the full electronic Hilbert space  which has infinite dimension, to a subspace  that contains a total of  states. This truncation reduces the size of the Hilbert space of the entire problem from the original space, , to , where  and  represent the identity operators of the nuclear and the photonic DOF, respectively.

#### 2.4.1 Gauge Ambiguities

Truncating the momentum operator and dipole operator as  and , the p · A Hamiltonian under the truncated subspace are commonly defined as
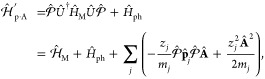
66whereas the d·E Hamiltonian under the truncated subspace is commonly defined as^52,80^
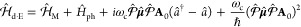
67

It is well-known that the above two Hamiltonians do not generate identical polariton eigenspectra^52,80,88,91−95^ under the ultrastrong coupling regime,^47^ explicitly breaking down the gauge invariance. This leads to the gauge ambiguity^87,88,96^ as to which Hamiltonian,  or , is correct for computing physical quantities when applying . This is a well-known result in quantum optics^84,88^ that  usually requires a larger subset of the matter states to converge or generate consistent results with , and apparently, under the *complete* basis limit, they should be gauge invariant. For clarity, we reiterate that the gauge ambiguities mentioned in this review only refer to different eigenspectra obtained from different gauges due to the same level of truncation (defined by  in eq 65). As such, the size of the projected Hilbert space must still be treated as a convergence parameter to produce accurate results.

**Figure 2:**
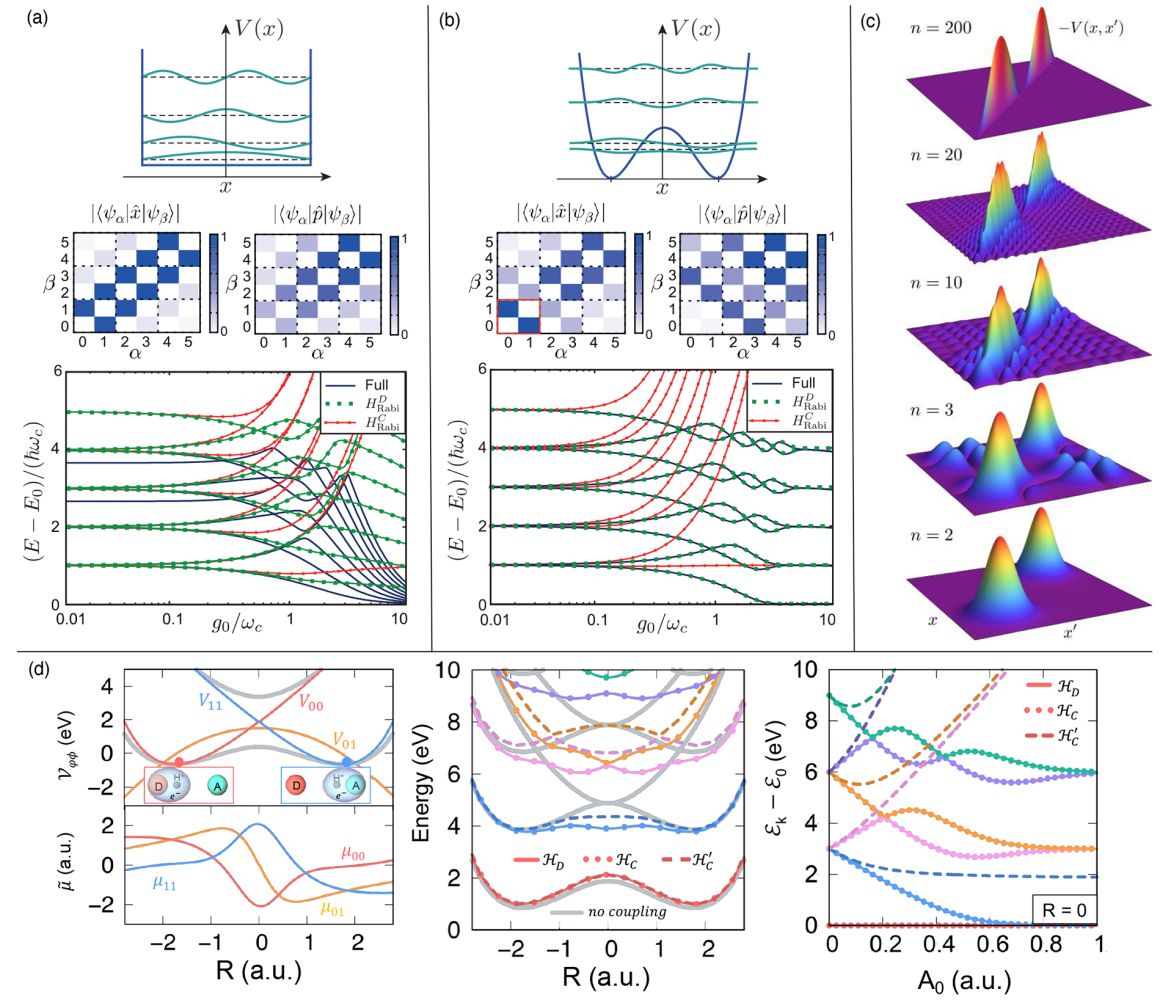
Gauge ambiguities and the recently proposed resolutions. (a) Demonstration of gauge ambiguities when an electron in a 1-D square potential is strongly coupled to a cavity whose frequency is resonant to the electronic transition from the ground state to the first excited state. Electronic matrix element magnitudes shown for the coordinate *x̂* and its conjugate momentum, *p̂*. Note that the coordinate matrix is significantly more diagonal than the momentum matrix. The bottom panel shows the eigenspectra of the Coulomb (*H*_Rabi_^C^) and dipole (*H*_Rabi_^D^) gauges truncated to two levels compared to the full basis limit. The stark disagreement between to two gauges demonstrates the gauge ambiguities. For this model, the two level approximation is not a terribly good approximation. (b) This repeats the analysis for panel (a) for a double well potential. For this model, the two-level approximation is valid. This shows how for a valid level of truncation the dipole gauge results match very well with the full space results. (c) Demonstration of nonlocal potentials, *V*(*x*, *x*′), that form upon a finite *n*-level truncation of the electronic Hilbert space. In the infinite basis limit, *V*(*x*, *x*′) → *V*(*x*) and is completely local. As *n* decreases, the potential becomes increasingly nonlocal. These numerical results are for an electron in a double well potential (similar to panel (b)). (d) Proposed resolution to the gauge ambiguities discussed in panels (a–c) for molecular systems. For a simplified 1-D proton and electron transfer model, the eigenspectra of three two-level truncated polaritonic Hamiltonians under the Born–Oppenheimer approximation are compared: the truncated dipole gauge Hamiltonian , the naively truncated Coulomb gauge Hamiltonian , and the newly proposed properly truncated Coulomb Hamiltonian . The properly truncated Coulomb Hamiltonian perfectly matches the results calculated in the dipole gauge. Panels (a) and (b) are adapted with permission from ref (88). Copyright 2018 American Physical Society. Panel (c) is adapted with permission from ref (89). Copyright 2020 American Physical Society. Panel (d) is adapted with permission from ref (90). Copyright 2020 American Physical Society.

The fundamentally different behavior of  and  upon matter state truncation is attributed to the fundamental asymmetry of the **p̂** and **μ̂** = ∑_*j*_ z_*j*_**x̂**_*j*_ operators.^88^ This can be more clearly seen when considering just a single electron confined in a 1D potential *V̂*(*x̂*) (such that *Ĥ*_M_ – *Ĥ*_el_ = 0, since there is no nuclear DOF), where *Ĥ*_M_|α⟩ = *E*_*α*_|α⟩. Under the energy representation {|α⟩}, the matrix elements of the position operator *x*_*αβ*_ = ⟨α|*x̂*|β⟩ satisfy the following well-known Thomas-Reich-Kuhn (TRK) sum rule
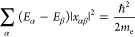
68where *m*_e_ is the mass of the electron. This means when (*E*_*α*_ – *E*_*β*_) is larger (for well-separated energy levels), |*x*_*αβ*_| will be smaller in order to satisfy the TRK sum rule. This can be clearly seen in the middle panels of Figure 2a,b, where the largest matrix elements for *x*_*αβ*_ only show up for nearest neighbor energy levels. Thus, in the energy representation, *x̂* is “local” in the sense it only strongly couples the |*E*_*α*_⟩ and |*E*_*β*_⟩ energy levels when their energies are close. The transition dipole operator for a single electron is μ̂ = −*x̂* (where the fundamental charge of the electron is *z* = −1), and thus *μ*_*αβ*_ = −*x*_*αβ*_. This explains why  often gives accurate numerical results of polariton eigenvalues, due to the fact that μ̂ behaves locally in the energy space and thus truncation is often a valid approximation. The matrix element of the momentum operator *p*_*αβ*_ = ⟨α|*p̂*|β⟩, on the other hand, is related to *x*_*αβ*_ as follows
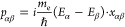
69

Thus, the momentum operator behaves in a “non-local” fashion in the energy representation, because (*E*_*α*_ – *E*_*β*_) can get very large even when the corresponding *x*_*αβ*_ is small. This behavior can be seen from the middle panels of Figure 2a,b, where the large amplitudes of *p*_*αβ*_ exist among states |α⟩ and |β⟩, even when (*E*_*α*_ – *E*_*β*_) is large. Rabl and co-workers^88^ argue that this why  behaves less accurately upon matter state truncation due to the nonlocal behavior of the coupling term  in eq 66. Thus, the large energy gaps in molecular systems do not guarantee small matrix elements of the *p̂* operator,^88^ hence a finite-level truncation in the “p·A” Hamiltonian often leads to large numerical errors. Hence, it is often more convenient to use the dipole gauge when applying the finite-level approximation for the matter DOFs.^88^ Note that such an asymmetry in the *x̂* and *p̂* operators disappears for the quantized electromagnetic mode or for a harmonically bound dipole, where momentum and position operators are interchangeable.^88^ However, when the molecular potential is highly anharmonic, the gauge invariance is explicitly broken under the finite-state approximation,^80^ for  (eq 66) and  (eq 67), due to the lack of a complete basis.

Figure 2a,b demonstrates the breakdown of gauge invariance^88^ between  (eq 66) and  (eq 67) for model systems with a square and double well potential, respectively. In both models, the energy eigenspectra using each gauge (eqs 66 and 67) for an election in a given potential is plotted as a function of coupling strength when truncated to only two matter levels, and the matrix elements of *x̂* and *p̂* are visualized. In Figure 2a, these results are shown for a square potential. In this case, the dipole gauge results outperform Coulomb gauge results but still fails to capture much of the physics of the full system. This is a consequence of the locality of *x̂* and *p̂* in the energy picture, shown by the matrix element visualizations in Figure 2a. The position matrix elements are much more localized than the momentum matrix elements. However, a two level truncation is still not a good approximation, since |⟨ψ_1_|*x̂*|ψ_2_⟩| matrix elements are significant, meaning that at least three states are needed to accurately describe the first two states. These results can be contrasted with those of Figure 2b, where the potential is a double well potential. In this case, the matrix elements of *x̂* are more localized for the first two levels, such that it can be well approximated as a two-level system. For the momentum matrix elements, however, the first two states are strongly coupled to many high energy states. This disparity is apparent in the energy eigenspectra from the dipole and Coulomb gauges. The dipole gauge results follow the fully converged results, while the Coulomb gauge results diverge.

In the truncated electronic basis, the PF Hamiltonian  is expressed as
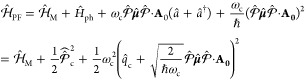
70

Note that *Û*_0_ (eq 54) is only a function of the photonic DOF, thus it does not bring any matter operator that was originally confined in  to . Hence,  provides consistent results from , ensuring no ambiguities from truncation between  and .

#### 2.4.2 Proposed Causes and Resolutions of Gauge Ambiguities

In recent literature,^75,80,89−91,97^ the source of these gauge ambiguities and corresponding resolutions (see Figure 2a,b) has been thoroughly discussed from both an intuitive physical perspective^80,89^ and a rigorous mathematical perspective.^75,89−91,97^

In refs (80, 89), Stefano et al. and Garziano et al. describe the source of gauge ambiguities in terms of the locality of the matter potential energy operator in the truncated Hilbert space, . In other words, upon matter truncation to a finite basis, the potential energy operator is defined based on two positions in space, hence it is no longer local in space (only depending on *x*). Equivalently, by Fourier transforming in *x̂*′, one can say that this operator is dependent on both the position and momentum operators. Figure 2c shows how for an *n*-level matter truncation,  gets increasingly nonlocal as *n* shrinks. Refs (80, 89) argue that this nonlocality leads to gauge ambiguities since the expression in eq 66 contains the nonlocal potential, , to which the gauge transformation has not been applied (due to the fact that *V̂*() commutes with *Û*). This can be seen by rewriting eq 66 as,
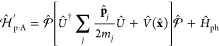
71where **x̂** = {**x̂**_*j*_}. To fix this problem, it was proposed to first truncate *Ĥ*_M_ and then transform it by the projected PZW operator , which will gauge transform the nonlocal potential . However, ref (91) points out that this does not formally solve the gauge ambiguities. Instead, it works specifically when the matter is truncated to a two level system, making the proposed solution just a rotation on a Bloch sphere.

In refs (75, 90), Taylor et al. go further to propose a general resolution to gauge ambiguities for any matter system under the dipole approximation. The key insight discussed in these works is the concept of proper confinement of all operators in the truncated subspace. For a given projection operator, , there is a complementary operator, , such that . Taylor et al. describe a new gauge theory that is “properly contained” in the subspace defined by . In other words, all the information on the truncated system lives entirely in the  in the  subspace. For example, consider the case of  =  ≠ . In this manner,  is not properly confined in , since it contains *x̂* information from the  subspace, .

This concept of proper confinement is then used to resolve gauge ambiguities by ensuring that any two arbitrary gauges can be connected through unitary transformations within the  subspace. For either the dipole or Coulomb gauge in the full Hilbert space, the truncated analog can be formulated in four steps. First, represent the full space Hamiltonian in terms of *Ĥ*_M_, *Ĥ*_ph_, and *Û* (as done in eqs 49 and 50). Second, truncate *Ĥ*_M_ and *Ĥ*_ph_ in their eigenbases. Third, redefine the PZW operator, *Û*, to be properly confined in the  subspace in terms of **x̂** and **p̂**. This can be done by applying the projection operator inside the exponential of *Û* as follows
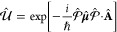
72

As discussed in ref (75), this idea can be generalized to any kind of truncation of a Hilbert space, even for those going beyond just material truncation. For example, the gauge-transformation operator can also be constructed for cavity photonic mode truncation, where the projection operator  will also include the cavity mode truncation (by projecting out the corresponding Fock states of those truncated modes, except for the group Fock state). For that case, the most general expression of  becomes^75^
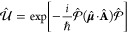
73where  enforces both matter and photonic Hilbert space projection. An example of the mode truncation related  can be found in eq 95, which also contains the photonic operators and thus needs to project **μ̂·Â** all together.^75^ When  only contains projections on the electronic DOF of the matter, eq 72 and eq 73 are equivalent.

Finally, one can reconstruct the full Hamiltonians using the forms from eqs 49 and 50 and the truncated operators, , , and  (from eq 73). The properly truncated Coulomb gauge Hamiltonian takes the form
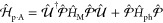
74

By ensuring proper confinement of all operators, this method strictly ignores any information from the  subspace. The  operator is also strictly unitary in its own Hilbert subspace, so the gauge invariance between the dipole and Coulomb gauges is ensured. There are scenarios where the Coulomb gauge is more convenient^98−102^ than the dipole gauge for describing light–matter interactions, such as for a solid state material^98,99,102^ interacting with the radiation field where the wave function satisfies periodic boundary conditions and the expectation value of the dipole operator becomes ill-defined.^103^ For these scenarios, instead of using , the currently derived  should be used to investigate the light–matter interactions. Compared to  which requires many electronic states to provide a reasonable polariton eigenspectrum,^88,99,104^ requires as few electronic state as *Ĥ*_D_ and provides identical results.

The properly truncated Coulomb gauge Hamiltonian in eq 74 can then be explicitly written for molecular systems as
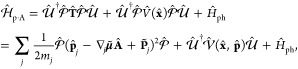
75where  is the residual momentum and  is the truncated dipole operator.^90^ Note that (eq 66) as well as *Ĥ*_p.A_ (eq 45) only contain the vector potential **Â** up to the second order. This is no longer the case for  in eq 75. In fact, both the **P̃**_*j*_ term and the  term in principle contain infinite orders of **Â**. Hence, the consequence of level truncation on *Ĥ*_p·A_ is not just simply modifying the matrix elements of the momentum operator (as incorrectly indicated by  in eq 66), but rather profoundly changing the structure of light–matter interactions^80^ through both the new potential  as well as the new momentum shift -∇_*j*_**μ̃Â** + **P̂**_*j*_, due to the mixing of the light and the matter DOFs through  and  in the truncated subspace. It is clear that (eq 76) will return to *Ĥ*_pA_ (eq 45) under the complete electronic basis limit, such that , thus ∇_*j*_**μ̃** → ∇_*j*_**μ̂** = *z*_*j*_, hence **P̃**_*j*_ → 0, as well as *Û* → *Û*, hence . In ref (97), Gustin et al. further generalizes the resolution of gauge ambiguities beyond the dipole approximation by defining *Û* in terms of the full matter polarization instead of the dipole operator. They then properly confine  by truncating the polarization operator in terms of **x̂**. Unfortunately,  in eq 75 no longer remains in the minimum coupling form in eq 45 which only involves charges but not higher multipole moments. Of course, when approaching the complete electronic states limit, the minimum coupling form is restored. Nevertheless,  is invariant from  through the  transformation, thus resolving the ambiguity between them.

#### 2.4.3 Molecular QED Hamiltonian in the p·A form

Going back to the molecular cavity QED Hamiltonian, by splitting the matter Hamiltonian as *Ĥ*_M_ = **T̂**_**R**_ + *Ĥ*_el_ (see eq 20), one can express eq 75 as follows
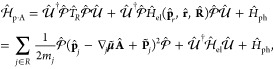
76where the sum over *j only* includes nuclei. In the above expression, we did not specify the choice of , which could be either adiabatic (eq 26 or diabatic states (eq 31).

Figure 2d shows numerical results for this Hamiltonian for a simple 1-D proton-transfer (Shin-Metiu^105^) molecular model. The left graph shows a characterization of this model with its adiabatic and diabatic states, diabatic coupling, and dipole matrix elements as a function of the proton’s 1-D coordinate, *R*. Additionally, the small insets pictorially depict the ions, proton, and electron positions for different *R*. The middle figure then plots the Born–Oppenheimer surfaces as a function of *R* for different Hamiltonians, compared to the zero coupling case. For *R* values where the polariton states differ from the uncoupled case, the naively truncated Coulomb gauge Hamiltonian results differ from the gauge invariant results. The right figure, similarly, shows how the naively truncated Coulomb gauge Hamiltonian behaves very poorly as the coupling strength is increased for a given *R* value. For this model, the dipole gauge results converge to the accuracy of the graph with two levels, so for the results in these graphs, the dipole gauge can be considered “exact” for this model. This numerically demonstrates the necessity of maintaining gauge invariance.

There are several interesting limits of (eq 76). Under the limiting case when **A**_0_ = 0 or **μ̃Â** = 0, both the −∇_*j*_**μ̃Â** and **P̃**_*j*_ terms become 0, and . Thus, under a such limit, ; hence, the matter and the cavity becomes decoupled. When using adiabatic states for the truncation, one can show that^60,61^ = , where **d**_*αβ*_^*j*^ ≡⟨α|∇_*j*_ |β⟩ is the well-known derivative coupling. Besides these adiabatic derivative couplings, the light–matter interaction also induces additional “derivative”-type couplings, −∇_*j*_**μ̃Â** and **P̃**_*j*_, regardless of the electronic representation used in constructing . When using the Mulliken-Hush diabatic states^63,64^ which are the eigenstates of the  operator, such that **μ̃**= ∑_*ϕ*_*μ*_*ϕϕ*_ |ϕ⟩⟨ϕ|, one can prove that **P̃***_j_* = 0 for all nuclei. This is because that ∇*_j_***μ̃** = ∑*_ϕ_*∇_*j*_*μ*_*ϕϕ*_ |ϕ⟩⟨ϕ|, thus both **μ̃Â** and [**μ̃Â**,**p̂***_j_*] become purely diagonal matrices, hence all of the higher order commutators in  become zero, resulting in **P̃***_j_* = 0 for *j* ∈ **R**. Unfortunately,  no longer remains in a minimum coupling form in eq 45 (except when approaching the complete electronic states limit), by only involving charges but not higher multipole moments. Nevertheless,  is invariant from  through the  transformation, thus resolving the gauge ambiguity between them.

Additionally, this method explains why the proposed resolution of ambiguities in ref (80). only works for matter systems that can be well approximated by two-level systems without a permanent dipole. For those types of systems, the truncated dipole operator is proportional to the Pauli *σ̂*_*x*_ matrix, and , where **μ***_eg_* is the transition dipole from the ground state to the excited state. In this special case, . Then, the properly truncated PZW operator is .

In ref (90), the closed analytic formalism for arbitrary two-level molecular systems is presented. Without the loss of generality, such a system can be expressed in terms of the diabatic states {|0⟩, |1⟩}, which represent a broad range of chemical systems.^106−108^ To simplify the algebra, one assumes there is only one nuclear DOF with the coordinate *R̂* and momentum *p̂*_*R*_, and **μ̂** is always aligned along the polarization direction **ê**. Note that both the transition and permanent dipoles are functions of *R̂*.

In this special case, the properly truncated PZW operator becomes,
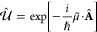
77where  and **μ̃**’s explicit dependence on *R̂* is suppressed in this notation for clarity. Since **μ̃** can be written as a sum of Pauli matrices, evaluating  and ***P̃****_j_* becomes tractable using the Pauli matrix commutator relations.

The electronic Hamiltonian in this truncated subspace is , where  = ,  = , and  =  (i.e., they are *Ĥ*_el_’s matrix elements). Using the above spin representation for μ̃ and *Ĥ*_el_, as well as the BCH identity, one can analytically show (ref (90)) that the terms in  from eq 76 are
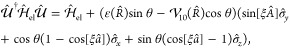
78where  = , tan θ = 2μ_01_/(μ_00_ – μ_11_), and the residual momentum is  =  –  +  – . Thus, for a given  and **μ̃**(*R*), under a two-level approximation, the properly truncated Coulomb gauge Hamiltonian can be written in this analytic form.

### 2.5 Connections to Quantum Optics Models

In quantum optics, a two-level atom coupled to a single mode in an optical cavity is a well-studied subject. This setup has been described using well-known model Hamiltonians, such as the quantum Rabi model^109,110^ and the Jaynes-Cummings model.^25^ Since these two models are also widely used in recent investigations of polariton chemistry, here we briefly derive them from the truncated Pauli-Fierz Hamiltonian (eq 70). The original derivations^25,109,110^ of these two models are slightly different than the procedure outlined here, but the general physical insights are the same.

We consider a molecule with two electronic states and consider its electronic Hamiltonian as
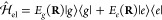
79such that the transition dipole is **μ̂***_eg_* = ⟨*e*|**μ̂**|*g*⟩. Note that the permanent dipoles in a molecule **μ̂***_ee_* = ⟨*e*|**μ̂**|*e*⟩, **μ̂***_gg_* = ⟨*g*|**μ̂**|*g*⟩ are not necessarily zero, as opposed to the atomic case where they are always zero. Hence, it is *not always* a good approximation to drop them. The breakdown of the quantum optics models for computing polariton potential energy surface will be discussed in Section 3.1.3

The Rabi model assumes that one can ignore the permanent dipole moments (PD), and leads to the dipole operator expression in the subspace  as follows
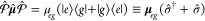
80where we have defined the creation operator σ̂^†^ ≡ |*e*⟩⟨*g*| and annihilation operator σ̂ ≡ |*g*⟩⟨*e*| of the electronic excitation. The PF Hamiltonian (eq 55) in the subspace  thus becomes the following  with no permanent dipole (nPD)
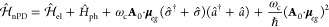
81

Dropping the DSE (the last term) in eq 81 leads to the quantum Rabi model as follows
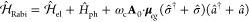
82

The exact solution of the quantum Rabi Hamiltonian *Ĥ*_Rabi_ was first discovered by Braak^111^ by noticing the parity symmetry in the Rabi model is sufficient to solve the Hamiltonian exactly using bosonic operators in the Bargmann space.^111^ Later, it was shown that the same solution can also be obtained from the Bogoliubov transformation.^112^

Dropping both the DSE and the counter-rotating terms (CRT) σ̂^†^*â*^†^ and *σ̂â* leads to the well-known Jaynes-Cummings (JC) model^25^ as follows
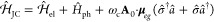
83which is eq 2 in the Introduction (Section 1.1) when choosing *g*_c_ = ω_c_**A**_0_ · **μ***_eg_*.

As we go beyond these simplified Hamiltonians, however, the most physically relevant coupling parameter becomes ambiguous. The dipole operator is no longer expressed as , and instead takes the form of an arbitrary Hermitian matrix as indicated in eq 28 (for adiabatic basis) or eq 29 (for MH diabatic basis),

Of course, the JC model and the Rabi model, which are motivated to describe two-level atoms interacting with a single-mode cavity, will eventually break down with an increasing light–matter coupling strength. For atomic cavity QED, the light–matter coupling constant is *g*_c_ = ω_c_**A**_0_ · **μ***_eg_*/*ℏ*. For comparative purposes, one often uses the unitless coupling parameter defined as
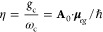
84

Under the condition η < 0.1, the JC model provides a reasonably accurate answer compared to the “exact” answer provided by (under the single molecule, single mode, and long wavelength approximations, without any permanent dipole). For the ultrastrong coupling regime 0.1 < η < 1, or deep-strong coupling regime η > 1, the JC model starts to break down. A detailed discussion of this breakdown can be found in ref (47). Interestingly, in the ultrastrong coupling regime, the JC model actually predicts more accurate results compared to the Rabi model because the DSE term ω(**A**_0_·**μ***_eg_*)^2^ in (eq 81) partially cancels with the energy shift (commonly referred to as the Bloch-Siegert shift^113,114^) caused by the counter-rotating wave terms σ̂^†^*â*^†^ and *σ̂ â*. A detailed analysis can be found in ref (12), as well as in ref (115). Interestingly, one can define unitary gauge transformation that depends on the coupling strength, such that the JC model (under this gauge transformation) remains reasonably accurate throughout different ranges of coupling strength.^92^

**Figure 3:**
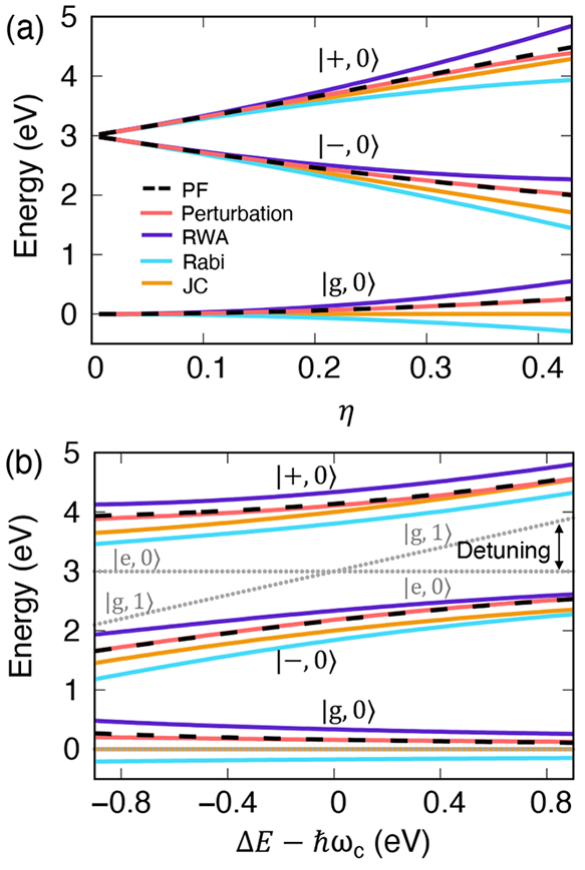
Polariton eigenspectrum of a two-level system coupled to cavity using various light–matter Hamiltonians. Polariton eigenspectrum (a) as a function of η = *g*_c_/ω_c_ at zero detuning Δ*E* – *ℏω*_c_ = 0 and (b) as a function of the detuning Δ*E* – *ℏω*_c_ at *ℏg*_c_ = 1.0 eV obtained with various levels of theory, including the exact solution of PF Hamiltonian (black dashed), JC Hamiltonian (yellow) that assumes RWA and ignores DSE, Rabi Hamiltonian (cyan) that ignores DSE, RWA (magenta) that ignores counter-rotating term (CRT), and Perturbation theory (PT) (red) which treats CRT perturbatively. Adapted with permissions from ref (12). Copyright 2020 American Chemical Society.

Figure 3 presents the three lowest polariton eigenenergies of a two-level atom (eq 79 without any nuclear DOFs) coupled to a single mode cavity. The figure presents three polariton states |g, 0⟩, |−,0⟩ and |+,0⟩. Figure 3a presents the polaritonic eigenvalues as a function of η = *g*_c_/ω_c_ at Δ*E* – *ℏω*_c_ = 0 (resonance condition). Figure 3b presents the polaritonic eigenvalues as a function of the detuning Δ*E* – *ℏω*_c_ with a light–matter coupling strength *ℏg*_c_ = 1 eV. The eigenenergies are obtained at various levels of theory, including the JC model (yellow) in eq 83 that ignores both CRT and DSE, the rotating wave approximation (RWA) Hamiltonian (magenta) that only ignores the CRT term but not the DSE term, the Rabi model (cyan) in eq 82 that ignores the DSE, and the full PF treatment (black dashed) in eq 70 that includes both the CRT term and DSE. The perturbation theory (PT) (red) which treats CRT perturbatively (see details in ref (12)) and includes the exact DSE and provides very accurate polariton eigenenergies in the range of the parameter regime investigated here.

In the JC model Hamiltonian (yellow), the ground state does not shift with increasing η, while the |+,0⟩ and |−,0⟩ states linearly split as a function of η. This behavior can be easily understood by examining the JC eigenspectrum in eq 5. The Rabi model (cyan), which only accounts for the CRT, overestimates the negative energy corrections and incorrectly decreases energies for all states. Thus, the Rabi model predicts that the ground state energy becomes unstable. The RWA Hamiltonian (magenta), which ignores the CRT but includes the DSE, overestimates the energy correction in the positive direction and shifts all states upward. The perturbative treatment (red) that includes CRT as a perturbation as well as the DSE performs well and is nearly identical to the exact PF curve within the range of the η or *ℏ*Δω_c_ presented here. Note that in Figure 3b, for *ℏ*Δω_c_ < – 0.5 eV, the polariton eigenenergy for |−,1⟩ becomes lower than |+,0⟩. As a result, a trivial crossing is formed between the third and fourth polaritonic eigenenergies as a function of *ℏ*Δω_c_ at *ℏ*Δω_c_ ≈ – 0.5 eV.

When dealing with the full molecular cavity QED situation, where both the permanent and transition dipoles (eq 28) need to be considered, the coupling strength *g*_c_ or η (eq 84) no longer accurately describes the systems because it only includes a particular value of the transition dipole, whereas both transition and permanent dipoles could change their values significantly as a function of the nuclear coordinate in a real molecular system (see example in Figure 6b). For this case, typically two different expressions for coupling parameters are used in the literature, either the magnitude of the vector potential, ,^14,75,90,116,117^ or a coupling parameter that does not explicitly depend on the cavity frequency^53,72,82,83,115,118−125^
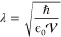
85

On the other hand, one should be careful because these coupling parameters do not include the magnitude of the dipole, either *μ*_*αα*_(**R**) or *μ*_*αβ*_(**R**), and both values could vary significantly by changing **R** for a given system. These values also need to be included when judging if a system is under a particular coupling strength.

Further, it should be noted that for Fabry–Pérot cavities, the area of the mirrors is typically considered constant when comparing different frequencies. In this case, the cavity volume is inversely proportional to cavity frequency, and *A*_0_ would be independent of frequency, while λ would be frequency-dependent. For the majority of this review, these two parameters are used to represent coupling strength.

Finally, even with the considerations of a single molecule coupled to the single cavity mode under the dipole approximations, we want to emphasize that the accuracy and validity of JC and Rabi models need to be carefully assessed before adapting them to the field of molecular cavity QED. This is because these models only consider two electronic states {|*g*⟩, |*e*⟩} and the transition dipole **μ***_ge_*(**R**) between them, where the permanent dipole is often ignored. Unfortunately, these well-established approximations in the atomic cavity QED can explicitly break down for molecular cavity QED systems.^6,126,127^ A detailed example of the breakdown of these models is provided in Figure 6 of Section 3.1.3.

### 2.6 Many Molecules Coupled to Many Cavity Modes

**Figure 4:**
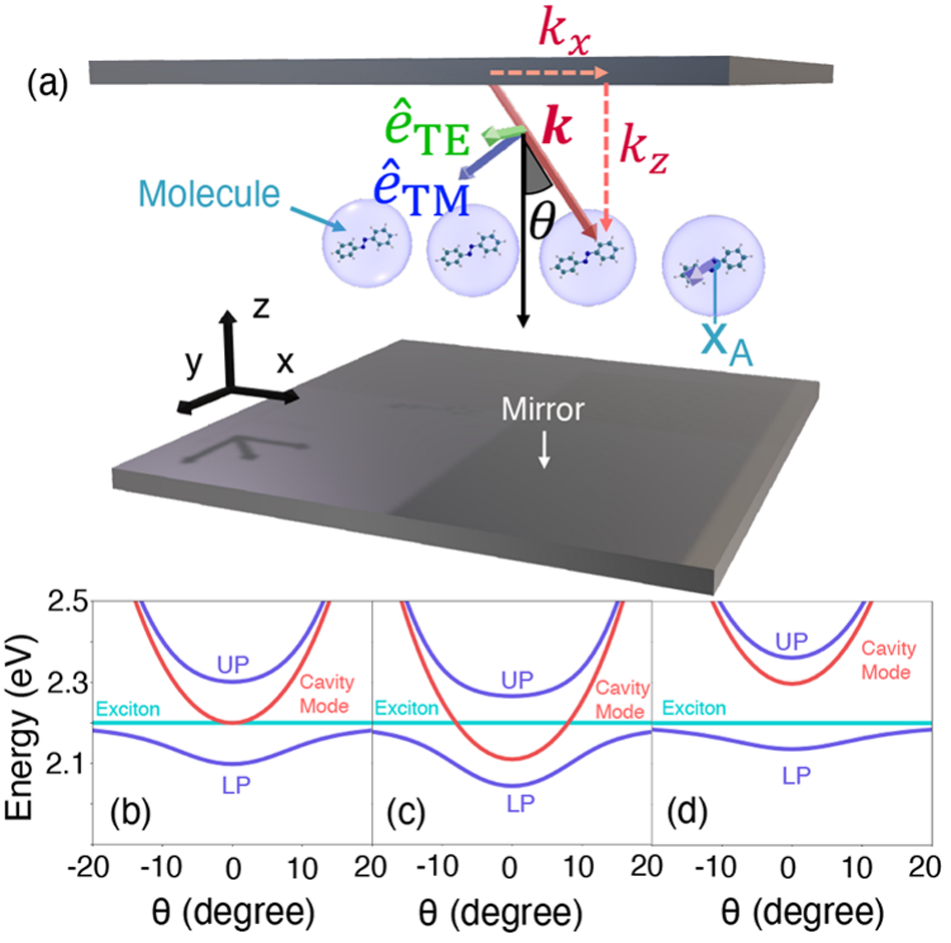
Many molecules and cavity modes. (a) Schematic of many colinear molecules in a Fabry–Pérot (FP) cavity. **ê**_TE_ and **ê**_TM_ are the unit vectors indicating the directions of the Transverse electric (TE) and Transverse magnetic (TM) polarized components of **Ê**_⊥_, respectively. (b-d) Schematic dispersion for zero detuning (b), positive detuning (c), and negative detuning (d). Plot of the upper and lower polariton states in a FP cavity (purple solid) as a function of the incident angle (θ) with the bare cavity dispersion (red lines) and the exciton dispersion (blue lines).

In the previous sections, we focused on the QED Hamiltonians under the long wavelength approximation and the single photonic mode approximation. However, these approximations are not adequate to accurately describe experiments conducted with Fabry–Pérot cavities.^1−4,17−20,37,39−41,128−131,131−137^ In this manner, we must start with the most general Hamiltonian in eq 35 and derive the convenient expressions for model Hamiltonians that can accurately describe many molecules interacting with many cavity modes. Specifically, many modes are considered with many molecules, and we partially relax the long wavelength approximation such that **Â** is no longer spatially invariant while the matter interactions are still approximated as dipoles. Such a Hamiltonian is necessary to describe many molecules coupled to a Fabry–Pérot cavity, depicted in Figure 4a. In that situation, we explicitly consider a 1-D array of molecules.^138^ Several useful review articles related to this topic can be found in ref (139).

In Fabry–Pérot cavities, the total wavevector of the photon can be decomposed into a component that is perpendicular to the cavity mirror, which we denote as *k*_*z*_
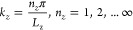
86

The value of *k*_*z*_ is explicitly quantized, due to the boundary condition imposed by two mirrors, where *L*_*z*_ is the distance between the two mirrors. In the literature,^40,128^*k*_*z*_ is often denoted as *k*_⊥_ because it is perpendicular to both mirrors (not to be confused with the transverse component of the field in eq 37a). There are two more degenerate wavevectors, *k*_*x*_ and *k*_*y*_, with their directions parallel to the mirror, and are commonly denoted as *k*_∥_ in the literature (not to be confused with the longitudinal component of the field, such as eq 36). Both *k*_*x*_ and *k*_*y*_ are in principle, quasi-continuous, because the boundary length for the lateral directions (*x* and *y* in Figure 4) are generally much larger than the mirror distance *L*_*z*_. The cavity quantization volume is , where *S* represents the effective quantization area at which molecules are coupled to the cavity. Using the experimentally measured Ω_R_ and , one can estimate how many molecules *N* are effectively coupled to the cavity.^40^

Overall, this leads to many photonic modes that can be energetically close to a matter state transition, such as electronic excitations^40,138−144^ or vibrational excitations.^1,4,17,131,137,145,146^ For these cavities, the photonic dispersion relations are the same for both the transverse electric (TE) and transverse magnetic (TM) polarizations, and experimentally, one can easily access both.^144,147,148^

For simplicity, let us focus on the TE mode, and set *k*_*y*_ = 0. For a field propagation direction **k** (see Figure 4), the total energy of the photon is
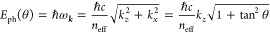
87where *c* is the speed of the light, *n*_eff_ is the effective refractive index inside the cavity, and θ is the angle of ***k*** from the normal of the mirror (see Figure 4a). This angle θ is often referred to as the “incident angle” of the photon, which is tan θ = *k*_*x*_/*k*_*z*_. When θ = 0, we have
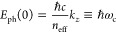
88where ω_c_ is the photon frequency of the quantized direction (*z*-direction) in the cavity, used in the single mode approximation of the cavity QED (see eq 42, eq 44, and eq 56 in Section 2.3). Further, under the single mode approximation (by setting *k*_*x*_ = 0) the photonic momentum **k** (or the field propagation direction) will be perpendicular to the cavity mirror.

Note that in principle, the Fabry–Pérot cavity has an infinite set of possible *k*_*z*_ that satisfy the mirror boundary conditions (eq 86). Often, one only considers the *k*_*z*_ that is close to the matter excitation energy. However, when *E*_ph_ is much smaller than the matter excitation energy, multiple modes that contain various *k*_*z*_ (eq 86) in the range of matter energy and a given range of θ have to be considered.^140,143^ In this review, we only consider the case for a single *k*_*z*_ (such that *k*_*z*_ = π/*L*_*z*_).

Hence, in the regime of small incident angles, the cavity photon energy can be approximated as
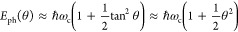
89which is the usual quadratic dispersion relation observed in the experiments.^40,139,144,149^ On the other hand, the matter energy is considered to be invariant in the typical range of the angles θ measured in the experiments, and thus *E*_M_ = *ℏω*_*eg*_ + *E*_*g*_ = *E*_*e*_, where *ω*_*ge*_ = (*E*_*e*_ – *E*_*g*_)/*ℏ*. If one considers θ as a parameter (under the continuous limit of *k*_*x*_), and the Tavis-Cummings model to describe light–matter interactions (see Section 1.2), one can then write down the following two-by-two matrix for polariton Hamiltonian in the {|*G*, 1⟩, |*B*, 0⟩} subspace^139^
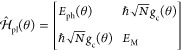
90

The diagonal terms are *E*_ph_ (θ) (red parabolic curves in Figure 4b–d) and *E*_M_ (cyan straight-line in Figure 4b–d), and the coupling term  causes the “band bending” when the matter and photon dispersion branches intercept. Note that *g*_c_ (θ) picks up a θ dependence from the cavity dispersion relation of ω_**k**_ (eq 87).

Figure 4b–d shows examples of this θ dependence for a Fabry–Pérot cavity for the situation of (b) zero light–matter energy detuning, (c) positive detuning, and (d) negative detuning, where the polariton dispersion curves are depicted in purple. For each **k** (that corresponds to a specific θ or *k*_*x*_), the model Hamiltonian in eq 90 is diagonalized to find the dispersion plots. Similarly, the polariton eigenenergies are now functions of **k** (or equivalently, θ) as follows
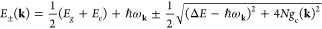
91

The dispersion plots in Figure 4(b–d) plot these eigenenergies for different *k*_*z*_ values (corresponding to the frequency for θ = 0). The corresponding quantum eigenvectors for the |±⟩ polariton states are
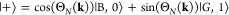
92a
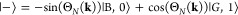
92bwhere the mixing angle
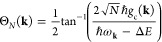
93explicitly depends on the wavevector, according to the dispersion relation in eq 87. The expansion coefficients for the states in eq 92 are often referred to as the Hopfield coefficients^150^ which indicate the character of polariton states^40,149^
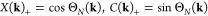
94where *X*(**k**)_+_ is the exciton character and *C*(**k**)_+_ is the photonic character of the |+⟩ state.^149^

Note that for Fabry–Pérot cavities, ω_***k***_ is polarization independent, so typically only the TM mode is considered. We emphasize that for a *plasmonic* cavity, eq 87 no longer always holds. For example, the plasmonic cavity^151,152^ has a similar dispersion for the TM polarization , but a linear dispersion for the TE mode , where *a*_*x*_ is the lattice constant in the *x*-direction for the plasmonic lattice and *n*_eff_ is the effective index of refraction of the ambient material in the cavity. Due to this polarization dependence for the cavity dispersion with plasmonic cavities, both polarizations must be considered for such systems.^151−156^

For a plasmonic cavity, one should note that the dipole approximation (and the long wavelength approximation in eq 41) is not as valid since the electric field distribution is varying rapidly on the scale of the distribution of the electronic density. Further, the size of the plasmonic excitation is comparable to the size of the interacting molecular electron density, so the use of the dipole approximation for the molecular DOFs will no longer hold for these plasmonic interactions. It is also worth noting that molecules are thought to couple to the plasmonic environment via longitudinal fields (i.e., direct Coulomb interactions between the plasmon oscillations and the adjacent molecules). Nevertheless, the light–matter coupling in plasmonic cavities may still contain a similar form of the DSE as in the Pauli-Fierz Hamiltonian (eq 56), which has been pointed out in refs (157, 82). Finally, these plasmonic cavities also exhibit Landau-type damping as a primary source of dissipation.^157−159^ All of these aforementioned differences place plasmonic cavities in a separate category for discussion. For this section and most of this review, we will focus on Fabry–Pérot cavities. We refer the reader to other references for a more in-depth discussion on the modeling and simulation of plasmonic cavities.^50,153,157,159−170^

It is worth mentioning that while in this review we focus on the linear polarization of radiation, exotic effects may be achieved when coupling matter to circularly polarized radiation modes in chiral cavities which allows to break fundamental materials symmetries.^171^ This recent direction in polaritonic chemistry in chiral cavities may enable enantioselective photochemistry,^172^ enhancing the circular dichroism signal^173^ and inducing valley polariton depolarization^174^ according to recent theoretical works. This preferential treatment of molecules may give rise to additional tunability afforded by the cavity to control outcomes of reactions that exhibit one or more chiral centers. Although it is beyond the scope of this review, there have been many recent studies exploring this phenomenon from both theoretical and ab initio perspectives.^172,173,175,176^

With the motivation of this model in mind, in the next section, we first present a generalized dipole-gauge Hamiltonian and then a generalized Tavis-Cummings Hamiltonian.

#### 2.6.1 Many-Molecule Dipole-Gauge Hamiltonian

When considering cavities with many *k*_*x*_ modes, the energy eigenspectrum is typically visualized on a dispersion plot, where the eigenenergies are plotted as a function of *k*_*x*_. To find these *k*_*x*_-resolved energies and states, the Hamiltonian in question needs to be truncated to the set of modes with a given *k*_*x*_. This truncation is classified by the projection operator,
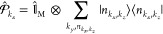
95where  is the identity for all matter degrees of freedom, and {|*n*(*k*_*x*_,*k_z_*)⟩} are the Fock states for a given *k*_*x*_ and *k*_*z*_. To avoid gauge ambiguities, this mode truncation can be performed as discussed in ref (75), where the  enters into the exponential of the PZW operator (see eq 73). Then, for each *k*_*x*_, this truncated Hamiltonian is diagonalized to find the dispersion plots and corresponding Hopfield^150^ coefficients as a function of *k*_*x*_.

To derive such a Hamiltonian, we start from the minimal coupling Hamiltonian (eq 45), following the framework discussed in ref (55). It is convenient to rewrite this Hamiltonian by grouping the matter particles into well-separated molecules, where the intermolecular distances are much longer than the intramolecular/interatomic distances. In such circumstances we can write *Â*(**x***_j_*) ≈ *Â*(**x̅***_J_*) for all *j* particles within the molecule *J* with center of mass of the molecule **x̅***_J_*, and the total Hamiltonian is written as
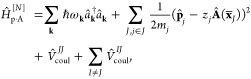
96where {*I*, *J*} are the indices over the molecules in the system whose centers of mass are located at **x̅***_I/J_*, {*j*} are the indices over each particle *j* in the molecule *J*, *V̂*_coul_^*JJ*^ is the intramolecular Coulomb potential in molecule *J*, and *V̂*_coul_^*JJ*^ is the intermolecular Coulomb potential between molecules *I* and *J*.

To transform this into the dipole gauge, we use the PZW operator (eq 48), but now with **Â** (**x***_J_*) not under the long wavelength approximation
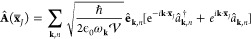
97where the general expression of the quantized electric field *E*_⊥_ and magnetic field *B̂* can be found in standard QED textbooks (for example refs (54, 55) or the Appendix of ref (12)).

The corresponding PZW gauge transform operator becomes a *multicentered* PZW operator^55,99^ expressed as
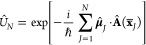
98which has specific centers of molecules **x̅***_J_*. This *Û*_*N*_ is still a momentum boost operator on **p̂***_j_* (of the *j*_th_ charged particle that belongs to the *J*_th_ molecule), given that we assume the individual molecules are neutral, much smaller than the wavelength of the mode, and can be well described by their dipoles.^55^ Under these approximations, *Û*_*N*_**p̂**_*j*_*Û*_*N*_^†^ = **p̂***_j_* + *q*_*j*_**Â** (**x̅***_J_*). We can also evaluate *Û*_*N*_*â*_**k**_*Û*_*N*_^†^ as,^55^
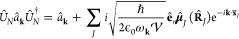
99where **μ̂***_J_*(**R̂***_J_*) is the dipole operator of molecule *J* with the nuclear configuration **R̂***_J_*. Additionally, the phase rotation from eq 54 can be generalized for many modes as
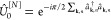
100where all the modes now experience a phase rotation.

Now, we can write our many molecules and many modes Pauli-Fierz Hamiltonian in the full Hilbert space as,
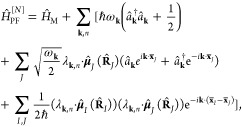
101where we introduced a coupling parameter for this more complicated system, . While this is rigorous, its computational cost can quickly become enormous. The following simple basis size analysis can demonstrate this. For *j* molecules with *l* electronic states and *m* modes with *n* Fock states, the basis size scales as *l*^*j*^*n*^*m*^. Due to this unfavorable scaling, the generalized Tavis-Cummings Hamiltonian is a useful approximation to simulate these systems.

#### 2.6.2 Generalized Tavis-Cummings Hamiltonian

Intuitively, the generalized Tavis-Cummings (GTC) Hamiltonian is to the generalized dipole gauge Hamiltonian as the Jaynes-Cummings Hamiltonian is to the traditional dipole gauge Hamiltonian. In this manner, there are a series of approximations from eq 101 to get the GTC Hamiltonian. Namely, we first truncate each molecule to the two-level approximation and remove the permanent dipole, such that the dipole operator for a given molecule can be written as **μ̂***_J_* = **μ**_*J*_^eg^ σ̂_*x*_, where **μ**_*J*_^eg^ is the transition dipole moment between the ground and excited state for molecule *J*. Then, the dipole self-energy terms (last line of eq 101) are neglected entirely. Finally, the rotating wave approximation is performed such that the interaction terms go as
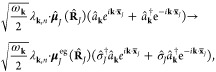
where σ̂_*J*_ is the lowering operator for molecule *J*’s two-level system. This series then leads to an expression of the GTC Hamiltonian
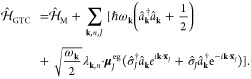
102

Further making a single cavity mode approximation (only keeping one **k** with *k*_*x*_ = 0, where ê is along *x*), with frequency ω_c_) and the long wavelength approximation, this Hamiltonian reduces to the Tavis-Cummings Hamiltonian discussed in Section 1.2. Note that the GTC Hamiltonian in eq 102 has been used in recent ab initio polariton quantum dynamics simulations, such as those in refs (138) and (177).

The benefit of having this generalized Tavis-Cummings model is that now it is easier to run simulations in the single excited subspace since different excitation levels are now decoupled from each other. This drastically reduces the computational cost of modeling large systems. In particular, since even in simulations *N* is typically fairly large, most numerical calculations using this model consider only the first excitation subspace. This drastically reduces the basis size from 2^*N*^ × *N*_F_ for *N*_F_ Fock states to (*N* + 1). Recently, studies involving this GTC Hamiltonian have been able to shine new light on the dispersion plots seen in experiments^40,128^ (see Figure 4(b–d)).

One such observed phenomenon that can be predicted by the GTC is the presence of collective ”bright” and “dark” states formed by the hybridization of each molecule with each *k*_*x*_ mode. It should be noted that these terms refer to the presence (or lack thereof) of photonic character in the energy eigenstates of this system. By hybridizing *N* singly excited molecular states with 0 photons with a collective molecular ground state with a single photon, *N* + 1 energy eigenstates are formed. The upper and lower polaritons make up the two bright states, and the other *N* – 1 states become dark states with no photonic character, making them energetically degenerate (when ignoring disorder).

It should be noted that the typical Tavis-Cummings Hamiltonian, as discussed in the Introduction, is found by making the long wavelength approximation on the GTC Hamiltonian shown in eq 102. This simply removes the phase terms, exp{ ± *i***k** · **x̅**_*J*_}, essentially stating that the molecules are identical and that the field is spatially invariant across all the molecules.

The Tavis-Cummings Hamiltonian in general can be used with various matter Hamiltonians. One specific model that is commonly used is the Holstein-Tavis-Cummings (HTC) model.^8,178^ In this model, the matter Hamiltonian consists of an array of two-level systems with phenomenological phonon modes added to the system. This HTC Hamiltonian can then be extended from eq 12 as,
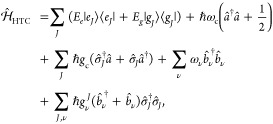
103where *b*_ν_^†^ and *b̂*_*ν*_ are the creation and annihilation operators for the ν_th_ phonon mode, respectively, with frequency *ω*_*ν*_, phonon coupling strength *g*_ν_^*J*^ and molecular excitation operator σ_*J*_^†^ = |*e*_*J*_⟩⟨*g*_*J*_|. Both the GTC Hamiltonian and the HTC Hamiltonian have been extensively used in recent theoretical simulations in molecular polariton systems.^40,126,138,139,152,155,177,179^ The details will be discussed in Section 6.1.

## 3 Ab Initio Methods for Molecular Polaritons

Coupling polaritonic Hamiltonians such as eq 55 with realistic, ab initio calculations for molecular systems has generated much recent work. Most of the molecular ab initio polariton chemistry works are based on the single-mode light–matter interaction Hamiltonian in eq 57, which is equivalent to eq 56 as explained in Section 2.3.2. Here, for consistency, we choose to use the PF Hamiltonian in eq 56 to describe the ab initio polaritons. In particular, we express eq 56 as follows
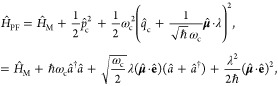
104where, to be consistent with the ab initio polariton literature, we use the light–matter coupling strength defined as^53,72,82,83,115,118−125,180^
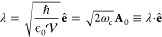
105where **ê** is the electric field unit polarization vector,  is the cavity volume, and ϵ_0_ is the permittivity of free space. Note that in Section 2, we have used the magnitude of the vector potential, *A*_0_, itself as the coupling strength. On the other hand, when coupling solid state materials, the total dipole operator is no longer well-defined, and *Ĥ*_pA_ (eq 45) is often used. For example, in ref (98) and ref (174), the polariton states of a 2D TMD coupled to an optical cavity are computed based on *Ĥ*_pA_, where one needs to evaluate the matrix elements of the matter momentum operator.

The central task of the ab initio molecular polariton chemistry is then to solve the polariton states and obtain polariton potential energy surfaces, which are the eigenstates and eigenenergies of the following polariton Hamiltonian
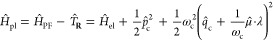
106where *Ĥ*_PF_ is expressed in eq 104, and *Ĥ*_el_ is the electronic Hamiltonian defined in eq 20. In term of the raising and lowering operator of the field, the polariton Hamiltonian in eq 106 becomes
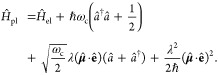
107

The eigenvalue equation of *Ĥ*_pl_ is expressed as
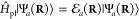
108where |Ψ_*a*_(**R**)⟩ is referred to as the *a*_th_ polariton state and  (**R**) is the *a*_th_ polariton surface or cavity Born–Oppenheimer surface.^181,182^ Note that both |Ψ_*a*_(**R**)⟩ and _*a*_(**R**) parametrically depend on **R**, analogous to eq 21 for the adiabatic states and energies of the bare molecule.

By far, there are two popular approaches in literature to solving this coupled electron-photon-nuclear system described in eq 108. Approach (I): solving the electron–nuclear problem followed by diagonalizing eq 55 with these electronically adiabatic basis states along with a photonic basis (e.g., number/Fock states, generalized coherent states,^183^ polarized Fock States,^51^ etc.). Approach (II): incorporating the photonic DOFs (through eq 55) into the common electronic structure framework whereby self-consistently solving the electron-photon-nuclear problem in one step. Both methods afford adiabatic polaritonic states as a result. This is because the two methods only only differ in the resulting basis describing the polaritonic system.

In Approach (I), the basis of electronic adiabatic states and, e*.*g., number states, never changes, and upon diagonalization of eq 55 gives some description of the polaritonic states, which may require extensive basis sets for the electronic DOFs.^115^ This scheme will be referred to as the frozen adiabatic basis approach or parametrized QED (pQED).

In (II), the initially adiabatic electronic and photon basis sets are self-consistently updated to minimize the number of basis states needed to properly describe the polaritonic system, which, in general, should give a more accurate and trustworthy description of the ground and excited states due to their self-consistent nature. This scheme will be referred to as the self-consistent QED scheme (scQED). In this scheme, the electronic DOFs will be perturbed by the presence of the photonic terms in the Hamiltonian, which has led to studies involving how the ground state orbitals will react to these additional photonic terms, which will be discussed in more detail later..^124,184,185^

### 3.1 Parameterized QED Approach

#### 3.1.1 Adiabatic-Fock Electron-Photon Basis

We now discuss a simple approach to solve the QED problem where one treats the electronic and photonic basis states as frozen (i.e., not self-consistently updated). This is often referred to as the “adiabatic”, frozen basis, or parametrized QED approach, and is commonly used in the atomic cavity QED problems.^88,90^ We will exclusively refer to this procedure as the parametrized QED (pQED) approach in this review. In this approach, one first solves eq 21 using *any* electronic structure method of choice, obtaining the adiabatic electronic states, |*ψ*_*α*_(**R**)⟩. One can then construct the tensor product of adiabatic electronic states, |*ψ*_*α*_(**R**)⟩, and Fock states, |*n*⟩, as the basis, |*ψ*_*α*_(**R**),*n*⟩ ≡|*ψ*_*α*_(**R**)⟩⊗|*n*⟩, where the character of this basis explicitly depends on the nuclear position, **R**. This basis is commonly referred to as the adiabatic-Fock basis.

Because we are going to work with a finite set of electronic states, that means one should use eq 70 for , and the polariton Hamiltonian in the finite electronic space is
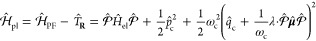
109where the matter state truncation needs to be performed as  and not (See Sec. 2.4.2 for a detailed discussion). For the polariton Hamiltonian in eq 106 one can use the adiabatic-Fock basis {|*ψ*_*α*_(**R**),*n*⟩} to evaluate the matrix elements  resulting in^118,180^
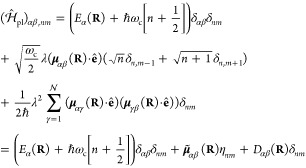
110where {α, β, γ} label the electronic adiabatic states (where there is a total of  electronic adiabatic states being considered), {*n*, *m*} label the photonic Fock states, **ê** is the polarization unit vector of the electric field, ,  =  + , and  = . Here, only the electronically adiabatic state energies *E*_*α*_ and transition dipole matrix elements *μ*_*αβ*_ are required as input. As has been known for many decades, solving the many-body electronic system is not trivial, while the harmonic oscillator problem is an easy text book problem. The purpose of this pQED procedure is to make use of the simplicity of the photonic subsystem, while still relying on complicated many-body methods to extract the necessary information from the electronic subsystem as input. An important distinction to make is that the basis states {α, β, γ} are many-particle states (Slater determinants or their combinations) instead of single-particle states (spin orbitals). The square electronic dipole operator μ̂^2^ using the many-body wave functions can be directly evaluated by inserting a complete set of many-body wave functions (as was done in eq 110) since the dipole matrix elements between the many-particle states are known directly. However, in the basis of single-particle states (e.g., Kohn–Sham orbitals), one needs to consider terms arising from one and two-particle dipoles, as is shown in many works using the scQED method (to be discussed later in Section 3.2.^122,124,175,184^

Upon diagonalizing the matrix of (eq 110), one obtains the expansion coefficients for the polaritonic states {|Ψ_*i*_ (**R**)⟩} in the basis of the adiabatic electronic and Fock states as,
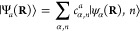
111where the coefficients *c*_*α,n*_^*a*^ (**R**) = ⟨*ϕ*_*α*_(**R**),*n*|Ψ_*a*_(**R**)⟩ can be used to compute any observables of the resulting polaritonic system (which will be revisited later). Note that the expansion coefficients also explicitly depend on the nuclear configuration, due to the **R**-dependent adiabatic states |*ϕ*_*α*_(**R**)⟩. This is also the common procedure in quantum optics to solve polariton eigenstates of model systems couple to cavity, for example, the results presented in Figure 2.

More practically, the construction of the Hamiltonian matrix can be easily achieved through tensor products, but it is worth examining the block structure of the matrix to understand how the dipole matrix plays such an important role in resolving the low-lying polaritonic states. The Hamiltonian matrix (eq 110) can be written as
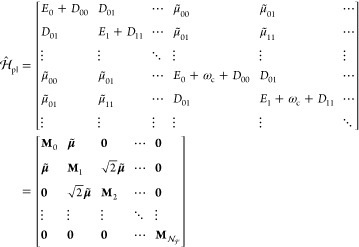
112where  is extremely sparse with a triblock-diagonal structure connecting the block  =  +  + , to the block of electronic states dressed with *n* ± 1 photons **M**_*n*±1_ is now evident. The **M**_*n*_ and **M**_*n*+1_ blocks are coupled through . Additionally, the electronic states with the same photon number (i.e., *n* = *m*) are connected only via the DSE terms *D*_*αβ*_. Note here that  is the maximum number of included Fock basis states for the photonic subsystem. Recall for blocks with larger numbers of Fock states, one picks up the additional  term on each of the μ̃ blocks, *which effectively increases the effects of the coupling terms***μ̃***with increasing numbers of photons*.

Noting again the block structure in eq 112, the computational efficiency of this exact diagonalization can be drastically increased by the use of sparse matrix methods (e.g., Lanczos),^186−188^ which can be heavily relied on for approximate diagonalization of the lowest eigenvalues and eigenvectors without loss of physics but with a large computational speed-up. For this approach to be successful, one is required to treat the number of electronic (i.e., size of **M** and **μ̃**) and photonic basis states (i.e., ) as convergence parameters which provides a rigorous approach to solving the QED Hamiltonian, and it is exact for an infinite basis set. However, in the literature, often the electronic system is truncated to only include the ground and first excited molecular states and only the vacuum |0⟩ and |1⟩ photonic states. As we will see in the following section, this will lead to a breakdown of the physics, especially at larger coupling strengths specifically due to the DSE terms connecting blocks of the Hamiltonian far-away in energy.

**Figure 5:**
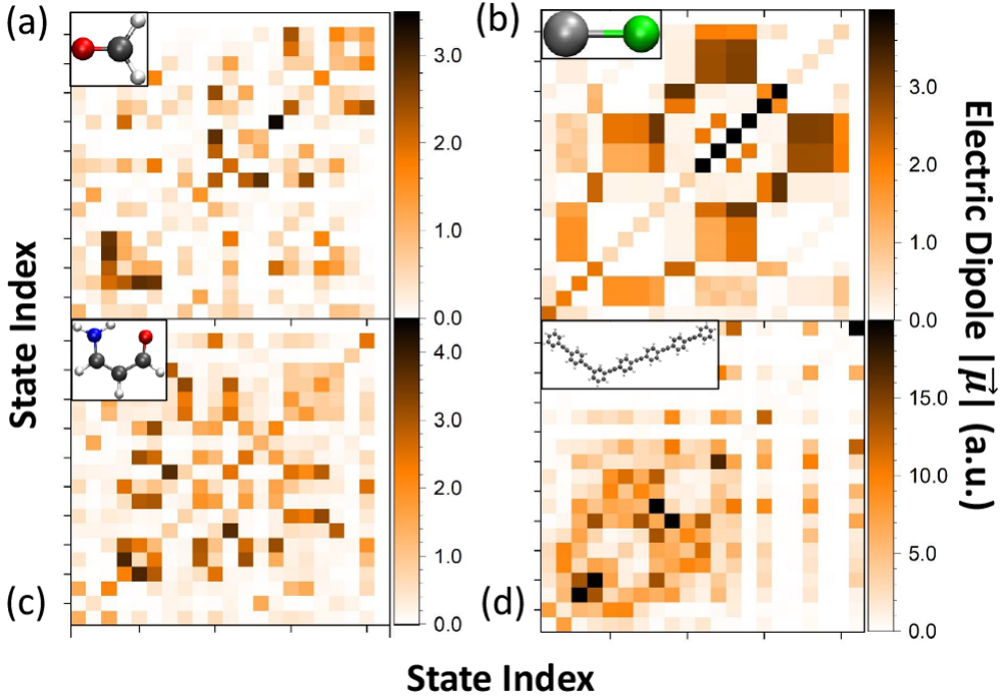
Norm of the transition dipole vector matrix elements |*μ⃗*| calculated with TD-DFT/B3LYP for the lowest 20 electronic states of four molecules. (a) Formaldehyde. (b) Aminopropenal. (c) LiF. (d) 35PPE. These matrices appear directly in the μ̃ block terms in eq 112, and their matrix squares form the DSE terms which are located inside the ***M*_n_** blocks.

Furthermore, the truncation of the electronic dipole matrix with the number of included adiabatic electronic states will drastically affect the results, since the transition dipole matrix appears directly in the light–matter coupling term and its square appears in the DSE term, thus possibly contributing a great deal of complication to the off-diagonal (and on-diagonal) couplings due to the shape and distribution of the transition dipole matrix itself. As an example, Figure 5 showcases the dipole matrix for four molecules: (a) formaldehyde, (b) LiF, (c) animopropenal, and (d) 35PPE, all of which under recent study in polaritonic schemes.^51,121,125^ In each case, the 20 lowest energy adiabatic electronic states are shown as the vector norm of the dipole matrix elements as computed at the TD-DFT level. In all four cases, although symmetry-based arguments regarding selection rules could be applied, it is hard to discern any pattern of the dipole matrix elements. The two small organic molecules (Figure 5a,c) showcase the most scattered of the dipole matrices, the LiF (Figure 5b) shows a block-like structure (due to the reduced dimensionality), and the large organic species (Figure 5d) shows an intermediate regime where the high-energy states are weakly coupled and have some structure while the low-energy states showcase a strong degree of coupling in a block-like fashion. In this molecule, there is evidence of charge-transfer states (e.g., states 2 and 3) with large permanent dipoles due to the large spatial reorganization of charge upon electronic excitation.^189^

Using the pQED procedure, one needs to pay careful attention to the electronic dipole matrix and discern the distribution of strong coupling. At larger numbers of electronic states (i.e., ∼100 states), it is usually straightforward to see where the strong coupling away from the diagonal elements will decay to near zero. This effective “width” is expected to play a direct role in the convergence of the electronic basis states used for the pQED procedure. However, one also requires the square of this dipole matrix (which will change depending on the choice of electronic state truncation ) for obtaining the DSE terms, which adds additional complexity to the situation. To be clear, the convergence of the polaritonic states requires a balance between the matter and photonic expansions^53^ which is, in general, not trivial to know a priori nor necessarily guaranteed to converge at all when the light–matter coupling is large for an arbitrary large molecular system with many electronic states.^115^

#### 3.1.2 Polarized Fock State Basis

We have outlined the pQED scheme using the adiabatic electronic state and photonic Fock states as the basis. Another popular representation for the photonic degrees of freedom includes the grid basis, which is the eigenbasis of *q̂*_c_ and has been extensively used.^5,107,181,190^ The choice of basis can significantly enhance computational efficiency or reduce the conceptual complexity of a problem.

One such basis that provides computational as well as conceptual convenience is the recently proposed polarized Fock State (PFS) basis introduced in ref (51). Here, the Pauli-Fierz Hamiltonian (eq 109) is rewritten using an entangled electronic-photonic basis, where matter is represented in the eigenstates of the dipole operator  and is referred to as the Mulliken-Hush (MH) representation (see details around eq 29). The light–matter Hamiltonian (see eq 109) using the MH basis can be written as,^51,191^
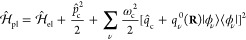
113where . Notice that the photon field is now described by the MH-state specific displaced harmonic oscillators centered around – *q*_ν_^0^ (**R**). This displacement can be viewed as a *polarization* of the photon field due to the presence of the molecule-cavity coupling, such that the photon field corresponds to a nonzero (hence polarized) electric field, in contrast to the vacuum photon field. Within this representation the Fock states have been explicitly shifted by a quantity proportional to the molecular dipoles and light–matter coupling λ, whose shift is evident from direct examination of the last term in eq 109.

This Hamiltonian can be now block-diagonalized using the polarized Fock basis (PFS) {|*n*_*ν*_(**R**)⟩} for each |*ϕ*_*ν*_⟩, which is defined as,
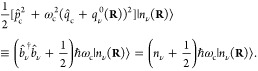
114

The electron-photon subsystem can be represented with the following tensor product of MH and PFS basis
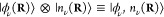
115which is a light–matter *entangled* basis because one needs to specify both the nuclear position **R** and the MH diabatic electronic state |*ϕ*_*ν*_⟩ to define the polarized Fock states |*n*_*ν*_(**R**)⟩. Using this basis,  is expressed as
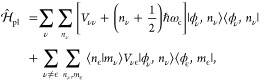
116where we have dropped the **R** dependency for simplicity. Note that there is a finite coupling between the MH state *ϕ*_*ν*_ with *n*_*ν*_ photons and the MH state *ϕ*_*ϵ*_ with *m*_*ϵ*_ photons through the ⟨*m*_*ϵ*_|*n*_*ν*_⟩ *V*_*νϵ*_ term, which is the off-diagonal matrix element of the electronic Hamiltonian, *V*_*νϵ*_, scaled by the overlap, ⟨*m*_*ϵ*_|*n*_*ν*_⟩, of the PFS. This overlap is nonzero and is simply the overlap of two harmonic oscillator wave functions that are shifted from one another by . Thus, instead of having an explicit light–matter interaction term (and the DSE) as shown in eq 111, these interactions are now completely carried through ⟨*m*_*ϵ*_|*n*_*ν*_⟩· *V*_*νϵ*_(**R**). This basis is expected (and has been explicitly shown for models systems^51^) to efficiently converge the photonic basis, especially when the permanent dipoles *μ*_*νν*_(**R**) in the MH basis are large. For additional discussion, see ref (51).

A similar formulation was also presented in ref (53), where displaced Fock states |*n*(**R̂**,****r̂****)⟩ were introduced which parametrically depend on the nuclear position operator **R̂** as well as the electronic position operator **r̂**. Here, the displacement of the Fock state is proportional to the total dipole *∑*_*j*_*R*_*j*_ – *∑*_*j*_*r*_*j*_. Using this framework, ref (53) also introduced a generalized Born–Oppenheimer approximation to include photons, such that the nuclear part of the many-body electronic-nuclear-photonic wave function ansatz is factorized from the electronic-photonic part. The basis based on this work will be discussed in Section 3.2 and is called the generalized coherent state (GCS) basis.^183^

#### 3.1.3 An Example: LiF Coupled to Cavity with the pQED Approach

Here, we give an interesting example that has been extensively explored, which is a LiF molecule coupled to a single mode cavity. We will only focus on the polariton potential energy surfaces and not consider the time-dependent polariton dynamics (which will be discussed in Section 4.1). In addition, we will only focus on two electronic states of the LiF molecule . We emphasize that one should treat the number of electronic states  as a convergence parameter in pQED calculations.^115^

**Figure 6:**
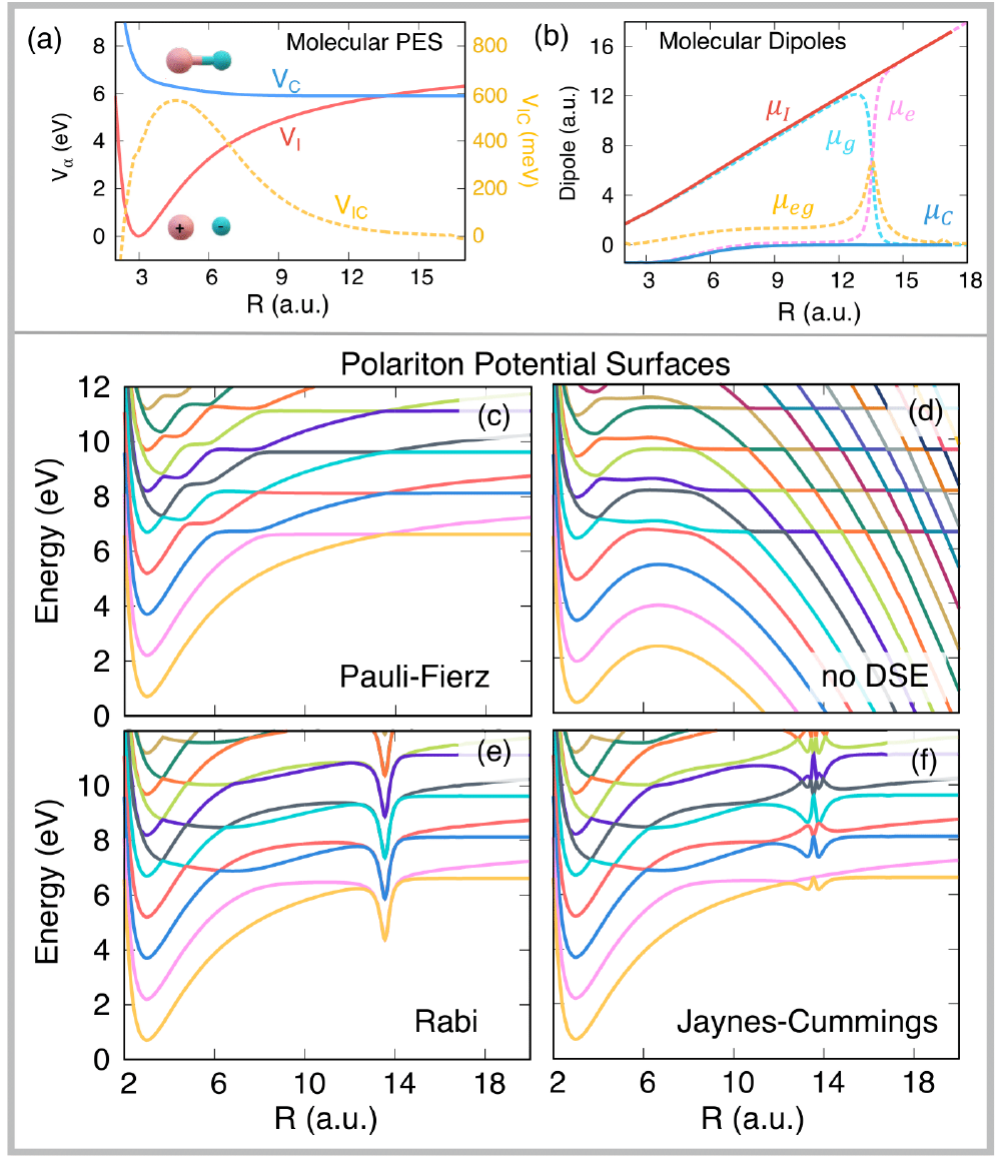
Molecular Polaritons in LiF Dissociation: A comparison between and other quantum optics models. Polariton Eigenstates of a LiF molecule (two level model system with details in ref (51)) coupled to a single mode optical cavity, using the (a) rigorous PF Hamiltonian as well as various quantum optics models, including (b) PF Hamiltonian without DSE, (c) Quantum Rabi model (eq 82) and (d) JC model (eq 83).

Figure 6 presents the polariton potential energy surfaces predicted by various quantum optics model Hamiltonians for the model LiF molecule shown in Figure 6a,b (the details of the model can be found in ref (51)). Here, only two diabatic states are considered, which are denoted as the ionic state |I⟩, and covalent state |C⟩. These two diabatic states are coupled through a diabatic coupling *V*_IC_(*R*) (dotted yellow line in Figure 6a) that causes a splitting (avoided crossing) near the anticrossing of the diabatic potentials *V*_C_(*R*) and *V*_I_(*R*) (solid red and blue line in Figure 6a, respectively). The adiabatic electronic states, ground |*g*(*R*)⟩ and excited |*e*(*R*)⟩ states can be obtained by diagonalizing the electronic Hamiltonian ***Ĥ***_el_ = *V*_I_ (*R*)|I⟩⟨I| + *V*_C_ (*R*)|C⟩⟨C|+*V*_IC_ (*R*)(|I⟩⟨C|+|C⟩⟨I|) at each *R*.

The dipole moment matrix at each *R* is diagonal in this diabatic representation. This is because the diabatic states |I⟩ and |C⟩, so-called Mulliken-Hush diabatic states, are the eigenstates of the electronic transition dipole operator by definition (see discussion around eq 29). Figure 6b presents the matrix elements of **μ̃** in both the diabatic (solid lines) and the adiabatic (dashed lines) representations. As expected, the permanent dipole for the ionic state |I⟩ (corresponding to Li^+^F^–^) μ_I_(*R*) linearly increases, while the permanent dipole for the |C⟩ state (corresponding to covalently bonded Li–F) μ_C_(*R*) remains nearly zero with increase in interatomic separation *R*. The adiabatic states switch their characters around *R* ≈ 13.5 au, as a result, the adiabatic permanent dipole switches in that region, and *μ*_*eg*_(*R*) peaks at *R* ≈ 13.5 au

The relative importance of different terms in the PF Hamiltonian and the consequences of ignoring them is illustrated in Figure 6c–f. For example, the dipole self-energy (DSE) plays a crucial role in molecular polaritons to guarantee a bounded ground state and excited states,^12,48,82,88,125,192,193^ even though DSE is a constant in atomic polaritons and are dropped out in most of the atomic cavity QED models (see Section 2.5). Without the DSE, there will be an unphysical bending of the polariton potential, which is clearly demonstrated in Figure 6d. Without DSE, the gauge invariance between the minimal coupling Hamiltonian and the electric-dipole Hamiltonian will break down.^48,53,88,90,104,192^ Further, without the DSE, the ground state is no-longer bounded and becomes dissociative (and unbounded) at a large nuclear distance.^48^ The Rabi model, which explicitly ignores the presence of the permanent dipole, explicitly breaks down when electronic states have a large permanent dipole difference.^51^ Neglecting the permanent dipole, as commonly done for most of the current molecular cavity QED studies,^6,127^ can cause unphysical dips in the polariton potentials,^127^ as demonstrated in Figure 6c. The JC model which assumes RWA, explicitly breaks down in the recently emerged ultrastrong coupling regime,^47,80^ and also gives unphysical dips of the potential (Figure 6). Thus, one need to use the most rigorous Hamiltonian to describe the light–matter interactions and try to avoid unnecessary approximations.

### 3.2 Self-Consistent QED Approaches

We will briefly overview the recent work to integrate the PF QED Hamiltonian (eq 55) into a variety of electronic structure methods to provide a self-consistent solution to the ground and excited polaritonic states.^194^ Note that in the previous section for the pQED approach we chose a basis for polaritons that cannot change in a variational or self-consistent sense, while for the self-consistent methods (scQED, Approach (II)), the basis is iteratively updated to minimize the energy of the entire Hamiltonian. In this sense, this procedure may require a smaller number of overall electronic/photonic states than the pQED procedure; however, the scQED method requires knowledge of the low-level basis of the electronic system (e.g., atomic orbitals, plane waves, etc.) while the pQED method only requires the resulting many-body state energies and transition dipoles (i.e., as solved by CIS, TD-DFT, EOM-CC, etc.). In this way, the technical details of the self-consistent schemes become more complicated. In contrast to this, in the pQED procedure, the convergence of the basis becomes a more important consideration due to the lack of response of the basis to the presence of the photon field. That is to say, the scQED schemes also rely on a truncation in both the electronic and photonic subspaces. However, the character of higher-lying excitations (specifically in the electronic subsystem) can still be mixed through the inclusion of more virtual single-particle orbitals (i.e., a convergence parameter) while the higher excitations in the photonic DOFs can be captured through the polarized Fock state^51^ or coherent state^122,124,183,184,195^ representations.

We want to make a special comment regarding the choice of notation for the self-consistent methods. In this work, we use scQED to refer to these methods; however, this is not to be confused with another common notation of similar, but different, meaning, SC-QED, which is the strong coupling QED.^124,176^ As we will see in this section, in the strong coupling regime of light–matter interactions, a fully self-consistent method is often necessary to converge the polaritonic properties, so in this way, the scQED and SC-QED notations are intimately related.

As is usually done, we first approach this problem by way of mean-field Hartree–Fock (HF) theory and continue toward higher-level schemes such as time-dependent density functional theory and coupled cluster methods, with the final section of this chapter covering recent applications of the aforementioned scQED and pQED schemes. Thus, far, we are not aware of any theoretical work on scQED work that explicitly solves many molecules coupled to many cavity modes beyond long wavelength approximation, such as described by the Hamiltonian in eq 101. Since this is highly relevant to the description of the actual molecule-cavity coupling in most of the experimental setup, future theoretical works should focus toward this direction to achieve a more direct comparison with experiments. Nevertheless, the ongoing ab initio scQED approaches layout the groundwork toward that goal and will be the topic of the following discussion.

#### 3.2.1 QED Hartree–Fock

Canonical HF theory attempts to describe a many-body system’s ground state by the use of a single Slater determinant, |Φ^HF^⟩, that yields an uncorrelated ground state. This is usually the basis for so-called post-HF methods that will be discussed later, such as the configuration interaction (CI) and coupled cluster (CC) methods. For the polaritonic system, one extends this ideology to include the photonic DOFs such that the uncorrelated electrons and photons use the following direct product state. However, to simplify the problem, many authors have opted to use the coherent state basis^183^ for their implementations of the scQED schemes^122,195^ for the photonic DOFs, which alleviates some of the complexity in notation as well as provides a useful interpretation of the effects of the cavity on the ground state properties.

In order to illustrate the convenience of the coherent states, following closely the notation of ref (122), we first construct the HF ground state Ansatz for the hybrid system via a tensor product of the bare molecular HF ground state (which is a Slater determinant of molecular orbitals who are themselves linear combinations of atomic orbital basis) and a Fock state of the cavity mode as follows
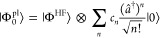
117where |0⟩ is the photon vacuum state of *H̃*_ph_ (eq 42), *a*^†^ is the photon creation operator (see eq 42 and eq 43) and *c*_*n*_ is the expansion coefficients for the photon number states. The HF energy for the molecule-cavity hybrid system is then computed in the usual variational way by using the |Φ_0_^el+ph^⟩ to sandwich *H̃*_pl_ (eq 106) as follows
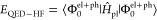
118where the usual HF mean-field procedure is used to iteratively modify the electronic HF molecular orbitals and photon coefficients, eventually reaching a self-consistent solution to the ground state energy of the molecule-cavity hybrid system. In practice, one can first obtain the bare molecular HF energy, *E*_HF_, outside the influence of the cavity and variationally optimize the photonic expansion coefficients of the partially evaluated PF Hamiltonian as follows^122^
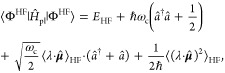
119where ⟨···⟩_HF_ = ⟨Φ^HF^|···|Φ^HF^⟩ is the HF ground state expectation value of the molecular subsystem, and we have not used any photonic basis to evaluate the expectation value for *â*^†^ and *â*. Note that in eq 119, the light–matter interaction is carried by the term ⟨**λ μ̂**⟩_HF_ ·(**â**^†^ + *â*) resulting from the *Ĥ*_PF_ in eq 55.

This partially diagonalized expectation value in eq 119 can be fully diagonalized in the coherent state basis^183^ defined by the unitary transformation^115,122,124,195^
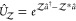
120which will shift the photonic creation and annihilation operators for an arbitrary complex , such that  and . This is in the same spirit of the polarized Fock state idea in the previous section (Section 3.1), which is a polaron-like transform on the photonic DOF. Choosing the particular  as follows^115,122,124,195^
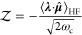
121one can transform the Hamiltonian *Ĥ*_pl_ by unitary rotation , resulting in  as follows
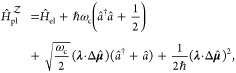
122where Δ*μ̂* = *μ̂* – ⟨*μ̂*⟩_HF_. Note that because  is a unitary operator, it will not change the eigenvalue of the problem.

With the transformed Hamiltonian in eq 122, one can still evaluate its HF variational expectation value as
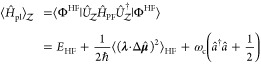
123and the light–matter coupling term explicitly vanishes due to the fact that ⟨Δ*μ̂*⟩_HF_ = ⟨*μ̂* – ⟨*μ̂*⟩_HF_⟩_HF_ = 0. Using this strategy, the light–matter coupling term ⟨λ · **μ̂**⟩_HF_ · (*â*^†^ + *â*) resulting from the *Ĥ*_PF_ (in eq 119) no longer explicitly shows up in eq 123, and the implicit coupling between molecule and cavity is now carried through
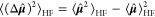
124which can be intuitively understood as the dipole fluctuations due to coupling to the cavity.

The variational expectation value in eq 123 suggests that the eigenstates of this Hamiltonian are simply the Fock states. However, one should not be confused by its appearance as HF needs to be solved through many iterations (in a self-consistent manner), and for each iteration, the shift  needs to be re-evaluated, and a new unitary transformation needs to be constructed, similar to how the HF density matrix needs to be reconstructed to progress the self-consistent cycle.

In the original Hamiltonian (eq 119), the eigenvectors become the generalized coherent states themselves,
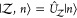
125where  is the cavity Fock state.

The HF equations can be solved through iterative diagonalization, and at each iteration the HF electronic molecular orbitals are updated and are used to evaluate the shift  expressed in eq 121. The Fock matrix can be written explicitly as
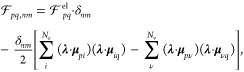
126where {*p*, *q*} indicate all possible molecular orbitals, and {*i*} and {ν} indicate strictly occupied, and strictly virtual HF electronic orbitals, and *F*_*pq*_^*el*^ is the bare molecular Fock matrix in the HF orbital basis. {*n*, *m*} are the photonic Fock/number basis states. The Fock matrix here, by construction, is similar to eq 123 and does not contain the electron-photon interaction term, which necessarily drops out in this picture since that term mixes states with varied numbers of photon basis states while the DSE term connects only electronic states. Noting that the solution to the bare molecular Fock matrix is achieved if , the QED-HF energy can be written as^122^
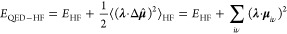
127which is then variationally minimized. More details on the scQED-HF scheme in varying complexity can be found in refs (122, 124, and 184).

We note here that, compared to the analogous pQED scheme which requires knowledge of the many-particle excited states of the bare molecular system, the scQED scheme offers a substantially cheaper calculation since no explicit excited states are required. In this case, the scQED scheme only requires convergence with respect to the number of virtual single-particle orbitals included in the variational scheme.

Furthermore, an interesting result of coupling cavity photons to molecular systems is the breakdown of Koopman’s theorem, used to approximate the ionization energies of molecules, due to the intrinsic spatial and orientational dependence on the molecular orbitals of the electronic system. Ref (176) has extensively discussed such consequences on the reinterpretation of Koopman’s theorem using the recently developed strong coupling QED-HF methods.^122−124,176,196,197^

#### 3.2.2 QED Coupled Cluster Theory

An improvement over the mean-field and single-reference methods can be systematically achieved by increasing the number of reference states (configuration Slater determinants). In electronic structure theory, one can achieve these by building a correlated wave function theory starting from HF, such as a configuration interaction (CI) approach that includes all possible singly (S) excited Slater determinants (CIS),^184^ or one that includes doubly (D) excited Slater determinants (CISD), or CISD(T), etc. However, the most computationally feasible and accurate methods stem from the coupled cluster (CC) approach. In particular, CCSD, which includes up to two electronic excitation operators in principle but indirectly includes correlation from higher-level excitations due to the location of the excitation operators in an exponential function. As such, this method has been shown to systematically achieve more accurate results compared to the analogous method in CI (e.g., CISD method) and sometimes even outperforms the CISDT methods.^198−202^ In this case, it is the most appropriate choice to extend to the QED formalism to correctly capture the correct electronic and electron–photon correlations that will result from coupling to the cavity. Even though this method is too expensive for most medium sized molecules, it provides a useful benchmark for other lower-order methods (e.g., scQED-TD-DFT).

Following closely with refs (122 and 203), the CC ansatz for the ground state polaritonic wave function is
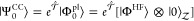
128where |Φ_0_^pl^⟩ is the polaritonic ground state calculated at the uncorrelated HF level (see previous section) and |Φ^HF^⟩ is the uncorrelated HF electronic ground state. Here,  is the photon vacuum state in the rotated coherent state representation with (see eq 120) at the variationally optimized coherent state parameter  after the HF self-consistent procedure.  is the cluster operator (not to be confused with the kinetic energy operator *T̂*_R_ or *T̂*_r_ in eq 20). This cluster operator involves a sum of electronic, photonic, and mixed electron-photon excitations as follows
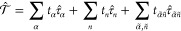
129where *τ̂*_*α*_ represents creation and annihilation operators for an α^th^-order electronic excitation. For example, τ̂_*i*_^ν^ = *ĉ*_ν_^†^*ĉ*_*i*_ excites an electron from an occupied orbital *i* to an unoccupied orbital ν. Similarly, τ̂_*ij*_^νυ^ = *ĉ*_ν_^†^*ĉ*_υ_^†^*ĉ*_*i*_*ĉ̂*_*j*_ will excite two electrons *i* → ν and *j* → υ, respectively. The photonic excitation operator can be written in a simple idempotent form^203^ as *τ̂n* = |*n*⟩⟨0| for a finite number of Fock states^203^. The coupled excitation operator *τĮ*_*α̃ñ*__;_ can be written, for example, as *ĉ*_ν_^†^*ĉ*_*i*_ |*n*⟩⟨0| for a single electron excitation coupled to an *n*_th_-level photonic excitation while *ĉ*_ν_^†^*ĉ*_υ_^†^*ĉ*_*a*_*cĮ*_*b*_ |*n*⟩⟨0| will provide the double electron and *n*_th_-level photonic excitations. Each of these excitation operators and one for every choice of *n* up to the photon level truncation  with a unique cluster amplitude *t*. It is important to note that refs (203) and (122) use different definitions of the cluster operator. Notably the photonic excitation operators in ref (122) (as well as in ref (195)) are instead the true photonic ladder operator *â* rather than the idempotent form used in ref (203).

**Figure 7:**
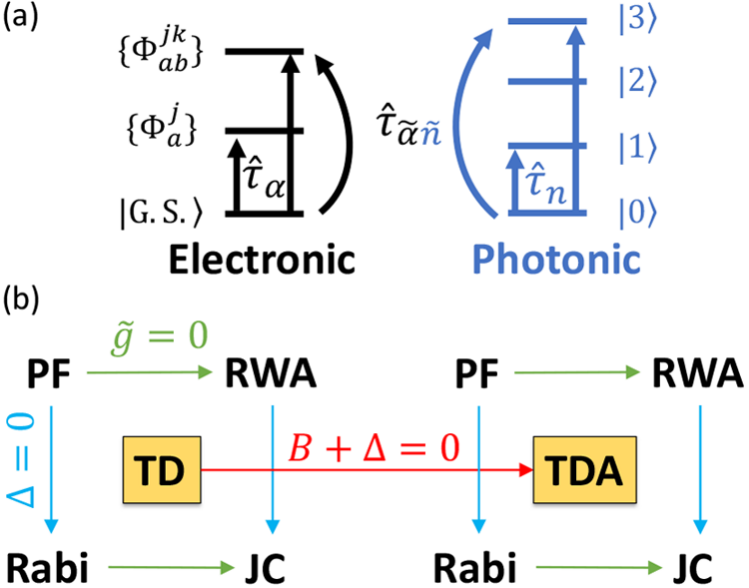
Implementations of QED-Electronic Structure Methods (a) The coupled cluster (CC) method can be understood via linear combinations of the excitation operators generated by the exponential of the total cluster operator (eq 129). Here, the CC excitations are visualized for the (left, black) electronic and (right, red) photonic subsystems, where each subsystem has an excitation operator (*τ̂*_*α*_, *τ̂*_*n*_) as well as a shared coupled-excitation operator *τ̂*_*α̃ñ*_. Including the maximum number of excitations (which trivially on the number of electrons in the system), one recovers the full configuration interaction (FCI) limit. (b) Various approximations to the QED-TD-DFT method (eq 142), generating the usual (but generalized to many states) QED models such as Jaynes-Cummings (JC), Rabi, and the rotating wave approximation (RWA), as well as an approximation to the electronic subsystem within the Tamm-Dancoff approximation (TDA). Panel (a) is adapted with permission from ref (115). Copyright 2021 American Institute of Physics. Panel (b) is adapted with permission from ref (203). Copyright 2020 American Physical Society.


A graphical representation of these partitioned and coupled excitations can be found in Figure 7a. The amplitudes *t*_*α*_, *t*_*n*_, and *t*_*ãñ*_ can be solved by projection (eq 130). This requires to evaluate , which is the similarity-transformed Hamiltonian operator, where *Ĥ*_PF_ is expressed in eq 104 and is usually rotated to the coherent state basis (see eq 120). This leads to the ground state energy as a solution to the following set of equations,

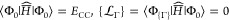
130with |Φ_{Γ}_⟩ = τ̂_{Γ}_ |Φ_0_⟩, where {Γ} is the set of possible excitations in the cluster operator  leading to the set of projection equations . These projections lead to the equations for the excitation amplitudes *t*_{Γ}_ and are usually solved in a self-consistent manner.

There are many different notations for the methods developed by changing the highest level of excitation for each term in the cluster operator. In this review, we will use the notation whereby CCSD-*n*-jm, which implies that the electronic DOFs are treated up to double excitations in the cluster operator, the photonic excitation is limited to *n* levels, and the mixed excitation is set to j electronic and m photonic. As per usual CC theory, the cutoff of excitation level leads to effects that include yet higher excitations through the exponential treatment of the cluster operator , thus effectively outperforming similar methods like CI with the same excitation level cutoff. However, due to the  scaling (with *N* electrons and  cavity modes each with  Fock states) of the scQED-CC method in general, including more than two Fock states has been a challenge even for small molecular systems,^122,125,195,204^ and limited study has been performed including up to 10 Fock states for a half-filled four-site Hubbard model with direct comparison to the full configuration interaction result.^203^ This will have unfavorable scaling on low-frequency cavities or for purposes of multiphoton up-conversion, where higher numbers of photons are required to resolve the physics.

#### 3.2.3 QED Equation of Motion Coupled Cluster Theory

The excited states in the CC theory are generated most naturally by the equation of motion (EOM) formalism, which is often referred to as the EOM-CC approach, whereby the excited wave functions are generated through the Jacobian matrix defined as the derivative of the projected eqs (eq 130) with respect to the cluster amplitudes *t*_*α*_ as,^198−202^
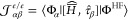
131where |Φ_α_⟩ is is defined below eq 130 and |Φ^HF^⟩ is the exact ground state This leads to the following non-Hermitian Hamiltonian for the bare electronic system,^205^
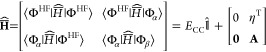
132where the explicit elements (as well as additional discussion) for the vector **η** and matrix **A** can be found in ref (205). Extending the CC formalism to the coupled electron-photon system, we have
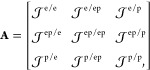
133where each block Jacobian matrix  mixes various DOFs through coupled excitations in the individual or mixed subspaces. Note that  is the same as outside the cavity, and the rest of the Jacobian matrix elements can be written similarly as follows
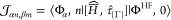
134where τ̂_{Γ}_ can be either the electronic *τ̂*_*β*_, photonic *τ̂*_*m*_, or mixed electronic-photonic *τ̂*_*β̃m̃*_ excitations. These coupled equations are usually solved via iterative diagonalization for the amplitudes in the standard coupled-cluater implementations. For more details on the exact expressions for ground and excited polaritonic amplitude equations, see refs (122, 206, and 203).

#### 3.2.4 QED Density Functional Theory

In this section, we turn to a different and robust approach to include explicit electron-photon correlation, using density functional theory (DFT) approaches. DFT^207^ is formally exact, up to the choice of the exchange correlation functional, which is currently not known. To make it practical for realistic systems, multitudes of approximate density functionals have been developed with varying complexity that involves different orders of derivatives on the electronic density in order to capture long-range correlation. For the electron-photon hybridized system, one must extend this ideology to include correlations between the electronic and photonic subsystems, which has only recently been studied.^182,208^ We will only give a general outline of this approach based on ref (182), which uses the optimized effective potential (OEP) approach to generate a simple functional to include electron-photon exchange interactions. However, other discussions related to the recent advances of the scQED-DFT approach can be found elsewhere in the literature.^209−212^

DFT uses the total density, *n*(**r**), as the main variable. For cavity QED, the photon provides additional DOFs, notably the photonic coordinate *q̂*_c_, which we will see is hidden in new single-particle (SP) orbitals that can be interpreted as corrections to the original SP states due to the cavity. The DFT equations for the noninteracting Kohn–Sham (KS) system can be written as
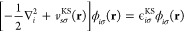
135where *i* labels the noninteracting KS orbitals {*ϕ*_*iσ*_} with spin σ. The total density *n*(**r**) is computed as the sum of the spin densities *n*_σ_ = ∑_*i*_*ϕ*_iσ_^*^ϕ_*iσ*_. The effective KS potential is written as,
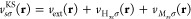
136where *v*_ext_ is the usual external potential, *v*_H_xc_σ_ is the electron–electron exchange-correlation, and *v*_*M*_*xc*_*σ*_ is the cavity-dependent exchange-correlation potential. Both *v*_H_*xc*_*σ*_ and *v*_*M*_*xc*_*σ*_ contain unknown exchange-correlation functionals. The ground state is a simple case where the exchange-correlation energy can be written as,
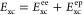
137where *E*_xc_^ee^ is an exchange-correlation term between electrons and *E*_xc_^ep^ is an exchange-correlation term between electrons and photons with corresponding potential,
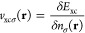
138

Here, ref (182) asserts an additional approximation such that only the exchange energy is accounted for as *E*_xc_ ≈ *E*_x_^ee^ + *E*_*x*_^ep^ = E_*x*_. In the ground state, only the second-order exchange energy contributes to the total energy (i.e., only the DSE term) The electron-photon exchange energy *E*_x_^ep^ = ∑_σ_∑_*i*_ (*E*_x_^ep^)_*iσ*_ can be written purely as a functional of the KS orbitals {*ϕ*_*iσ*_} and two orbital shifts {Φ_*iσ*_^(1)^} and {Φ_*iσ*_^(2)^} (to be interpreted as the KS orbital response to the cavity field) as,
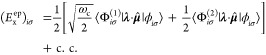
139where the “c.c.” term in above equation indicates to add the complex conjugate of all preceding terms. Here, the two orbital shifts can be written in terms of the KS orbitals themselves as follows
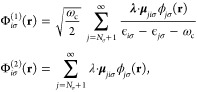
140with a total of *N*_*σ*_ occupied KS orbitals of spin σ.

Using these expressions, one can take explicit functional derivatives of *E*_*x*_ (see eq 138) with respect to {*ϕ*_*iσ*_}, {Φ_*iσ*_^(1)^}, and {Φ_*iσ*_^(2)^} to obtain the total OEP exchange potential *v*_xσ_ (*r*) used in the DFT formulation and further iterated to obtain the electronic density *n*(*r*) under the influence of the cavity.

One possible observable that can be computed from the photonic subspace is the average photon number in the ground state *N̂* = ⟨*a*^†^*â*⟩ and can be written in terms of the orbital shifts {Φ_*iσ*_^(1)^} as,^182^
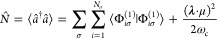
141

Note that the average photon number in the PF Hamiltonian (PZW frame) is not the same as the photon number in the p·A Hamiltonian (Coulomb gauge), which is usually interpreted as the physical photon number. See more detailed discussions around eq 64. As such, the quantity written in eq 141 may be more appropriately referred to as the mode occupation in the PZW frame. Here, the first term represents the one-photon wave functions that arise due to the quantum fluctuations of the photon while the second term is the correction due to the dipole self-energy contribution (after variational SCF procedure is performed).^182^ For the sake of brevity, we refer the reader to refs (208) and (213) for additional details and discussion on the scQED-DFT formulation.^73,209,209^

#### 3.2.5 QED Time-Dependent Density Functional Theory

The time-dependent analogues of single-particle approaches have proven to be powerful probes of nonequilibrium states that give rise to electronically excited distributions. Linear response (LR), one of the most popular formalisms, results in the LR-TD-HF and LR-TD-DFT methods in the random phase approximation (RPA). Although, it should be noted that the real-time propagation of the single-particle density matrix–leading to the real-time TD-HF and real-time TD-DFT approaches–is, in principle, a more robust approach that captures many finer details of the nonequilibrium state.

Note that these real-time TD-DFT schemes have already been developed for the simulation of molecular polaritons using classical photon DOFs^214,215^ as well as fully quantum approaches^210,216^

In this section, we focus on the LR formalism using the random phase approximation (RPA) for its potential wide use in the community for a broad range of applications to both small and large molecular systems, originally formulated by Flick and co-workers^121,217^ using the QEDFT method (or scQED-DFT in the notation of this work) in the language of Casida and further used/extended by the groups of Shao^115^ and DePrince.^175^ It should be noted that other formulations of CIS-like excited polariton states can be found in the community, such as the non-Hermitian configuration interaction singles (CIS) aimed at simulating cavity loss via a complex photon frequency.^184^

Time-dependent DFT (TD-DFT) in the linear response framework^218^ has been used ubiquitously over the last couple decades to describe electronic excitations in all manner of chemical systems, due to its computational simplicity and feasibility for large systems from 100s to 1000s of atoms in size.^187,218−233^ For the cavity QED community, it is natural to extend this powerful method to describe the coupled electron-photon system. Recent work on developing a new scheme to include the additional photonic DOFs are underway with promising results in a variety of molecular systems.^72,73,115,121,125,181,182,208−210,212,213,217,234−239^ The Casida-like generalized eigenvalue equation can be recast in an increased dimensional space to include the excitation and de-excitation transition densities for both the electron and photon subsystems including all terms in *Ĥ*_PF_ (eq 55).

This approach was first rigorously formulated in ref (217) by considering the response of the approximate ground state density generated by the scQED-DFT formalism. Further work by Shao and co-workers^115^ has led to a similar scQED-LR-TD-DFT formulation without the use of the ground state scQED-DFT. Instead, it uses the unperturbed ground state electronic Kohn–Sham orbitals as input. More specifically, ref (115) relies on the uncorrelated ground state of the bare electronic system, where the only light–matter interaction and self-consistency is found in the iterative diagonalization of the RPA equations themselves. Ref (217) relies on the combined effort of the ground state response (self-consistent cycle) to the photon field followed by the linear response self-consistency. While the approach in ref (217) is no doubt more rigorous and reliable, given a good enough exchange-correlation functional for both the electron–electron and electron-photon interactions, the simplified approach of ref (115) was chosen as the workhorse for this section of the manuscript. Although we acknowledge that the approach in ref (115) is only approximately capturing the response to the electronic subsystem and may require convergence of the virtual single-particle orbitals (i.e., increased number of singly excited Slater determinants used in the RPA equations) to completely validate the results, which is discussed in the Supporting Information of ref (115).

The central result of ref (115) is the following generalized Casida equation^115^
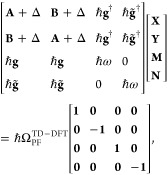
142where ℏΩ_PF_^TD-DFT^ are the TD-DFT excitation energies (for the Ĥ_PF_), **X**, **Y** and **M**, **N** are the electronic and photonic transition density matrices, respectively, **Δ** is the DSE term (eq 52), and *ℏ***g** is the light–matter coupling term. The nontraditional matrix elements are constructed as^115^ Δ_*ov*,*o*′*v*′_ = (**μ**_*ov*_ · **λ**) (**μ**_*o*′*v*′_ · **λ**), , , where **A**, **B** are the usual electronic TD-DFT matrix element blocks^218,225^ for the particle-hole and hole-particle components, respectively, and ω_c_ and **λ** are the cavity frequency and coupling strength, respectively. The indices {*o*, *o*′}, {*v*, *v*′} correspond to occupied and unoccupied Kohn–Sham orbitals, respectively.

There are two important things to note about this scQED-TD-DFT procedure:^115^ (i) Only a single Fock state was included in the off-diagonal coupling blocks *ℏ***g** (which is standard in a linear-response regime where only single excitations are constructed). However, additional photonic basis states become extremely important at large coupling strengths,^203^ which is not included in this scQED-TD-DFT method. It is our own opinion that one should seek a scQED-TD-DFT method which allows for the inclusion of additional Fock states to converge these interactions. This is not straightforward in the language of linear response, but it may be possible to add an additional step for self-consistent formulation of the scQED-TD-DFT (which accounts for the singly excited subspace of electron and photonic DOFs) to include a pQED-like diagonalization with additional excited photon states. It may be possible to construct such a scheme that would allow for additional relaxation to the singly excited subspace, even if in an ad hoc way.

(ii) This work^115^ does not inherently rely on the use of the ground state scQED-DFT method discussed previously^121,217^ and can be instead coupled with any molecular ground state as computed by any exchange-correlation functional and further used in the canonical TD-DFT blocks **A** and **B**. In this way, the ground state orbitals (i.e., Kohn–Sham basis states) themselves do not directly respond to the presence of the cavity through self-consistent iteration, through dipole self-energy^122,240^ nor through electron-photon correlation built in to the exchange-correlation functional,^209−212^ but instead only interact with the cavity through the iterative diagonalization cycles that provide the excited states (i.e., Lanczos/Davidson^188,241,242^ algorithms).

A recent work has explored this topic at length and provided important results regarding the mean-field approaches and the relaxation/response of various self-consistent schemes to the electron-photon interaction.^240^ Similar to the previous pQED scheme (see Section 3.1), this is an approximate solution to the excites states given a finite number of unperturbed electronic basis states (i.e., virtual orbitals included in the singly excited Slater determinant basis expansion) since the Kohn–Sham basis is frozen throughout the iterative cycles. Other works, such as those in refs (217, 234), and (175) to name a few, solve the scQED-TD-DFT RPA equations using the self-consistently relaxed Kohn–Sham orbitals. To be clear, the scQED-TD-DFT used in ref (115) is a particular case of the scQED-DFT scheme where the electron-photon exchange-correlation kernel is neglected, leading to unperturbed ground state single-particle orbitals used in the solution of the RPA Casida equation. We believe that an important consideration needs to be made clear in that the inclusion of the electron–photon exchange-correlation kernel may lead to interesting effects. Often, a reduction in electron–electron exchange-correlation is made and may lead to an overall reduction in the ability of the scQED-DFT single-particle orbitals to capture the correct physics of the bare molecular system. Although, with the advent of novel electron-photon exchange-correlation functionals appearing, this trade-off will hopefully be reduced and will replace the bare electronic exchange-correlation functionals of similarly high quality.

Various approximations can be achieved by setting different blocks of eq 142 to zero. The usual Tamm-Dancoff Approximation (TDA) can be achieved by setting **B** = **0**, while other QED Hamiltonians (in their many-state generalizations) can also achieved. For example, setting **g̃** = **0** is the generalized RWA, while also setting the DSE term to zero (Δ = 0), one arrives at an analogue of the JC Hamiltonian. These various choices have been extensively discussed in ref (115), and is schematically depicted in Figure 7b.

Other similar forms of the TD-DFT and CIS equations have been derived for the scQED scheme, such as those presented in refs (121, 175, and 184), respectively, where all approaches yield similar results. In principle, in all methods discussed until now, an arbitrary number of photonic basis states can be included in order to converge the photonic contribution with little-to-no increase in overall expense due to the relative simplicity of the photonic subsystem compared to the electronic one. Although more work is needed to test the results against an increasing number of photonic basis states when using these QED approaches, most of the work usually only includes the vacuum state |0⟩ and |1⟩ Fock state. In the previous section on wave function-based methods and specifically with the QED coupled cluster methods, more work has been done to test such convergence. However, due to the high computational expense of these QED-CC methods themselves, the cost becomes too large to include more than a couple photonic states yet still does not capture qualitative features (such as the number of avoided crossings between polariton states), especially when examining higher-energy polaritonic states for low cavity frequencies (with respect to electronic transitions), in comparison with a full configuration interaction (FCI) approach as shown in such model systems.^203^ The current status of these scQED methods is quite limiting in the sense that either additional Fock states cannot be added to the formulation in a straightforward way (e.g., scQED-TD-DFT) or are simply too expensive to include in most situations (e.g., scQED-EOM-CC).

### 3.3 Recent Results in the Calculation of Ab initio Polariton States

#### 3.3.1 Polaritonic Excited States

Historically, the development of electronic structure methods started in the ground state with HF, DFT, CC, etc. methods and then moved to the excited state with TD-HF, TD-DFT, EOM-CC, etc. The recent development of scQED methods took a similar path, but the production of ground and excited state methods largely overlapped due to the already available electronic structure theory for solving complex many-body Hamiltonians. In the following, we will review some recent studies using scQED as well as pQED schemes, but we will begin with our discussion for the excited state. This is more akin to the original context of quantum optics decades ago, where coupling a cavity to a single atomic transition (ground to excited electronic excitation) was prevalent, as illustrated in the simple features of the Jaynes-Cummings Hamiltonian (Figure 1c).

**Figure 8:**
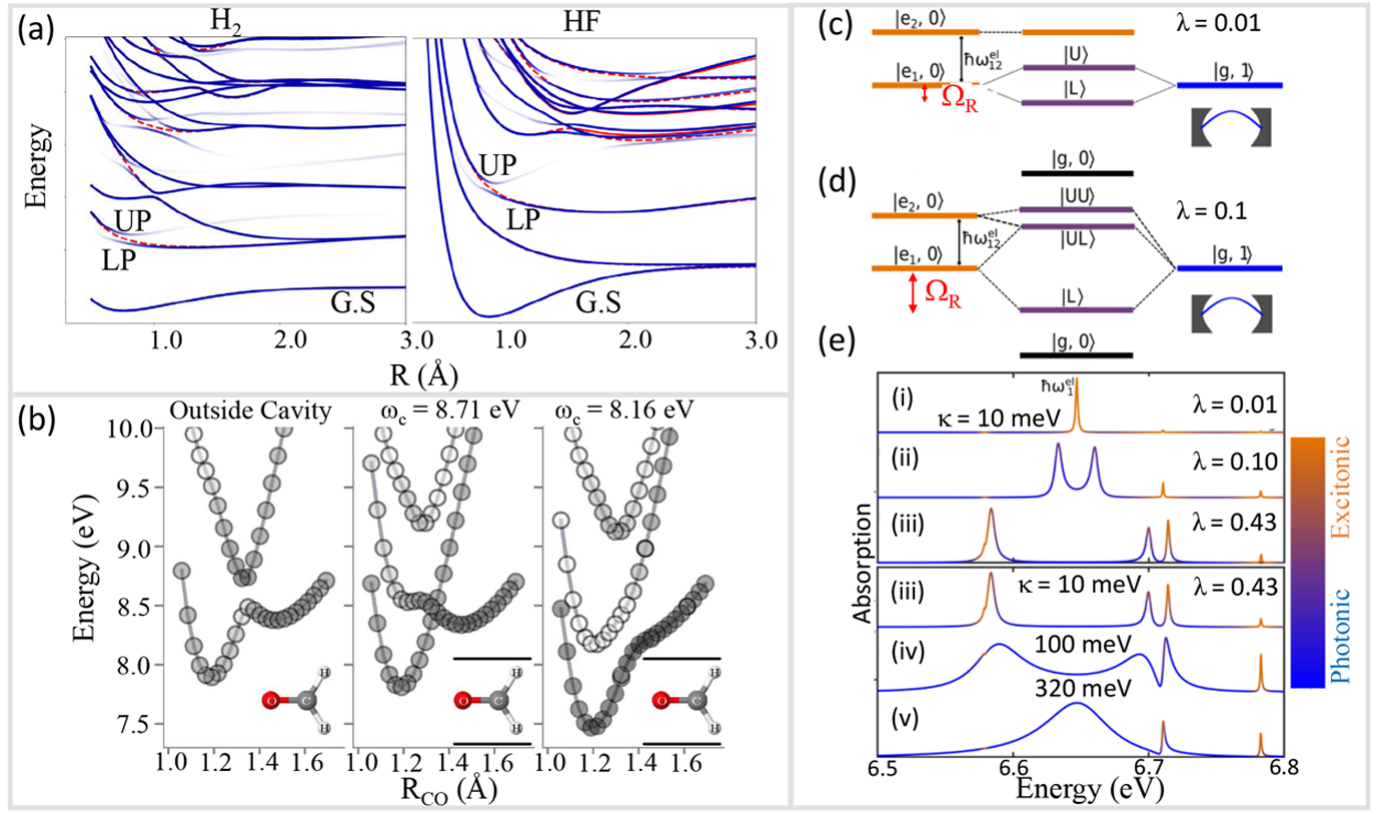
Ab initio electronic-photonic structure for polaritonic excited states. (a) scQED-EOM-CC scheme exploring H_2_ and HF dissociation potential energy surfaces (PESs) inside a cavity. For each molecule, the cavity frequency is in resonance with the first bright excitation at the equilibrium geometries with polarization along the bond axis with coupling strength λ = 0.05 au The polaritonic levels are shown in blue, while the bare molecular levels are shown in red. The lowest-energy levels participating in electron-photonic hybridization are labeled as LP and UP in each panel. (b) PESs are shown the formaldehyde C–O bond stretch (left) outside the cavity and inside two cavities of frequencies ω_c_ = 8.71 (middle panel) and ω_c_ = 8.16 eV (right panel), respectively, for a coupling strength λ = 0.04 au and polarization along the C–O bond axis. (c,d) Hybridization diagrams and (e) absorption spectra for the toluene molecule. (c) Small coupling λ = 0.01 au leads to an effective two-level system, while (d) at larger coupling λ = 0.10 au higher electronic excited states become important. (e) The absorption spectra at small cavity loss rate κ = 10 meV for multiple coupling strengths λ = (i) 0.01, (ii) 0.10, and (iii) 0.43 au and at large coupling strength λ = 0.43 au for varied cavity loss rates κ = (iii) 10, (iv) 100, and (v) 320 meV. The colorbar indicates the electronic and photonic character. 36,000 external cavity modes were usedd to model the cavity loss in this effective single-mode cavity. Panel (a) is adapted with permission from ref (122). Copyright 2020 American Physical Society. Panel (b) is adapted with permission from ref (121). Copyright 2020 American Institute of Physics. Panels (c–e) are adapted with permission from ref (236). Copyright 2021 American Institute of Physics.

Figure 8 presents a few examples that illustrate the modifications of the excited state electronic structure (or potential energy surfaces) when forming molecular exciton-polaritons. Figure 8a is one of the first examples^122^ of scQED calculations using the equation-of-motion coupled cluster (EOM-CC) approach (scQED-EOM-CC) to examine the polariton potential energy curve *ε*_*a*_(*R*) (see eq 108) of H_2_ (left panel) and HF (right panel) when coupled inside a cavity, with the field polarization along the bond axis of each molecule and with a coupling strength λ = 0.05 au The upper (UP) and lower (LP) polaritons are labeled to indicate the location of the main Rabi splitting caused by the |*g*, 1⟩ and |*e*, 0⟩ hybridization (as explained by simple JC model in eq 6), but the presence of the many-electronic-states and additional many photon-dressed adiabatic states make the UP/LP picture (by the JC model) overly simplified. In Figure 8a, the blue solid lines indicate the excitonic character, the white solid lines indicate photonic character, and the red dotted lines indicate the original electronic states outside the cavity. Using the photon-dressed electronic states, one can manipulate and tune the excited state potential energy surfaces to mediate additional transitions or eliminate them.

For H_2_, the cavity frequency is close to the first singlet electronic transition (at the Franck–Condon region of the nuclear DOF). The modifications to the excited state PES curvatures can be seen by the induced localization of the UP state, which possesses a minimum near the Franck–Condon point while the original molecular PES (red dashed line) has a purely dissociative character. For the HF molecule coupled to the cavity (right panel), the cavity frequency is near resonant to the ground to the second excited electronic transition. Similar features of the polariton potential can be obtained when this polar molecule (which possesses a permanent ground state dipole) is coupled to the cavity. The authors of that work^122^ thus concluded that the permanent dipole does not induce additional interesting effects, which is accurate for this particular system. However, as a reminder of what was discussed previously (eq 5), not only the ground state permanent dipole contributes to the light–matter interactions, all excited state permanent and transition dipoles will, in principle, contribute interesting effects. The effects of the permanent dipoles have been studied in many recent works,^48,51,122,124,184,195^ which have noted that the molecular dipole along the direction of the cavity polarization becomes reduced through interaction with the cavity.

As a small note, the energetic alignment and multitude of electronic states plays an important role in photophysical properties and interpretations of the polariton states. For example, the commonly used language of “upper polariton” and “lower polariton” could potentially be misleading if the system has many electronic states nearby in energy, as is the case for the H_2_ and HF examples presented in this panel.

Figure 8b presents another recent example of scQED simulations^121^ for obtaining polariton potential energy surfaces _*a*_(*R*), using the scQED-TD-DFT level of theory to investigate a formaldehyde molecule coupled to the cavity. Outside the cavity (left), an avoided crossing can be found along the C–O bond stretching coordinate near to *R*_CO_ ≈ 1.35 Å. The shading of the curves in this panel indicates the magnitude of the electronic transition dipole moment between the ground and excited state.^121^ At a large cavity frequency ω_c_ = 8.71 eV, the avoided crossing can be reduced by the couplings between the photon-dressed ground state |*g*, 1⟩ and the higher-energy excited state with zero photons. At a slightly smaller cavity frequency ω_c_ = 8.16 eV, the original potential energy minimum near *R*_CO_ = 1.5 Å (for the bare molecule) can now be completely removed through the light–matter potential curvature hybridization, tilting the polariton potential all the way back to a global minimum energy located at the Franck–Condon point of *R*_CO_ ≈ 1.2 Å. This work,^121^ demonstrates that by forming polaritons, one can in principle manipulate photoexcited reactions via modification of the excited state pathways and curvatures.^10,21,72^

Figure 8c presents the first few polariton states generated from coupling a toluene molecule (under the cavity-free equilibrium nuclear geometry) to a single mode cavity. In particular, the first two electronic excited state states (orange) and one photon-dressed ground state (blue) are shown. At a weak coupling of λ = 0.01 au and a cavity frequency that is in resonance with the first electronic excitation, the electronic and photonic DOF strongly mix and generate the polariton states (purple), resulting in the usual UP and LP polariton states. The second excited states, due to their off-resonant frequency, is not explicitly involved into polariton formation under this particular coupling strength. For a larger coupling strength λ = 0.10 au (Figure 8d), the Rabi splitting Ω_R_ (red arrow) becomes large enough to mix the UP with the second excited electronic state, thereby forcing the change in terminology to now include three polaritons: the LP, the upper lower polariton (ULP) and the upper upper polariton (UUP). In this case, the LP and ULP are strongly coupled through the light–matter interaction, while the ULP and UUP are interacting via the derivative couplings from the bare electronic interactions and DSE couplings mediated by the cavity. This will lead to interesting dynamical interplay between all DOFs; this was done explicitly in ref (236) using a simplified pQED Hamiltonian based on the data in Figure 8e to perform model polariton dynamics to elucidate the dynamical effects of a multilevel system.

Figure 8e presents the results of absorption of the same toluene-cavity hybrid system. In particular, the work in ref (236) uses scQED simulations to directly examine the condition to achieve a strong coupling by incorporating cavity loss into the analysis. This is done through broadening of the cavity coupling strength across a multitude of cavity modes localized at the primary cavity frequency ω_c_ following a Lorentzian broadening of varied width κ (i.e., loss rate). The coupling strength λ (eq 105) is distributed across the multitude of modes in the spectral function (i.e., a Lorentzian) such that
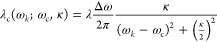
143where the original coupling strength λ has been broadened by the Lorentzian function, with Δω = *ω*_*k*__+1_ – *ω*_*k*_ as the discrete mode frequency separation, ω_c_ as the central mode frequency, and *ω*_*k*_ as the frequency of the *k*_th_ mode. In this work,^236^ 36 000 cavity modes were used to mediate the cavity loss effects for an effective single-mode cavity. Recall that λ is a generalization of the commonly used Jaynes-Cummings coupling strength *g*_c_ and can be related as  in eq 3. This approach to cavity loss is, in principle, equivalent to adding a photonic bath to the cavity mode *q̂*_c_ (see eq 186 in Section 4.7). The loss rate κ can be either calculated with knowledge of the cavity setup using methods such as the transfer matrix method[1] or directly measured from experiments by the width of the absorption peak of the bare cavity assuming that the spectral width of the bare cavity is dominated by the photonic loss, which is a good approximation for most realistic experimental configurations. In Figure 8e, the absorption spectra (see details in Section 3.3.3) was computed^121^ using the effective polaritonic dipole by mixing the electronic dipoles according to expansion coefficients of the adiabatic electronic and Fock basis states **μ**_0α_^pol^ = ∑_*j*_^*N*_*el*_^*c*_*j*_^α^**μ**_0*j*_^el^, where  is the number of included electronic states.^121^

Recall that the strong coupling in cavity QED is commonly defined as *g*_c_ ≫ κ, where *g*_c_ is the matter-cavity coupling strength and κ is the cavity loss (if matter de-excitation rate is much smaller than the cavity loss). In this example (Figure 8e), the absorption spectra (calculated using eq 147) is shown for different cases of cavity strength λ and cavity loss rate κ, which will turn the light–matter couplings from the weak coupling (no Rabi splitting) to the strong coupling regimes (has Rabi splittings). The excitonic character (orange) and the photonic character (blue) are depicted in the color bar in Figure 8e. For each subpanel, (i) at a low coupling strength (λ = 0.01 au) and cavity loss κ = 10 meV, the Rabi splitting is not visible in the spectral resolution, and the feature is dominated by the excitonic character; (ii) at a larger coupling strength (λ = 0.1 au) and the same κ = 10 meV, the Rabi splitting is clearly visible; however, the second excited state is not affected by the presence of the cavity due to the large energetic separation (detuning) between the UP state and the second electronic excited state. Now, the main polaritonic absorption features are mixed between electronic and photonic contributions. (iii) at a very large coupling strengths (λ = 0.43 au), the UP polariton state now strongly mixed with the second excited electronic state and forms the ULP and the UUP polariton states.^121^ Further, one can fix the coupling strength λ and gradually increase the loss rate κ. (iv) For the case of the very strong coupling λ = 0.43 au, the cavity loss rate is increased from κ = 10 meV to κ = 100 meV, effectively increasing the spectral signature of the cavity modes centered at ω_c_. Here, the character of the absorption is dominated by the photonic DOF (blue color), and the width of each main feature in the absorption becomes much broader compared to the case in (iii). (v) At an even larger cavity loss rate κ = 320 meV, the identities of the LP and ULP start to disappear, leaving only a single broad peak centered at ω_c_ with dominating photonic character. This effectively returns the system back to the weak coupling regime, due to the light–matter coupling strength is now much smaller than cavity loss. The second excited electronic state is nearly unperturbed now due to the decoupling between the cavity modes and the electronic states. Note that the absorption spectra is arbitrarily scaled to showcase the features and may not reflect the exact nature of the spectra.

#### 3.3.2 Computing Polariton Properties

Now we move to more examples of using the scQED approaches to analyze excited state properties of molecular exciton-polaritons. There are many quantities in the electronic structure community in determining the character of an excitation, such as natural transition orbitals,^243−245^ transition density,^121,187,244,245^ difference density,^122,195,244,245^ electrostatic surfaces/charges,,^246,247^ etc. In principle, they can all be generalized for polaritons and be able to used to characterize the nature of polaritons. Our main focus here is on the transition density,^121,187,234,244,245^ which is the most straightforward quantity that can be obtain from electronic structure packages. The one-electron polaritonic transition density between the ground and *a*_th_ polaritonic states can be written as
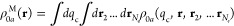
144where *q*_c_ is the photonic coordinate of the cavity and **r**_*j*_ is the *j*^th^ electronic coordinate of *N*_*e*_ electrons.^121^ Here, ρ̂_0*a*_ = |Ψ_*a*_⟩⟨Ψ_0_ | is the usual transition density operator from ground to the *a*_th_ excited polaritonic state. One can also show that this one-particle polaritonic transition density can be written in terms of the bare one-particle electronic transition densities according to the expansion coefficients *C*_*αm*_^*a*^ (i.e., after diagonalizing the pQED Hamiltonian in eq 112 with the adiabatic-Fock basis defined in eq 111) as
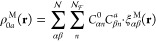
145where ξ_*αβ*_^M^ (**r**) = *ψ*_*α*_ (**r**)ψ_β_^*^ (**r**) is the bare one-particle electronic transition density between adiabatic states *ψ*_*α*_ and *ψ*_*β*_ and *C*_*βn*_^*a*^ is the *βn*^th^ expansion coefficient for the *a*_th_ polariton (see eq 111), and  and  indicates the total number of adiabatic states and Fock states, respectively. Further, *ψ*_*α*_(**r**;**R**) = ⟨**r**|*ψ*_*α*_(**R**)⟩ are the many-electron adiabatic states outside the cavity. Note that since the Pauli-Fierz Hamiltonian (eq 104) is purely real, the coefficients {*C*_*αm*_^*a*^,*C*_*βn*_^*b*^} are also real. In the matter-projected polaritonic transition density ρ_0*a*_^M^, the photonic DOFs were traced out, leaving only linear combinations of electronic matrix elements of the same photon number (i.e., *n* = *m*). Using these simple expressions, one can easily compute any polaritonic observables from the pQED scheme, relying on electronic quantities from widely available electronic structure codes as well as benefiting from the simplicity of the photonic subsystem.

**Figure 9:**
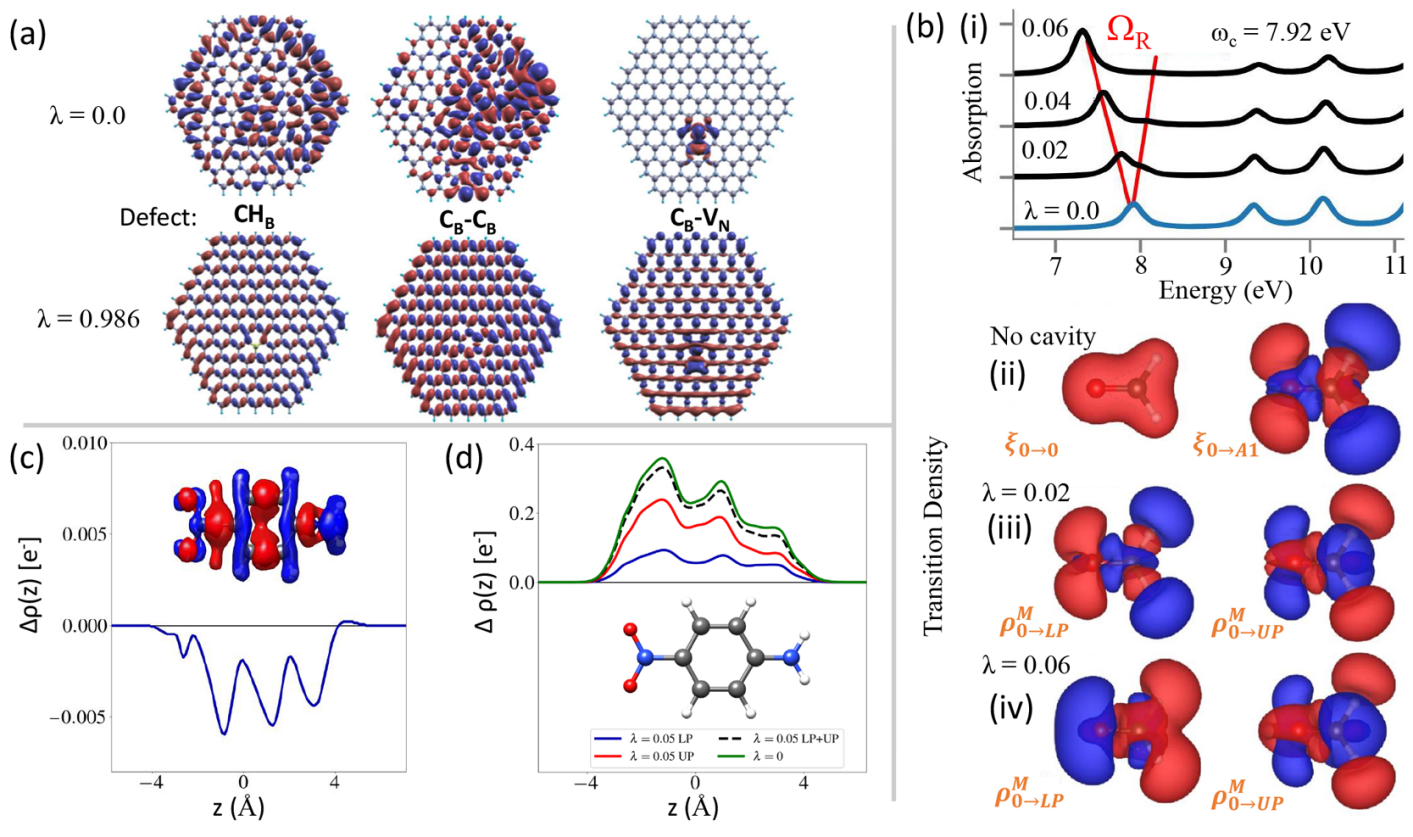
Ab initio electronic density and transition density analysis. (a) Transition density (top) outside and (bottom) inside a cavity with coupling strength strength λ = 0.986 au of graphene flakes (or quantum dots) with three types of localized defects: (left) CH_*B*_, (middle) C_*B*_–C_*B*_, and (right) C_*B*_–V_*N*_. (b) (i) Absorption spectra of formaldehyde at varying coupling strengths λ = 0.0, 0.02, 0.04, and 0.06 au for cavity frequency ω_c_ = 7.92 eV. (ii) (Left) Ground state total density and (right) bare molecular transition density (λ = 0.0 au) (iii,iv) Matter-projected transition density for the (left) lower and (right) upper polaritons at λ = (iii) 0.02 and (iv) 0.06 au (c) Ground state density difference function for a charge-transfer benzene derivative with amino- and nitro- groups in the *para* position (see panel d). the cavity frequency was set to ω_c_ = 4.84 eV with coupling strength λ = 0.05 au (d) Excited-ground density difference function with the same parameters as in (c). Panel (a) is adapted with permission from ref (234). Copyright 2021 American Chemical Society. Panel (b) is adapted with permission from ref (121). Copyright 2020 American Institute of Physics. Panels (c and d) are adapted with permission from ref (122). Copyright 2020 American Physical Society.

Further, eq 145 can be generalized to the following structure to include any one-particle electronic or photonic observables
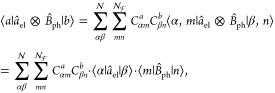
146where *Â*_el_ and *B̂*_ph_ are any one-body operators in the electronic and photonic subspaces, respectively. Here, *Â*_el_ or *B̂*_ph_ may be the dipole, excitation number, total density, transition density, etc. operators from the respective subspaces. For example, one can compute the exciton-polariton absorption spectra, *A*(*E*), shown in Figure 8e and Figure 9. Here, the polaritonic transition dipole matrix element **μ**_0*a*_^pl^ can be computed using eq 146 as  where  is the identity operator in the photonic subspace. With this expression, the absorption spectra can be written as
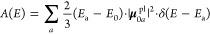
147

Note that the delta-function is usually broadened with a normalized, finite-width Gaussian or Lorentzian function to account for excitonic, photonic, and/or environmental relaxation/broadening processes present in realistic experimental conditions. In principle, another term should be added to account for the photonic part of the absorption/emission, which is proportional to  ∼  = . However, the relative magnitude of the electronic and photonic contributions in experiment is extremely reliant on the experimental setup (e.g., cavity loss, direction of the probe etc.). Other works have used different quantities to explore the cross-correlation of various observables for the spectroscopic analysis of molecular systems in cavities.^209^ In experiment, usually the photonic contribution to the absorption and emission will dominate the intensity of the spectrum in Fabry–Pérot-type cavities.^248,249^ however, for theoretical calculations, the excitonic absorption spectra expressed here and in other works^115,121,217,236^ is better-suited to understand the effects of the cavity on the electronic subsystem and gives more direct insight into the local reactivity and electronic reorganization in the molecule upon excitation. It is also important to recall that most of these reported results are in a single-mode cavity and, in principle, only represent the θ = 0 special incident angle in a FP cavity (see Figure 4 and Sec. 2.6).

Figure 9a,b presents the polariton transition density when coupling matter to an optical cavity.^234^ The molecular transition density indicates the electron and hole overlap in real space and provides information regarding the localization of the exciton and on molecules, which may provide useful insights into possible reactive bonding sites upon photoactivation.^187,244,245^Figure 9a showcases the polaritonic ground-to-excited transition density^234^ (see eq 145) for three defected hexagonal boron nitride quantum dots: (left) carbon substitution at a boron site CH_B_, (middle) carbon substitutions at *meta*-boron sites C_B_–C_B_, and (right) carbon substitution and adjacent nitrogen vacancy C_B_–V_N_. Outside of the cavity (top row), these defects each have a unique low-lying exciton of varied localization character. The nitrogen vacancy C_B_–V_N_ (right) presents the most localized features in the transition density (where the electron and hole are strongly overlapped only in this region near the defect). When coupling the system inside a cavity (bottom row), the transition density for all species becomes mostly delocalized. This delocalization in the transition density facilitates an increase in polaritonic dipole moment and hence the increase in the lowest absorption peak in all species. Here, the tunability over the bright, low-lying transition in defected boron nitride quantum dots has been achieved through cavity QED.

Figure 9b, presents the lowest bright excitation in formaldehyde when it is coupled to a cavity. In panel (i), when varying coupling strengths λ = 0.0, 0.02, 0.04, and 0.06 au, one can clearly see an an increasing Rabi splitting Ω_*R*_ in the absorption spectra. The transition density of the bare molecular system is shown in panel (ii) right figure while the ground state density is shown in panel (ii), left figure. Inside the cavity, for a weak coupling (λ = 0.02 au), the transition density from the ground state to the upper polariton (iii, right) and to the lower polariton state (iii, left) indicate a significant modification of the excitation character compared to the transition density for the bare molecule. Similarly, at increased coupling strength (λ = 0.06 au). the transition density continues to change, although, the ground to the lower polariton transition (iv, left) is significantly modified compared to the case in (iii, left).

Another analysis technique common to electronic structure theory is the density difference function, which is capable to illustrate the change of the electron distribution in a molecule upon excitation. More specifically, these can be defined in two ways: (I) the difference between the density of polaritonic state |Ψ_*a*_⟩ inside and an analogous electronic state outside the cavity |*ψ*_*α*_⟩ ⊗|*n*⟩ (usually the ground polariton state and the ground electronic states) and (II) the difference between the density of one polaritonic state |Ψ_*a*_⟩ and another state |Ψ_*b*_⟩ (usually for ground and an excited polariton state). Figure 9c describes the density difference of type (I) for the ground state while Figure 9d for the same molecular shows the difference density of type (II) for the polaritonic excited state and ground state.^122,250^

In Figure 9c, the ground state density difference (Δρ(*z*) = ρ_00_^cav^ (*z*) – ρ_00_^nocav^ (*z*)) is presented where the cavity (with coupling λ = 0.05 au) is placed in resonance with the lowest-lying charge transfer state (from NH_2_ group to the NO_2_ group, see panel d for molecule) and showcases a modulation of the ground state indicating charge displacement, where blue and red isosurfaces represent charge accumulations and depletions, respectively. A charge migration of −0.005 |e|, induced by the cavity, is seen from the *acceptor* (NO_2_) to the nitrogen atom of the *donor* (NO_2_), effectively reducing the ground state dipole from 6.87 to 6.77 D. This reduction in dipole moment is thought to by a direct result of the cavity inducing charge migration in order to reduce the variation in the dipole Δμ̂ (see discussion near eq 124).^182^ Here, Δρ(*z*) = ∫_-∞_^∞^*dx*∫_-∞_^∞^*dy*∫_-∞_^z^*dz*′ Δρ(*x,y,z*′). Here, the authors have directly shown that the ground state density is modified by mixing with excited electronic states and adopting their character via coupling through the cavity. Similarly, in Figure 9d, the ground/excited state density difference can be plotted (Δρ(*z*) = ρ_E.S._ (*z*) – ρ_G.S._ (*z*)) to showcase the effects of the cavity on the exited state character with respect to the polaritonic ground state. Here, the charge displacement is seen moving from the *donor* (NO_2_) to the *acceptor* (NH_2_) species with a magnitude of −0.4 |e|. The upper (red) and lower polaritons (blue) observe different amounts of charge displacement, but the sum of the two (dashed black) nearly reproduce the original bare molecular ground/excited density difference (green). In this analysis, one can investigate the distribution of charge between the ground and excited states inside and outside of the cavity for effects on the electrical current in materials and for reactivity in the excited state and will have direct application to the design of photovoltaic technologies.^122^

#### 3.3.3 Comparison between Self-Consistent and Parameterized QED Methods

Now that we have seen the types of studies that have been performed mainly using the scQED procedure, we will circle back to an explicit comparison between the pQED and scQED methods and showcase some results obtained on similar systems as already described. Further, the use of either pQED and scQED schemes should give the same result in the infinite basis limit. However, as we shall see in this section, each approach has its own strengths and limitations that need to be considered when applying to a specific calculation.

**Figure 10:**
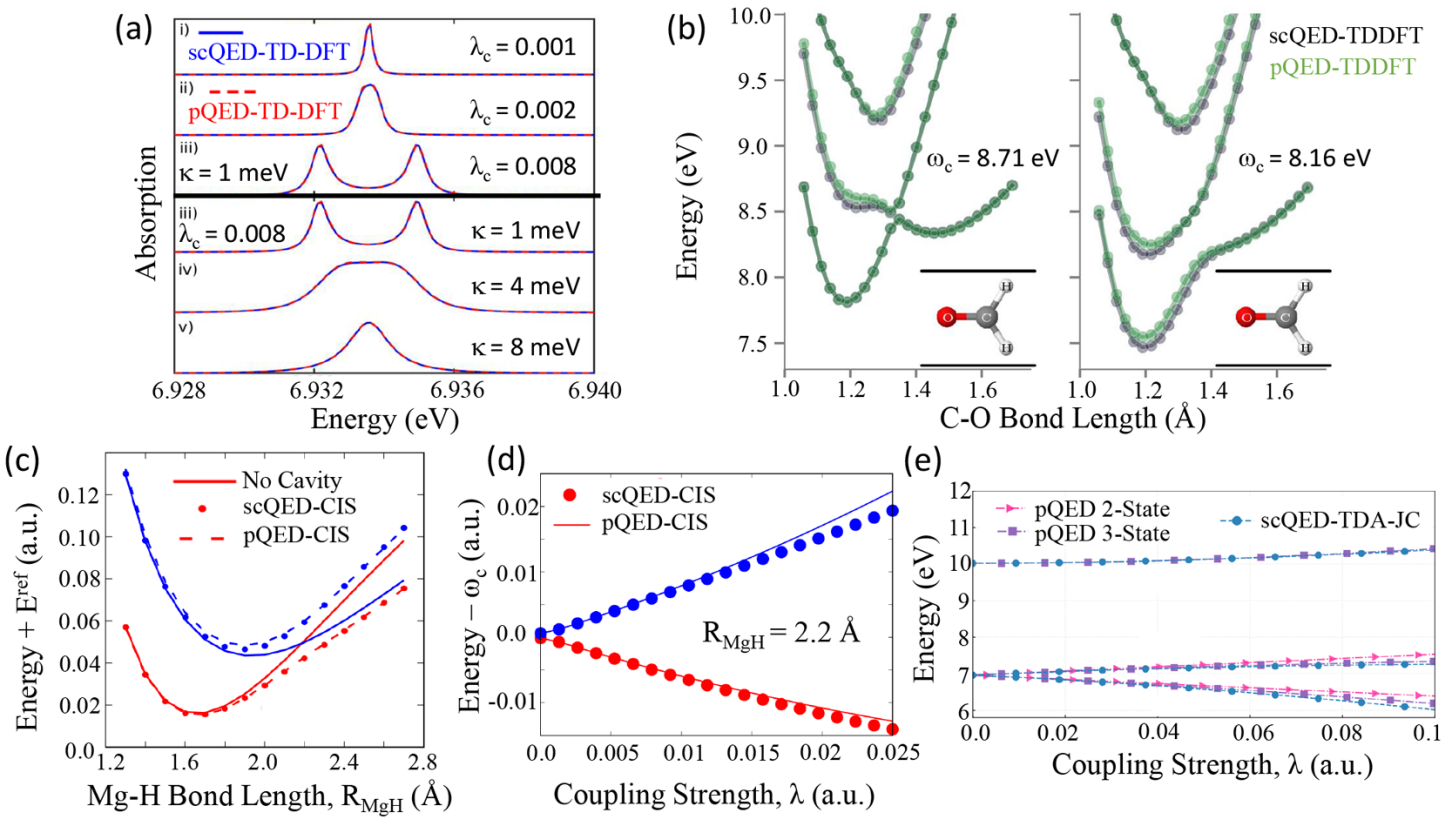
Comparison of parameterized QED Hamiltonians with self-consistent solutions. (a) Absorption spectra of benzene for a variety of weak coupling strengths, λ = (i) 0.001, (ii) 0.002, and (iii) 0.003 au, and for a variety of cavity loss rates, κ = 1, 4, and 8 meV. The cavity frequency is is resonance with the first bright transition of the bare benzene molecule. The pQED Hamiltonian is shown in red, while the scQED solution is shown in blue. (b) Born–Oppenheimer polaritonic potential energy surfaces for formaldehyde projected along the C–O bond stretch. The two cavity frequencies used were ω_c_ = (left) 8.71 and (right) 8.16 eV with coupling strength λ = 0.04 au The cavity polarization is along the C–O bond vector. The pQED result is shown in green, while the scQED result is shown in black. (c,d) The MgH^+^ molecule was placed into the cavity and the (c) Mg–H bond length *R*_MgH_ at fixed coupling strength λ = 0.0125 au and (d) coupling strength λ at fixed bond length *R*_MgH_ = 2.2 Å were scanned and compared between the pQED (dashed lines) and scQED (dotted lines) schemes. (e) The pQED scheme was tested on the ethylene molecular using a 2- (pink) and 3-state (purple) electronic basis and compared to the scQED-JC model scanning over coupling strength λ at the Franck–Condon geometry. Panel (a) is adapted with permission from ref (236). Copyright 2021 American Institute of Physics. Panel (b) is adapted with permission from ref (121). Copyright 2021 American Institute of Physics. Panels (c and d) are adapted with permission from ref (184). Copyright 2022 American Institute of Physics. Panel (e) is reproduced with permission from ref (115). Copyright 2021 American Institute of Physics.

In Figure 10a, the absorption spectra for the benzene molecule is shown in analogy to the toluene molecule discussed in Figure 8e, with cavity loss introduced in the same way (see discussion near eq 143). For these choices of coupling strength Figure 10a(i–iii) and cavity loss in Figure 10a(iii–v) parameters, the pQED-TD-DFT and scQED-TD-DFT methods provide nearly identical numerical results. It should be noted that this system is simpler than the toluene example since no nearby electronic excited states are present to mix with the character of the polaritons using these choices of parameters.^236^ Additionally, the coupling strength and cavity loss rates are very small in this example. Importantly, note that one would not have a priori knowledge on how many electronic or Fock states are required to obtain this pQED-TD-DFT Hamiltonian, and as mentioned before the number of of basis electronic and photonic states *should* be treated as convergence parameters.

Figure 10b showcases an investigation of the formaldehyde excited state PESs (as discussed previously in Figure 8b). This comparison leads to some deviations between the pQED-TD-DFT and scQED-TD-DFT schemes. Here, however, the pQED Hamiltonian was treated with a multistate generalization of Jaynes-Cummings model Hamiltonian (eq 83) while the scQED was treated with Pauli-Fierz QED Hamiltonian (eq 106). As such, the deviation might due to the use of different QED Hamiltonians.

Figure 10c presents the results of dissociation of the MgH^+^ molecule coupled to the cavity.^184^ The comparison between the scQED-CIS approach (at the level of configuration interaction singles) and pQED-CIS are performed, where a three-state Pauli-Fierz model was used including the |*g*,0⟩, |*g*,1⟩, and |*e*,0⟩ states for the pQED-CIS (where the energies, permanent dipoles, and single transition dipole were taken from bare CIS calculations). In other words, only one electronically excited state and one excited Fock state were used. This is the “minimum basis” for constructing the pQED Hamiltonian and one should enlarge the basis for achieving more accurate results at large light–matter coupling strengths. The agreement between the pQED-CIS and scQED-CIS are not perfect, and the deviations can be seen near the minima of the upper polariton (blue). Even with the minimal basis, the pQED Hamiltonian performs well for these parameters, and the deviation may be due to the simplicity of the excited state manifold (i.e., minimal basis) or rather the exclusion of dipole coupling and dipole self-energy contributions from higher-energy excitations (not included in the pQED simulation) for the choice of coupling strength used (see discussion regarding Figure 5). In Figure 10d, the bond length of the MgH^+^ molecule (coupled inside an cavity) was fixed while the light–matter coupling strength was increased. Here, at low coupling strengths λ < 0.01 au, the pQED-CIS Hamiltonian perfectly matches the scQED-CIS results. At large couplings λ > 0.01 au, the pQED-CIS deviates from the scQED-CIS results, indicating that the minimal basis of |*g*,0⟩, |*g*,1⟩, and |*e*,0⟩ is no longer good enough to converge the interaction and/or DSE terms that require additional electronic or photonic states.

In the final example of the comparison between the scQED and the pQED schemes, an explicit test using either two (pink triangles) or three (purple squares) electronic states in the pQED approach compared to the variational scQED approach (blue circles), both at the Jaynes-Cummings level, was performed on the ethylene molecule,^115^ as shown in Figure 10e. Here, both the pQED as well as the scQED schemes used the generalized Jaynes-Cummings Hamiltonian (see Section 3.2). At low light–matter coupling strength λ < 0.05 au, the two- and three-electronic-state pQED-TD-DFT and the scQED-TD-DFT result in the same energies. At larger light–matter coupling strength λ > 0.05 au, none of the three methods are in agreement, indicating that more basis states are required to converge the pQED results. In this work, only the number of electronic states was explored, with only a single excited Fock state included in the basis. In this case, only the |0⟩ and |1⟩ states were used. In the Supporting Information of this work,^115^ the number of electronic states was further tested for large values of coupling. Here, the authors used up to 1000 electronic states, and neither the energies or dipoles were fully converged at this size of basis. There are a couple potential causes for this deviation.^115^ First, at this size of electronic basis, the number of included Fock states becomes *extremely* important for the convergence. For example, the |*e*_*j*_, *n*⟩ basis state could be very close in energy to some nearby |*e*_*k*_, *n* ± 1⟩ state, and recalling the block structure of eq 112, the interaction are then be nonzero if the transition dipole between electronic states |*e*_*j*_⟩ and |*e*_*k*_⟩ is nonzero, which is undeniably hard to predict for an arbitrary system (see examples in Figure 5). Additionally, the effects of the DSE terms that connect electronic basis states of the same photon number via the square of the dipole matrix (see eq 112) can mediate an interaction between a high-energy electronic state with a low-lying state, making the convergence of the number of electronic and Fock states of supreme importance. Careful convergence of the electronic *and* Fock states must be done carefully and simultaneously.

In principle, both pQED and scQED will generate identical results under the complete basis limit, which is rigorously discussed in ref (53) for the Coulomb-gauge Hamiltonian. Compared to scQED, the pQED scheme is much simpler in the sense that it does not require additional redevelopment of electronic structure theory for the QED Hamiltonian as well as the simplicity that comes with a nonself-consistent solution. With the above available examples, one can see that if the light matter coupling strength is high and more electronic states are needed for a fully converged pQED calculation, then in principle, one needs to fully converge these excited electronic states first before doing the pQED simulation. Further, as one increases the number of electronic basis states, one also needs to balance the convergence with the number of photonic states, which can be complicated and nonmonotonically convergent with respect to various polaritonic properties one aims to compute, such as the density, eigenenergies, or mode occupation.^53^

One important consideration, of many, is the fulfillment of the TRK sum rule in eq 68, which is a fundamental requirement by exact quantum mechanics. However, due to the use of approximate electronic structure methods, the TRK sum rule becomes method-dependent. For example, TD-DFT satisfies this rule but TD-DFT in the Tamm-Dancoff approximation (TDA) does not (see Section 3.2.3).^251^ Thus, the accuracy of the excited state dipole matrix elements (such as those shown in Figure 5) might violate this rule for some electronic structure methods and become less accurate for high-lying excited states, which are a necessary input into the pQED method at large coupling strengths and will eventually lead to a less accurate description of polariton states.

On the other hand, one can start with a reasonably sized basis, construct excited configurations from a reference trial electronic-photonic wave function, and solve only the first few polaritonic states, as needed, directly by using scQED approach with high accuracy combined wither iterative diagonalization techniques.^186−188^

Here, it is important to note that the scQED schemes may require substantially less computational effort than the analogous pQED scheme. For the ground polaritonic state in particular, the pQED scheme requires, in principle, knowledge of all of the many-particle excited states in order to converge the ground polaritonic state, while the scQED scheme is roughly the same cost as a standard ground state variational scheme. The excited states calculation in both schemes are more similar in computational expense, since both require an excited state method (e.g., TD-DFT, EOM-CC, etc.). The pQED scheme may require the calculation of many more (∼10–20) bare electronic excitations to converge the few lowest-energy polaritonic states; whereas, the scQED scheme only requires the calculation up to the number of polaritonic states needed. Note that even in the excited state, the convergence in the number of included virtual orbitals in the scQED scheme is still required, especially at large light–matter coupling strengths. Further, the gauge invariance of the problem is always satisfied when using the properly truncated dipole-gauge Hamiltonian (eq 70) for either pQED or scQED schemes. As such, we believe that the self-consistent evaluation of the Pauli-Fierz Hamiltonian will be a much more general and reliable scheme to produce converging results toward chemical accuracy, especially at very strong light–matter coupling strengths. However, the pQED scheme (in our opinion) is a very useful tool aimed at the convenient calculation of polaritonic properties for application-style studies, where only semiquantitative trends may be important.

Additionally, it is important to make a distinction between gauge invariance and numerical convergence. In principle, the pQED framework using only two electronic states (eq 70) is gauge invariant (see the discussion in Section 2.4).^75,80,90^ However, the results generated by such a calculation are likely incorrect since two electronic states is likely not enough to numerically converge the polaritonic states (due to the lacking included correlations) for most realistic molecules or model systems which have many electronic states. Similarly, scQED results are intrinsically gauge invariant (by using a Hamiltonian such as eq 70 in this work), regardless of the number of virtual orbitals considered. As such, the convergence may be more easily obtained in the scQED schemes by the inclusion of more virtual single-particle orbitals while the pQED scheme requires the inclusion of additional many-particle excitations which may be more costly to include.

Despite the enormous recent progress in both scQED and pQED schemes, what is generally missing is a *consistent and fair comparison* of both approaches^53^ and assessment of the strengths/limitations of each method under different realistic, ab initio scenarios. Almost all results generated by scQED approaches have benchmarked against a some form of pQED approach.^53,115,121,184,236^ However, in most of these comparisons, a minimal basis (that usually only includes one excited photon state and one or two excited electronic states) are used in the pQED comparison (as largely discussed in Sec. 3.3.3), with the exception of a few works.^53,115^ A *consistent and fair comparison* is still needed to provide a convergence test for the pQED scheme (if possible for moderate-to-large light–matter coupling strengths) as well as for the scQED results.

#### 3.3.4 Modification of the Polaritonic Ground States

We now move to another recent direction, where the *ground state* of a molecular system can be significantly modified by coupling to a cavity photon mode with a photon frequency beyond the infrared (IR).^73,122,124,182,195,203,208,235^ From the technical perspective of electronic structure theory, this appears to be a simpler problem because ground states (even for polaritons) are often easier to obtain compared to excited states. Meanwhile, an intuitive understanding of cavity modification of the molecular ground state is not available when using simple quantum optics models. In fact, the JC model predicts that the ground state is simply |*g*,0⟩ irrespective of the cavity coupling strength λ or the cavity photon frequency ω_c_ and is therefore completely decoupled from the manifold of excited adiabatic-Fock basis states. Of course, the JC model Hamiltonian is known to explicitly break down, especially for large coupling strengths (see Figures 3 and 6). Therefore, the investigations focused on the ground state properties of a polaritonic system necessarily belong to a regime beyond the JC model. This failure of the JC model also indicates that the cavity modification to the molecular ground state operates in the ultrastrong coupling regime (USC) or beyond. Further, this is also an interesting direction where new chemical reactivity could occur in the ground state of the hybrid system, which is not well-understood and may require one to “re-learn” molecular orbital theory in the presence of the cavity.^124^

In this case, there is no semblance of the Rabi splitting (eq 6), since the electronic state in question (i.e., the ground state) is far away in energy from the cavity frequency (e.g., ∼1–10 eV) as we will see in the following examples. However, the dipole self-energy, which couples electronic states through dipole interactions mediated by the cavity, will still have a drastic effect, especially at large coupling strengths. To be clear, the cavity frequency in these examples is far away from those of the vibrational strong coupling (VSC) cases, which have cavity frequencies on the order of ∼0.1 eV (see Section 5 for more details of VSC) lying in the IR regime. Additionally, the difficulties of such calculations are significantly simplified because they only require a ground state electronic-photonic structure method for the scQED scheme (e.g., QED-HF, QED-DFT, QED-CC, etc., see Sec. 3.2) and are therefore computationally simpler than those previously discussed simulations in this section that required the explicit calculation of excited polaritonic states. In contrast, performing pQED calculations require the calculation of excited states, since these ground state modification reviewed in this section are induced by off-resonance couplings between the molecular ground state and other electronic states (through DSE) or other light–matter dressed states (through light–matter coupling term). Of course, the pQED Hamiltonian will provide the same results as the scQED approaches in the infinite basis limit.

**Figure 11:**
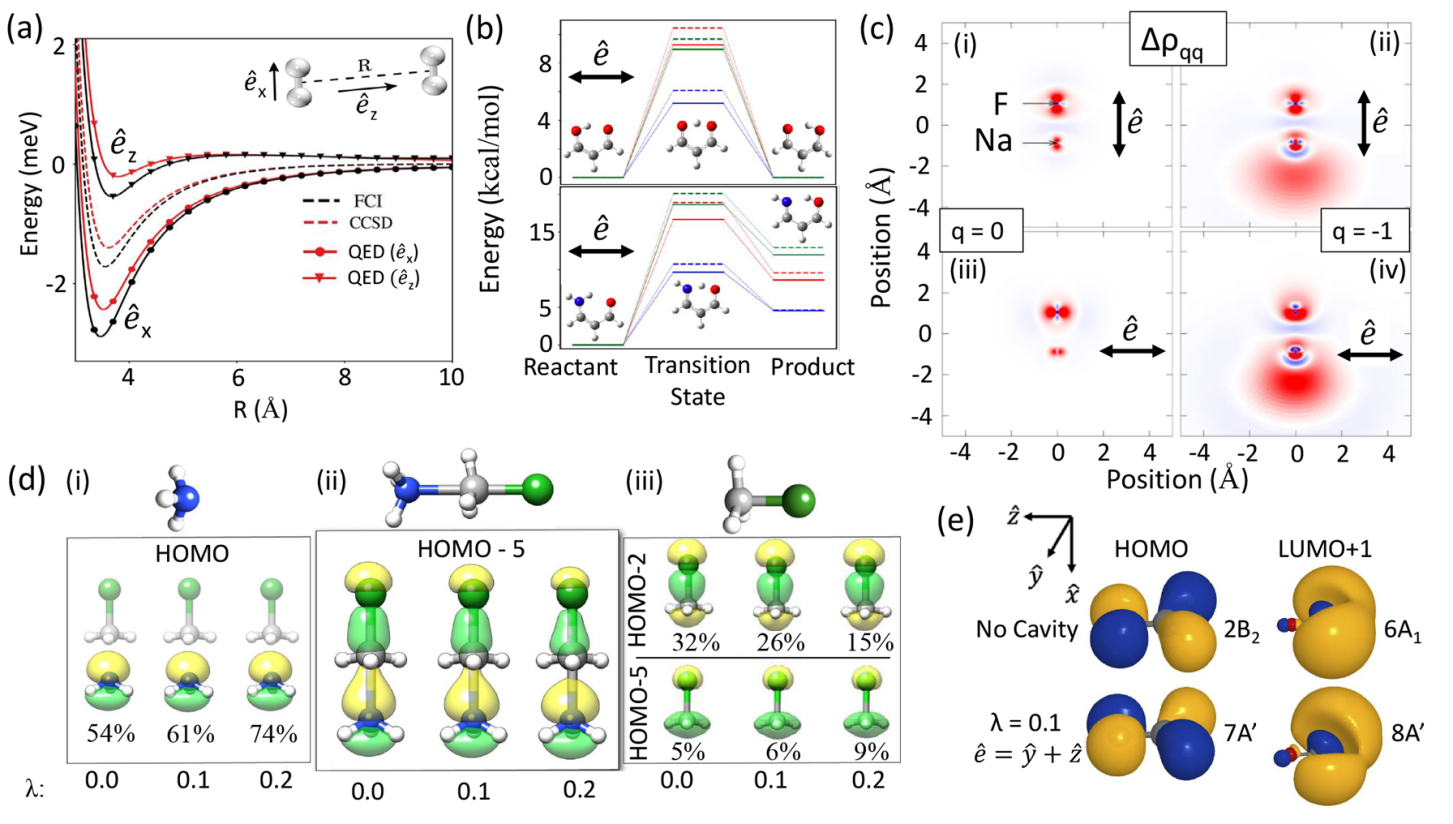
Ab initio electronic-photonic structure for the polaritonic ground state. (a) Polariton-induced modifications to noncovalent ground-state (i.e., van der Waals) interactions between a pair of H_2_ molecules is shown, including interaction with either an X- (circle) or Z- (triangle) polarized cavity at either the QED-CCSD-1–21 (red) or QED-FCI (black) levels of theory. The single-mode cavity frequency is ω_c_ = 12.7 eV with coupling strength λ = 0.1, and dotted lines showcase the out-of-cavity results. (b) The reaction barrier of the proton-transfer in malonaldehyde is modulated through interactions with the cavity (X-polarization) at the QED-CCSD-1–21 (blue), QED-DFT (green) and QED-HF (red), where the solid lines correspond to outside the cavity. The cavity frequency is ω_c_ = 3 eV with coupling strength λ = 0.1. (**c**) Ground state density differences Δ*ρ*_*qq*_ = *ρ*_*q*_ – *ρ*_*q*_ of (i, iii) neutral NaF and (ii, iv) anionic (NaF)^−^ with the cavity polarization *ê* (i, ii) parallel and (iii, iv) perpendicular to the Na–F bond (black arrows). Here *ρ*_*q*_ indicates the polaritonic ground state total density with *q* ∈ {0, – 1} total charge. The colormap indicates that red (blue) is increased (decreased) electron density. (d) Molecular orbitals of the methyl chloride CH_3_Cl and ammonia NH_3_ molecules (center, at transition state of the S_N_2 reaction) and its two main components at varying light–matter coupling strengths λ = 0.0, 0.1, and 0.2 au The molecular orbitals are chosen as they comprise the majority of combined. The percent contribution is shown for each choice of light–matter coupling. system based on the amount of contribution to the combined system. (**e**) The HOMO and LUMO+1 molecular orbitals are shown for the formaldehyde molecule (bottom) inside and (top) outside of cavity. The light–matter coupling is set to λ = 0.1 au The symmetry of the electron orbitals are shown to the right side of each orbital. Panel (a) is adapted with permission from ref (123). Copyright 2021 American Institute of Physics. Panel (b) is reproduced with permission from ref (125). Copyright 2022 American Chemical Society. Panel (c) is adapted with permission from ref (195). Copyright 2021 American Institute of Physics. Panel (d) is adapted from ref (124). Panel (e) is adapted with permission from ref (184). Copyright 2022 American Institute of Physics.

Figure 11 presents several recent works on modifying the ground state properties when coupling molecules to a high frequency cavity (in the electronic excitation range). Figure 11a examines the effects of modulating intermolecular interactions by coupling an *H*_2_ dimer to a cavity with frequency ω_c_ = 12.7 eV and coupling strength λ = 0.10 au^123^ Using the coupled cluster (CC) and full configuration interaction (FCI) methods, as well as their scQED variants, QED-CC and QED-FCI,^122,123^ it was determined that the presence of the cavity drastically modulates the intermolecular interactions between the two *H*_2_ molecules. Depending on the cavity polarization direction with respect to the hydrogen dimers, the intermolecular potential well can be increased by ∼0.75 meV (for *ê*_*z*_ polarization along the intermolecular axis) or decreased by ∼1.0 meV (for *ê*_*x*_ polarization along the intramolecular bond axis), respectively. These weak intermolecular interactions, on the order of meV, are ubiquitous in chemistry. As such, a drastic change in the intermolecular potential may give new and interesting effects in many of these processes. In this simple example, the well was modified by up to 100% compared to outside of the cavity.

Along the same vein, Figure 11b also uses the scQED-based approach to investigate the ground state proton transfer reaction in the symmetric malonaldehyde (top) and asymmetric aminopropenal (bottom) molecules.^125^ Here, the cavity frequency was set to 3.0 eV with light–matter coupling λ = 0.10 au The ground state energy at the transition and product (for the asymmetric molecule) geometries, both of which are relative to the reactant energy, were computed using a variety of scQED methods, including QED-CC (blue), QED-HF (red), and QED-DFT (green). The reaction profile outside the cavity (solid lines) is also calculated using the corresponding level of the theory. Inside the cavity (dashed lines), for both molecules (top and bottom panels) and for all levels of theory, the reaction barrier height is increased by nearly ∼1 kcal/mol (top panel) and ∼0.85 kcal/mol (bottom panel) when coupling molecule with the cavity. For the aminopropenal molecule (bottom panel) the product was only changed by ∼0.1 kcal/mol for the QED-CC method and ∼1.0 kcal/mol for QED-DFT and QED-HF. This evidence the fact that coupling between the cavity and the excited electronic states may have drastic consequences for the ground-state potential energy landscape for *large* coupling strengths. This is in contrast to the case of vibrational strong coupling between light and matter, where the classical potential barrier on the ground polaritonic potential energy surface is not changed.^85^ More discussions related to VSC can be found in Section 5.2.

In another work,^195^ the authors use the scQED-HF and the scQED-CCSD-1-21 approaches (see Section 3.2) to explore the effects of adding (i.e., electron affinity) or removing (i.e., ionization potential) from sodium halide molecules coupled to the cavity^195^ Specifically, Figure 11c shows the ground state electronic density difference, defined as Δ*ρ*_*qq*_ = ρ_*q*_^pl^ -ρ_*q*_^el^ where *q* is the total charge in the system, ρ_*q*_^pl^ is the ground state density inside the cavity, and ρ_*q*_^el^ is the ground state density of the bare molecular system. As shown in Figure 11c, the bond of the NaF molecule can be destabilized upon insertion into the cavity and further destabilized if the molecular system is negatively charged. The results of the ground state difference density function evaluated in the plane of the molecule are shown in Figure 11c with the coupling strength λ = 0.05 au and with the cavity polarization along the Na–F bond vector (panels i and (ii) and perpendicular to the bond vector (panels iii and (iv). Further, panels (i) and (iii) are for a neutral system, and panels (ii) and (iv) are for a negatively charged system. The red color indicates an increase in the electron density upon coupling to the cavity, whereas the blue color indicates a decrease in electron density. For the neutral systems, when setting the cavity polarization along the bond (panel i) or perpendicular to the bond (panel iii), it was found that there is a relatively small change of the electron density, except at positions very close to the nuclei. In both cases, the electronic density differences showcase a p-orbital-like increase in density with the same polarization as the cavity. In panel (i), there is a small reduction in electron density (blue color) between the Na and F nuclei, indicating a **reduction in bonding character**. It is clear from the electronic redistribution in both polarization directions (i, iii) that the stability and bonding character of the NaF system is significantly modified, which will lead to changes in the ground state dissociation of these molecules.^195^

Figure 11c (ii) and (iv) present the same type of analysis but for a negatively charged species (NaF)^−^. Here, the electronic redistribution is more drastic, and at larger distances from the atomic centers, there is a less noticeable impact from the polarization direction. In both cases of the cavity polarizations, a large addition of electron density can be seen below the Na atomic center, more prominent in the case of perpendicular polarization (iv). This indicates that the cavity is able to significantly redistribute this additional electron *from* three places: (I) close to the nuclei, (II) between the Na–F bond, and (III) far-away (light blue >2 Å from Na nucleus). In all four cases, the bonding character is expected to be reduced while a major electron density reorganization is seen for the negatively charged molecule. This work demonstrated the capacity of the scQED-CC method for investigating the electron affinity and ionization potentials of various small systems and provided simple physical explanations of the cavity-induced effects through the ground state density difference function.

Further, the authors^195^ also explain effects of the cavity coupling on the ground state of molecule, prior to perform ab initio polaritonic calculations. In this scQED framework, the authors used the coherent state basis^183,252−254^ (defined earlier in Section 3.2) which allows one to observe the size of the variance in the dipole with respect to the electronic ground state which is (Δμ̂)^2^ (see discussion around eq 124). The size of the variance will give direct insight into the magnitude of the cavity effects on the ground state and can be calculated for the bare molecular system outside the cavity. This was computed for the sodium-halide species in the present example, which predicted the larger effects for the anionic (negatively charged) species that was later observed to be accurate after performing the explicit scQED procedure.^184,195^

In ref (124), the reactivity of a generic S_N_2 reaction between methyl chloride CH_3_Cl and ammonia NH_3_ (which forms methylamine and hydrogen chloride) was explored from the viewpoint of molecular orbital (MO) theory. Here, the authors^124^ portray a new ideology of MO theory inside the cavity, referred to as cavity MO theory. Using the self-consistently updated ground state MOs from a scQED-HF scheme, the authors make predictions regarding the thermodynamic driving force of the reaction based on the strongly participating MOs between reactive substituents. Figure 11d (ii) presents the main results of the work, where the transition state geometry of the reaction is shown along with the dominant MO, HOMO-5, with strongly overlapping orbitals between all participating species for coupling strengths λ = 0.0, 0.1, and 0.2 au (left to right within each panel).

Figure 11d (i, iii) show the projections onto the substituents’ MOs that largely contribute to the bonding process at each light–matter coupling strength. Notably, for the full molecule shown in (ii), the bonding of the nitrogen to the carbon gradually decreases with increasing coupling strength λ, effectively due to the localization of the nitrogen’s lone pair to the nitrogen atom. Here, the presence of the cavity influences the relative contributions of the substituent MOs as shown for the (i) ammonia and (iii) methyl chloride. For the ammonia species, the contribution of the antibonding lone pair localized to the nitrogen is increased with increasing coupling strength λ. This is the main driving force for the reduction in the nitrogen–carbon bond at the transition state geometry found in Figure 11d (ii). The other effect found in (ii) is the weak conversion of the carbon-chloride bonding character to antibonding character with increasing coupling strength. This is exemplified in (iii) which showcases two main contributing projected orbitals, HOMO-2 and HOMO-5, of the methyl chloride subsystem. HOMO-2 possess the bonding character between the chloride while HOMO-5 provides the antibonding character. As the coupling strength increases, the bonding orbital contribution decreases from 32% to 15% while the antibonding orbital increases from 5% to 9%. This accounts for the reduction in bonding character found in (ii). This work^124^ exemplifies that molecular orbital theory still applies but needs to be further understood in the presence of a cavity. Through self-consistent electronic-photonic structure theory (i.e., scQED ground state methods), one can more easily understand the response of the MOs due to the presence of the cavity. Performing a similar calculation via the pQED scheme, on the other hand, is not trivial for the analysis of the ground state MOs. In principle, it should be possible to reformulate eq 112 in the basis of MOs rather than electronically correlated excited Slater determinant states, carefully accounting for the occupation numbers of photon-dressed MOs.

A similar work performed an analysis of the ground state of formaldehyde with scQED-HF.^184^Figure 11e shows the (left) HOMO and (right) LUMO+1 for the molecule coupled inside the cavity (bottom) and for the bare molecule outside the cavity (top). The cavity frequency was set to ω_c_ = 10.4 eV with coupling strength λ = 0.1 a.u and polarization *ê* = *ŷ* + *ẑ* (see Figure 11e for Cartesian axes). Here, the authors make note of the *loss* in the symmetry of the MOs resulting from the influence of the cavity. The overall symmetry of the molecule changed from *C*_2*v*_ to *C*_s_ after orbital relaxations under influence from the cavity. The bare molecular system contains a HOMO with 2B_2_ symmetry and LUMO+1 with 6A_1_ symmetry. Upon coupling to the cavity, the MOs become distorted (similar to what was seen in the previous example Figure 11d) and take on new types of symmetry with labels 7A′ and 8A′, respectively. The LUMO+1 state has the most visually obvious effects in that the p-orbital on the oxygen (left-most atom) rotates to becomes parallel with the polarization direction, while the other part of the orbital changes shape entirely with the dominating part of the orbital lying in-line with the oxygen p-orbital rather than symmetrically split according to the symmetry of the nuclei. These modifications to the frontier orbitals showcase the drastic effects the cavity may have on local reactivity of the molecules whereby the molecular orbitals exchange character and lead to various changes to the local electrostatic potential and atomic charges. In the same work, the configuration interaction (CI) theory is also developed for the sc-QED method, which is convenient for incorporating electronic-photonic correlations for calculations of the excited states.

In conclusion of this section, examining the response of the ground and excited electronic structure to the presence of molecule-cavity coupling is of extreme importance for all theoretical applications. The significant changes to the properties can elucidate a new and powerful method for manipulating chemical reactions in the ground state and tuning the local excitonic character of excited states to use in photochemistry and optoelectronic property modification. Further, the use of either pQED and scQED schemes is expected give the same result in the infinite basis limit; however, each scheme will have strengths and limitations that need to be considered when applying to a specific situation. When connecting theoretical simulations with experimental results with many molecules coupled to many cavity modes inside a Fabry–Pérot cavity, one needs to consider the generalized Pauli-Fierz Hamiltonian *Ĥ*_PF_^[*N*]^ in eq 101. The development of ab initio polaritonic methods for the GTC Hamiltonian (eq 102) has been achieved, with the pQED-type of method to solve the polariton state, and mixed quantum-classical dynamics approaches (see Section 4.1.4–4.1.5) to investigate polariton dynamics of many molecules coupled to many cavity modes.^138,177^ Future directions for the theoretical community may involve the application of such a Hamiltonian toward the simulation of a many-mode , many-molecule  cavity system through both pQED and scQED type of methods. Here, the scaling due to the molecules and photons will be of . However, including the self-consistency of the many-electron problem, the scaling will also include a factor  with *b* dependent on the choice of electronic structure method. These simulations are required to explore the true collective nature of the polaritonic system for a variety of realistic chemical reactions, polaritonic propagation, and energy/particle transfer processes.

In the following section, we move to a photophysical discussion on how polaritonic dynamics in the excited state can be performed with models as well as with ab initio information in order to demonstrate specific examples of modified excited state processes achievable in both experimental and theoretical realizations.

## 4 Polariton Photochemistry and Photodynamics

The emerging field of polariton photochemistry has seen tremendous growth over the past decade due to numerous experimental^3,27,38,39,255^ and theoretical advancements.^5,10,13,70,117,256−258^ The theoretical and computational investigations of polaritonic photochemistry thus far underpin the great potential for using cavities to control photochemical reactivity. This section aims to highlight these advancements and offer insight into the various mechanisms that light–matter coupling provides for modifying photochemistry.

There are two overall regimes of light–matter coupling which offer different mechanisms for changing chemical reactivity: the weak coupling and the strong coupling regimes. The primary characteristic that differentiates these two regimes is whether the light–matter coupling strength *g*_c_ is smaller than (weak coupling) or larger than (strong coupling) the various loss rates of the system.^31−34^ In the weak coupling regime, the primary mechanism for modifying chemistry is through an enhancement of the overall loss rate of the system, known as the Purcell effect.^33,259,260^ In this regime, there is a limited modification of the potential energy surfaces which limits the amount of control one has over modifying chemical reactions. On the other hand, in the strong coupling regime, significant changes to the potential energy surfaces can be observed and are adjustable based on fundamental physical characteristics such as the cavity frequency ω_c_ and the light–matter coupling strength *g*_c_. These potential energy surface modifications, along with other factors such as the initial photonic state, the rate of cavity loss, and the presence of the dark state manifold, offer several mechanisms for theorists and experimentalists to use to control chemical reactivity in the strong coupling regime.

**Figure 12:**
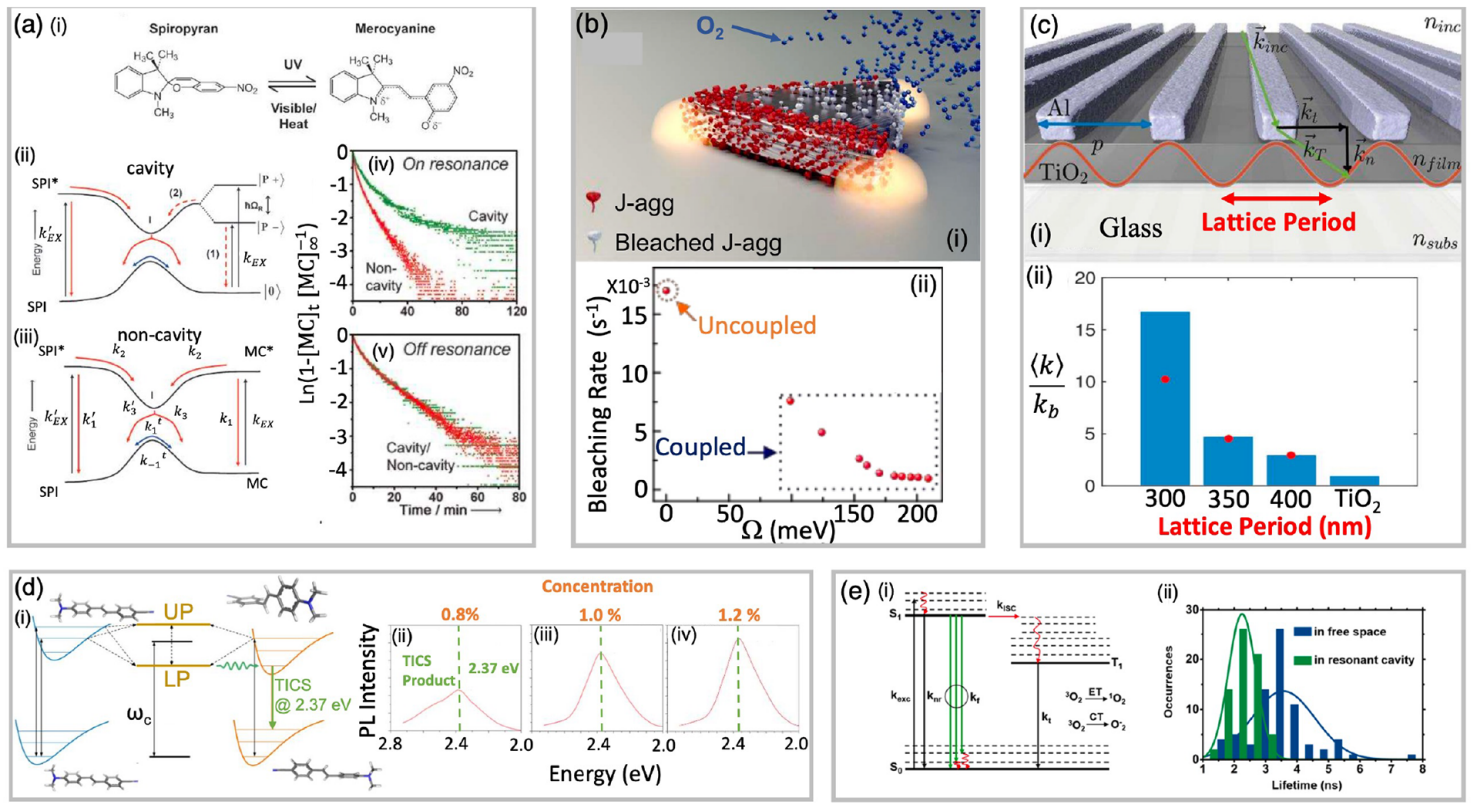
Recent polariton photochemistry experiments. (a) (i) Schematic of the ring-opening reaction of spiropyran (left) to merocyanine (right) under ultraviolet (UV) irradiation while the reverse reaction occurs under visible (VIS) irradiation along with thermal energy. (ii, iii) Schematic of the potential energy surfaces (ii) inside and (iii) outside the cavity with red arrows showcasing the possible reaction/emission pathways and black arrows indicating possible electronic or polaritonic excitations. (ii) The dashed red arrows exemplify the modified pathways due to the cavity. (iv, v) The time-dependent concentration of the merocyanine (MC) product (plotted as ln(1–[MC]_*t*_[MC]_∞_^–1^)) inside (green) and outside (red) for a cavity that is (iv) resonant and (v) off-resonant with the MC electronic excitation (in the UV). (b) (i) Schematic of J-aggregates of TDBC dye molecules coupled to a plasmonic nanoantennae cavity. The dye molecules can undergo photobleaching which involves reactions with atmospheric oxygen (blue) and creation of reactive oxygen species. (ii) The photobleaching rate of the dye molecules outside the cavity (uncoupled) and inside the cavity (coupled) for different Rabi splittings. (c) (i) Schematic of a plasmonic cavity which contains a periodic repetition (i.e., a lattice) of aluminum (Al) strips on a TiO_2_ film on a glass substrate. The decomposition rate of the molecule methyl orange is examined outside and inside the plasmonic Al lattice. (ii) The lattice period of the Al strips is varied and a ratio of the rate inside the cavity ⟨*k*⟩ to the *bare* “*b*” rate outside the cavity *k*_*b*_ is obtained. (d) (i) Potential energy diagram of an excited state energy transfer pathway of a E-4-dimethylamino-4′cyanostilbene (DCS) molecule from its planar excited state (PES) to another DCS molecule in its twisted ICT excited state (TICS) whereby the photonic state of the cavity (center black line) mediates transitions between various vibronic states (thin horizontal lines) of the electronically excited isomer states (left and right, thick curved lines) via the formation of upper (UP) and lower (LP) polaritonic states (center, dark orange). (ii–iv) Photoluminescence intensity (PL) as a function of emission energy for various concentrations of DCS: (ii) 0.8%, (iii) 1.0%, and (iv) 1.2%. The vertical dashed green lines indicate the energy of the TICS species at 2.37 eV. (e) (i) Energy diagram of the tautomerization reaction of a single phthalocyanine molecule showing various reaction pathways including nonradiative singlet decay (*k*_nr_), singlet fluorescence (*k*_f_), singlet to triplet intersystem crossing (*k*_ISC_), and triplet decay (*k*_t_). The singlet fluorescent decay rates are increased inside the cavity. (ii) Single molecule fluorescence lifetime distributions outside the cavity (blue) and inside the cavity (green). Panel (a) is adapted from ref (3). with permission. Copyright 2012 WILEY-VCH Verlag GmbH and Co. KGaA, Weinheim. Panel (b) is adapted from ref (38) under the CC BY-NC license. Panel (c) is adapted from ref (255) with permission. Copyright 2015 WILEY-VCH Verlag GmbH and Co. KGaA, Weinheim. Panel (d) is adapted from ref (39) under the CC BY-NC license. Panel (e) is adapted from ref (136) with permission. Copyright 2021 American Chemical Society.

Recent experiments in polariton photochemistry have demonstrated some promising results of using molecule-cavity coupling to change photochemical reactivity. One of the first experiments to demonstrate a change of photochemical reactivity in the strong coupling regime is shown in Figure 12a, adapted from ref (3). In this work, the rates of a photoisomerization reaction (panel i) between spiropyran and mecrocyanine via a photoexcited ring cleavage) were modified by resonant coupling between the molecules to a Fabry–Pérot cavity, with a reported Rabi splitting of Ω_R_ = 700 meV. In Figure 12a-(ii), the proposed mechanism of this modification^3^ was an increase in the decay rate of the pathway (1) (radiative relaxation from the lower polariton state) relative to the pathway (2) (excited state isomerization) caused by the formation of the lower polariton. This mechanism led to the slowdown of the isomerization reaction inside the cavity at resonance (Figure 12a-(iv)) but was not present in the off-resonant case (Figure 12a-(v)).

Another experiment shown in Figure 12b, adapted from ref (38), demonstrates a suppression of photobleaching rate of J-aggregates of TDBC dye molecules with dependence on the Rabi splitting as shown in panel b(ii). This work utilized plasmonic nanoantennae (panel i) to produce a strong cavity field that couples to the dye molecules and generates a large Rabi splitting between the polariton states. These polariton states are able to decay the excited population to the ground state more quickly before the excited population can transfer to the triplet state and undergo photobleaching, which reduces the photobleaching rate inside the cavity (panel ii) thus increasing the stability of the dye inside the cavity. This effect of this mechanism is enhanced for larger Rabi splittings.

In Figure 12c, a plasmonic array cavity (panel i) was used to modify the photochemistry of the photocatalytic decomposition of methyl orange from ref (255). The methyl orange can become reactive when the adjacent TiO_2_ undergoes UV irradiation and ionizes the methyl orange, which can then react with other radicals in solution and break down. The coupling of this pathway to the plasmonic array allows for the formation of waveguide-plasmon polaritons which can increase visible light absorption and decrease radiative damping,^255^ which can alter the reaction rate. In order to control the decomposition reaction using a cavity, the array nature of the cavity (in panel c(i)) allows the lattice period, and thus the cavity frequency, to be adjusted. This ability to selectively modify the lattice period was used in panel c(ii) to demonstrate the frequency-dependent modification of a photocatalytic decomposition reaction rate.^38^ In addition to adjusting the cavity frequency and coupling strength, the pump excitation frequency can also be adjusted to selectively control excited state energy transfer as demonstrated by the work shown in Figure 12d, adapted from ref (39). When the cavity-coupled system (i) was pumped at the frequency of the lower polariton instead of at the bare reactant frequency, the photoluminescence spectra was dominated entirely by the twisted ICT (TICS) excited state (ii–iv) instead of a mixture of planar (PES) and twisted (TICS) isomer signals. This selectivity of excited state population based on pumping frequency was further enhanced by increasing the molecule concentration and thus the light–matter coupling strength (ii–iv). Photochemistry has also been shown to be modifiable in the weak coupling regime in the work of Figure 12e, adapted from ref (136). The decrease in excitation lifetime (ii) due to the Purcell effect caused a reduction of population transfer from the singlet to triplet excited state (i) which ultimately reduced the rate of excited state tautomerization.

While the aforementioned photochemical experiments have demonstrated some promise for cavity-controlled photochemistry, recent theoretical investigations on the topic have shown a wider array of ways to control photochemistry with polaritons and have elucidated the possible mechanisms behind this control. The following section details some of these theoretical works in polariton photochemistry. Section 4.1 outlines various methods for performing nonadiabatic polariton photochemical simulations. Section 4.2 describes how light–matter hybridization allows for control over photochemical processes. Section 4.3 overviews the results of realistic ab initio simulations of cavity-coupled photoisomerization reactions. Section 4.4 outlines the various ways that light–matter coupling can control charge transfer reactions. Section 4.5 details the influence of cavity-induced conical intersections on photochemical reactions. Section 4.6 introduces how the initial state of the photonic mode can be manipulated to influence photochemical dynamics. Lastly, Section 4.7 goes over the impact of cavity loss on photochemical reactivity.

We also recommend the recent review articles in polariton photochemistry: refs (2) and (37) provide the general ideas of using polariton as a new platform for controlling chemistry; refs (10), (31), and (21) provide general discussions on the potential surface hybridization due to molecule-cavity interactions; and ref (261) summarizes the theoretical challenges for simulating polariton quantum dynamics in a molecule-cavity hybrid system.

### 4.1 Nonadiabatic Polariton Photochemical Simulations

Here, we provide a short discussion of dynamical simulations of polaritons chemistry. The essential task is trying to solve the time-dependent Schrödinger equation (TDSE)
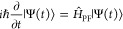
148where |Ψ(*t*)⟩ is the total quantum states of the electronic-nuclear-photonic quantum state of the molecule-cavity hybrid systems, whose time-evolution is governed by the QED Hamiltonian *Ĥ*_PF_ (eq 56). For more than a few nuclear DOF, solving the TDSE exactly is prohibitively expensive. Depending on the complexity of the molecular system, one may perform the dynamics exactly as dictated by the TDSE or resort to various approximations, such as mixed quantum-classical (MQC) approaches, semiclassical approaches, various approximate master equation approaches (e.g., Lindblad, Redfield, etc.) and approximate wave function approaches.

In the following discussion, we will brief introduce two popular mixed quantum-classical approaches as well as an exact method for solving polariton quantum dynamics.

#### 4.1.1 Exact Polaritonic Quantum Dynamics

We begin by briefly discussing how to solve eq 148 exactly, thus giving an exact solution to the polaritonic quantum dynamics. There are, in principle, many possible strategies for exact quantum dynamics propagation, and we only outline one of the most commonly used strategies based on the Born-Huang expansion.

We describe the total wave function of the electron-photon-nuclear DOFs using the Born-Huang expansion^262^ using the polaritonic basis as,
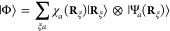
149where *χ*_*αn*_(**R**_ξ_) = ⟨**R**_ξ_|⊗⟨Ψ_*a*_(**R**_ξ_)|Φ⟩. Here {|Ψ_*a*_(**R**)⟩} are the polaritonic state at **R** which can be written in expressed in the adiabatic-Fock state representation as |Ψ_*a*_(**R**)⟩ = ∑_*α,n*_C_*α,n*_^*a*^|*ψ*_*α*_(**R**_ξ_)⟩,*n*⟩ and are obtained by diagonalizing (see eq 111). Within this representation, the total light–matter Hamiltonian is written as
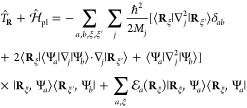
150where we have used the simplified notation |Ψ_*a*_⟩ ≡ |Ψ_*a*_(**R**_ξ_)⟩. We refer the reader to refs (263) and (264) for evaluating the nuclear kinetic energy (first term) and the derivative coupling term (second term) using spectral functions or the DVR basis.

Upon diagonalization of this Hamiltonian , the electronic-nuclear-photonic eigenstates can be obtained as,
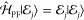
151

The electronic-nuclear-photonic wave function is then evolved simply as
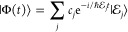
152where _*j*_ is the *j*_th_ eigenvalue and *c*_*j*_ is the projection of initial total wave function onto the *j*_th_ eigenstate |_*j*_⟩
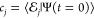
153where |Ψ(*t* = 0)⟩ is the initial condition and can be arbitrarily defined in each case. Additional details on the exact propagation can be found in refs (118, 265, and 107).

Here, we have depicted only one possible way of performing exact polaritonic dynamics; however, many other exact (or almost exact) quantum dynamics approaches exist that can be utilized. In the following, we will mention a few of these approaches: The multiconfiguration time-dependent Hartree (MCTDH) scheme has recently been used to simulate polariton photochemistry,^266^ conical intersections in cavity,^267,267^ and vibrational polariton dynamics.^190,268,269^ The exact factorization (XF) approach has only recently been developed and has been to used to simulate polariton photochemistry giving rise to novel interpretations of the wave function and the *exact* potential energy surface depending on the choice of factorization of the electronic, photonic, and nuclear DOFs.^11,270^ The hierarchical equation of motion (HEOM) approach has been used to simulate conical intersection inside cavity^271^ and vibrational polariton chemistry.^272^ Additionally, ab initio multiple spawning (AIMS),^273,274^ Ehrenfest multiple cloning (EMC),^275^ and their variants^276−278^ could also be adapted for polaritonic dynamics to give nearly exact results.

#### 4.1.2 Ehrenfest Dynamics

Ehrenfest (EH) dynamics is a mixed quantum-classical (MQC) approach for propagating the coupled electron-photon-nuclear dynamics.^275,279,280^ Within this approach, the nuclear DOFs are evolved classically while the electronic and photonic DOFs are treated quantum mechanically. Below, we define the wave function for the quantum subsystem (which includes the electrons and the photons)
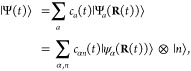
154where {|Ψ_*a*_(**R**(*t*))⟩} are the polaritonic basis states that are eigenstates of (see eq 109) and {|*ψ*_*α*_⟩⊗|*n*⟩} are the adiabatic electronic and Fock/number photonic basis states. The time-dependent electronic–photonic wave function |Ψ(*t*)⟩ is evolved by solving the following time-dependent Schrödinger equation (TDSE)
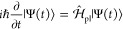
155which leads to the following set of differential equations for the expansion coefficients in the polaritonic basis as,
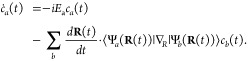
156

Thus, using the ab initio QED approach outlined in Section 3, one can obtain |Ψ_*a*_(**R**(*t*))⟩ and directly solve eq 156 using the propagation of **R**(*t*). Note that a similar expression can be obtained for the *c*_*αn*_(*t*) when using the adiabatic-Fock representation instead. Note that the derivative couplings in this basis (adiabatic-Fock) are sparse since ⟨*n*|**∇**_*R*_|*m*⟩ = 0, as the photonic Fock states have no dependence on the nuclear coordinates unlike the electronic adiabatic ones **d**_*αβ*_ ≡⟨*ψ*_*α*_(**R**(*t*))|**∇**_*R*_|*ψ*_*β*_(**R**(*t*))⟩ ≠ 0 (see eq 23). This is not true for the generalized coherent state (GCS)^183^ or polarized Fock state (PFS)^51^ bases, which intrinsically entangle the electronic and photonic DOFs. Note here that the adiabatic polaritonic states can be obtained through any of the excited state scQED schemes discussed in Section 3.2 while the adiabatic electronic and photonic basis can be computed from bare electronic structure calculations dressed with a photonic basis via the pQED scheme (see Section 3.1).

The force needed to solve Hamilton’s equations of motion for the nuclei can be written as,
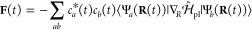
157where the matrix elements  are written as,
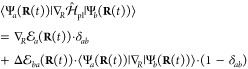
158where Δ_*ba*_(**R**(*t*)) = _*b*_(**R**(*t*)) – _*a*_(**R**(*t*)). Therefore, the forces **F**(*t*) are described by a weighted average over the population times the diagonal nuclear gradients on the polaritonic PESs ∇_*R*__*a*_(**R**(*t*)) *as well as* the coherence-weighted off-diagonal gradient terms Δ_*ba*_(**R**(*t*))·⟨Ψ_*a*_(**R**(*t*))|∇_*R*_|Ψ_*b*_(**R**(*t*))⟩. A similar expression can be obtained when using the adiabatic-Fock basis. The nuclear motion can be solved using a velocity-verlet algorithm.^263,281^

The nuclear DOFs can be initialized by sampling its thermal distribution on the ground state potential energy surface around the Franck–Condon region at a given temperature either by use of BO molecular dynamics (BOMD) using randomly sampled positions and velocities over long-time dynamics or via sampling the classical Wigner distribution. Both methods can be performed at arbitrary temperatures up to the point where the normal-mode analysis breaks down, at which point the system needs to be sampled via BOMD to obtain a meaningful distribution in a highly anharmonic ground state potential.

The elements of the reduced electronic-photonic density matrix can be calculated as an average over the distribution of nuclear configurations as
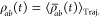
159where *ρ̅*_*ab*_(*t*) = *c*_a_^*^(*t*)*c*_*b*_(*t*) is the density matrix element for a single trajectory (see eq 154 for the definition of *c*_*a*_(*t*)). Any one-particle observable *Ô* can be computed from the reduced density matrix as a trace written as,
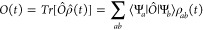
160

Here, *Ô* can be either an electronic, photonic, or nuclear observable. For the case of a nuclear observable, the operator is simply downgraded to a function *O*, removed from the trace, and averaged over all initial conditions.

For light–matter hybrid systems with much lower photon frequencies (such as in the IR regime), a possible alternative is using the above-mentioned mixed quantum classical treatment to group photon modes and nuclear DOF together and propagate them using their classical equations of motion, while treating electronic DOF quantum mechanically (refs (53, 74, 86, 258, 282, and 283)). In this case, the Hamiltonian  is used to evolve the electronic wave function |Ψ(*t*)⟩ = *∑*_*α*__,*n*_c_αn_(*t*)|*ψ*_*α*_(**R**(*t*),*q*_c_(*t*))⟩ quantum mechanically while *R* and *q*_*c*_ are evolved classically.

#### 4.1.3 Ab Initio Nuclear Gradients

For ab initio nonadiabatic dynamics of realistic molecules, the difficulty often is obtaining the necessary components for the propagation of the nuclear and electronic DOFs, such as the gradients of the PESs ∇_*R*_ and more nontrivially the derivative couplings between electronic states **d**_*αβ*_. In polaritonic systems, one encounters new terms which contain gradients on the electronic dipole operators through the light–matter coupling term **∇**_*R*_**μ**_*αβ*_. The gradient expression for the JC type Hamiltonian has been derived in ref (6). For the rigorous PF Hamiltonian (eq 104), the gradient also arises from counter rotating wave term and dipole self-energy terms (see eq 104), where the DSE related term reads ∑_γ_∇_*R*_[(**μ**_*αγ*_·*ê*)(**μ**_*γβ*_·*ê*)]. Explicit expressions for these quantities was recently formulated in the adiabtic-Fock basis for on-the-fly quantum dynamics simulations.^118^ However, these quantities are rarely available in standard electronic structure packages, including the derivative couplings **d**_*αβ*_, due to the complexity of obtaining the analytical expression for excited state electronic structure methods. The analytic derivative couplings have only recently been developed for NAMD simulations for common excited state methods like TD-DFT^284,285^ over the past decade or so and implemented in only a few electronic structure or NAMD packages.^286,287^ Additionally, a recent work indicated that the explicit formulation of the derivative couplings may not be needed and can in fact be approximated very accurately only using the diagonal gradients and potential energies.^288^

Recently, Zhang, Nelson, and Tretiak implemented analytic nuclear gradients on the dipole and simulated the photoexcited dynamics of the stilbene molecule.^289^ In this work, the authors modified the NEXMD software package^189,286,290−299^ to include the pQED Hamiltonian (see Section 3) at the Jaynes-Cummings level with all proper gradients required for this Hamiltonian (i.e., without DSE and making the rotating wave approximation). Additionally, the gradients on the potential energy surfaces, nonadiabatic couplings, and dipole gradients were achieved *analytically* at the TD-AM1^300^ level of theory in the collective electronic oscillator (CEO) framework.^187,286,301^ Most importantly, the nuclear gradient on the bare transition dipole between the ground and excited electronic states was computed as **μ**_0α_ = Tr[**μ̂***X̂*^0α^] in the atomic orbital {*o*, *v*} basis and can be understood as,
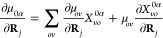
161where *X*_*vo*_^0α^ is the transition density matrix similar to that found in eq 142 between the ground and α_th_ excited electronic state in the CIS-approximation^187,218,231,232^ (see additional discussion in Sec. 3.2) and **μ**_*ov*_ is the transition dipole between atomic orbitals *o* and *v*. From a computational perspective, obtaining both terms in eq 161 is not always trivial and may require additional methods such as iterative optimization algorithms (e.g., biconjugate gradient optimization) to acquire the individual terms themselves, which adds an additional layer of complexity and consideration when performing on-the-fly NAMD simulations inside the cavity.^289^

When generalizing beyond the Jaynes-Cummings model, one needs to additionally account for the excited state permanent and transition dipoles matrix elements.^118^ For more complicated excited state methods (e.g., EOM-CC, CISD, etc., see Section 3.2), acquiring analytic gradients is not trivial and extremely expensive. However, the analytic expression for the nuclear gradients on the atomic orbital dipoles **∇**_*R*_**μ** and transition density **∇**_*R*_X^0α^ (as well as the bare electronic nonadiabatic couplings **d**_*αβ*_ and excited state PES gradients **∇***E*_*α*_) can, in principle, be achieved analytically in any TDSCF method^289,302,303^ and has been shown possible in similar works.^70,257,304,305^ However, the implementation of such quantities in commercial or open-source electronic structure packages are few and far between.^306^

Ref (118) has recently derived the exact nuclear gradient expression for the PF Hamiltonian. This gradient is formulated in the pQED framework (eq 110) and is derived based on the conservation of total energy for the mixed quantum classical system.^118^ As before, the electronic and photonic DOFs are considered as the quantum subsystem, and the nuclear DOFs are considered as the classical subsystem. Using the adiabatic-Fock basis {|*ψ*_*α*_(**R**)⟩⊗|*n*⟩}, the nuclear gradient that corresponding to the PF Hamiltonian in eq 110 is expressed^118^ as follows
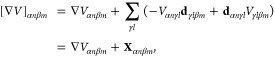
162where *V*_*αnβm*_ = ⟨*ψ*_*α*_*n*|*Ĥ*_pl_|*ψ*_*β*_*m*⟩ (see *Ĥ*_pl_ in eq 110), **d**_*αnβm*_ = ⟨*ψ*_*α*_*n*|**∇**|*ψ*_*β*_*m*⟩ are the derivative couplings among the polaritonic states, and **X**_*αnβm*_ = ∑_*γl*_ (−*V*_*αnγl*_**d**_*γlβm*_ + **d**_*αnγl*_*V*_*γlβm*_) is the gradient originating from the derivative coupling matrix elements. A simplification can be made by noting that the derivative couplings between electronic basis states of different photon numbers vanish as **d**_*αnβm*_ = ⟨*ψ*_*α*_|**∇**|*ψ*_*β*_⟩*δ*_*nm*_.

The above general expression of the nuclear gradient naturally reduces back to the gradient of a Jaynes-Cummings model^5,70,126,257,289,305^ in the subspace of {|*e*,0⟩ ≡ |*e*⟩⊗|0⟩, |*g*,1⟩ ≡ |*g*⟩⊗|1⟩}. As shown in ref (118), the non-JC **X**_*ij*_ components (colored curves) in the general gradient expression have a similar magnitude compared to the regular JC-type gradient (black curve). When the system starts to explore all of the states and generate sizable populations and coherences among them, their contributions in the nuclear force are required to be explicitly and correctly counted. All of these terms are missing in many of the recent MQC studies of polariton dynamics based upon the JC model.^5,70,257,305^ This new gradient expression has also been used in the quasi-diabatic propagation simulation of polariton quantum dynamics.^180^

All possible permanent and transition dipoles (between all electronic states) as well as their derivatives are necessary ingredients to perform polariton dynamics simulations. However, these quantities are rarely available in most commonly used excited-state electronic structure methods and have required approximations toward obtaining these gradients.^138,177,307,308^ In this case, one may turn to *machine learning* techniques to circumvent this need.^309^ Recently, ref (309) implemented such a scheme for the simulation of ab initio polariton dynamics. In this work, the authors employed the kernel ridge regression (KRR) method, which yields an accurate and analytically differentiable dipole. The gradient related to the derivative of the dipole can then be analytically computed. Using this approach, the authors successfully simulated the photoexcited isomerization via conical intersection dynamics of the azomethane molecule after benchmarking the molecular dipoles against numerical gradients.

#### 4.1.4 Fewest Switches Surface Hopping

Surface Hopping (SH) approach is a widely used approximate quantum dynamics approach^310,311^ for simulating nonadiabatic molecular dynamics. The SH approach, a mixed-quantum classical approach (also see EH approach in Section 4.1.4), is a stochastic method where the nuclear DOFs “jump” or “hop” between adiabatic states. Between such hops, the nuclear DOFs evolve classically following one adiabatic state referred to as the active state. This is in contrast to the mean-field EH approach where the nuclear DOFs evolve over a mean surface. This approach has also been recently used to simulate polariton chemistry.^70,118,180,257,289,305,312,313^

Since there are many flavors of SH dynamics present in the community, we only present the procedure for the most commonly used implementation by the community with others briefly mentioned below. Here, we provide a brief overview of the fewest switches SH (FSSH) approach for simulating polariton quantum dynamics. Similar to the EH approach the electronic-photonic subsystem is treated quantum mechanically while the nuclear DOF are evolved classically. Just as in Section 4.1.4 the electronic-photonic wave function is written as,
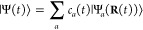
163

The expansion coefficients *c*_*a*_(*t*) undergo direct TDSE propagation as in eq 156. The forces on the nuclear DOFs then simplify to,
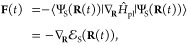
164which only includes the gradient along a single polaritonic PES corresponding to the active state |Ψ_S_(**R**(*t*))⟩. The active state S jumps from polaritonic state S = *a* to S = *b* with probability  as,
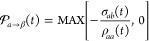
165with
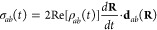
166where **d**_*ab*_ (**R**) = ⟨Ψ_*a*_(**R**(*t*))|**∇**_*R*_|Ψ_*b*_(**R**(*t*))⟩ and *ρ*_*ab*_ (*t*) = *c*_a_^*^(*t*)*c*_*b*_(*t*). The hop from polaritonic state S = *a* to S = *b* will occur if the following condition is met,
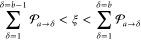
167

At the moment of a hop, the velocities of the nuclei are rescaled in the direction of the nonadiabtic coupling vectors **d**_*ab*_(**R**) ∼ (*d***R**/*dt*)_new_ – (*d***R**/*dt*)_old_ to retain a constant total energy.^314^ If no solution exists to rescale in this direction, the hop is called “frustrated” and is usually discarded or the velocities of the nuclei are simply reversed and the active state remains the same.^286^

The initial conditions are similar to that of the EH approach; however, if there exists a distribution of polaritonic coefficients at initial time {*c*_*α*_(0)}, then initial active state should also be sampled independently for each trajectory (similarly to sampling of nuclear DOFs) from the probability distribution defined by . It is well-known,^311^ FSSH suffers from producing overly coherent (or lack of proper electronic decoherence) within the expansion electronic coefficients and will subsequently be problematic for the polaritonic coefficients.^311^ Many *ad hoc* corrections exist to modify the expansion coefficients in FSSH to account for decoherence, such as the instantaneous decoherence correction (IDC),^275,286^ the energy-based decoherence correction (EDC),^315^ etc., as well as other forms of the surface hopping scheme, such as the augmented surface hopping (A-FSSH),^316^ the decoherence-induced surface hopping (DISH),^317^ and the global flux surface hopping^318^ schemes.

A major simplicity afforded by the FSSH method is that the derivative coupling vectors **d**_*ab*_(**R**) are not explicitly required as the nuclear forces (unlike in the mean-field EH method) do not require this quantity for time-evolution (except at the hops for rescaling), and the electronic propagation only requires the scalar nonadiabatic coupling terms **d**_*ab*_ · *d***R**/*dt* = ⟨Ψ_*a*_|*d*/*dt*|Ψ_*b*_⟩, which can be easily obtained via finite difference wave function overlaps of the polaritonic states throughout the trajectory.^319,320^ This procedure is immensely cheaper than the direct computation of the nonadiabatic coupling vectors themselves, wherein one only needs to compute the nonadiabatic coupling vectors to rescale the nuclei at the moment of a hop.^286^ Or, one can ignore the asymmetric nuclear velocity rescaling altogether and perform uniform energy-based rescaling, which is known to provide slightly worse dynamics but alleviates the computation of the vector nonadiabatic coupling altogether.

#### 4.1.5 Other Approximate NAMD Methods

There exist a multitude of other schemes to approximately solve the TDSE for a realistic system that will not be discussed in this review. However, future applications in simulating polaritonic dynamics will require the use of more accurate methods compared to EH and FSSH. Similar methods to EH exist that are an extension to the Meyer-Miller-Stock-Thoss mapping schemes^321,322^ and lead to methods such as the symmetric quasi-classical (SQC),^323−333^ partially linearized density matrix (PLDM)^334−336^ and later the spin-mapping (SM) approaches,^337−342^ which are all mean-field-level methods in that they treat the forces on the nuclear DOFs as an average over the electronic state population and coherences similar to the EH method but all drastically outperform EH through, for example, the inclusion of zero-point energy (all methods) or using the correct mapping space to constrain the population (sM). Note that many of these approximate quantum dynamics approaches (such as PLDM, SQC, SM, etc.) are formulated in the diabatic representation and are incompatible with adiabatic electronic or polaritonic representation. The recently developed quasi-diabatic scheme resolves this issue and allows combining any of these diabatic dynamics approaches with adiabatic electronic or polaritonic representation without requiring any additional nontrivial theoretical efforts such as diabatization.^180,329,332,335,343^ Finally, methods stemming from the exact factorization (XF) formalism, which range from trajectory-based XF surface hopping (XFSH) to coupled trajectory approaches (CTXF), can also be utilized in the polaritonic basis which may lead to additional methods depending on the choice of factorization of the electronic, photonic, and nuclear DOFs.^344−351^

### 4.2 Influencing Photochemical Reactivities through Light–Matter Hybridization

**Figure 13:**
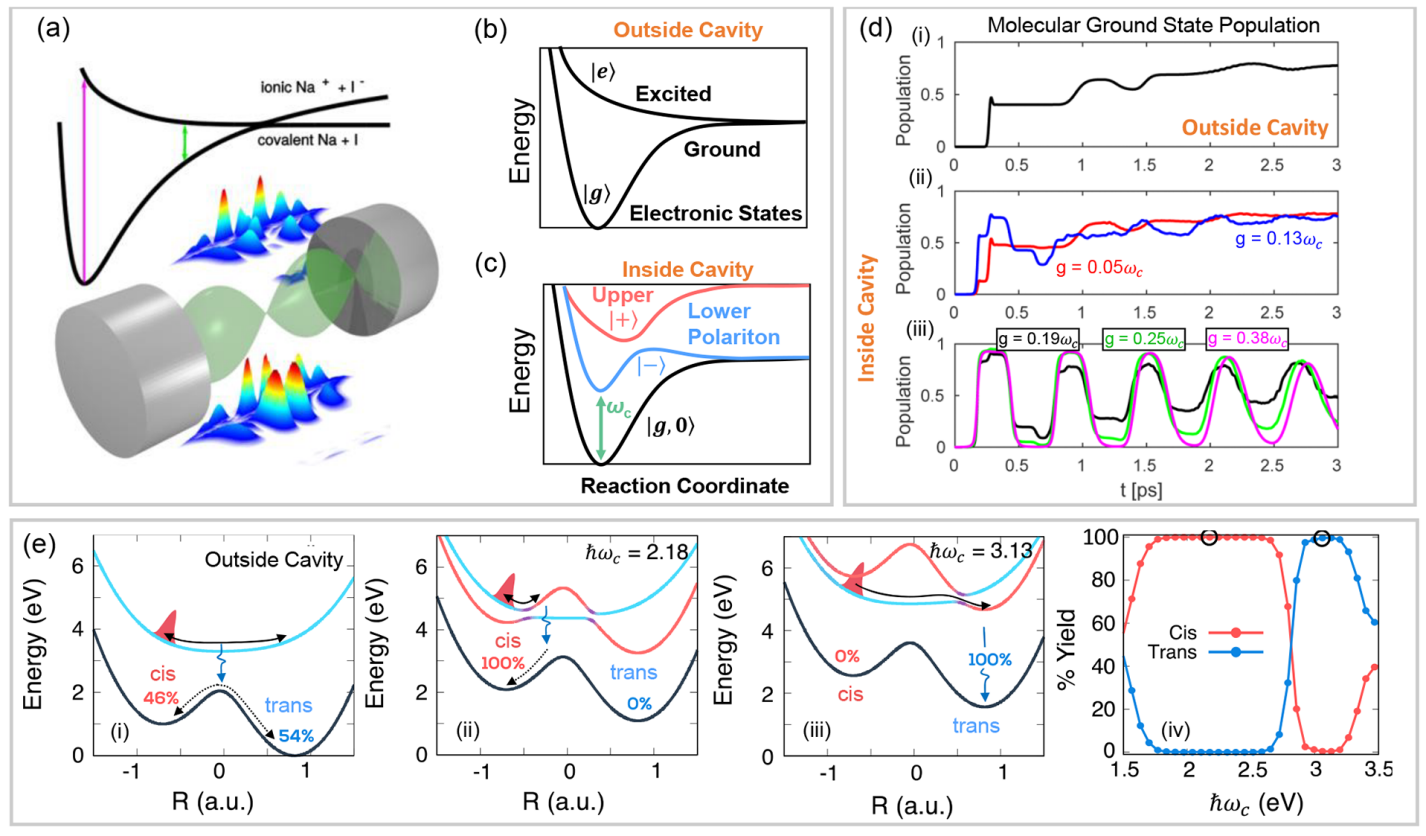
Polariton Photochemistry: Modifying molecular photochemistry through light–matter hybridization. (a) Schematic illustration of a cavity and the potential energy surfaces of an uncoupled NaI molecule with ionic and covalent molecular states. (b) Ground and excited state potential energy surfaces for a molecule outside the cavity and (c) when coupling the reaction to a cavity leading to the formation of the upper (red) and lower (blue) polaritons. The cavity frequency ω_c_ is shown by the green arrow. (d) Photodissociation of a NaI molecule inside and outside the cavity. Subpanel (i) presents the molecular ground state population dynamics after photoexcitation outside the cavity while subpanels (ii-iii) present these population dynamics inside the cavity at various light–matter couplings *g*. (e) Modifying a photoisomerization reaction inside cavity. Subpanel (i) shows the molecular potential energy surfaces outside the cavity. These surfaces result in a nearly 50%/50% mixture of *cis* and *trans* after reacting on the excited surfaces. Subpanels (ii, iii) show the PESs when coupling to cavities of different photon frequencies *ℏω*_c_. These hybrid surfaces allow either nearly 100% *cis* (subpanel (ii)) selectivity or nearly 100% *trans* (subpanel (iii)) selectivity. Subpanel (iv) shows the relative % yield of the *cis* or *trans* isomer as a function of *ℏω*_c_. Panels (a) and (d) are adapted from ref (5) with permission. Copyright 2016 American Chemical Society. Panel (e) is adapted from ref (13) with permission. Copyright 2019 American Chemical Society.

Coupling molecular excitations to a cavity photonic excitation causes a hybridization of both types of excitations, leading to the creation of new light–matter hybrid states.^10^ When the PESs of the molecular ground and excited states are considered, the light–matter hybridization creates hybrid polariton surfaces, as discussed in Section 1.1 (see eq 11). These polariton surfaces hybridize the curvatures from both the ground and the excited molecular states (see Figures 6, 8, and 13) and possess different levels of matter or photonic excited character as a function of their nuclear coordinates (as we have seen). Additionally, the curvature of these surfaces is modulated by the Rabi splitting and creates new light–matter avoided crossings. These features of the potential energy surfaces can modify the path that a chemical reaction takes, resulting in a polariton-induced change of reactivity. By tuning the cavity frequency ω_c_ and light–matter coupling strength *g*_c_, the features of these hybrid polariton surfaces can be optimized to control the outcomes of a variety of photochemical reactions.

The effects of changing ω_c_ and *g*_c_ on the hybrid polariton surfaces can be understood as follows. Changing the cavity frequency ω_c_ will change the energy of a quantum state that has *n* photons associated with it by the amount *ℏω*_c_*n*. For an electronic transition between a molecular ground and excited state that is coupled to the cavity photon mode, the molecular ground state with *n* + 1 photons (the |*g*,*n*+1⟩ state) will couple to the molecule excited state with *n* photons (the |*e*,*n*⟩ state). When the PESs of these ground and excited states have different curvatures, different energetic shifts of *ℏω*_c_ will cause the |*g*,*n*+1⟩ and |*e*,*n*⟩ PESs to intersect at different nuclear configurations. Different points of intersection (in the nuclear configurational space) lead to different composite curvatures for the upper and lower polariton surfaces which will affect the force the nuclei feel at a given configuration, thus influencing the motion of the nuclear DOFs and altering the reaction pathways compared to the bare molecules outside the cavity. Note that in the above intuitive argument, we have interpreted the Fock state |*n*⟩ as *n* photons contained inside the cavity. This is only true when there is no matter inside the cavity, and approximately accurate when the light–matter coupling strength is weak. Rigorously, the photon number operator needs to be gauge transformed as discussed in eq 62.

Changes of the light–matter coupling strength *g*_c_ have two primary effects. The first is that the upper and lower surfaces will energetically “split” apart where the |*g*,*n*+1⟩ and |*e*,*n*⟩ PESs intersect, by the energy of Rabi splitting which is  when considering the JC model (see eq 5). This is also known as a cavity-induced avoided crossing,^5,10^ which can impact how much populations on the upper and lower polaritons can transfer to each other. The second effect is that larger values of *g*_c_ will increase the extent of the regions of the polariton surfaces that have mixed electronic-photonic excited character. This change in excited character can impact how strongly these polariton states interact with other quantum states.

These cavity-induced effects can be clearly demonstrated using simple single-molecule model reactions, which is ideal for an experimental setup with certain plasmonic cavities^27^ (see Figure 1a). One of the simplest photochemical reactions is that of bond photodissociation. The primary mechanism of this reaction is a Franck–Condon photoexcitation of a molecule to a molecular excited state, which has a curvature that pushes the nuclei away from the bonded regime and toward the dissociated regime. Absent this photoexcitation, the nuclear wavepacket remains in the equilibrium geometry of the ground state potential and resists dissociation. How exactly to translate this PES hybridization principle into the collective coupling regime is still an open question, and the recent progress along this direction will be discussed in Section 6.2.

Recent theoretical works have examined the effects of coupling the ground-excited transition of photodissociation reactions to optical cavities.^5,6,10,21,117,119,127,267,352−355^Figure 13a,b illustrates the typical molecular ground and excited surfaces present in photodissociation reactions which are composed of covalent and ionic bond characters.^5^ A key feature of these surfaces is that they become nearly degenerate at some finite nuclear distance away from the equilibrium bond configuration. Additionally, some nuclear configurations have larger gaps between the excited and ground surfaces (pink arrow) than other configurations (green arrow). Tuning the cavity frequency to match these energy gaps will create a *cavity-induced avoided crossing* at that respective nuclear configuration, which will generally be closer to the equilibrium bond configuration than the original molecular ground-excited avoided crossing. In particular, Figure 13c demonstrates the effect of hybridizing the molecule excited state |*e*,0⟩ with the photon-dressed ground state |*g*,1⟩. The |*g*,1⟩ state surface has the *same* curvature of the molecule ground state |*g*(*R*)⟩ and is energetically raised by *ℏω*_c_ (due to the single photon dressing) which allows it to intersect and hybridize with the molecule excited surface |*e*,0⟩, much closer to the equilibrium bond configuration than the bare molecule surfaces illustrated in Figure 13b. This causes the upper polariton surface (red curve in Figure 13c) to have a broad well shape that resists dissociation, and the lower polariton surface (blue curve in Figure 13c) to have a potential well centered around the equilibrium bond configuration. Upon Franck–Condon photoexcitation and with large Rabi splittings, the curvatures of these surfaces encourage the nuclear wavepacket to stay near the equilibrium bond configuration until the excitation eventually relaxes to the molecule ground state through loss channels. Figure 13d shows molecular ground state population dynamics of the dissociation reaction of a NaI molecule coupled to an optical cavity, as illustrated in panels a–c. With (i) no coupling or (ii) weak light–matter coupling, a large portion of the nuclear wavepacket moves toward ionic–covalent avoided crossing, transfers to the flat part of the covalent curve, and dissociates readily. With (iii) a stronger light–matter coupling, the nuclear wavepacket becomes trapped in the wells of the upper polariton state (red surface shown in panel c), resulting in an oscillatory covalent character and less dissociation since the original ionic–covalent avoided crossing and energetic plateau is not reached by the wave packet.

Coupling photoisomerization reactions to an optical cavity have also been shown to alter the reactive outcomes, both experimentally^3^ and theoretically.^7,10,13,256^Figure 13e(i), adapted from ref (13), presents the model isomerization reaction, with a ground state PES (black curve) which has two well-defined minima that correspond to the *cis* and *trans* configurations. The excited state |*e*⟩ (cyan curve) is modeled with a relative flat PES due to the delocalization of the electron density. Panels (ii, iii) present the modifications of the polariton potentials due to light–matter coupling, for two different cavity photon frequencies. In the zero coupling case, outside the cavity (i), the nuclear wavepacket, once excited to the state |*e*⟩, can freely explore the excited PES. Once the decay channels take over (radiative and nonradiative decay), the system will relax back to the ground state |*g*⟩, and end up in either in the *cis* or the *trans* nuclear configuration. The reaction exhibits barely any selectivity for the *cis* or *trans* configuration. When the molecules are coupled inside the cavity (ii-iii), the excited surface curvatures are modified specifically based on the cavity frequency. For cavity frequency ω_c_ = 2.18 au (ii), the emerging feature of the potential is a new barrier on the upper polariton surface. Through a Franck–Condon excitation of the system, a nuclear wavepacket is placed on the upper polariton surface. Due to the presence of the new barrier, the nuclear wavepacket is trapped on the left side (cis) which gives *cis* selectivity upon relaxation to the ground state. Alternatively, for cavity frequency ω_c_ = 3.13 au (iii), a nuclear wavepacket starting on the lower polariton surface transfers to the *trans* side of the nuclear configuration space and becomes trapped in a potential well, resulting in *trans* selectivity upon relaxation to the ground state. As a consequence, the percent yield of the isomerization reaction (iv) can be controlled to be nearly 100% *cis* or 100% *trans* by tuning the cavity frequency.

These theoretical investigations on the hybridization of light and matter excited surfaces highlight the possibility for photochemistry to be controlled by tuning the coupling strength *g*_c_ and the cavity frequency ω_c_. However, the investigations in Figure 13 only involved a single molecule coupled to a single mode with idealized model potentials inside a lossless cavity. These simplifications merit further investigation into simulations of more realistic polaritonic systems. In particular, there exist several other factors that play significant roles in the ability to control photochemistry, which will be elaborated upon in the following subsections. The collective coupling effect will be extensively discussed in Section 6. On the other hand, the theoretical investigations presented in this section might be able to be carried out in actual experimental investigations using a plasmonic cavity setup.^27^

### 4.3 Ab Initio Simulations of Polariton Photo-Isomerizations

Utilizing the NAMD methods described in Section 4.1, several realistic ab initio simulations of polariton-mediated photochemical reactions have been investigated.^70,257,356^ Using these methods provides a simulation with more atomistic details compared to the simpler model simulations (e.g., in Figure 13e) and allows for detailed molecular insight into cavity modified photochemical reactions.

**Figure 14:**
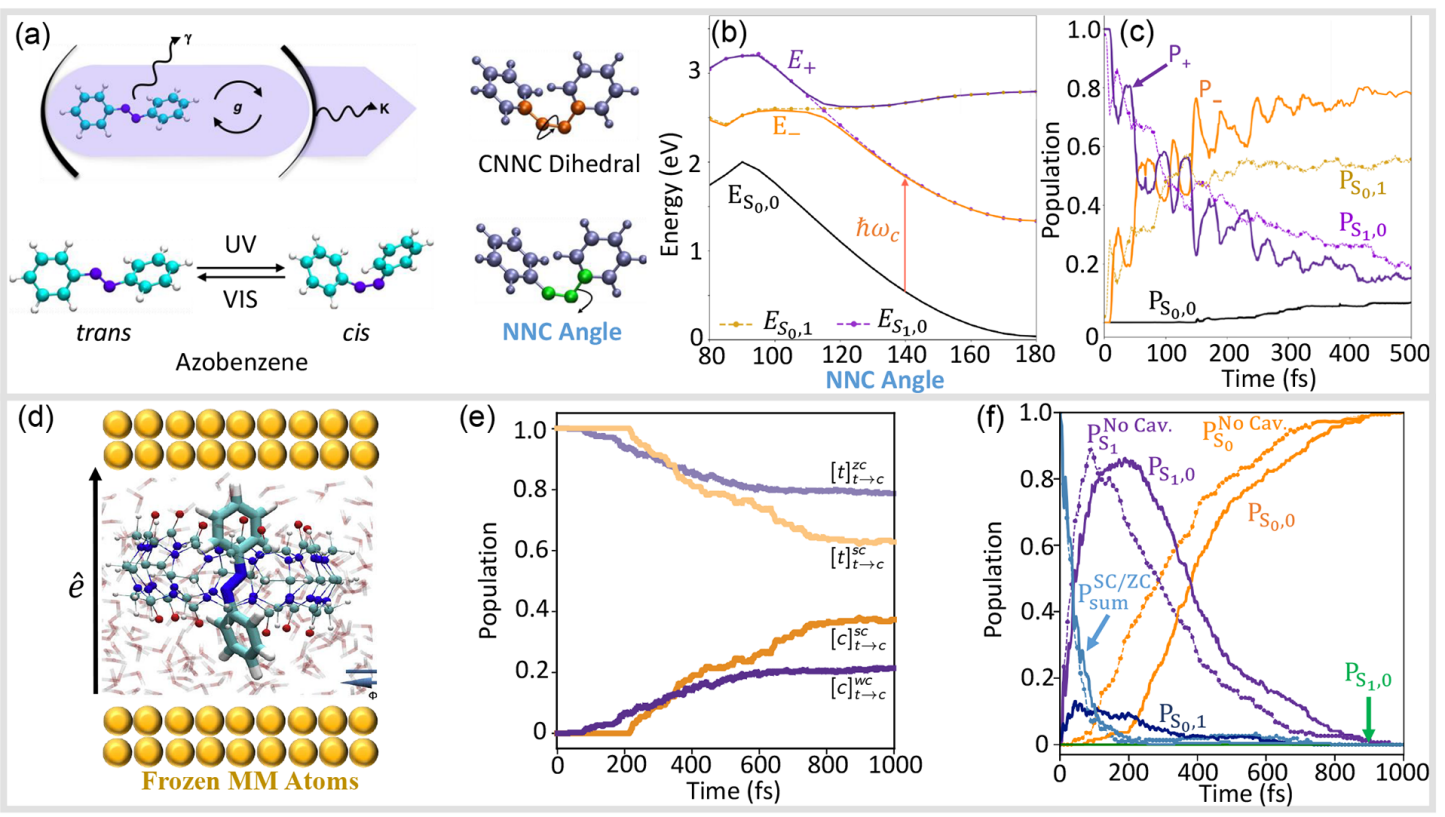
Realistic photochemistry. (a) (Top, left) Schematic of the azobenzene molecule coupled to a Fabry–Pérot cavity with coupling strength *g*, cavity loss κ, and molecular photon emission rate γ. (Bottom, left) The isomerization reaction of azobenzene from the *trans* to the *cis* configuration at ultraviolet (UV) wavelengths and the reverse at visible (VIS) wavelengths. (Right) Two dominating molecular coordinates for the CNNC torsional dihedral angle as well as the NNC angle which dictate the intrinsic bare molecular conical intersection and subsequent cavity-induced conical intersection. (b) Polaritonic potential energy surfaces at the Jaynes-Cummings level with the uncoupled ground state *E*_*S*_0_,0_ (black) as well as the upper *E*_+_ (purple) and lower *E*_–_ (orange) polaritonic states at the semiempirical AM1 level coupled with the floating occupation number molecular orbital configuration interaction (FOMO–CI) approach. (c) Populations of the various states (solid lines, color-coded with panel b) after initial excitation to the upper polaritonic state. The dashed lines with symbols are the populations of the basis states |*P*_S_0_,1_⟩ (tan) and |*P*_S_1_,1_⟩. Here, no cavity loss or molecular photon emission rates were used (i.e., κ, γ = 0), assuming a perfect cavity and infinitely long molecular emission time. The cavity coupling was set to *g* = 0.01 au and cavity polarization *ê* perpendicular to the main axis of the mirror, as shown in panel (a). (d) Schematic of the azobenzene molecule in a plasmonic cavity with polarization *ê* shown by the black arrow. The computational system includes QM (azobenzene) and MM (water solvent and metal lattice atoms). (e) Population dynamics of the *trans* and *cis* populations for zero coupling strength (zc, purple lines) and strong coupling (sc, orange lines). Strong coupling results in a larger steady-state *cis* population. (f) Population dynamics of the diabatic states for outside the cavity (dotted lines) and inside the cavity (solid lines). The excited diabatic states inside the cavity take longer to decay to the ground state than outside the cavity. Panels (a–c) are adapted from ref (70) under the CC BY license. Panels (d–f) are adapted with permission from ref (257). Copyright 2019 Elsevier Inc.

The influence of cavity coupling on the mechanisms of the photoisomerization of azobenzene was investigated through realistic ab initio simulations^70,257^ in the work shown in Figure 14a–c, adapted from ref (70). The reaction involves photoexcitation of azobenzene under ultraviolet light, which allows for isomerization from *trans* to *cis* on the excited state potential energy surfaces (Figure 14a). In this molecule, there is an intrinsic conical intersection between the *S*_0_ (ground adiabatic electronic state) and *S*_1_ (first excited adiabatic electronic state) potential energy surfaces, in the nuclear configurational space of the CNNC dihedral angle (coupling coordinate) and NNC angle (stretching coordinate). Both coordinates are illustrated in Figure 14a. The light–matter interaction between the |*S*_0_,1⟩ and |*S*_1_,0⟩ surfaces causes a Rabi splitting between the upper and lower polariton surfaces, as shown along the NNC angular DOF in Figure 14b.

The electronic structure was computed using the pQED scheme (see Section 3.1) using the Jaynes-Cummings Hamiltonian (i.e., no counter-rotating terms or dipole self-energy) with a minimal basis of |*S*_0_,0⟩, |*S*_0_,1⟩, and |*S*_1_,0⟩. Here the electronic structure was computed at the semiempirical AM1 level^300^ coupled with the floating occupation molecular orbital configuration interaction (FOMO–CI) scheme^70,357−359^ for the calculation of the lowest singlet excited state *S*_1_.

The Rabi splitting is nuclear configuration dependent, due to the nuclear-dependent adiabatic energy gap and dipole (both transition and permanent dipoles). This avoided crossing region centered at the Rabi splitting, along with the nearby polariton-induced conical intersection^70^ (where the light–matter coupling term **μ̂·ê** (eq 104) goes to zero because the component of the dipole along the cavity field polarization direction goes to zero for a certain nuclear configuration), allows for population to transfer between the upper and lower polariton surfaces.

Figure 14c presents the polariton population dynamics computed with a decoherence-corrected surface hopping approach.^70^ While the diabatic excited state population dynamics of |*S*_0_,1⟩ (golden dotted line) and |*S*_1_,0⟩ (purple dotted line) show a trend of smooth decays/increases, the upper polariton population *P*_+_ (purple solid line) and lower polariton population *P*_–_ (orange solid line) show oscillations which are mediated by of the polaritonic avoided crossing and polaritonic conical intersection.^70^ The consequence of these population transitions is that a large amount of population was transferred to the lower polariton whose curvature resists a conversion from the *trans* to the *cis* configuration, resulting in a quenching of the photoisomerization reaction rate relative to outside the cavity.

In the previous example, the coupling of the azobenzene photoisomerization reaction to a cavity was seen to reduce the isomerization quantum yield relative to outside the cavity. However, in another work,^257^ the authors showed that one is able to enhance the rate of the photoisomerization reaction (see Figure 14d–f). Here, the azobenzene molecule is confined inside a molecular ring (or host molecule) using a QM/MM level of description with the molecular ring and explicit water solvent treated at the MM level. Both the molecular rings, solvent, and azobenzene are further situated between two gold planar mirrors (Figure 14d). This configuration of the simulation closely resembles some actual experiments where a single molecule is coupled to a plasmonic cavity in ref (27). In principle, one should be able to experimentally check the prediction of this simulation work.^257^ In this calculation, several higher molecular excited states were included,^257^ increasing the chemical accuracy of the simulation relative to the simulations that only consider a single electronic excited state. The population dynamics revealed that strong light–matter coupling enhanced the conversion of the *trans* to *cis* configurations (Figure 14e). In particular, the *trans* to *cis* reaction was faster at short times outside the cavity, but the strong light–matter coupling allowed the reaction inside the cavity to persist much longer. This resulted in a steady state *cis* population nearly twice that of outside the cavity. The proposed mechanism for this photoisomerization rate enhancement is that the photonic |*S*_0_,1⟩ state acts as a reservoir for the |*S*_1_,0⟩ state population which helps to delay the decay to the ground state before the isomerization can occur. This can be seen in the population dynamics of the diabatic states (Figure 14f) where the strong coupling |*S*_1_,0⟩ state maintains a large population for longer than the no cavity |S_1_⟩ state.

These studies on the photoisomerization of azobenzene inside optical cavities demonstrate that the details of the electronic structure and surrounding environment can have a strong influence on the ability of cavity coupling to control chemical reactions. In particular, it was seen that the reaction modeled in Figure 14a–c experienced more quenching and less steady state *cis* product compared to outside the cavity, whereas the reaction modeled in Figure 14d–f was able to enhance the isomerization relative to outside the cavity. These differences in reactivity can arise due to experimentally relevant differences in the details of the structural setup of the model, which may not be able to be captured in simpler model systems that lack ab initio detail. Thus, it is important to verify the results of simple model simulations with more realistic ab initio simulations whenever possible and to explore the different photochemical reaction mechanisms inside optical cavities that are possible when utilizing electronic structure calculations during the reaction dynamics.

### 4.4 Polariton-Mediated Charge Transfer Reactions

Another fundamental, yet important, type of photochemical reaction is excited state charge transfer. The basic principle of this reaction is that a charged particle, often an electron, can transfer among molecules after the system is excited, often due to photoexcitation. This transfer is allowed by the presence of electronic coupling between so-called “donor” and “acceptor” states.^360−368^ Accompanying the transfer of charge, there is often a reorganization of the nuclei based on the new electric potential of the acceptor state. The free energy difference Δ*G*, donor–acceptor coupling strength *V*_DA_, and reorganization energy λ all play key roles in the rate of excited state charge transfer.

Marcus theory^369−372^ is one of the most commonly used descriptions of charge transfer in the weak donor–acceptor coupling regime when *V*_DA_ ≪ *k*_B_*T*, where *k*_B_ is the Boltzmann constant and *T* is the temperature. The electron transfer rate constant *k*_ET_ for the |D⟩ → |A⟩ transition described by Marcus theory^369−372^ is
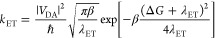
168where *V*_DA_ = ⟨D|*Ĥ*_el_|A⟩ is the donor–acceptor coupling strength, β = 1/*k*_B_*T* with Boltzmann constant *k*_B_ and temperature *T*, λ_ET_ is the reorganization energy associated with the electron transfer reaction (not to be confused by the light–matter coupling strength in eq 105), and Δ*G* is the difference in free energy between the donor and acceptor states (also known as the driving force). When including the ground state in this description, the ground, donor, and acceptor states can be thought of as parabolas that are shifted from each other in terms of their minimum energy and nuclear configuration, with coupling between the donor and acceptor parabolas. While there have been experimental demonstrations of photoinduced charge transfer enhancement in organic crystals^373,374^ and solar cells^375−377^ with the use of external laser driving, these reaction modifications can potentially also be achieved through strong coupling to a cavity.

**Figure 15:**
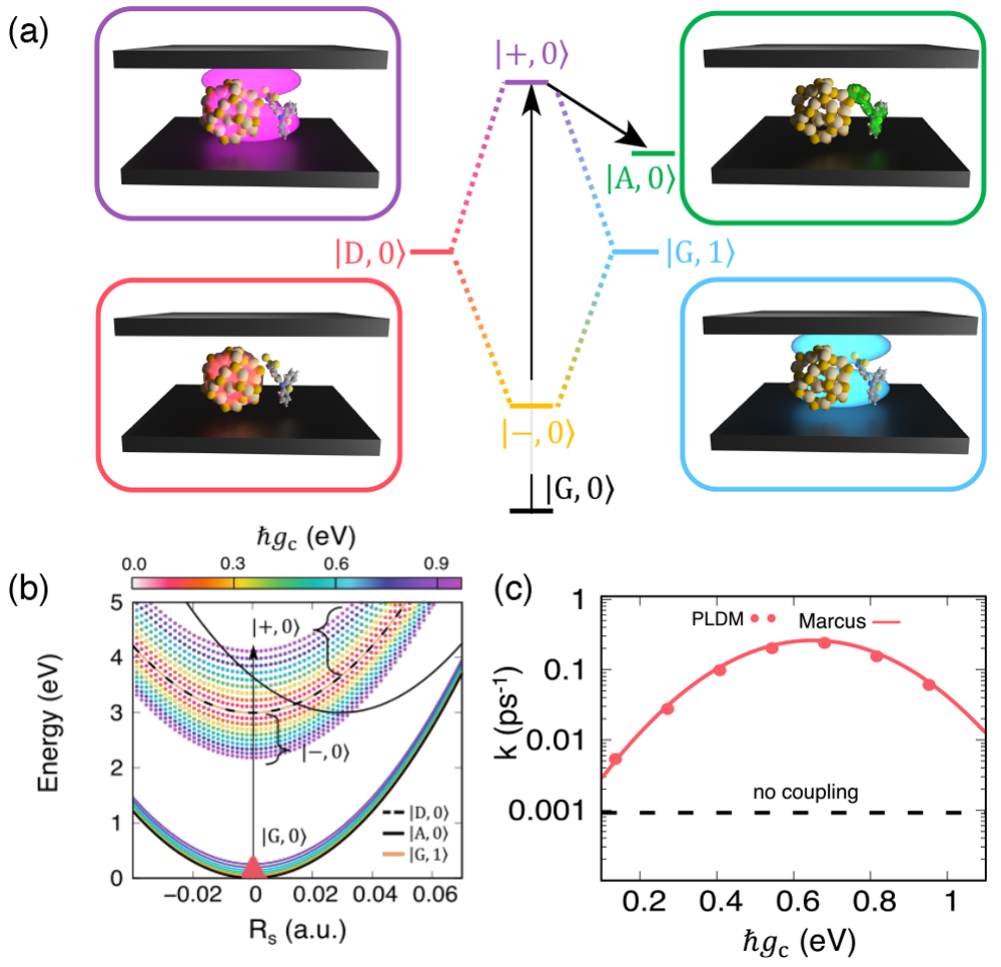
Polariton-mediated electron transfer. (a) Schematic illustration of modifying the driving force of photoinduced electron transfer reaction by coupling to the cavity. Polariton state |+,0⟩ lie above the acceptor state |*A*,0⟩ (allowing downhill chemical reaction) while the original donor state |*D*,0⟩ lies below |*A*,0⟩. (b) Polaritonic potentials |*G*, 0⟩, |±,0⟩ that are color coded by light–matter coupling strength *g*_c_. (c) Electron transfer rate constant as a function of light–matter coupling strength *g*_c_ computed from Marcus theory and from direct quantum dynamics simulation using the PLDM approach.^334,336^ Adapted from ref (12). with permissions. Copyright 2020 American Chemical Society.

When a charge transfer reaction is coupled to an optical cavity, modifications to many of the key parameters in eq 168 due to light–matter coupling can be calculated. We emphasize that such modifications have not been observed experimentally and that more experiments need to be performed to validate the theoretical principles of polariton mediated charge transfer. Here, we use this simple analysis to illustrate how modifications would affect the corresponding Marcus-regime charge transfer rates. One of the most important modifications is to the driving force Δ*G* as shown in Figure 15a, adapted from ref (12). In this model, three diabatic electronic states are considered: a ground state |G⟩, an optically bright excited state denoted as the donor state |D⟩, and an optically dark excited state, denoted as the acceptor state |A⟩. This model setup could correspond to many experimental systems such as a colloidal nanocrystal (NC) as a donor molecule and an organic acceptor molecule.^368,378^ The molecular excitation transition |G⟩ → |D⟩ is coupled to the cavity (due to its nonzero ground-to-excited transition dipole moment) whereas the acceptor state |A⟩ (which is also an electronic excited state) is not directly coupled to the cavity but is coupled to the donor state |D⟩ through the diabatic electronic coupling *V*_DA_. It is also assumed that the donor excited state |D⟩ and the ground state |G⟩ have the same minima position, meaning there is no Huang–Rhys factor (or reorganization energy) between |D⟩ and |G⟩. This means that the |G,1⟩ state (orange solid curve in Figure 15b) and the |D,0⟩ state (black dashed curve in Figure 15b) are nested with the same minimum position, indicating the ET reorganization energy λ is not changed upon coupling to the cavity (as opposed to the case illustrated in Figure 28a).

Assuming a thermal equilibrium in the ground state and quasi-classical nuclear initial conditions upon instantaneous photoexcitation, Marcus theory can be used to describe the charge transfer rate between the |±,0⟩ state to the |A,0⟩ state. The polariton-mediated electron transfer (PMET) rate constant is expressed as
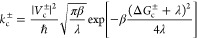
169where Δ*G*_c_^±^ is the polariton-mediated driving force between the photon-dressed acceptor state |A,0⟩ and the polariton |±⟩ states (only considering the *n* = 0 case of the JC ladder in eq 4), is expressed as
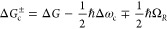
170where *ℏ*Δω_c_ = *ℏω*_c_ – (*E*_D_ – *E*_G_) is the light–matter detuning, and *V*_c_^±^ is the polariton-mediated coupling
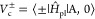
171

Since the acceptor state does not carry any ground-to-excited transition dipole, the matter-cavity coupling term *g*_c_ does not provide any coupling between polariton states and the |A,0⟩ state. Thus, the polariton-mediated effective coupling *V*_c_^±^ only has a contribution from the electronic Hamiltonian operator. Under the JC model consideration, the cavity-mediated electronic couplings between |+,0⟩ and |A,0⟩ states is
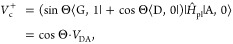
172and similarly, *V*_c_^–^ = sin Θ·*V*_DA_. Thus, the effect of light–matter coupling always reduces the effective electronic couplings between the |±⟩ states to the acceptor state |A,0⟩. For the resonant coupling condition *E*_D_ – *E*_G_ = *ℏω*_c_, , and thus , resulting in a 2-fold reduction of the rate due to the light–matter hybridization. However, more significant modifications can come from the exponential part of eq 169, which depends on Δ*G*_c_^±^.

When considering a wide range of light–matter coupling strengths *ℏg*_c_, the PMET driving force Δ*G*_c_^±^ and thus the PMET rate (eq 169) can be tuned significantly. Note that eq 169 is based on the JC model, which is simple and intuitive but will eventually breakdown (see Figure 3) when *g*_c_/ω_c_ ≥ 0.1. Directly numerical calculations of Δ*G*_c_^±^ and *V*_c_^±^ are necessary when going beyond the JC approximation, which is detailed in ref (12). Figure 15b demonstrates the upper and lower polariton surfaces and their energetic shifts for several different Rabi splittings. Comparing these polariton surfaces to the acceptor surface |A,0⟩ in solid black, the polariton surfaces are able to access many different charge transfer regimes (normal, activation-less, and inverted), where the forward ET reaction can be made more or less favorable depending on the initial state (upper or lower polariton) and the magnitude of the Rabi splitting.^12^ This can be seen in the effect of different light–matter coupling strengths *g*_c_ on the PMET rate as predicted from Marcus theory.^12^ The cavity-induced Rabi splitting raises the energy of the upper polariton surface and lowers the energy of the lower polariton surface (Figure 15a). Consequentially, the Δ*G* from the upper polariton to the acceptor will decrease while the Δ*G* from the lower polariton to the acceptor will increase. In particular, if the acceptor state has higher energy than the bare donor state, but lower energy than the upper polariton state, an uphill reaction outside the cavity can be modified as a downhill reaction inside the cavity, when exciting to the upper polariton surface (and when the upper polariton lifetime is long enough for the reaction, e.g., under a continuous irradiation condition that constantly supplies |UP⟩ population.^379^

Figure 15c presents the PMET rates from the upper polariton |+⟩ to the acceptor |A,0⟩ as a function of *ℏg*_c_, predicted from Marcus theory (solid line) and from a partial-linearized density matrix (PLDM) dynamics simulation^334^ (dotted line). The PMET rate can be enhanced by a factor of over 100 for this model when the system is resonantly coupled to an optical cavity with a coupling strength of *ℏg*_c_ = 600 meV. Beyond this coupling strength, the rate begins to decrease due to the |+⟩ state sitting in the Marcus inverted regime. Alternatively, the lower polariton could be initially excited to more readily sample other Marcus regimes resulting in a PMET rate smaller than those outside the cavity.^12^

For systems with donor states that have nonzero reorganization energy relative to the ground state, the donor excited state |D⟩ and the ground state |*G*⟩ are modeled as parabolas with different minima positions, as illustrated in Figure 28a. In the limit that the Rabi splitting is larger than the donor-ground reorganization energy and the light–matter detuning, the polariton states |±⟩ (generated by hybridizing the |D,0⟩ and |G,1⟩ states) are nearly harmonic and have a potential minimum that is in between the minima of the |D⟩ and |G⟩ surfaces, as illustrated in Figure 28a. This results in an effective reduction of the reorganization between the polariton states and the ground state. This effective reorganization energy is reduced by a factor of 1/4 in the strong coupling limit relative to the original donor-ground reorganization energy outside the cavity due to the polariton superposition only having half of the donor character.^8^ Note that the PMET rate is exponentially sensitive to the effective reorganization energy (eq 169). This mechanism of enhancing PMET due to the effective reduction of the ET reorganization energy is referred to as the polaron decoupling mechanism.^8^ Note that this effect only changes the donor-ground reorganization energy and does not affect the acceptor-ground reorganization energy for acceptor states that do not couple to the cavity.

Although we have only considered a single molecule coupled to the cavity, the proposed modification of PMET rate can also be accomplished in the collective coupling regime (which involves many molecules coupled to the cavity as described in Section 6.3), involving both a modification of the effective driving force Δ*G*_c_^379,380^ as well as the polaron decoupling mechanism.^8^

While having different chemical mechanisms, singlet fission reactions share much in common with charge transfer reactions in terms of how they can be controlled using light–matter coupling.^381^ Like charge transfer reactions, singlet fission reactions are often modeled quantum mechanically with singlet and triplet surfaces that are shifted parabolas with certain driving forces and reorganization energies. These fission reactions can thus be controlled through light–matter coupling with the same effects previously described in this section. In particular, theoretical investigations^382−385^ have shown that cavities may increase or decrease triplet yield and production rate depending on the singlet fission parameters as well as cavity parameters such as the cavity frequency and coupling strength. The similar cavity control of singlet fission reactions and charge transfer reactions highlights the broad applicability of cavity modifications to many different types of photochemical reactions.

### 4.5 Cavity-induced Conical Intersections and Berry Phase

Coupling molecules to an optical cavity can also create a new type of conical intersection (CI), which is referred to as the polariton-induced conical intersection (PICI).^117,386^ Conical intersections in general arise when the separation between adiabatic electronic surfaces goes to zero at a particular nuclear configuration, causing a degeneracy (which appears as a cone type of structure). The cavity photon mode and the molecule are coupled through the **λ·μ̂** term in eq 104, which characterizes the light–matter coupling vector oriented in the direction of the cavity polarization unit vector **ê**. We denote the angle between the dipole vector **μ̂** and **ê** as θ (not to be confused with the incident angle in the Fabry–Pérot cavity illustrated in Figure 4), and μ̂ = |**μ̂**|, hence the light–matter coupling can be expressed as
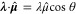
173

For polaritonic systems, the PICI can form when the orientation of a molecule’s ground-to-excited transition dipole moment becomes orthogonal to the cavity polarization vector^117,386^ such that θ = π/2, and the light–matter coupling vanishes at this orientation. Thus, one can see that even for a diatomic molecule where there are no intrinsic electronic CIs, there will be a PICI due to the presence of the additional DOF, i.e., the angle θ between the dipole and the field polarization direction. This angle serves as the tuning coordinate in the CI. One can thus engineer a new CI that did not exist previously by coupling molecules with a cavity. These CIs, either intrinsic or cavity-induced, lead to a singularity in the nonadiabatic coupling (see eq 23) and thus cause a breakdown of the Born–Oppenheimer approximation in the vicinity of the CI. Unlike those intrinsic molecular CIs, cavity-induced CIs depend on the properties of the cavity and can thus be tuned to control photochemical reactivity.

**Figure 16:**
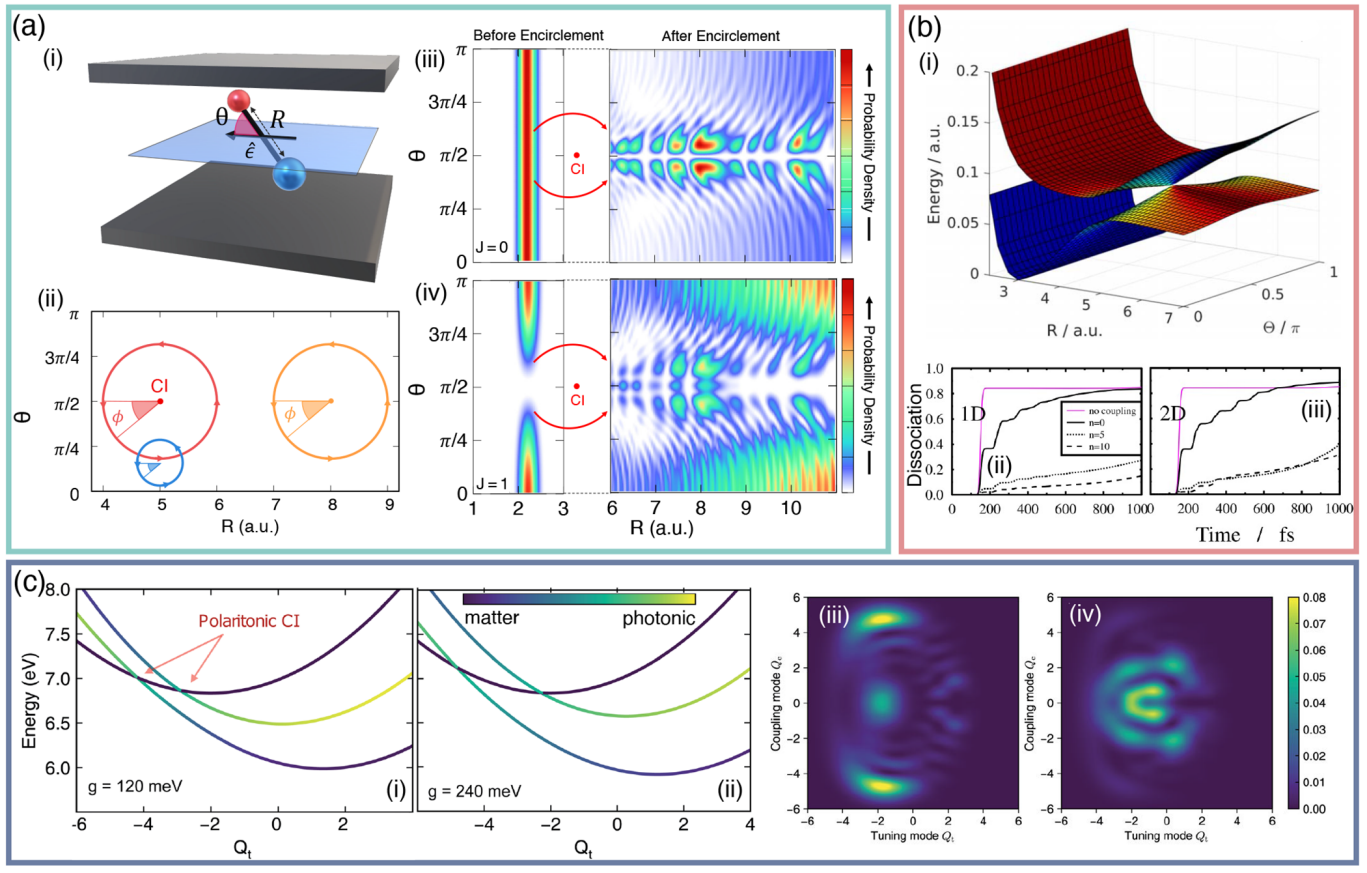
Cavity induced conical intersections. (a) (i) Diagram of a diatomic LiF molecule with bond length *R* inside a Fabry–Pérot cavity. The molecule has a transition dipole moment along the *R* axis which forms an angle θ with the cavity polarization vector ϵ̂. The molecule is free to rotate along this angular DOF which allows the magnitude of the light–matter coupling to change and thus creates a light-induced conical intersection (LICI). (ii) Phase-space plot of various nuclear encirclement paths (a full 2π rotation in ϕ around a point) for the LiF molecule. For paths that do not encircle the LICI (yellow, blue), no Berry phase is accumulated. For paths that do encircle the LICI (red), a nonzero Berry phase is accumulated. (iii) Nuclear probability density before (left) and after (right) encircling the LICI for the *J* = 0 angular momentum state. Note that a node is formed at θ = π/2 after encirclement. (iv) Same as (iii) but for the *J* = 1 angular momentum state. Note that the original node at θ = π/2 before encirclement has disappeared after encirclement. (b) (i) Potential energy surfaces of the upper and lower polariton for a LiF dissociation reaction coupled to a cavity. Note the LICI where the separation between the two surfaces vanishes. (ii) Population dynamics of the dissociated state when the molecule is prevented from rotating (hence “1D”). The different lines correspond to either the no light–matter coupling case or to strong coupling with different initial Fock states *n*. (iii) Same as (ii) but the molecule is allowed to rotate (hence “2D”). The population dynamics of the 2D case versus the 1D case become more different as the initial Fock state becomes larger. (c) (i) Potential energy surfaces for a cavity-coupled pyrazine molecule with two molecular excited surfaces that share an intrinsic CI. The coupling strength, in this case, is *ℏg*_c_ = 120 meV. The coupling to the cavity causes the original intrinsic CI to “split” into two polaritonic CIs (PICI). Two of the three excited surfaces have partial photonic character and both of these states form PICIs with the third molecular excited state. (ii) Same as (i) but for coupling strength *g*_c_ = 240 meV. The larger coupling has increased the Rabi splitting which causes the two partially photonic states to be further apart. Consequentially, the PICIs are at different locations and are further apart. This dependence of the CI position on the coupling strength is specific to PICIs while the position of LICIs does not depend on the light–matter coupling strength. (iii) Nuclear probability density of pyrazine outside the cavity. Note the lack of a node at coupling mode *Q*_c_ = 0 and tuning mode *Q*_t_ = −1. (iv) Same as (iii) but inside the cavity. The nuclear density is less spread out relative to outside the cavity. Additionally, a node has appeared at *Q*_c_ = 0 and *Q*_t_ = −1, indicating that the position of the original (intrinsic) CI outside the cavity has shifted to a new (polaritonic) CI position due to light–matter coupling inside the cavity. Panel (a) is adapted from ref (117) with permission from the PCCP Owner Societies. Panel (b) is adapted from ref (386) under the CC BY license. Panel (c) is adapted from ref (271) under the CC BY-NC license.

In order to understand how cavity-induced CIs could affect photochemical reactivity, a characteristic of CIs called the Berry phase^387^ (also known as the geometrical phase) should be discussed. There is some experimental evidence for the effect of the Berry phase inside certain bare optical cavities,^388,389^ but there is a lack of experimental evidence of the effects of the Berry phase (BP) on molecules inside optical cavities. Thus, it is important to understand the Berry phase’s predicted modification of nuclear density and population dynamics to both aid in experimental design and to explain the features of theoretical calculations of cavity-modified nuclear densities. In ref (117), these polariton induced BP effects (when coupling an LiF molecule with a cavity) are further demonstrated through the photofragment angular distribution, which can be measured with a state-of-the-art experimental set up of an intense laser coupling to molecules.^390^ For a diatomic system with a stretching coordinate *R* and a rotation angle, θ, relative to the cavity polarization vector (not be confused with the incident angle of photon used in Figure 4), the coordinates in the configuration space can be denoted as ***X*** ≡{*R*,θ}. Based on the JC model (eq 4), the upper and lower polariton states can be expressed as
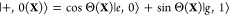
174
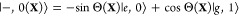
175with the mixing angle
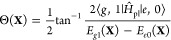
176where the coupling  = ·, the energies are *E*_g1_(**X**) = ⟨*g*,1|*Ĥ*_pl_|*g*,1⟩ and *E*_*e*__0_(**X**) = ⟨*e*,0|*Ĥ*_pl_|*e*,0⟩, and *Ĥ*_pl_ is defined in eq 106. The Berry phase^387,391^ is the change of sign of the electronic adiabatic wave function when the nuclei follow a closed path around the CI, which can be expressed as
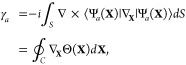
177where |Ψ_*a*_⟩ is a single valued polariton adiabatic wave function, and Θ(***X***) is the mixing angle (eq 176). The derivation of eq 177 can be found in ref (117). For a molecule inside a cavity, this Berry phase was analyzed in the work shown in Figure 16a, adapted from ref (117).

When a molecule can freely rotate inside a cavity, the angle θ between the dipole of the molecule **μ̂** and the cavity polarization **ê** will change and influence the strength of light–matter coupling (see Figure 16a(i)). At a particular bond distance *R* where the |*e*,0⟩ and |*g*,1⟩ surfaces intersect, a PICI is formed when the ground-to-excited transition dipole moment and cavity polarization vectors are orthogonal (θ = π/2). The nuclear path that takes a particular encirclement around this PICI in the {*R*,θ} configuration space (Figure 16a(ii), red path) will gain a phase of π on its adiabatic wave function |Ψ_*a*_⟩ after one full encirclement of the PICI based on eq 177. On the other hand, taking any other closed path that is not encircling the CI point will not add any additional phase to the wave function (Figure 16a(ii), blue or yellow paths).

The effect of this Berry phase can be seen in the probability density of nuclear wavepackets that pass through the PICI point. Note that even though we have used the JC model to intuitively explain the Berry phase effect of the PICI, the actual quantum dynamics simulation^117^ was performed using a numerically exact simulation to solve the full PF Hamiltonian (see eq 148). Figure 16a presents the PICI generated from coupling a LiF molecule with an optical cavity.^117^ For a nuclear wavepacket that is initially uniformly distributed in θ (Figure 16a(iii)) which corresponds to a rotational state with a quantum number *J* = 0, passing through the PICI from the left will cause half of the density to encircle the CI clockwise and the other half, counterclockwise. This causes the two halves to gain phases with opposite signs, causing interference effects when the wavepacket branches meet after the PICI point. These interference effects can be seen in the patterns of the probability density (Figure 16a(iii)) where, notably, a node at θ = π/2 appears after encirclement due to destructive interference from the Berry phase. Considering a different initial nuclear distribution with a rotational state *J* = 1 (Figure 16a(iv)) where the probability density has a much larger amplitude at the parallel (θ = 0) and antiparallel (θ = π) angles of the light–matter coupling, the probability density after encirclement has a lack of a node at θ = π/2 due to constructive interference of the Berry phase. These particular interference features are not consistently present if the Berry phase is removed from the dynamics or if the molecule is prevented from rotating.^117^ The presence of the Berry phase is thus an important feature of photochemical simulations involving conical intersections, and can also be experimentally observed when measuring the photofragment angular distribution (PAD) in a recent work of the light-induced conical intersection for a *H*_2_^+^ molecule coupled to an intense laser field.^390^ It is thus possible to experimentally test the effect of PICI by measuring the PAD, which is computed in ref (117).

The impact of cavity-induced conical intersections on photodissociation reactions inside optical cavities was also investigated in the work shown in Figure 16b, adapted from ref (386). In a photodissociation reaction of a LiF molecule coupled with the cavity, a PICI forms at the point when |*e*,0⟩ and |*g*,1⟩ surfaces cross and the angle θ between the transition dipole moment and cavity vector polarization is π/2 (Figure 16b(i)). To investigate the impact of the PICI (and the rotation dynamics as a whole), the dissociated population as a function of time was calculated both when including the rotational dynamics (Figure 16b(iii), referred to as the 2D model) and when fixing the angle with the cavity (Figure 16b(ii), referred to as 1D model). Further, the number of initial photonic excitations was varied to understand the effect of PICI. The dissociated population dynamics between the 1D and 2D scenarios were different, and this difference became larger for a larger number of initial photonic excitations in the cavity.^386^ This is because that for a larger photon number *n* (associated with the photonic Fock state |*n*⟩), the nonadiabatic coupling between the upper and lower polariton surfaces increases, thus making the effect of the PICI more pronounced (which is to quickly relax populations from the higher energy surface to the lower energy surface).

In addition to creating new conical intersections, coupling molecules that have an intrinsic electronic CI to cavities can split the original CI into two CIs, each having a mixed character of electronic excitation and photonic excitation, as described in the work shown in Figure 16c, adapted from ref (271). As shown in Figure 16c(i), when a model pyrazine molecule with an intrinsic CI between two molecular excited states is coupled to a cavity, such that one of the molecular excited states experiences light–matter coupling, two CI appear among the 3 excited state surfaces with properties different from those of either an intrinsic CI or an isolated PICI (Figure 16a). The locations of both CIs vary with the light–matter coupling strength, and as the light–matter coupling increases (from Figure 16c(i) to c(ii)), the distance between these two CIs also increases. This is in contrast to the individual PICI in Figure 16a whose location is independent of light–matter coupling strength. This feature of polariton-induced CIs allows for tunability of the CI position and thus a more flexible control over photochemical reactions that involve polariton-induced CIs.^271^ The geometric phase effects caused by these CIs can be seen in the nuclear probability density distribution in Figure 16c(iii) and (iv). The nuclear density is more spread out when outside the cavity (Figure 16c(iii)) than when coupled to the photonic mode inside the cavity (Figure 16c(iv)). This is consistent with the fact that the cavity coupling causes less energy to be stored in the vibrational modes and more to be stored in the cavity photonic mode.^271^ Additionally, the presence of light–matter coupling has pushed the polariton-induced CI closer toward the Franck–Condon position which enhances the Berry phase-induced destructive interference seen at the coupling nuclear coordinate *Q*_c_ = 0 (not to be confused with the cavity *q*_c_) in Figure 16c(iv). These effects ultimately influence the electronic-photonic population dynamics, allowing for cavity control of these molecular systems that contain intrinsic CIs.

As demonstrated by the theoretical works above, these cavity-induced CIs can play a major role in the dynamics of photochemical reactions. The features of enhanced nonadiabatic coupling and Berry phase offer new mechanisms for an optical cavity to control photochemical reactivity. With that said, more experimental work is needed to demonstrate clear evidence of these cavity-induced CI features and to verify the proposed theoretical mechanisms of how cavity-induced CIs can control photochemical reactivity. All of the above examples are considering a single molecule coupled to the cavity, whereas the possible collective effect^267^ of using PICIs for chemical reactivity will be discussed in Sec. 6.2.

### 4.6 Controlling Chemical Reactivity with Quantum Photon States

Aside from tuning the cavity frequency or light–matter coupling strength to control polariton photochemistry, one can take advantage of various initially prepared quantum mechanical states of the photon, such as Fock states, coherent states, or squeezed coherent states. Preparing and controlling these quantum mechanical states are mature techniques in the quantum optics community. These different initial states can have a strong influence on the subsequent dynamics and on how the system’s phase space is sampled.^107,352^

A single cavity photon mode can be described in a variety of representations. The two most common representations are the Fock basis, |*n*⟩, and the positional basis of the photon,^53,181^ |*q*_c_⟩ (see eq 43 for *q̂*_c_). While the Fock basis is most convenient when considering initial conditions of single Fock states, the positional basis is convenient when starting from two related types of states: coherent and squeezed-coherent states.^107,181^ The construction of these related states takes advantage of their property that they have the minimal position-momentum uncertainty as allowed by the Heisenberg uncertainty principle.

A coherent state (CS) is defined as^392,393^
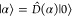
178where *D̂*(α) = *e*^*αâ*^†^–α^*^â^ = *e*^–|α|^2^/2^*e*^*αâ*^†^^*e*^–α^*^â^ is the displacement operator (analogous to eq 120 where the second equality comes from the Glauber formula) and |0⟩ is the vacuum state. By operating the displacement operator on the vacuum state |0⟩, the coherent state |α⟩ can be expressed as
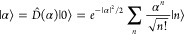
179

The parameter α is a dimensionless complex number that determines the displacement of the vacuum states expressed as follows
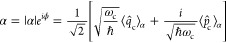
180and can be related to the expected value of photons in the cavity through ⟨⟩ = |α|^2^. The magnitude of the displacement is given by |α| and the phase ϕ determines the composition of the displacement in momentum and position space.

**Figure 17:**
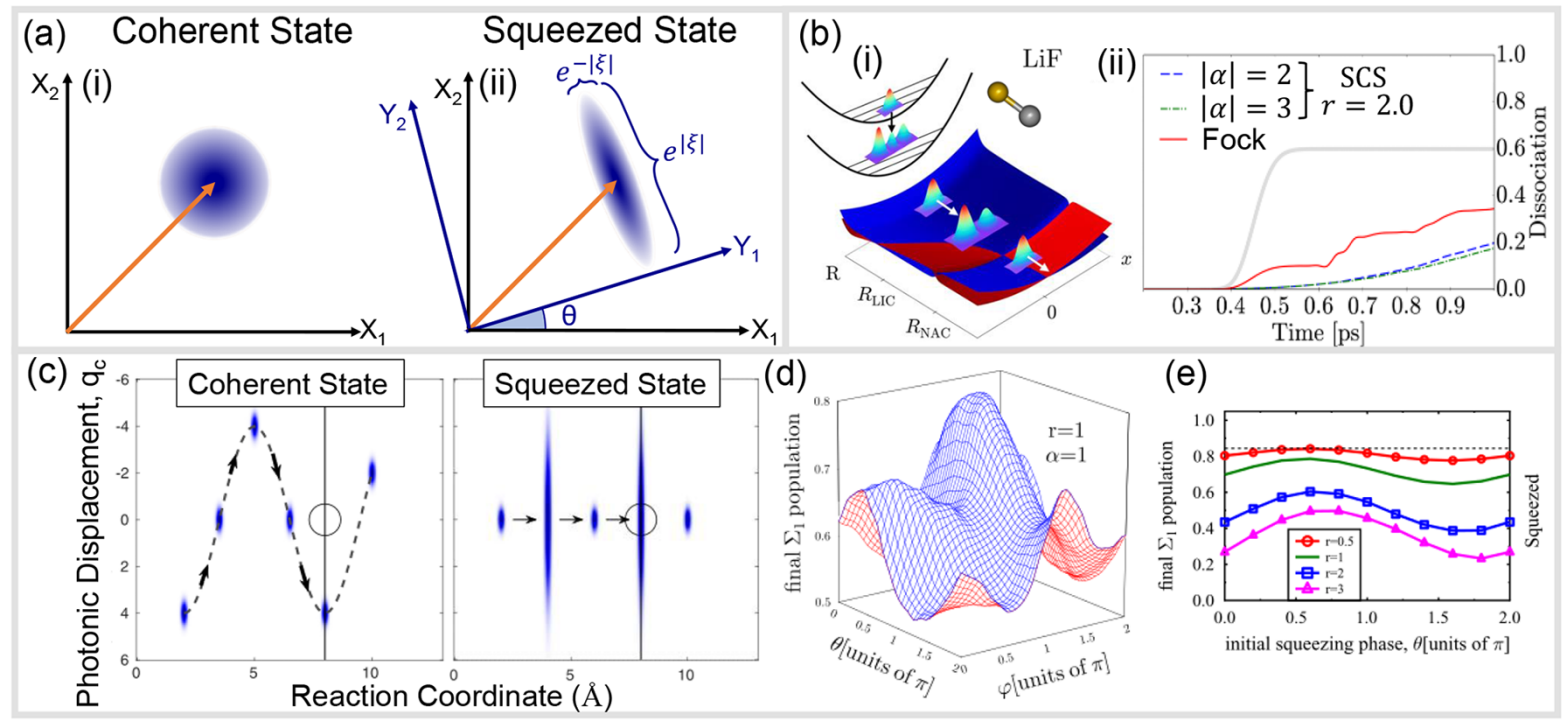
Photon mode initial state. (a) Phase space illustration of (i) a coherent state and (ii) a squeezed state with a squeezing parameter ξ = |ξ|*e*^*iθ*^, where the shaded areas depict the phase space area within one standard deviation of the expectation values. (i) Schematic of the dynamics on the polaritonic PES for a LiF molecule inside a cavity initialized with a quantum photon state. (ii) Dissociation as a function of time when the system is initialized with a Fock state (red), an SCS with |α| = 2 (blue), and an SCS with |α| = 3 (green) compared to outside the cavity (gray). (c) Time evolution (denoted by black arrow) of the photonic displacement for the polariton system of a LiF molecule in a cavity initialized with either a coherent state or a squeezed state. (d) Shows the final excited state population with a system initialized with a squeezed state as a function of the phase and, ϕ, and squeezing rotation, θ. (e) Shows the final excited state population with a system initialized with a squeezed state as a function of both *r* and θ compared to the free space limit (black). Panel (b) is adapted with permission from ref (107). Copyright 2018 American Chemical Society. Panels (c–e) are adapted with permission from ref (352). Copyright 2018 American Physical Society.

Further, the CS in the position space representation is given by
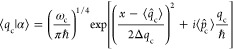
181where , , , and . In the phase space representation, this can be intuitively visualized as in Figure 17a(i), where the  and  values within one standard deviation of the expectation values are represented by the shaded circle. This shows how coherent states equally distribute the x-p uncertainty across *x̂* and *p̂*.

The squeezed-coherent state (SCS) “squeezes” the x-p uncertainty shared between *x̂* and *p̂* such that it still is the minimum *x* – *p* uncertainly allowed by the Heisenberg uncertainty principle. These states are defined as^392,394^
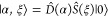
182where  is the squeezing operator with a squeezing parameter ξ = |ξ|*e*^*iθ*^ being a complex number. In the position representation, the SCS is expressed as
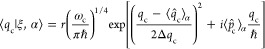
183where *r* = (cosh|ξ|+*e*^*iθ*^ sinh|ξ|)^−1/2^,  = , and ⟨*q̂*_*c*_⟩_α_ and ⟨*p̂*_*c*_⟩_α_ are the same expectation values as the corresponding coherent state with a displacement of α. While this representation can be difficult to parse at a first glance, additional intuitive insight is gained by looking at the distribution of SCS states in phase space as shown in Figure 17a(ii). As the SCS name implies, *Ŝ*(ξ) “squeezes” the probability distribution of the state in phase space. Instead of equal uncertainties in *X*_1_ and *X*_2_, now the distribution takes an elliptical form and is squeezed exponentially by |ξ| in a given direction. Additionally, the axes of this ellipse are rotated by the angle θ (not to be confused with the incident angle in Figure 4) such that the uncertainties are now squeezed in the *Y*_1_ and *Y*_2_ directions. This creates a more general class of minimal uncertainty states that redistribute the uncertainty across different pairs of observables (position and momentum, photon number and phase, etc.).

Recent theoretical works^107,352^ have demonstrated how starting from one of these minimal uncertainty quantum photon states inside a cavity can influence polariton photochemistry. In ref (107), the authors simulate the dynamical evolution of a LiF molecule strongly coupled to a cavity (see Figure 17b(i)). Specifically, the dissociation probability is calculated as a function of time. In Figure 17b(ii) they showed that by initializing the photonic state in a squeezed state (eq 183) and the molecule in the excited state |*e*⟩, thus having the tensor product state |*e*⟩⊗|α,ξ⟩ for the hybrid system, the dissociation pathway of the reaction can be suppressed relative to using |*e*⟩⊗|*n*⟩, which is a Fock state, as the initial cavity excitation.

ref (352)., similarly discusses how initializing in a squeezed state can affect polariton dynamics. In that work, a LiF molecular is coupled to a cavity, described by the quantum Rabi model Hamiltonian (eq 82). Figure 17c presents the dynamical progression (illustrated by the black arrows) of the polariton system in the position representation (with photonic coordinate *q*_c_ as the *y*-axis and nuclear coordinate *R* as the *x*-axis) when the initial photonic condition is set to be either a coherent state (left) or a squeezed state (right). The coherent state exhibits the standard oscillator behavior that is a “trademark” of these states. The squeezed coherent states, on the other hand, evolve in a “breathing” manner, where the expectation value of the photonic coordinate remains constant but the uncertainty oscillates. Figure 17d presents how the excited state final population changes as a function of the quantum phase term in α, ϕ, and the phase term of ξ, θ, for squeezed states of a constant *r* = |α| = 1. Further, Figure 17e presents that if α is held as a constant, varying *r* and θ can also dramatically change the excited state final population. These theoretical investigations^107,352^ with squeezed coherent states demonstrate how using these minimal uncertainty states (quantum photonic states) can affect polariton dynamics. In these examples, the reactivity of the LiF molecule changes due to the photonic state introduced in the cavity, showing how for a given cavity-molecule system the dynamics can be altered by introducing different photonic states. If can be realized experimentally, this will be a prime example of using tuning knobs in quantum optics to control chemistry.

### 4.7 Influence of Cavity Loss on Polariton Photochemistry

There can exist several sources of energetic relaxation in polariton systems. In additional to the typical dissipative sources in bare molecular systems such as vibrationally induced dissipations, molecules coupled to optical cavities also experience cavity loss. Many of the aforementioned works in polariton photochemistry have assumed that the optical cavity of study has a perfect internal reflectance with no loss of electromagnetic energy to the outside world. In reality, the photonic modes inside every optical cavity have some nonzero coupling with the photonic modes outside the cavity, which causes cavity loss to occur. This cavity loss reduces excitation energy in the molecule-cavity system and can have significant effects on the outcomes of polariton-mediated reactions. Thus, it is important to highlight the effects that cavity loss can have on simulations of polaritonic systems.

The starting point of a rigorous description of cavity loss is to describe the loss as an interaction of the cavity modes with an environment of external far-field photonic modes. The total Hamiltonian of a system plus its environment can be written as
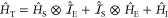
184where *Ĥ*_S_ is the system Hamiltonian,  is the identity in the system Hilbert space , *Ĥ*_E_ is the environment Hamiltonian,  is the identity in the environment Hilbert space , and *Ĥ*_I_ is the interaction Hamiltonian between the system and the environment. For cavity QED systems, *Ĥ*_S_ is the PF Hamiltonian *Ĥ*_PF_ (eq 104) while *Ĥ*_E_ describes the far-field photon modes as free bosons^397,398^
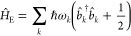
185where *b̂*_*k*_^†^ and *b̂_k_* are the raising and lowering operators, respectively, for far-field mode *k*. The interactions between the cavity mode and the far-field modes can be described by the Gardiner-Collett interaction Hamiltonian^397,399,400^ as
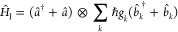
186where the coupling strength between the cavity mode and the *k*_th_ environmental mode is *g*_*k*_, characterized by a spectral density. This Hamiltonian can be rigorously derived from QED first-principles and has been used to investigate polariton quantum dynamics in a dissipative cavity.^398,401−404^

While there may exist some important non-Markovian effects caused by the explicit cavity-bath description, most often one is only concerned with the primary effect of cavity loss on the molecule-cavity system which is incoherent decay of excited population, which can be described using Markovian dynamics. As such, most discussions of cavity loss in the literature are based on the Lindblad master equation which is the most general description of the Markovian dynamics of open systems.^405,406^

The Lindblad master equation incorporates jump operators to describe the dissipative dynamical effects of the implicit bath. Most polariton literature up to this point that have described cavity loss with the Lindblad formalism have used the phenomenological jump operator^355^
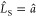
187to describe cavity loss. This jump operator is an approximation of the rigorously derived jump operators that describe jumps between the energy eigenstates of the system and include thermal effects. Regardless, the dynamics of the rigorously derived jump operators are typically well approximated by those of the phenomenological one for polariton systems.^402,403^

Using one of the single photon mode cavity QED Hamiltonians in Sec. Two as the system Hamiltonian *Ĥ*_S_ and the jump operator *L̂*_S_ in eq 187 to describe cavity loss, the Lindblad master equation for single mode polariton systems with cavity loss is as follows
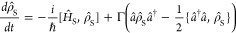
188where the anticommutator term  causes population decay as well as decoherence among states, whereas the *âρ̂*_S_*â*^†^ term (refilling term) makes the population reappear in the new state that the decay leads to (in this case, the state with one fewer photons). In order to make connections to other methods of propagating loss, the Lindblad master equation can be written in an equivalent form as
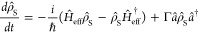
189where the effective Hamiltonian is
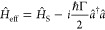
190

The expression in eq 189 has been used in the development of the stochastic Schrödinger equation^407−409^ which converges to Lindblad dynamics in the limit of large trajectory number. Alternatively, some recent works in cavity QED (refs (184, 312, 396, and 410−413)) have made the approximation to completely ignore the refiling term Γ**â*ρ̂*_S_*â*^†^ and approximate the Lindblad dynamics as the time-dependent Schrödinger equation (TDSE) with the complex Hamiltonian *Ĥ*_eff_. In situations where the refilling term is negligible, this approximation scheme matches the dynamics of the Lindblad master equation. However, when the refiling term is significant, the Lindblad dynamics must be included in full, either by propagating the density matrix or by using a stochastic wave function method.^395,407−409^

**Figure 18:**
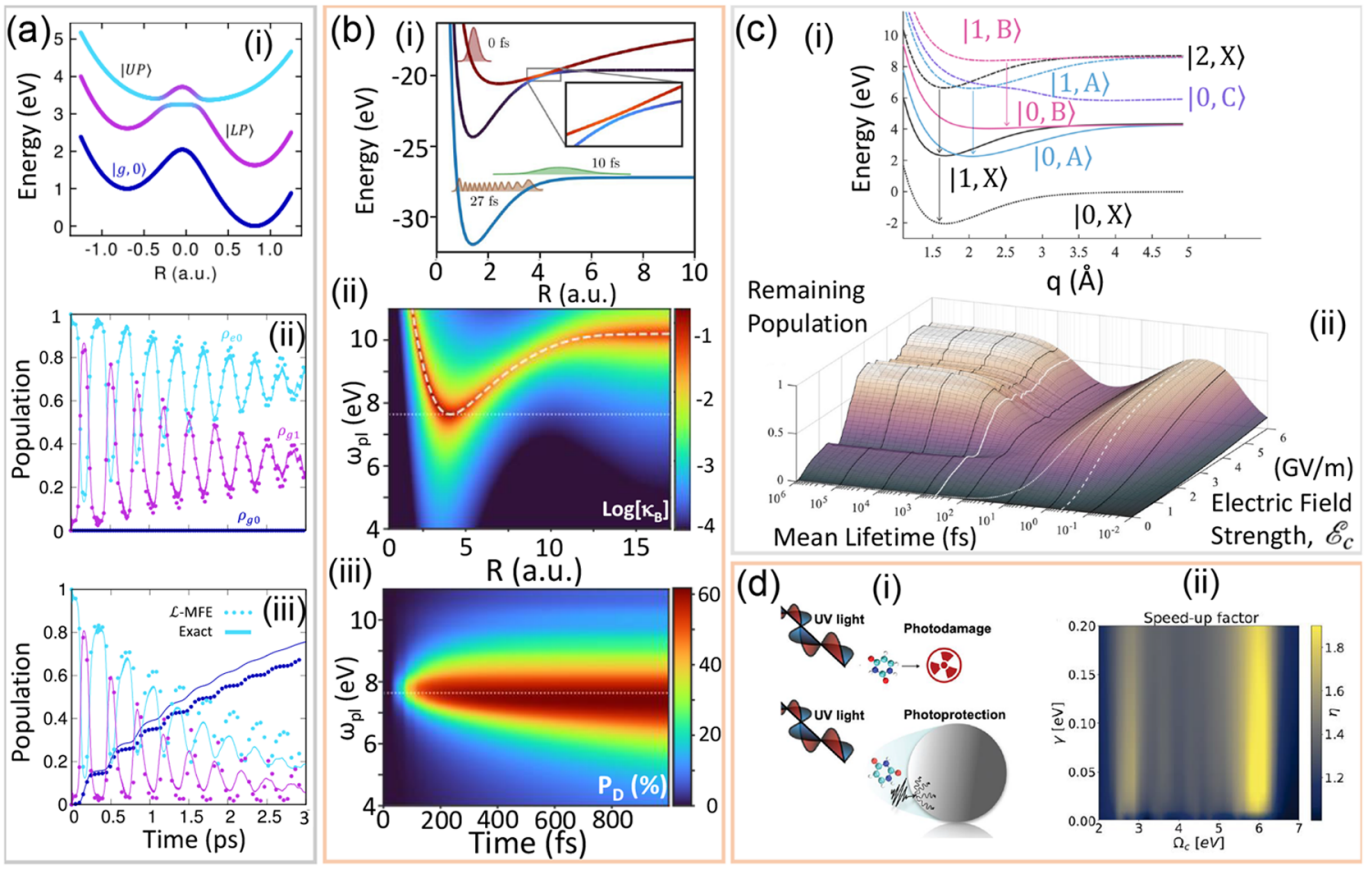
Cavity loss in polariton photochemistry. (a) (i) Polaritonic potential energy surfaces for an asymmetric isomerization model at the Jaynes-Cummings level with an uncoupled ground state |*g*, 0⟩ (dark blue). The cavity frequency is *ℏω*_c_ = 1.632684 eV with coupling strength *ℏg*_c_ = 0.136 eV. Purple color indicates molecular excited character while light blue indicates photonic character for the upper (UP) and lower (LP) polaritons. (ii) Population dynamics of the diabatic states (|*g*,0⟩ in dark blue, |*g*,1⟩ in magenta, |*e*,0⟩ in light blue) for a cavity without loss. The solid lines are exact quantum dynamics while the dotted lines are computed using the stochastic mixed quantum-classical -MFE method. The |*g*,0⟩ state does not become populated since it does not couple to the polariton states and there is no loss channel. (iii) Same as (ii) but with a cavity loss rate of κ = 1 meV. The |*g*,0⟩ state becomes populated due to the loss channel while both excited state populations (|*e*,0⟩ and |*g*,1⟩) loss population. Note that the |*g*,0⟩ state gains population at a higher rate when the |*g*,1⟩ state is more populated due to the use of the phenomenological jump operator *L̂* = *â*. (b) (i) Potential energy surfaces of the ground and polaritonic states. Representative examples of nuclear wavepackets at different times (0, 10, and 27 fs) are overlaid to demonstrate typical wavepacket behavior when cavity loss is present. Note that some of the nuclear density dissociates at later times which is not shown. (ii) Light–matter coupling-induced loss rate as a function of bond distance *R* and plasmonic cavity frequency ω_pl_. The loss rate is larger where the photonic |*g*,1⟩ state intersects the molecular |*e*,0⟩ state (white dashed line) and is maximized for the lowest cavity frequency that lets the diabatic states intersect (white dotted line). (iii) Dissociation probability as a function of time and ω_pl_. The dissociation probability is largest near the lowest cavity frequency that lets the diabatic states intersect (white dotted line). (c) (i) Potential energy surfaces of the diabatic states of a MgH^+^ molecule coupled to a cavity. There are multiple electronic states present (ground state |*X*⟩ and excited states |*A*⟩, |*B*⟩, and |*C*⟩) along with multiple Fock states ranging from *n* = 0 to *n* = 2 within the plotted range of energy. Cavity loss channels are shown as downward arrows, indicating several different possible paths for loss-induced population transfer to occur. (ii) The remaining population (not dissociated) at the steady state for a range of mean cavity lifetimes and electric field strengths _c_ (which is proportional to the light–matter coupling strength *g*_c_). The remaining population shows significant variability and nonmonotonicity over a wide range of lifetimes and coupling strengths. (d) (i) Diagram of a uracil molecule experiencing photodamage from UV irradiation outside a cavity (top) and being photoprotected by coupling to a plasmonic cavity (bottom). The photoprotection of the uracil molecule is caused by the photorelaxation induced by cavity coupling. (ii) The rate of relaxation from the excited state to the ground state inside the cavity relative to outside the cavity (speed-up factor η) for a range of Rabi splittings Ω_c_ and cavity loss rates γ. A higher speed-up factor allows for more photoprotection from UV photodamage. Panel (a) is adapted from ref (395) with permission. Copyright 2022 American Institute of Physics. Panel (b) is adapted from ref (355) with permission. Copyright 2021 American Institute of Physics. Panel (c) is adapted from ref (353) with permission. Copyright 2020 American Institute of Physics. Panel (d) is adapted from ref (396) under the CC-BY-NC-ND license.

The consequences of this cavity loss have been demonstrated in a number of works on polariton photochemistry.^70,257,312,353,355,380,395,396^ The most pronounced effect of cavity loss, the reduction of excited state population with photonic character, is demonstrated in Figure 18a, adapted from ref (395). Shown in Figure 18a(i), a model isomerization reaction in a perfect cavity (Figure 18a(ii)) undergoes its excited state dynamics while maintaining a total excited state population of 1.0. In contrast, when there is a nonzero cavity loss rate (Figure 18a(iii)), both the upper and lower polariton states lose population to the ground state. This loss of excited state population generally reduces the ability of a system to undergo reactions on excited surfaces. Consequentially, a significant cavity loss rate often, but not always, reduces the ability to enhance excited state reaction rates through light–matter coupling.

Cavity loss may also enhance the rate of photochemical reactions as demonstrated in Figure 18b(i), adapted from ref (355). In this H_2_ dissociation model, the molecular excited state has a broad potential well that resists photodissociation while the molecular ground state has a potential well that resists dissociation near the equilibrium bond distance but allows dissociation at farther nuclear configurations (Figure 18b(i)). With the presence of light–matter coupling and cavity loss, a nuclear wavepacket starting on the molecular excited surface can transfer to the |*g*, 1⟩ state and experience cavity loss to the ground state while maintaining the momentum in the direction of dissociation it gained while on the molecular excited surface. Afterward, part of this wavepacket can dissociate on the molecular ground state potential. As shown in Figure 18b(ii), the loss rate of the excited state due to coupling with the lossy photonic state is most pronounced where the two surfaces intersect and is maximal for the cavity frequency shown in Figure 18b(i) which corresponds to the dashed white line in Figure 18b(ii). This large loss rate along with the wavepacket dynamics mentioned previously showcases significant photodissociation probability (Figure 18b(iii)) when the cavity frequency is near the resonance point shown in Figure 18b(i), and is much smaller for other cavity detunings. This demonstrates the ability of cavity loss to take advantage of the curvatures of both the molecular ground and excited states to encourage a reaction that was resisted outside the cavity.

However, when multiple excitation manifolds are accessible, the effects of cavity loss become more complicated, as demonstrated by the work shown in Figure 18c (adapted from ref (353)). The potential energy surfaces considered (Figure 18c(i)) in this model of MgH^+^ coupled to a cavity span states with different numbers of excitations, including doubly excited states composed of a molecular excitation and a photonic excitation (states |1, *A*⟩ and |1, *B*⟩) or two photonic excitations (state |2, *X*⟩). These doubly excited states can undergo cavity loss (indicated by downward arrows) and incoherently transfer population to the singly excited manifold. The combination of these loss channels along with the multiple cavity-induced avoided crossings leads to nonmonotonic effects when the cavity loss rate or light–matter coupling strength are varied. The remaining nondissociated population after photoexcitation (Figure 18c(ii)) was found to be smaller with a cavity lifetime of 10 fs than with a cavity lifetime of 1 or 1000 fs. The remaining population did generally increase with larger electric field strength, but this was not always the case since there are multiple local maxima and minima in the remaining population for longer lifetimes above 1000 fs. These nuanced, nonmonotonic features highlight the importance of using detailed theoretical calculations to predict the optimal cavity parameters for controlling photochemical reactions.

Additionally, cavity loss may protect molecules from photodamage by altering the time the photoexcitation spends in a nuclear configuration prone to damage. In the work shown in Figure 18d, adapted from ref (396), a photorelaxation model is considered where a molecule is susceptible to photodamage when in a nuclear regime where intersystem crossing may occur (Figure 18d(i)). Outside the cavity, the photoexcitation has some probability to transfer to a conical intersection regime which allows relaxation and prevents photodamage. When this reaction is coupled to a lossy cavity, a speed-up of this relaxation occurs (Figure 18d(ii)) which enhances photoprotection. This speed-up is maximized at a particular Rabi splitting and a particular cavity loss rate. This result stands in contrast to the typical idea that a reaction rate change would be maximized or minimized at either very large or very small cavity loss rates.

The preceding discussion on the effects of cavity loss on photochemical reaction demonstrates that while cavity loss may sometimes be a hindrance to enhancing reactivity on polaritonic surfaces, it may also serve to improve the desired reactivity and even act as another tunable knob to control photochemical reactivity light–matter coupling.

## 5 Vibrational Strong Coupling and Ground State Chemical Kinetics in Infrared Cavities

Recent experiments^4,17,18,41,131,137^ have demonstrated that coupling molecular vibrations to quantized radiation modes inside an optical cavity can lead to enhancement^18,137^ or suppression^4,17,41,131^ of the rate constant for a reaction in the electronic ground state. Further, it has been shown that this vibrational strong coupling (VSC) regime can be leveraged to selectively break chemical bonds,^4^ thus effectively realizing mode-selective chemistry.^414,415^ Interestingly, such modifications of chemical reactions operate “in the dark”,^414^ requiring no external source of photons (laser excitation), unlike the polariton photochemistry experiments summarized in Figure 12. This new strategy in the VSC regime, if feasible, will allow one to bypass some intrinsic difficulties (such as intramolecular vibrational energy transfer) encountered in mode-selective chemistry that uses IR excitations to tune chemical reactivities,^416−419^ offering a paradigm-shift of synthetic chemistry through cavity-enabled bond-selective chemical transformations.^4,41,414,420^ On the other hand, recent experimental works have also reported possible discrepancies with negligible cavity modification to ground-state chemical kinetics.^19,20^

Currently, *there does not exist a satisfactory theory to explain these observed modified reactivities*, despite recent progress.^85,116,272,421−430^ In the following section, we will provide a comprehensive overview of the existing experimental and theoretical works that have attempted to solve the mysteries of vibrational polariton chemistry.

**Figure 19:**
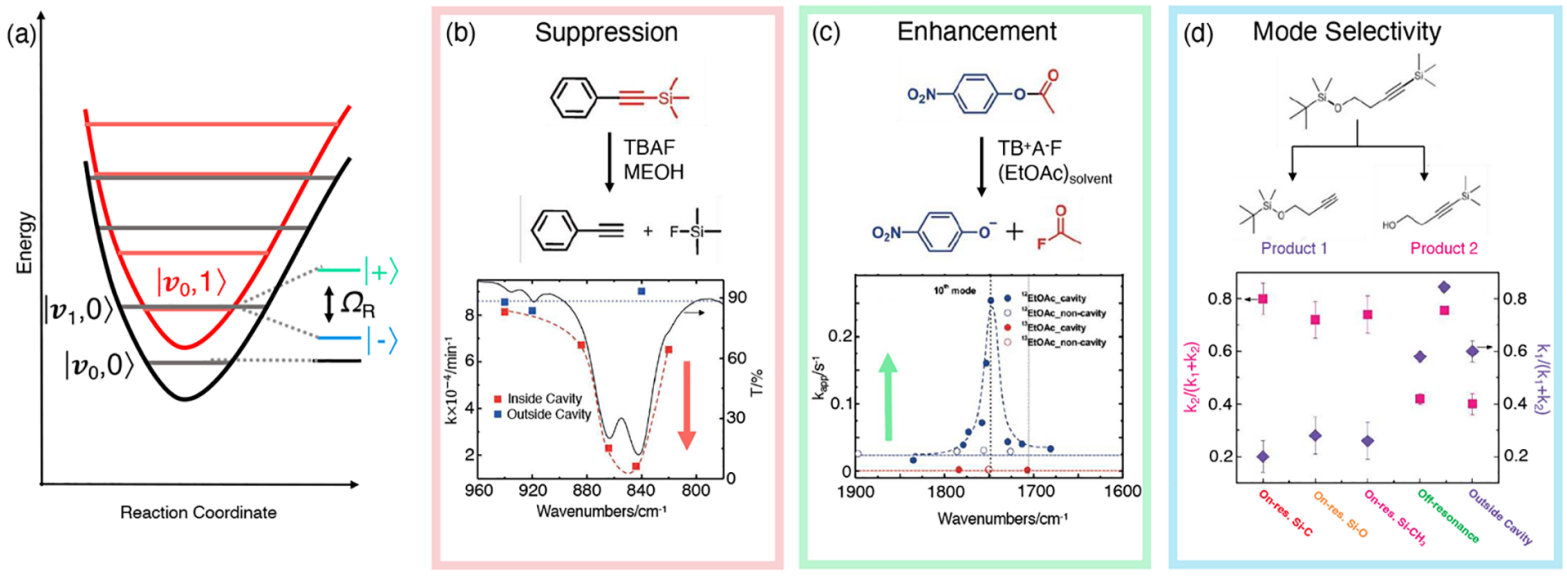
Forming vibrational polaritons and experimental observation of cavity modified of ground state chemical kinetics. (a) Schematic diagram of the formation of vibrational polaritons |±⟩ by hybridizing vibrational excited state |*v*_1_,0⟩ and cavity excited state |*v*_0_,1⟩. (b) Suppression of chemical kinetics: Chemical rate constant as a function of cavity photon frequency inside (red squares) and outside cavity (blue squares) and IR spectra of the molecule outside cavity (black solid line), the splitting should not be confused with the light–matter Rabi-Splitting. (c) Enhancement of chemical kinetics: Chemical rate constant as a function of cavity photon frequency inside (filled blue circles) and outside cavity (filled red circles). (d) Experimental demonstration of mode-selectivity inside the cavity for chemical reaction with two possible products (see top panel) labeled as 1 and 2. Pink and violet squares represent the relative yield of products 1 and 2 respectively. Panel (b) is reproduced with permission from ref (17). Copyright 2016 Wiley-VCH. Panel (c) is reproduced with permission from ref (18). Copyright 2016 Wiley-VCH. Panel (d) is reproduced with permission from ref (4). Copyright 2019 American Association for the Advancement of Science.

When molecular vibrational excitations are coupled to the optical cavity, one generates the vibrational polaritons, as illustrated in Figure 19a, where a vibrationally excited state with 0 photons in the cavity |*v*_1_,0⟩ (black energy levels) hybridizes with the ground vibrational state with 1 photon in the cavity |*v*_0_,1⟩ (red energy levels) that is in resonance to |*v*_1_, 0⟩. The resulting hybridized states |±⟩ (green and blue energy levels) that are energetically separated by the Rabi-splitting Ω_*R*_ (with details of the Rabi splitting provided in Section 5.1, eq 195). The Rabi-splitting is spectroscopically visible if it is larger than the rates of other competing dissipative processes (typically estimated from the spectral line-widths), such as solvent dissipation or cavity loss, and consequently, the light–matter coupling is said to be in the vibrational *strong* coupling regime. Note that this is only a schematic based on the JC type of model, where we only considered a single vibrational DOF coupled to a single cavity mode. In actual experiments, an estimated *N* = 10^6^ ∼ 10^10^ molecules are collectively coupled to the Fabry–Pérot cavity for each cavity mode.^45,46,128^

The interaction between many molecules and cavity radiation can be described using the Tavis-Cummings (TC) model (see Section 1.2) in the long-wavelength limit, which ignores the counter rotating terms (CRT) as well as the dipole self-energy (DSE) term. Meanwhile, the generalized TC (GTC) model can be used to capture the spatial variations of the cavity radiation. Note that the TC/GTC models break down in the ultrastrong coupling regime, as DSE and CRT become important.^132^ Assuming that the vibrations are perfect harmonic oscillators, the collective vibrational ultrastrong coupling regime can be described using the Hopfield model^150^ which can be analytically solved to obtain the spectra.^132^

**Figure 20:**
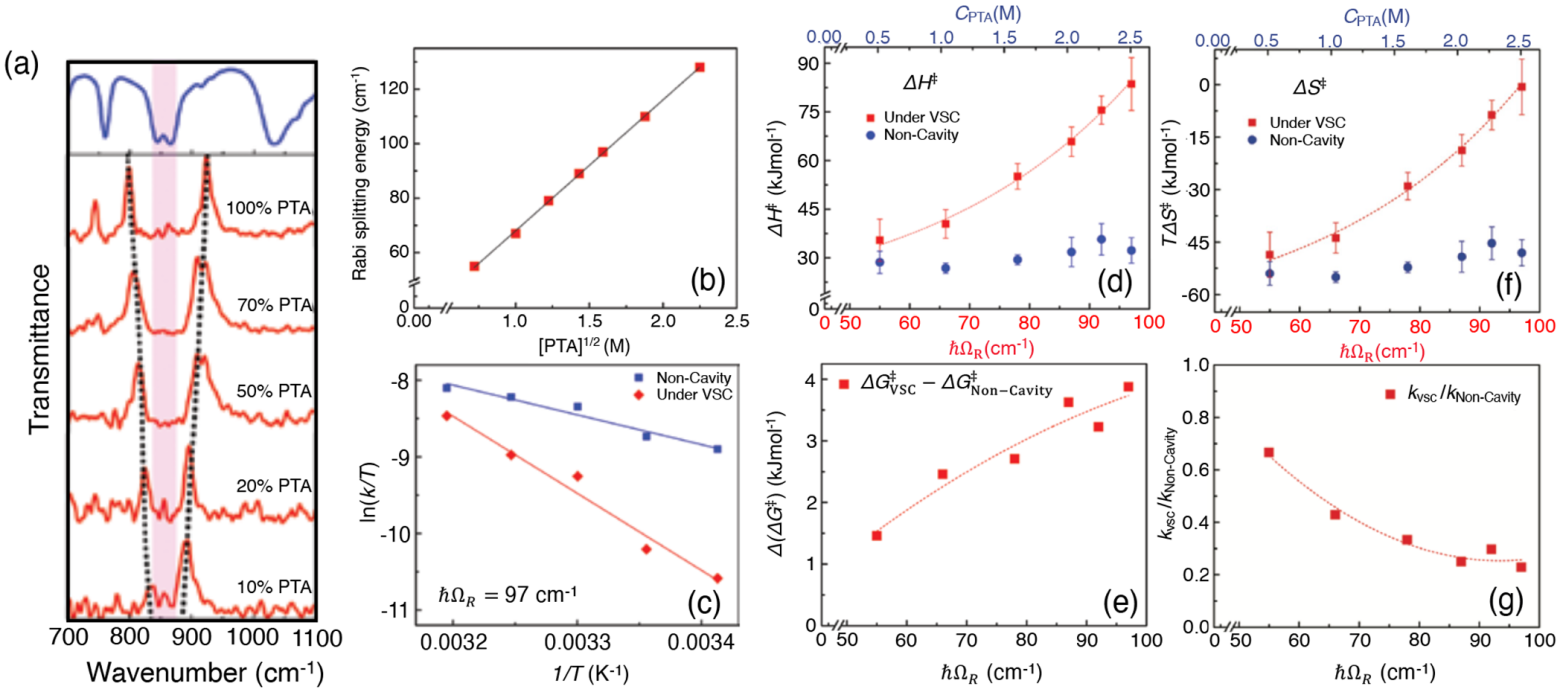
Modification of thermodynamic parameters under vibrational strong coupling. (a) Transmission spectra under vibrational strong coupling (VSC) showing Rabi-splitting Ω_*R*_ at various solute concentrations. (b) Linear increase in Ω_*R*_ as a function of the square root of the concentration. Chemical rate inside (under VSC) and outside (non-Cavity) cavity as a function of (c) temperature and (g) Rabi-splitting. Modification of thermodynamics parameters, (d) change in enthalpy Δ*H*^‡^, (f) entropy Δ*S*^‡^ and (e) modification to free-energy barrier Δ*G*^‡^ under VSC as a function of Rabi-splitting Ω_*R*_ compared to the noncavity scenario. This figure is reproduced from ref (41) under the CC BY license.

The vibrational strong coupling (VSC) regime has been achieved and the Rabi-splitting has been experimentally observed.^17,41,42,131,132,431^ As expected from the Tavis-Cummings (TC) model (see Section 1.2), the Rabi-splitting linearly increases with . This collective effect has been verified experimentally Figure 20a,b, where the Rabi splitting measured from the transmission spectra linearly depends on the  (see eq 17), which means linearly depends on the square root of concentration .

Figure 19b, adapted from ref (17), shows the IR spectrum (black solid line) outside the cavity (the splitting is an intrinsic molecular splitting and should not be confused with the Rabi-splitting due to the light–matter interaction). When the reaction indicated in panel (b) occurs inside an optical cavity, it was found^17^ that the ground state rate constant of the reaction (red squares and dashed line) is *suppressed* by 4–5 times, compared to the rate constant of the same reaction outside the cavity (blue squares). This suppression of the rate constant will only happen under the “resonant condition” when the cavity frequency is close (in resonance) with a molecular vibrational frequency.^4,17,131^ Specifically, in Figure 19b, the resonant vibrational frequency refers to the Si–C vibrational stretching frequency.^17^ Further, Figure 19b demonstrates the key features of this type of VSC experiment, with the width and shape of the transmission spectra (black solid line) being similar to the cavity-modified rate constant (red dashed line). Similar studies that have observed cavity suppression include ref (432) that studied Prins cyclization and ref (420) that very recently studied the urethane addition reaction.

Note that the experimental condition for the “resonant condition” is specifically referred to the case at the incident angle θ = 0, where the cavity frequency matches a particular vibrational frequency.^4,17,433^ This is indicated by the schematics in Figure 4b. The setup illustrated in Figure 4c, on the other hand, has a finite detuning between light and matter at θ = 0. Even though it also has a resonant condition (zero detuning) at some finite θ, there is no VSC modification of the rate constant observed experimentally for this case.^4^

Meanwhile, other experiments show a resonant enhancement of ground state chemical kinetics.^18,137^ For example, as shown in Figure 19c adapted from ref (18)., the reaction rate constant is enhanced and peaks at a maximum when the photon frequency is close to a solvent vibrational frequency (which is the C=O stretching frequency of the EtOAc solvent). On the other hand, a recent work^20^ has observed much smaller (≈1.5 times enhancement) rate enhancement for the same reaction under VSC, conflicting the results in ref (18). Interestingly, their results show that modification of the chemical reaction occurs for nonzero detunings.^20^

More interestingly, when there are two competing reaction pathways outside the cavity, it has been demonstrated that coupling them to the cavity can achieve mode-selective chemical reactivity.^4^ That is, the coupling of molecular vibrations to the cavity can selectively favor one chemical reaction over another, completely reverting the original selectivities compared to the situation outside the cavity. This mode selectivity is shown in Figure 19d, adapted from ref (4), where the yield of product 2 exceeds that of product 1 inside an optical cavity when tuning the cavity frequency to be resonant with a variety of bond frequencies. This is in contrast to the situation outside the cavity (or in the off-resonant scenario with a very large cavity frequency ω_c_ ≈ 5000 cm^–1^), where product 1 is formed more than product 2. In a similar experiment, the site-selective reaction of the aldehyde over the ketone in 4-acetylbenzaldehyde is achieved by automated cavity tuning to maintain optimal VSC of the ketone carbonyl stretch during the reaction.^434^

Experimental works have attempted to provide physical insights by computing the modification of thermodynamic parameters.^41,137^ Ref (41) investigates the desilylation of 1-phenyl-2-trimethylsilylacetylene (PTA), the same reaction studied in ref (131), which is shown in Figure 19, and extracts thermodynamic parameters assuming that the chemical rate is given by the transition state expression (Eyring theory)
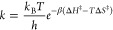
191

Based on the simple Eyring theory in eq 191, it gives
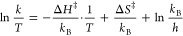
192

The effective cavity modification of reaction Entropy Δ*S*^‡^ and reaction Enthalpy Δ*H*^‡^ are extracted from the chemical rate constant *k*, measured experimentally. Figure 20c presents ln(*k*/*T*) as a function of 1/*T* under VSC and compares it to the noncavity scenario. The modification of the slope of ln(*k*/*T*) indicates that Δ*H*^‡^ is being modified (see eq 192) under VSC, and the changing of the *y*-intercept indicates that the reaction Entropy Δ*S*^‡^ is also modified (see eq 192) by VSC.

The modification of Δ*H*^‡^ and Δ*S*^‡^ under VSC as a function of Rabi-splitting Ω_R_ (due to the change of the concentration  in the experiment) is shown in Figure 20d and f, respectively. This analysis indicates that the free-energy barrier Δ*G*^‡^ = (Δ*H*^‡^ – *T*Δ*S*^‡^) is being modified under VSC inside the cavity, which is shown in Figure 20e that presents Δ(Δ*G*^‡^) = Δ*G*_c_^‡^ – Δ*G*_0_^‡^ as a function of Ω_R_ with corresponding chemical rate constant shown in Figure 20g. Interestingly, the chemical rate modification in Figure 20g shows a nonlinear relationship between Rabi-splitting Ω_R_ and rate constant *k*. Therefore, while Rabi-splitting is directly increases with , the modification of the chemical rate assumes a more complicated relationship. The full theoretical understanding and the physical origin of how cavity modifies Δ*S*^‡^, Δ*H*^‡^, and Δ*G*^‡^ remains unclear and is a subject of ongoing theoretical research. Note that if one hypothesizes that an unknown mechanism forces the upper or lower vibrational polariton states to be a gateway of VSC polaritonic chemical reaction,^435^ then the activation energy change should shift linearly^428^ with Ω_R_. The experimental results in Figure 20e, on the other hand, demonstrate a nonlinearity of reaction barrier.^41^Figures 19 and 20 summarize the basic features of the observed VSC modifications on chemical rate constants. Recent experiments also suggest that the symmetry of the vibrational normal mode coupled to the cavity mode also plays a role in modifying chemical reactivity^436^ and leads to the modification of stereoselectivity.^133^ Although it is not clear if the symmetry plays a key role in all VSC reactivities or just these specific ones.^133,436^

Recent theoretical investigations primarily aim to explain the following key features of the VSC-modified (adiabatic) ground-state chemical reaction. (i) cavity frequency dependence of the VSC-modified chemical rate: It is suggested that when the photon frequency is close (so-called *resonant* photon frequency) to some characteristic molecular vibrational frequency the chemical reaction kinetics is strongly modified. Meanwhile, when the photon frequency is far from these molecular vibrational frequencies (so-called *off-resonant* photon frequency) the chemical kinetics reduces to that of the cavity-free case. (ii) The collective regime of the VSC-modified reactivities: experimental studies that demonstrate cavity-modified ground-state chemical reactivity by coupling an ensemble of molecules to cavity photon modes. The Rabi-splitting that is formed due to collective light–matter coupling between molecular vibrations and cavity quantized radiation mode scales with , where *N* is the number of vibrational degrees of freedoms. It is suggested that the cavity modification of a chemical reaction also scales with . It is worth mentioning that for thermally activated *nonadiabatic* reactions both collective and resonant modification of chemical kinetics has been theoretically observed.^45,86,106^

In the following, we review several recent theoretical and computational works that have attempted to provide insights into cavity-modified ground-state chemical kinetics. In Section 5.1, we introduce the model Hamiltonian for the simplest scenario, a single molecule coupled to a single cavity photon mode. Section 5.2 shows why one-dimensional transition state theory (TST) predicts negative results of the VSC reactivities. In Sections 5.3 and 5.4, we review the Grote–Hynes rate theory^437,438^ that predicts a cavity frequency dependence for chemical reactivity,^14,85^ albeit with a much broader frequency dependency of the rate constant modification compared to experiments. Further, the GH theory predicts a maximum cavity modification of the rate constant occurring at photon frequencies close to the reaction barrier frequency, instead of some vibrational frequencies of the molecule which are actually observed in the experiments. This should be viewed as the major limitation of the GH theory when it is applied to the VSC regime. Then, in Section 5.5 we review recent works that demonstrate how cavities can resonantly enhance ground state chemical reactivity^421,423^ if the solvent–solute interactions are weak (such that the reaction is under the Kramers under-damped regime) and review the Pollak-Grabert-Hänggi classical rate theory that explains this phenomena. In light of the limited success that theoretical studies based on classical mechanics have had, we review works that investigate the importance of quantum effects in VSC modified chemistry.^272,429^ While (approximate) quantum corrections to the classical rate theories do not bring the theoretical prediction closer, exact quantum dynamical simulations performed *at the single molecule level*(272) show cavity modifications (both sharp suppression and enhancement) of chemical reactivity similar to the experiments. In Section 5.6, we review theories that show how IR-frequency cavities can modify ground-state nonadiabatic electron transfer reactions by directly coupling to the charge transfer transition dipole. In spite of the fact that all experimentally documented VSC-modified reaction rate constants fall under the regime of collective coupling, in this section, we will only review theoretical works that operate in the single-molecule limit. The progress of the collective coupling regime for VSC-modified reaction rate (which remains unresolved) will be discussed in Section 6.4.

We also recommend the readers for the following resources for further reading: Refs (1), (2), (433), and (22) reviewed recent experimental results of the VSC-modified reactivities. Refs (128) and (120) provide an overview of recent progress on the theoretical and computational developments in VSC-modified reaction rate constants.

### 5.1 Model Hamiltonian of Vibrational Strong Coupling

Recent theoretical works have focused on investigating a single molecule coupled to a cavity mode and try to obtain some insights into the VSC-modified reactivities (refs (85, 119, 193, 269, 272, 421, 423, 427, 429, and 430)). For simplicity, we assume that the direction of the dipole is always aligned with the cavity field polarization direction, such that **μ̂** · **ê** = μ̂. The universal light–matter Hamiltonian for this ground state reaction problem is given by  (eq 70) and using the projection operator that **only** includes the electronic ground state as , the projected light–matter Hamiltonian becomes
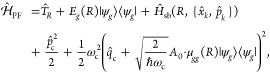
193where the first two terms describe a molecular system in terms of a reaction coordinate *R*, with a corresponding kinetic energy operator *T̂*_R_, and *E*_*g*_ (*R*) = ⟨*ψ*_*g*_|*Ĥ*_el_|*ψ*_*g*_⟩ is the ground state potential energy surface for the reaction coordinate *R*, with and a molecular ground state permanent dipole *μ*_*gg*_ (*R*) = ⟨*ψ*_*g*_|μ̂|*ψ*_*g*_⟩, see Section 2.1. Further, *E*_*g*_(*R*) takes the form of a harmonic potential near the reactant well *R*_0_, where  and ω_0_ is the reactant well frequency. Similarly, near the transition state configuration (R = *R*^‡^), *E*_*g*_(*R*) takes the form of a inverted harmonic potential, , where ω_‡_ is the barrier frequency and Δ*E*^‡^ = *E*_*g*_(*R*^‡^) – *E*_*g*_(*R*_0_) is the potential energy barrier. Most of the works reviewed here consider *E*_*g*_(*R*) to be a simple double-well potential^116,421^ or obtain it from a Shin-Metiu model.^14,85,178^

The *Ĥ*_sb_ term in eq 193 describes the system-bath coupling given by the Caldeira-Leggett model^439^
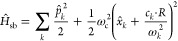
194which is the coupling between the reaction coordinate *R* and the dissipative bath with positions {*x̂*_*k*_} (such as solvent and other environmental DOFs). This system-bath coupling is characterized by the coupling constant *c*_*k*_ and frequency *ω*_*k*_, described by a spectral density . It should be noted that the Caldeira-Leggett system-bath model is only a crude model of the reaction coordinate coupling to its vibrational environment. A realistic description of molecular systems from ab initio simulations is a more desirable approach.^427^ The second line of eq 193 describes how a cavity photon mode *q̂*_c_ couples to matter through the matter dipole μ̂ which for the majority of this section is considered as the ground state permanent dipole that parametrically depends on *R*. Further, *p̂*_c_ and *q̂*_c_ are the cavity photon mode momentum and position operators, respectively, with a photon frequency ω_c_ and light–matter coupling strength *A*_0_.

In this model described by eq 193, the coupling between *q̂*_c_ and *R* creates a hybridization between the molecular vibrational states and photonic states, forming vibrational polariton states separated with a Rabi-splitting (Figure 19a). A simple expression for the Rabi-splitting can be obtained by considering the light–matter interaction term in  (eq 193) at the equilibrium position of the reactant, *R*_0_. At *R*_0_, we may approximate the permanent dipole as linear function of *R*, **μ**_*gg*_ (*R*) ≈ μ_0_ + μ_0_^’^´R, where μ(0) ≡ μ(*R*_0_) is the permanent dipole at the reactant well and  is the slope of dipole at the reactant well. While this linear-approximation is widely used in theoretical works investigating VSC mediated chemistry,^14,85,421,430,440^ new physical phenomena might emerge when moving beyond this approximation.^190^ On the other hand, direct molecular dynamics simulations of VSC mediated chemistry^423^ reveal the same physics as in other theoretical works employing the linear approximations.^421,430^ Further, previous works in model systems, where such linear approximations are tested, also show that this approximation is reasonable.^14,85^

The light–matter coupling term is then expressed as , which hybridizes the photon-dressed vibronic-Fock states |ν_0_,1⟩ ⊗|*ψ*_*g*_⟩ (photonic excitation) and |ν_1_,0⟩ ⊗|*ψ*_*g*_⟩ (vibrational excitation) causing a Rabi-splitting of *ℏ*Ω_*R*_ (under the resonant condition ω_c_ = ω_0_) of the form^85,431^
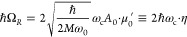
195where *M* is the reduced mass of the reaction coordinate *R* (the vibrational DOF that couple to the cavity), and the unitless coupling strength  characterizes the light–matter coupling strength. Note that to arrive at eq 195 we have used the fact that  =  = , where *b̂*^†^ and *b̂* are the creation and annihilation operators for the nuclear vibration associated with the coordinate *R*. Note that eq 195 is only valid for the single-molecule case, but the result can be generalized for *N* identical molecules {*R*_*i*_} coupling to *q̂*_c_. This is discussed in eq 220 of Section 6.4.

Looking at eq 193, the similarity between the vibration-phonon coupling (the second term) and the vibration-photon coupling (the third term) is apparent; for a linear permanent dipole *μ*_*gg*_(*R*) = μ_0_ · *R*, both second and third terms take the form of a typical Caldeira-Leggett system-bath Hamiltonian.^439^ Therefore, as much of the theories demonstrate, cavity modes act as additional *solvent* degrees of freedom providing fluctuations and dissipation to the reaction coordinate, resulting in the dynamical caging effect,^14,85^ or redistributing vibrational energy,^116,119,427^ hence leading to modifications of the reaction rate constant.

### 5.2 Simple Transition State Theory for VSC and its Limitation

Transition state theory can be employed by extracting the free energy barrier along the reaction coordinate, from the potential of mean force (PMF), *F*(*R*) that is defined as^372^
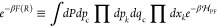
196using the classical limit of the Hamiltonian  (eq 193). Note that all phase space variables are integrated except the reaction coordinate *R*. The barrier along the PMF, Δ*F*^‡^ = *F*(*R*_‡_) – *F*(*R*_0_), is computed from its value at the reactant well, *R*_0_, and the barrier, *R*_‡_. The chemical rate using Δ*F*^‡^ within transition state theory is then written as,^372,438^
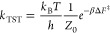
197

This TST expression effectively includes Entropic contribution from the other DOFs that are not *R* and should be more accurate than eq 198. Due to the quadratic form of the light–matter coupled Hamiltonian (eq 193), the free energy barrier (or equivalently the Δ*F*^‡^) is independent of cavity frequency, ω_c_, or light–matter coupling strength, *A*_0_.^428^ In ref (428), the PMF for a molecular reaction coordinate was computed for a cavity photon mode coupled to *N* noninteracting molecules. It was found that the free energy barrier extracted from the potential of mean force is not modified when coupling to the cavity and therefore no change in chemical rate due to cavity coupling is predicted.^428^ Thus, ref (428) concludes the VSC-modified reactivities can not be explained by TST.

Due to the harmonic system-bath interactions and the quadratic light–matter interactions in the Hamiltonian  in eq 193, the TST expression in eq 197 can be equivalently expressed as follows^438^
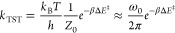
198where the ω_0_ is the reactant well frequency along the reaction coordinate, and the rate depends on the potential barrier height Δ*E*^‡^ = *E*_*g*_(*R*^‡^) – *E*_*g*_(*R*_0_) along the reaction coordinate. Further, in eq 198, the approximate expression is obtained in the classical limit for the partition function *Z*_0_, and it is assumed that solvent friction is nearly zero while thermal equilibrium in the reactant well persists at all times.^438^ The entropic contribution of the environment to the free energy barrier is set to zero as a result of this crude approximation.^438^ Therefore, for this simple one-dimensional transition state theory, cavity modification to chemical reactivity can occur only due to the modification of the barrier height Δ*E*^‡^.

When considering the cavity-molecule hybrid system, it is reasonable to examine the two-dimensional potential, so-called the cavity Born–Oppenheimer surface^181^
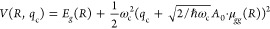
199which is  (see eq 193 for ). The energy barrier along the minimum energy path for the two-dimensional potential in eq 199 is unchanged in comparison to the original barrier Δ*E*^‡^ of the bare molecule (barrier along *R* in *E*_*g*_(*R*)). When the dipole self-energy (DSE, which is  in eq 199) is explicitly considered, *E*^‡^ remains invariant to changes of the light–matter coupling strength or the photon frequency. This is because the light–matter interaction Hamiltonian (eq 193 and eq 199) has a complete square of (*q*_c_ – *q*_c_^0^)^2^, and the stationary point along the photonic coordinate *q*_c_ is always  for all possible *R* (see ref (85) for details). As a result, Δ*E*^‡^ is not changed for *V*(*R*, *q*_c_), regardless of the magnitude of *A*_0_. Thus, *k*_TST_ is also independent of ω_c_ or *A*_0_ for the Hamiltonian in eq 193. Note that it is crucial to include the dipole self-energy term  for describing light–matter interactions inside a Fabry–Pérot cavity, without this term the barrier height and consequently the *k*_TST_ will be modified inside an optical cavity,^440,441^ which should be viewed as an artifact,^193^ at least for the FP type of cavity. On the other hand, the shape of the dipole can also play a role in determining the dynamics of the molecule in the absence of the dipole self-energy (DSE) term.^190^ However, these modifications will explicitly vanish when considering the DSE.^193^

We note that it has been argued for plasmonic cavities, the light–matter interaction Hamiltonian does not contain the DSE term.^442^ In ref (442), the authors state that the light–matter interaction in a plasmonic cavity setup originates from the Coulomb interaction between the molecule and the plasmonic nanoparticle, described by the pure longitudinal electromagnetic contribution (see eq 36). This implies that such a *light–matter* interaction term is not impacted by the PZW transformation and does not have an accompanying DSE term. In relation to this, ref (442) also pointed out an ambiguity in computing the DSE, where one arrives at two different expressions for the DSE depending on whether the PZW transformation is performed after or before the mode truncation. Ref (75) resolved this ambiguity of truncating cavity modes in the long-wavelength limit (similar results are found beyond the long-wavelength limit in refs (97 and 443)) and showed that it is appropriate to include the DSE term when considering a few energetically relevant cavity photon modes. At the same time, refs (82 and 157) argue that the response of the plasmonic nanoparticle due to its coupling to a molecular system will provide a DSE term (or a DSE-like quadratic term) that is necessary to describe a stable and physical system. Overall, the existence of the dipole self-energy (DSE) in the plasmonic light–matter coupling Hamiltonian remains an ongoing debate.^75,82,442^ On the other hand, the ground state potential of the coupled molecule-cavity hybrid system has been shown to be modified, even in the presence of DSE, when including electronic excited states^123,125^ but for high photon frequencies (in the UV regime) and is extensively discussed in Section 3.3.3. Thus, such theoretical treatments show neither resonance effect nor collective effect. In the next few sections, we will discuss theoretical works that have attempted to address these effects.

### 5.3 Dynamical Recrossing and Transmission Coefficients

The explicit dynamical interaction of the cavity DOF and the reaction coordinate should be taken into account explicitly, rather than integrating out as was done in ref (428) using *k*_TST_ (eq 197). Of course, the TST rate is only a very crude approximation of the rate constant, which explicitly assumes that once the reactive trajectory reaches the transition configuration, it will move forward to the product side (follow one direction) and no recrossing of the barrier nor turning back to the reactant side will occur. This is, of course, not accurate for reactions in the condensed phase where the solvent fluctuation can facilitate the reaction coordinate to recross the barrier many times before finally settling inside the product well.

A formally rigorous expression for the rate constant (under the classical limit of nuclei) can be written as
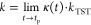
200where *t*_p_ refers to the plateau time of the flux-side correlation function, and κ(*t*) is the time-dependent transmission coefficient that captures the dynamical recrossing effects, measuring the ratio between the reaction rate and the TST rate. Since the classical κ(*t*) always starts from 1 and decays to a finite value (between 0 and 1) at *t*_p_, the *k*_TST_ is the upper limit of the actual rate constant *k*. Numerically, the transmission coefficient can be calculated from the flux-side correlation function formalism^444−446^ as follows
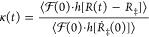
201where *h*[*R* – *R*_‡_] is the Heaviside function of the reaction coordinate *R*, with the dividing surface *R*_‡_ that separates the reactant and the product regions (for the model system studied here, *R*_‡_ = 0), the flux function  =  =  measures the reactive flux across the dividing surface (with δ(*R*) as the Dirac delta function), and ⟨···⟩ represents the canonical ensemble average (subject to a constraint on the dividing surface which is enforced by δ[*R*(*t*) – *R*_‡_] inside ). Thus, all degrees of freedoms (including the solvent and cavity photon modes) are sampled from the classical thermal distribution. Further, *Ṙ*_‡_ (0) represents the initial velocity of the nuclei on the dividing surface. The above flux-side formalism of the reaction rate can be derived from Onsager’s regression hypothesis, with derivations presented in standard textbooks (e.g., Chapter 8.3 in ref (446)). Numerical examples of κ can be found in refs (85) and (423) for the VSC problems.

Alternatively, κ can also be computed using the Grote–Hynes (GH) rate theory,^438,447^ or equivalently the multidimensional transition state theory (MTST),^448^ that treats all degrees of freedoms classically. Within GH theory, the transmission coefficient is given by
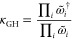
202such that *k* = κ_GH_ · *k*_TST_, where ω̃_*i*_^†^ are the frequencies associated with the stable normal modes (ω̃_*i*_^†2^ > 0) at the transition state geometry, and *ω̃*_*i*_ are normal-mode frequencies at the reactant well..^14,85,438^ The detailed expression of κ_GH_ for  (eq 193) can be found in ref (85).

### 5.4 Dynamical Caging Effect and Suppression of Rate Constant

In ref (85), it was theoretically demonstrated that the cavity photon mode acts as a *non-Markovian solvent-like degree of freedom* that is coupled to the molecular reaction coordinate *R*, such that the presence of photonic coordinate enhances the recrossing of the reaction coordinate and decreases chemical rate. In simple chemical processes and enzymatic catalysis, a closely related phenomenon is referred to as the “dynamical caging” effect,^449−453^ which has been well explained by the Grote–Hynes (GH) rate theory.^438,447,448^ Due to the low frequency of the cavity mode (in comparison to polariton photochemistry), which is in the same frequency range as the vibrational frequencies, both *R* and *q*_c_ are treated as classical DOFs in ref (85), and the GH theory is used to study how the cavity mode affects the dynamics of a reaction.^14,85,116^ We emphasize that treating the photonic coordinate *q*_c_ as a classical DOF is a drastic approximation, and we will discuss the quantum treatment in Sections 5.4.5 and 5.5.

**Figure 21:**
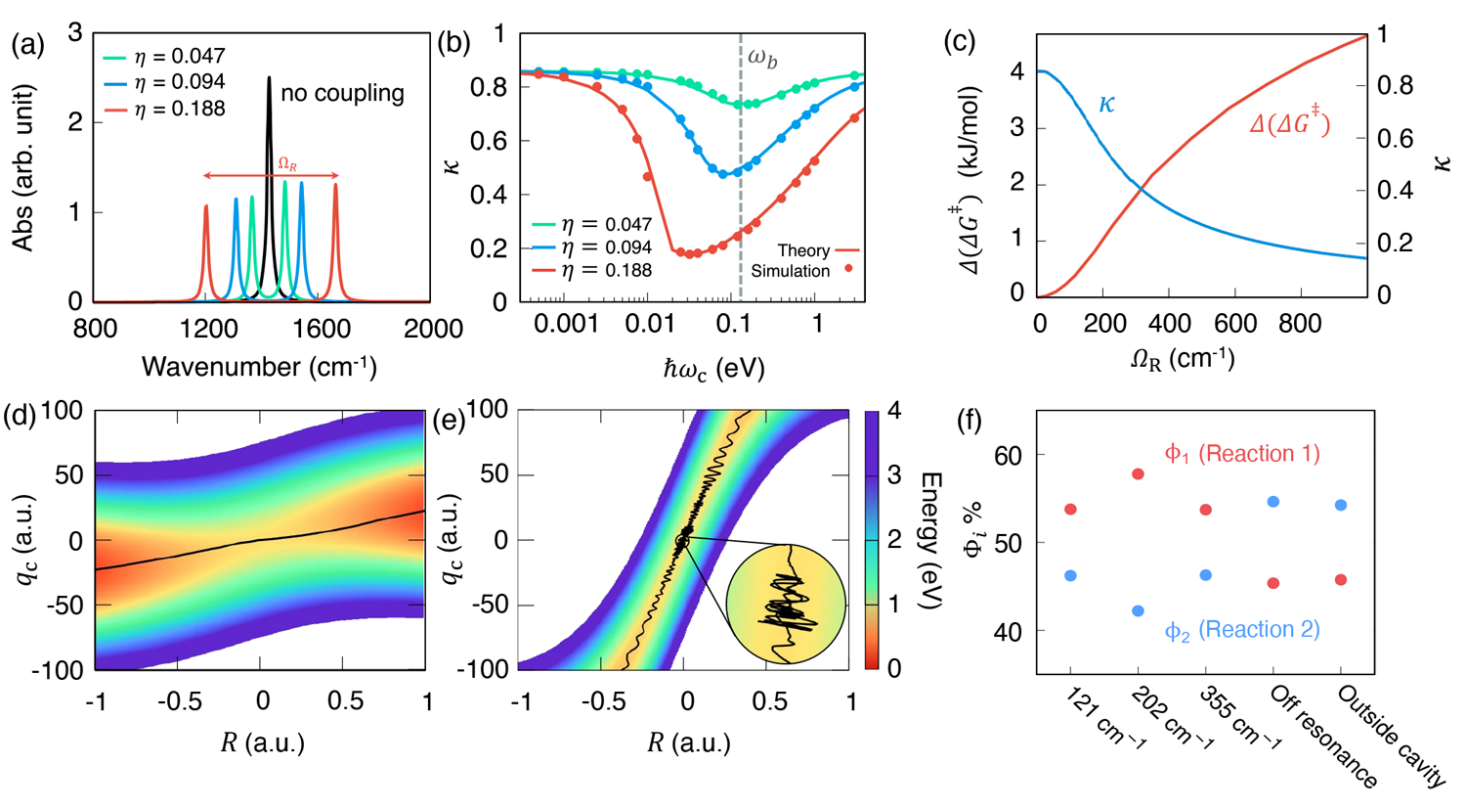
Cavity induced dynamical caging effect of the reaction coordinate and modification of ground state chemical kinetics. (a) Absorption spectrum of a model system at different light–matter coupling strengths. (b) Transmission coefficient κ as a function of the cavity frequency ω_c_ at different coupling strengths. (c) Transmission coefficient κ and the effective change of the Gibbs free energy barrier Δ(Δ*G*^‡^) at different Rabi splitting ω_R_, i.e., coupling strengths. The nonlinear change in Δ(Δ*G*^‡^) is similar to the experimental observation shown in Figure 20e. (d,e) Two-dimensional potential energy surface with respect to the molecular coordinate *R* and photonic coordinate *q*_c_ at small (d) and large (e) coupling strengths. The black solid lines represent typical reactive trajectories. (f) Percentage yield of two competing pathways at various cavity frequencies. Panels (a–e) are reproduced from ref (85) under the CC BY license. Panel (f) is reproduced with permission from ref (14). Copyright 2022 American Chemical Society.

In ref (85), such a classical description was employed to investigate cavity-modified ground state chemical rate for a single molecule coupled to a single cavity mode. The model system is described by  (eq 193), where the choice of *E*_*g*_(*R*) is the ground state potential of the Shin-Metiu hydrogen atom transfer model.^105^ The key results of these studies^14,85^ are summarized in Figure 21. Figure 21a presents the absorption spectra of the polariton system, where with an increased light–matter coupling strength η (eq 195), the Rabi splitting Ω_R_ also increases accordingly as observed in the absorption spectra. Figure 21b presents the transmission coefficient, κ, computed numerically using eq 201 (dots) or obtained using the GH theory through eq 202 (solid lines). Since *k*_TST_ remains invariant inside and outside the optical cavity, κ directly reports the absolute change of the overall rate constant (see eq 200). In this panel, we present the change of κ when the molecule is coupled inside the cavity, with a range of cavity photon frequency ω_c_. Three different light–matter coupling strengths, η = Ω_R_/2*ℏω*_c_, were chosen corresponding to the Rabi-Splitting observed in the absorption spectra shown in Figure 21a. The cavity modified transmission coefficient κ in Figure 21b clearly shows the cavity frequency-dependent suppression of chemical rate, which was not observed when only considering the TST level of theory^428^ or when ignoring DSE.^441^ At a fixed light–matter coupling strength *A*_0_, the transmission coefficient κ is minimized at a frequency ω_c_^min^ that is related to the imaginary barrier frequency ω^‡^. This effect can be physically understood as the cavity dynamically caging the reaction coordinate *R* near the barrier leading to a reduction in chemical rate.^85^ Further, one can also understand the significant red-shift of the ω_c_^min^, with the detailed theoretical explanation provided in ref (85). We emphasize that there are no existing experiments that report that matching cavity frequency ω_c_ with the top of the barrier frequency ω^‡^ will suppress the rate constant, under the single molecule limit. So even though there is a similarity between theory (Figure 21b) and experiment (Figure 19b), one must clearly understand that they are under very different coupling limits (single molecule for theory, and collective coupling for experiments) as well as at what photon frequency cavity most strongly modifies chemical reactivity. For the classical theories discussed above, it is when ω_c_ ≈ ω^‡^, and for experiments, it is ω_c_ ≈ ω_0_ (reactant well frequency). Nevertheless, the dynamical caging effect has also been observed in the ab initio VSC dynamics simulations^427^ of the reaction in Figure 19b. With the plasmonic cavity setup or the epsilon-near-zero cavity,^42^ it is possible to confine IR frequencies and even achieve an ultrastrong coupling regime for just a few (or a single) molecules.^454,455^ Thus, besides the purely theoretical value, the prediction in Figure 21 might also be within the reach of near-future experimental setups.

Figure 21c illustrates how the light–matter coupling modifies chemical reactivities. As one increases Ω_R_ (by increasing the light–matter coupling strength *A*_0_), the rate constant decreases in a nonlinear fashion which closely resembles the experimental trend in Figure 20g. For the PF Hamiltonian description that explicitly includes the DSE term, there is no change in *k*_TST_ because there is no change of potential energy barrier.^85^ The only change in the rate comes from κ. The modification of κ (formally κ contributed to the change in entropy^438^) will lead to the effective change of the free energy barrier height. To this end, we use the Eyring Rate equation to convert the change of rate from κ into an effective Δ(Δ*G*^‡^). The 4 times decrease in κ (blue curve in Figure 20c) results in ∼4 kJ/mol change in “effective” Δ(Δ*G*^‡^) (red curve in Figure 20c) at ∼700 cm^–1^ of Ω_R_. This theory indicates that such a nonlinear increase of the “effective” Δ(Δ*G*^‡^) as increasing Ω_R_ is in fact due to the change of κ.

To clearly demonstrate the dynamical caging effect, we further present representative reactive trajectory on the Cavity BO surface (eq 199). Figure 21d presents a typical nonadiabatic case of the GH theory. When the instantaneous friction is weak , the GH theory becomes a model of nonequilibrium solvation, where the friction from the photonic coordinate *q*_c_ does not severely impede the transitions.^456^ In this case, the transmission coefficient remains close to those without the cavity, and the reactive trajectory crosses the barrier without much influence from *q*_c_. Figure 21e presents a typical “dynamical caging” regime of the GH theory, where the instantaneous friction from *q*_c_ to *R* is strong , such that the reaction coordinate *R* becomes trapped in a narrow “solvent cage” on the barrier top.^456^ At longer times, the bath relaxations of *Ĥ*_sb_ (eq 194) allow the *R* to move away from the barrier top, but at shorter times, the reaction coordinate *R* oscillates within the cavity-induced “solvent” cage.^457^ The trajectory recrosses the dividing surface (*R*_‡_ = 0) many times, resulting in oscillations of κ(*t*) at a short time and with a small plateau value of κ(*t*) at *t*_p_. Similar dynamical caging effects from the solvent have been extensively studied in simple organic reactions (S_N_1 and S_N_2)^449,450,458^ and enzymatic reactions,^451−453^ where the solvent dynamics significantly influence the reaction rate constant.^438,456,459−461^ Here, the cavity photonic coordinate *q*_c_ acts like a “solvent coordinate”, and for strong couplings between *q*_c_ and *R*, the system exhibits the dynamical caging effect which effectively slows down the reaction rate constant.

In ref (14), this theoretical framework of dynamical caging was extended to two competing reactions coupled to the cavity, motivated to provide a theoretical explanation of the observed VSC mode-selectivity in Figure 19. In that work, two competing reaction pathways that have nearly identical barrier heights but different barrier frequencies are constructed as the model systems, both of which have their individual dipole that couples to a common cavity mode.^14^ The work finds that the dynamics of the cavity photon mode leads to a cavity frequency-dependent dynamical caging effect of a reaction coordinate, resulting in suppression of the rate constant. In the presence of competitive reactions, it is possible to preferentially (and selectively) cage a reaction coordinate when the cavity frequency matches one barrier frequency of two competing reactions, resulting in a **selective slow down** of the reaction between two highly competing ones.^14^Figure 21f presents several representative data points. In particular, it demonstrates that when using a high-frequency off-resonant cavity (ω_c_ is larger than all vibrational frequencies, such as ω_c_ > 1600 cm^–1^ in the current model), the selectivity is the same as the original selectivity without the cavity (effectively ω_c_ = 0). Further, the reverted preference occurs during *a range of cavity frequencies*, even though the maximum reduction of the rate constants for two competing reactions occurs at *two specific cavity frequencies*. These theoretical results provide a new perspective to understand the recent VSC enhanced selectivities of competing reactions, such as the results presented in ref (4) (see key results in Figure 19d). The results in Figure 21f closely resemble the basic feature of the experimental observation shown in Figure 19d.

Despite the similarities between the theoretical predictions in Figure 21 and the experimental observations in Figure 19, a number of significant differences must be noted. First, these theories suggest that κ is most strongly suppressed when the cavity frequency, ω_c_, is close to the barrier frequency, ω_‡_. This is in contrast to what the experiments suggest (such as in Figure 19b), where the chemical rate is strongly suppressed when photon frequency is close to the reactant well frequency. Second, the rate profile as a function of photon frequency is much broader, spanning several orders of cavity photon frequency. In contrast, experiments show sharp resonant cavity modifications, such as in Figure 19b. Third, these theoretical works^14,85,116^ that are based on the GH theory only predict cavity-mediated suppression, while the experiments also report enhancements (such as in Figure 19c). Finally, these works are studying cavity modifications at the level of single molecule and single cavity mode. This is in contrast to the experiments where an ensemble of molecules is coupled to a distribution of cavity photon modes.

Recent theoretical works^119,424,425,427^ have also explored dynamical effects related to intramolecular vibrational energy redistribution of the molecules coupled to an optical cavity. Ref (119) uses numerical simulations to investigate the dissociation dynamics of a triatomic molecular system (ozone) coupled to a cavity photon mode. Classical molecular dynamics is used to describe all degrees of freedom, including the nuclear DOF **R** and the photonic DOF *q*_c_. In this work, the dissociation dynamics were studied in the absence of a dissipative bath and by initiating the system in a nonthermal-equilibrium initial condition.^119^ Specifically, the cavity photon mode was initialized at zero temperature, while the molecular subsystem was initially deposited with enough energy (∼34 kcal/mol) to ensure that the dissociation of the ozone molecule takes place on a short time scale. It was found that when the cavity frequencies are close to vibrational modes, the “hot” molecular subsystem (with a high enough initial energy) efficiently exchanges energy with the “cold” cavity photon mode, leading to a suppression of the dissociation probability. While such a setup may not be representative of chemical kinetics in real molecular systems given its highly nonequilibrium initial state, it further illustrates the rich dynamical interplay between the cavity and molecular vibrations, which cannot be captured by static electronic structure calculations.

Similar conclusions have also been discovered from direct on-the-fly ab initio molecular dynamics simulations in ref (427). In this work, the deprotection reaction of 1-phenyl-2-trimethylsilylacetylene (PTA), experimentally studied in ref (17) (see Figure 19b) was investigated in the gas phase inside the cavity. The direct numerical simulations reveal that the cavity mode mediates the vibrational energy transfer between different vibrational modes, resulting in a shorter bond distance for the breaking bond during the reaction, thus in principle, suppressing the reaction. Interestingly, there is a resonant effect where the reactive bond distance will reach its minimum length when the cavity frequency matches the vibrational frequency of this bond. Future work is needed to investigate if such an effect still survives in the condensed phase (when considering the solvents) as well as if the bond shorting effect is equivalent to the reaction rate constant reduction.

#### 5.4.1 Quantum Corrections of the Rate Constant

Due to the initial success of the classical description of molecules interacting with cavity photon modes, the next natural question is how quantum effects (of the cavity mode or molecular vibrations) will influence the theoretical predictions. Along these lines, ref (429) attempted to add (approximate) quantum corrections to the GH rate theory to describe cavity-modified chemical kinetics. Two possible quantum corrections^429^ are added, including (i) replacing the classical partition function with their quantum counterpart using the quantum transition state theory (QTST)^462^ and (ii) adding tunneling effect using the formalism of the centroid TST (CTST).^463^ Using the QTST^429^ that only includes the quantum correction in (i), the total rate constant is written as
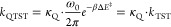
203where *k*_TST_ is expressed in eq 198, and κ_Q_ is the corresponding transmission coefficient. This transmission coefficient κ_Q_ = κ_ZPE_ · κ_S_ has two components, a zero-point energy (ZPE) correction (contributing to enthalpy) to the transmission coefficient κ_ZPE_, and an entropic component κ_S_ that depends on the normal-mode frequencies of reactant and the stable normal-mode frequencies at the transition state configuration. Under the high temperature limit, κ_S_ ≈ κ_GH_, and the κ_Q_ ≈ κ_GH_ (with κ_ZPE_ ≈ 1). Under the low temperature limit, κ_Q_ ≈ κ_ZPE_.

Based on the QTST formalism, ref (429) found that when the cavity photon frequency ω_c_ matches the reactant well frequency ω_0_ (i.e., a resonant condition), the ZPE correction κ_ZPE_ is minimized. This is in contrast to the high temperature (classical limit) where the GH theory predicts the transmission coefficient is independent of ω_0_, but depends on ω^‡^. Ref (429) further shows that it is possible to have chemical kinetics minimizing when ω_c_ is close to ω_0_ for specific sets of parameters when *k*_QTST_ is dominated by κ_ZPE_ (e.g., at low temperatures), and not dominated by κ_S_. But in general, the rate constant suppression will happen in a broad range of ω_c_, resulting in a much broader range of the photon frequency that suppresses the rate constant. This is in contrast to the sharp resonant behavior in experiments (see Figure 19). Meanwhile, this work,^429^ also shows that the additional quantum tunneling correction κ_T_ in the centroid TST (CTST) theory (where the rate is now *k* = κ_T_ · *k*_QTST_) is much larger than the ZPE correction κ_ZPE_. However, this tunneling correction minimizes the chemical rate when the photon frequency is close to the barrier frequency ω_‡_ in contrast to ω_0_, a behavior similar to the GH theory.^429^ As a result, the overall rate constant also minimizes when ω_c_ is close to ω_‡_. Overall, such quantum corrections, which are included through *approximate* rate theories, do not bring theoretical predictions closer, and potentially further, from experiments.

In ref (193), it has also been shown that when the DSE term is included, the cavity can only slightly modify the ZPE and bond lengths, but no obvious effects on dissociation energies and inversion barriers. When the quantum effects is considered, the reaction barrier will slightly decrease as the coupling strength increases. In a follow-up work,^269^ the authors further concluded that both the number of reactive channels and the tunneling probability will be reduced when the quantum effects are considered explicitly. It is also found that there is a coherent energy exchange between the system and cavity mode in the resonant case.

Finally, in ref (272), the chemical kinetics in a model molecular system coupled to a dissipative solvent bath and a lossy cavity mode was simulated with an *exact* quantum dynamics approach. It is found that the cavity can resonantly suppress the chemical reactivity of a molecular system that is strongly coupled to resonant solvent modes (i.e., sharp peaks in the solvent spectral density around reactant vibrational transitions). Such suppression occurs when the molecular vibrational states are split (through quantum light–matter interactions) further away from resonant solvent degrees of freedom, due to the formation of vibrational polaritons. This leads to a drastic reduction in molecule-solvent interactions. Since this particular mechanism relies on the formation of vibrational polaritons, the resonance condition between the cavity photon frequency and the vibrational frequency naturally appears.^272^ This work also showed that chemical reactivity can also be resonantly enhanced depending on the details of the molecule, solvent, and cavity. Overall, this work underscores the importance of the quantum dynamical interplay of solvent, molecules and cavity degrees of freedom.

### 5.5 Energy Diffusion and Enhancement of Rate constant

In the previous section, we reviewed theoretical works that attempted to explain resonant suppression of chemical reactivity. Importantly, some of the theoretical works suggest that the cavity plays a role in effectively modifying environmental friction. The works that used GH theory^14,85^ showed that the effective increase in environmental friction led to the suppression of chemical reactivity. The same argument can also be used to show that cavity modification to environmental friction leads to an enhancement of chemical reactivity if the solvent friction is much weaker in the energy diffusion-limited regime.^421,423,430^

**Figure 22:**
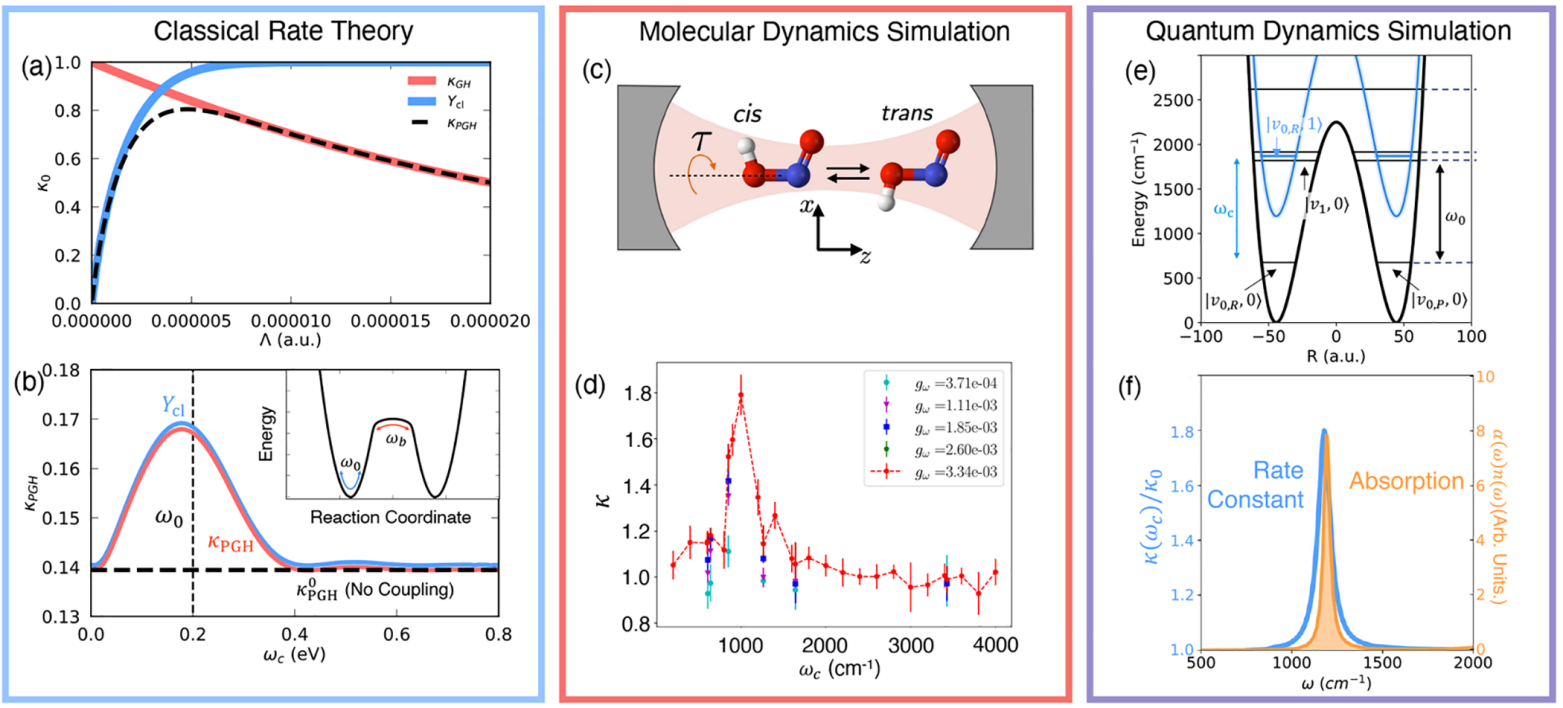
Cavity enhancement of ground state chemical kinetics. (a,b) Classical rate theory for cavity enhanced chemical reactivity. (a) The chemical transmission coefficient in bare molecular system (dashed solid lines) and the depopulation factor (blue solid line) as a function of solvent friction computed using the Pollak-Grabert-Hänggi rate (PGH) theory^464^ and the transmission coefficient within Grote–Hynes (GH) rate theory^438,447,448^ (red solid line). (b) Cavity photon frequency-dependent transmission coefficient (red solid line) and the depopulation factor (blue solid line). Inset shows the double well potential of the model system studied in (a,b). (c,d) Direct molecular dynamics simulation of cavity modification of the isomerization reaction in HONO, schematically illustrated in (c). (d) Cavity photon frequency-dependent transmission coefficient directly obtained from molecular dynamics simulations. (e,f) Exact quantum dynamics simulation of cavity enhancement in a model molecular system described with a double well potential shown in (e). (f) The chemical rate constant as a function of photon frequency was obtained from exact quantum dynamics simulations and compared with the absorption spectra of the molecule-cavity hybrid systems. Panels (a,b) are reproduced with permission from ref (421). Copyright 2022 American Chemical Society. Panels (c,d) are reproduced with permission from ref (423). Copyright 2022 American Chemical Society. Panels (e,f) are reproduced from ref (272) under the CC BY license.

To understand this, consider again a model molecular system described by a double well potential such as in the inset of Figure 22b. The chemical reaction rate as a function of environmental reorganization energy (proportional to environmental friction) is computed using a classical treatment for all degrees of freedom. The reorganization energy Λ of a solvent is directly computed from the solvent spectral density *J*(ω) (see below eq 194) as . The resulting rate constant is presented in Figure 22a. The transmission coefficient (black dashed line in Figure 22a) shows two distinct regimes: for Λ < 5 × 10^–6^ the chemical rate increases with increasing Λ (so-called energy diffusion-limited regime) and for Λ > 5 × 10^–6^ the chemical rate decreases with increasing Λ (so-called spatial diffusion-limited regime). The transition from the energy to the spatial diffusion-limited regime around Λ ≈ 5 × 10^–6^ is referred to as the Kramers turnover.^438^

Within the classical rate theory, the cavity photon mode is regarded as an additional environmental degree of freedom^85^ which increases the effective environmental friction. Thus, depending on whether the solvent friction is in the energy or spatial diffusion-limited regime the cavity mode is expected to enhance or suppress chemical reaction rates, respectively.^116,421,423^ However, in order to capture this cavity-modified enhancement of chemical kinetics, one must go beyond the GH theory which does not capture the energy diffusion-limited regime, as shown in Figure 22a where the *κ*_*GH*_ (red solid line) diverges from the true transmission coefficient (black dashed line) at low Λ.

In ref (421)., an analytical rate theory based on the Pollak-Grabert-Hänggi rate theory (PGH)^464^ was used to capture the complete range of solvent friction values, from the energy diffusion-limited to the spatial diffusion-limited regimes. Within the PGH theory, the reaction rate constant is given as
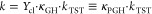
204where κ_PGH_ is the transmission coefficient within the PGH theory and *Y*_cl_ is the classical depopulation factor that accounts for the finite time for the reaction coordinate to reach thermal equilibrium in the energy diffusion-limited regime. In the spatial diffusion-limited regime, *Y*_cl_ → 1. As a result, in the spatial diffusion-limited regime the classical rate predicted by the PGH theory becomes *k* → κ_GH_ · *k*_TST_ which is the same as in the GH theory. Using this theory, the cavity-induced enhancement of chemical rate was predicted in ref (421)., which was also demonstrated using direct numerical simulations^423^ based on the flux-side correlation function formalism (eq 201). It is found that when the cavity frequency, ω_c_, is in resonance with the reactant well frequency ω_0_, the cavity can considerably improve the thermalization of the molecular system in the energy diffusion-limited regime.^421,423,430^ This is directly reflected in the photon frequency dependence of the depopulation faction *Y*_cl_ shown in Figure 22b (blue solid line). The overall transmission coefficient κ_PGH_ (consequently the shape of the rate constant as a function of photon frequency ω_c_) shown in Figure 22b is dominated by *Y*_cl_ in the energy diffusion-limited regime. Importantly, the chemical rate shows a clear resonant structure, peaking when ω_c_ ≈ ω_0_, and the width of the “resonant rate constant enhancement profile” is much sharper than the cavity suppression of chemical reactivity shown in Figure 21 in the spatial diffusion-limited regime. Ref (421) further points out that the extent of the cavity chemical kinetics modification is also more substantial in the energy diffusion-limited regime than in the spatial diffusion-limited regime, which often results in an enhancement by a factor of 2–3 (with a *A*_0_ = 0.01 as the light–matter coupling).^421^

In ref (423), the same effect of cavity-enhanced chemical reactivity was investigated for the *cis*-*trans* isomerization of the HONO molecule, as schematically illustrated in Figure 22c. With direct molecular dynamics simulations, it is observed that when cavity photon frequency is resonant to the O–N stretching mode at 900–1000 cm^–1^ the chemical kinetics is enhanced (Figure 22d). This is because the O–N stretch is strongly coupled to the torsion coordinate,^423,465^ which is the reaction coordinate for this isomerization reaction. Using the same computational setup they also verified the predictions made in GH theory^85^ where the chemical rate is suppressed due to cavity coupling in the spatial diffusion-limited regime (see Section5.4).

It must be noted that, while it is presently not known if the VSC related experiments operate in the spatial or energy diffusion-limited regime, chemical reactions in the liquid phase are typically expected to take place in the spatial diffusion-limited regime (strong solvent friction regime, either the plateau regime or the overdamped Kramers regime), whereas those in the gas phase are expected to take place in the energy diffusion-limited regime (weak solvent friction, or under-damped Kramers regime). Therefore, even if one disregards the issue of collectivity, the results obtained in these works^421,423,430^ may not be directly relevant to the experiments that were conducted in the liquid phase.^18,137^ That said, as has been argued in ref (421), the energy-diffusion-limited regime is more prevalent than is commonly assumed for chemical kinetics in liquid solvents.^437,466−468^ It is also possible for chemical reactions to be energy-diffusion-limited even if the solvent friction is large as long as the bath degrees of freedom are slow.^464,469^ A straightforward way to answer this question is to perform direct molecular dynamics simulation to extract the solvent spectral density. Overall, at this point we simply do not know what the precise value of the solvent friction is or in which regime the solvent coupling places those systems studied experimentally.

Finally, in ref (272) exact quantum dynamics simulations, using the hierarchical equations of motion (HEOM) approach, were carried out for a model system depicted in Figure 22e. In this work, a reaction coordinate was coupled to a dissipative solvent environment, a cavity photon mode which is also coupled to a dissipative bath composed of far-field radiation modes describing cavity loss (see Section 4.7).

In this fully quantum mechanical treatment, the chemical kinetics process can be easily understood in terms of solvent-mediated population transfer between vibrational states. The molecular subsystem is initially prepared in the ground vibrational state |ν_0,*R*_, 0⟩ (here 0 denotes no photon in the cavity) on the left well of the potential energy surface (shown in Figure 22e). Outside the cavity, the ground vibrational state on the left reactant well |ν_0,*R*_, 0⟩ is thermally excited to the vibrationally excited states such as |ν_1_,0⟩. Then, following a vibrational relaxation from the vibrationally excited states to the ground vibrational state |ν_0,*P*_,0⟩ on the right (product) well, the forward reaction occurs. In the weak solvent coupling (energy diffusion-limited) regime, the chemical kinetics is dominated by the thermal excitation process. The thermal excitation due to cavity loss mediated by the coupling of cavity photon modes to other far-field (outside of cavity) modes leads to the creation of a photon inside the cavity which can be absorbed by the molecular subsystem leading to the vibrational excitation. Therefore, coupling to the cavity provides (in addition to the solvent-mediated thermal excitation |ν_0_,0⟩ → |ν_1_,0⟩ outside cavity) an additional pathway
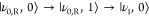
205which leads to an enhancement of chemical kinetics. Here, the first step is the thermal radiation fluctuation promoted transition and the second step is mediated by the quantum light–matter interactions. Since this mechanism requires strong hybridization between molecular vibrational and cavity photonic excitation (for the second step in eq 205), the resonance structure in rate constant modifications appears as a much sharper feature, shown in Figure 22f. Interestingly, the shape of the rate constant modification profile (blue) is similar to the absorption profile (yellow) in Figure 22f, which closely resembles the case in experiments,^18,137^ as illustrated in Figure 19. Importantly, this work reveals that the resonant cavity modification of chemical reactivity may have quantum origins. However, while this work underscores the limitations of the classical treatment in capturing VSC modified chemistry, it is not clear to what degree the cavity radiation has to be treated quantum mechanically as it may be possible that the semiclassical description photons (such as ring-polymer molecular dynamics,^86^ mixed quantum-classical dynamics,^74,83^ linearized path-integral model^283^) might provide accurate results when compared to the exact ones. Further, note that ref (272) operates at the single molecule level. Since it is prohibitively expensive to carry out such exact quantum dynamics in the collective regime, the development of approximate quantum dynamics methods (see Section 4.1) that allow efficient quantum dynamics of a large ensemble of molecules coupled to cavity photons will be vital in resolving the mysteries of the vibrational polariton chemistry.

### 5.6 Modifying Ground-State Electron Transfer Reactions

**Figure 23:**
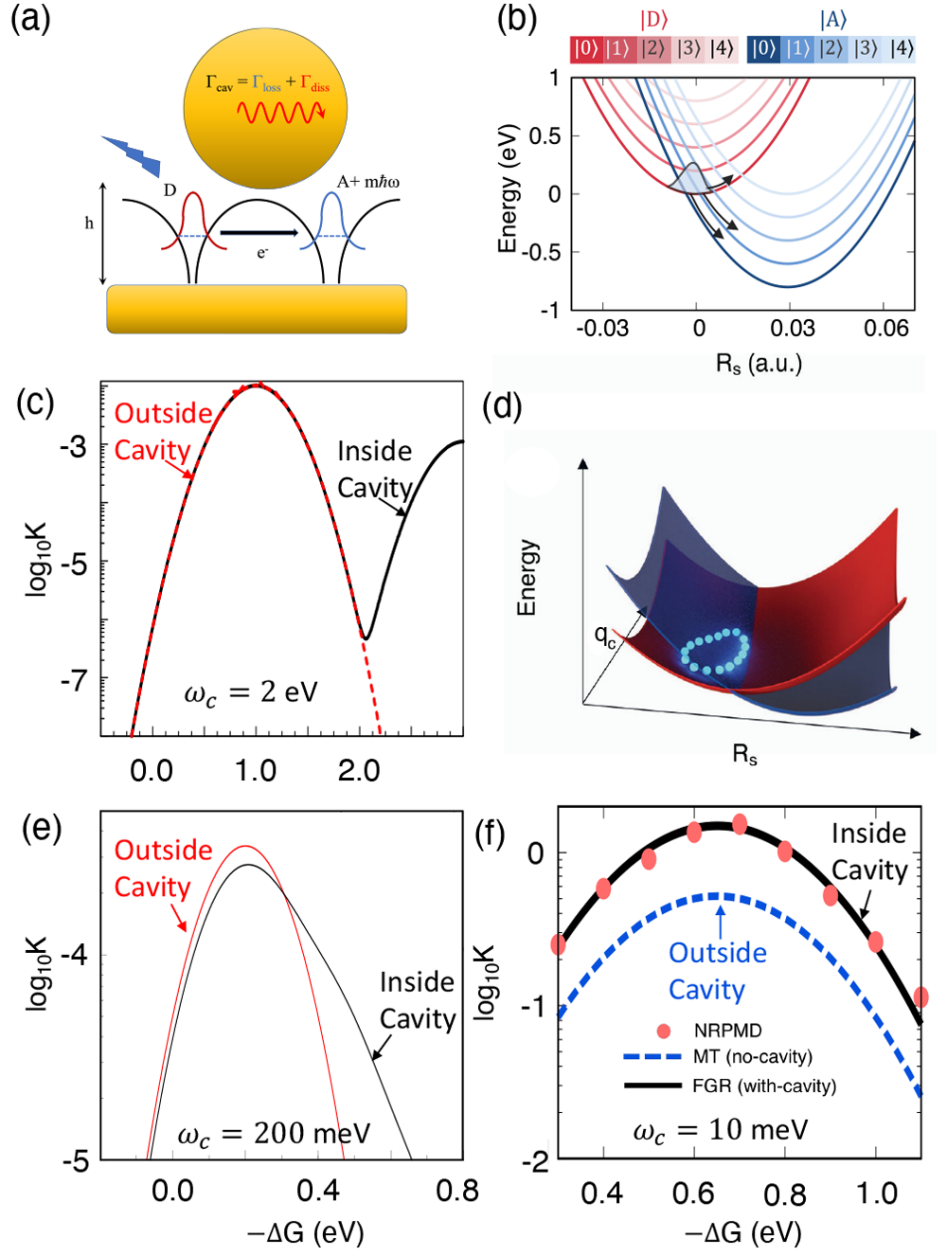
Cavity modified thermally activated nonadiabatic electron transfer reaction. (a) Schematic illustration of a plasmonic cavity coupling to an electron transfer reaction. (b) Potential energy surface of donor and acceptor dressed states. (c), (e,f) Cavity modification of electron transfer rate at (c) ω_c_ = 2 eV, (e) ω_c_ = 0.2 eV and at (f) ω_c_ = 0.01 eV. (d) Schematic illustration of the ring-polymer description of a cavity photon mode. Panels (a, c, and e) are reproduced with permission from ref (106). Copyright 2019 American Institute of Physics. Panels (b, d, and f) are reproduced with permission from ref (86). Copyright 2021 American Institute of Physics.

The chemical rate constant for nonadiabatic electron transfer reaction can be analytically computed using the Marcus theory^369,370,372^ as provided in eq 168. However, if the molecular system contains a *quantum* degree of freedom (such as a vibration with frequency *ℏω*_*v*_*β* ≫ 1), the rate constant requires a quantum description beyond the simple Marcus theory (especially in the inverted regime). This is because, with quantum degrees of freedom, the system can also access vibrational excited states for which the driving force is no longer just Δ*G* but is modified by *nℏω*_*v*_, where *n* is the quantum number of vibrations and *ω*_*v*_ is the vibrational frequency. The above situation is precisely the case when a nonadiabatic electron transfer reaction inside an optical cavity is considered. Such a setup is schematically shown in Figure 23a for plasmonic cavity. Note that the cavity frequency is either in the infrared regime or in the UV–vis region, but not matching any particular vibrational transition or electronic transitions. Instead, the cavity mode is directly coupled to the transition dipole of the charge transfer process^106^ between the |D⟩ and |A⟩ state, which is μ_DA_.

Due to the presence of the cavity photon mode, new photon-dressed donor and acceptor states, such as |D⟩ ⊗|*n*⟩ (donor state with *n* photons in the cavity) and |A⟩ ⊗|*n*⟩ (acceptor state with *n* photons in the cavity), become available for mediating the charge transfer process. The potential energy surface for these states along the charge transfer reaction coordinate (a collective solvent coordinate, which is often referred to as the Marcus coordinate) is shown in Figure 23b. The polariton-mediated electron transfer rate constant can then be computed by considering all possible reactive channels |D⟩⊗|*n*⟩ → |A⟩⊗|*m*⟩. The chemical rate constant in the presence of quantum degrees of freedom can be computed using the Marcus-Levich-Jortner (MLJ) theory^470,471^ as follows
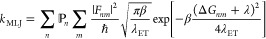
206where λ_ET_ is the reorganization energy (not to be confused with the light–matter coupling strength in eq 105), *F*_*nm*_ is the effective coupling among photon dressed states |D⟩⊗|*n*⟩ and |A⟩⊗|*m*⟩, Δ*G*_*nm*_ = Δ*G* + (*m* – *n*)*ℏω*_c_ is the driving force between photon-dressed states, and  = / is the thermal population of the corresponding cavity mode. Ref (106) investigated the modification of nonadiabatic chemical rate constant inside an optical cavity. In this work, the molecular system is coupled to the cavity photon mode via the molecular dipole μ̂ = μ_DD_ |D⟩⟨D| + μ_AA_ |A⟩⟨A| + μ_DA_ (|A⟩⟨D| + |A⟩⟨D|) where μ_AA_ and μ_DD_ are the permanent dipoles and μ_AD_ is the transition dipole. The authors^106^ (assuming that donor and acceptor wells are of the same frequency) derive the coupling  =  +  + , where  =  with Δμ = μ_DD_ – μ_AA_. In the absence of permanent dipoles *S*_*nm*_ → *δ*_*mn*_ and the coupling reduces to  =  +  + .

Figure 23c presents the modification of the ground state electron transfer rate when the molecule is coupled to a high-frequency photon mode (ω_c_ = 2 eV). Despite the fact that this example does not pertain to the regime of IR photons (or “VSC regime”), it clearly demonstrates the fundamental principles of such cavity modifications on electron transfer reaction rate constant. The red dashed line in Figure 23c depicts the rate constant as a function of driving force Δ*G* with one peak at Δ*G* = −λ_ET_, which is the famous Marcus turnover of the electron transfer rate constant. Inside the cavity, the chemical rate (black solid line) shows two peaks, instead of one. This additional peak that appears deep in the Marcus inverted regime (where – Δ*G* > λ_ET_) is due to the additional channel |D,0⟩ → |A,1⟩ due to the light–matter interaction via the electronic transition dipole moment μ_DA_ = ⟨D|μ̂|A⟩. For low photon frequency ω_c_ = 200 meV (panel b and panel e), the second peak becomes merged with the first and leads to a broadening of the overall rate profile shown in Figure 23e. In addition to this, suppression of the chemical reactivity is also observed around the peak of the rate curve. This suppression is due to the presence of the permanent dipoles^106^ that reduces the diabatic coupling between |D,0⟩ and |A,0⟩ by the factor *S*_*m*__,*n*_ as , a result that is obtained by performing the polaron transformation,^51,86,106^ but can also be understood through the polarized Fock state formalism (Section 3.1.3). Overall, this work^106^ points out that the cavity photon mode can act like high-frequency quantum vibration that modifies the nonadiabatic electron transfer rate constant, especially for driving forces in the inverted regime.

Ref (86) followed up on the work of ref (106), using an extended phase space path-integral framework,^472−474^ so-called nonadiabatic ring-polymer molecular dynamics (NRPMD),^475−477^ to describe discrete electronic states using mapping variables^321,322^ and the photon field and nuclear DOF using the extend phase space variables of the ring polymer. Using the RPMD framework, the photon and nuclear degrees of freedom are copied into multiple “beads” in the extended phase space, with the adjacent beads coupled through a Harmonic spring, forming a ring-polymer (shown schematically in Figure 23d). This ring polymer, together with the electronic mapping variables, is evolved classically through the corresponding equation of motion. Despite the classical evolution, NRPMD effectively captures all possible quantum effects, including the electronic nonadiabatic effect and the nuclear quantum effects, as well as similar effects exhibited by the photonic DOF *q*_c_. Ref (86) shows that even for a photon frequency as high as ω_c_ = 500 meV, the rate constant predicted by the direct NRPMD simulations provides the same result as the rates obtained from the analytic MLJ theory in eq 206 (that uses the quantum description of a cavity photon mode), which matches the analytic result perfectly for the model calculation presented in panel (c). Recent work^478^ has also used the adiabatic limit of RPMD (or referred to as path-integral MD) description of photon mode to perform molecular dynamics simulation using “real” molecular systems (using classical force fields) beyond any simple model systems. Regarding such development of semiclassical methods for accurately capturing such cavity-modified reactivities, ref (283) introduced a linearized semiclassical approximation with Fermi’s golden rule (FGR) rate theory, which can numerically reproduce the cavity-induced rate enhancement of such nonadiabatic electron transfer reactions with high accuracy.

Ref (86) also investigated the cavity modification of a nonadiabatic electron transfer reaction when photon frequency is very low, such that the classical description of the photon mode becomes accurate. With a classical treatment of the photon mode, the diabatic coupling becomes time-dependent and is a function of the photon coordinate, such that , In such a case, the photon mode plays the role of a Peierls coupling mode.^86,360,362,479^ For such fluctuating diabatic coupling the chemical rate is given by
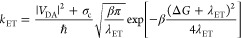
207where σ_c_ = 2ω_c_^3^*A*_0_*μ*_*DA*_⟨*q*_c_^2^⟩ with ⟨*q*_c_^2^⟩ = 1/*βω*_c_^2^ for classical distribution of the photon mode. Thus, the reaction rate is enhanced, (as shown in Figure 23f) when coupled to the cavity. This is due to the modification of the diabatic coupling due to the photonic mode serving as a fluctuating DOF that mediates the donor-to-acceptor coupling (commonly referred to as the Peierls coupling). It must be noted that while these works^86,106^ show the modification of the nonadiabatic electron transfer reactions for photon frequencies in the IR regime, the photon mode is coupled to the transition dipole between the donor and acceptor diabatic states (or a given permanent dipole), **not** any explicit vibrational excitation in the system.

In summary, despite enormous theoretical and experimental efforts, the mechanistic principles of VSC modified ground state chemical reactivities remain elusive. Theoretical and computational works do predict some modifications of the ground state chemical kinetics, and direct quantum dynamics simulations show photon frequency dependent rate profiles similar to the experiments. However, these results cannot be directly compared to experiments as they operate in different parameter regimes: while most theoretical works operate in the single/few molecule limit, the present experiments operate in the collective regime. In Section 6.4, we discuss some recent theoretical works that operate in the collective regime, which have had limited success so far. At the same time, there has been exciting experimental progress in achieving VSC at the level of few molecules.^42,480^ More concerted theoretical and experimental efforts are needed to realize the true potential of VSC mediated chemistry.

## 6 Polariton Chemistry under the Collective Coupling Regime

Most experiments of polaritonic systems involve many molecules coupled to many photonic modes in optical cavities. Although there have been exciting works demonstrating the possibility of strongly coupling a single molecule to a plasmonic cavity mode,^27,454,455,481^ it is understandably difficult (if not impossible) to achieve strong coupling in a Fabry–Pérot microcavity in the single molecule limit. This is because the relatively large cavity mode quantization volume in Fabry–Pérot microcavities leads to a negligible coupling for a single molecule.

When a large number of molecules are simultaneously coupled to the cavity, the effective coupling strength is scaled by  where *N* is the number of molecules (as will be discussed below). This collective coupling allows for significant Rabi-splitting despite the vanishingly small cavity coupling per molecule. Consequently, there has been a recent strong push by the community to better understand the collective coupling phenomenon from a rigorous theoretical perspective. In recent years, there have been a number of theoretical advancements that allow direct simulation of the quantum dynamics of a single cavity mode coupled to many molecules^8,45,116,126,256,313^ or many cavity modes coupled to many molecules.^138,141,177,179^

In the previous three sections of this review (see Sections 3, 4, and 5), the discussion has been focused on the properties, dynamics, and chemical transformations enabled by coupling a single molecule to a cavity mode. As we will see, the conclusions drawn in these previous sections that operate in the single molecule limit cannot be directly applied to the more experimentally relevant case of many molecules coupling to the quantized field inside an optical cavity. In this section, we will review recent theoretical works that investigate the modification of chemical and physical properties of matter in the collective coupling regime. In Section 6.1 we review computational works that study modifications to photophysical properties, such as absorption, photoluminescence, transport, decoherence, and population dynamics. In Section 6.3, we discuss theoretical works that show that charge transfer reactions can be modified in the collective coupling regime. Next, in Section 6.2, we review works that demonstrate the possibility of modifying chemical reactivity in the collective regime as well as works that provide conceptual insights on such processes. Finally, in Section 6.4, we discuss the unresolved mysteries of vibrational polariton chemistry in the collective regime and review some interesting works that have attempted to provide a resolution.

### 6.1 Polariton Photophysics in the Collective Coupling Regime

**Figure 24:**
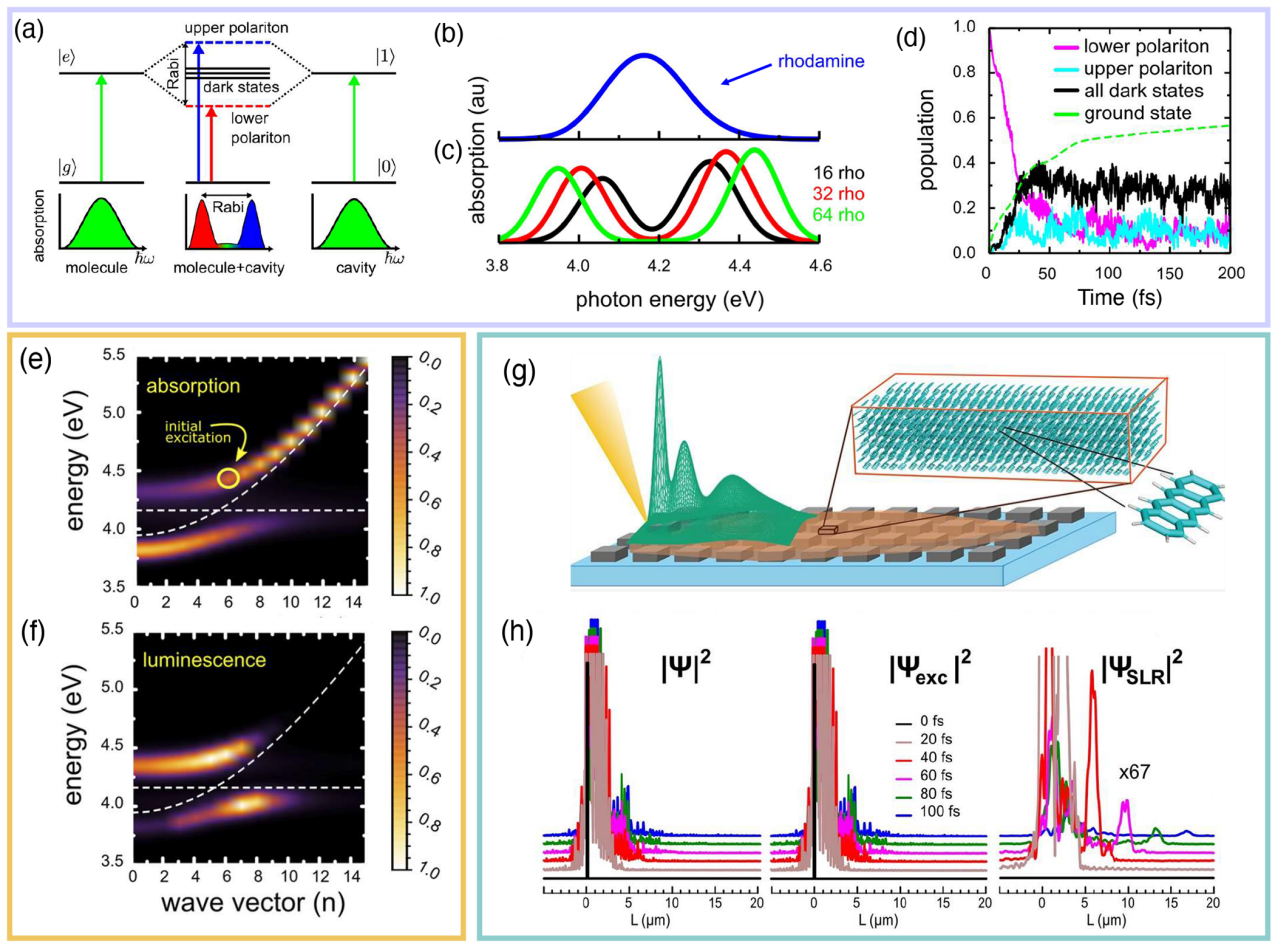
Modification of molecular photophysics in the collective coupling regime. (a) Schematic diagram of *N* identical emitters coupling to a cavity excitation leading to *N* – 1 dark states and 2 bright polariton states. Absorption spectra of (b) uncoupled molecules and (c) an ensemble of molecules coupled to the cavity. (d) Population dynamics of an ensemble of molecules coupled to a lossy cavity photonic mode. Simulated (e) absorption and (f) photoluminescence spectra for an ensemble of molecules coupled to several cavity photonic modes. (g) Schematic illustration of molecules coupled to plasmonic cavity arrays. (h) Time evolution of the polaritonic wavepacket and its excitonic and photonic components. Panel (a) is reproduced with permission from ref (313). Copyright 2017 American Chemical Society. Panels (b–d) are reproduced with permission from ref (126). Copyright 2019 American Chemical Society. Panels (e,f) are reproduced with permission from ref (138). Copyright 2021 American Institute of Physics. Panels (g,h) are reproduced with permission from ref (152). Copyright 2022 American Chemical Society.

In this section, we review theoretical works that shed light on interesting photophysical processes that are enabled or modified when coupling a large ensemble of molecules to one or more cavity photonic modes. An appealing simplified picture can be obtained by using the Tavis-Cummings Hamiltonian (see eq 12) for an ensemble of identical molecules coupled to a single cavity photon mode. In the Tavis-Cummings Hamiltonian, *N* singly excited molecular states {|*E*_*J*_, 0⟩ ≡|*g*_1_,···*e*_*J*_, *g*_*J*__+1_...⟩ ⊗|0⟩} (one molecule is excited while rest of the molecules are in their ground state with zero photons in the cavity) couples to the cavity excited state |*G*, 1⟩ ≡|*g*_1_, *g*_2_...⟩ ⊗|1⟩ (all molecules in their ground state with one photon in the cavity). Due to this coupling, a lower polariton, upper polariton, and *N* – 1 dark states are formed, as shown in Figure 24a. For identical molecules, the symmetry of the problem allows one to define the collective bright state  and other orthogonal states |*D*_*k*_,0⟩ = *∑*_*J*_*C*_*J*__,*k*_|*E*_*J*_,0⟩ such that . As a result, the bright state couples collectively to |*G*,1⟩ with a coupling strength  where *g* is the coupling between |*E*_*J*_,0⟩ and |*G*,1⟩. Notably, the |*D*_*k*_,0⟩ states do not couple to the |*G*, 1⟩ state, and thus are referred to as the dark states. The coupling  leads to the formation of the |+⟩ (upper polariton) and |–⟩ (lower polariton) states that are linear combinations of |*G*, 1⟩ and |*B*, 0⟩ (see more in eq 12 and onward) as depicted in Figure 24a. We note that the TC Hamiltonian is expected to break down in the ultrastrong coupling regime as it ignores the dipole self-energy and the counter rotating-wave terms. In addition to this the TC Hamiltonian also ignores the permanent dipoles, which might play an important role depending upon the molecular system under consideration.^51,354,482^

The formation of the polariton states |±⟩ is readily visible in the absorption spectra of the molecule-cavity hybrid system. Figure 24b presents the absorption spectra of the rhodamine molecules outside the cavity which peaks around the electronic transition |*G*⟩ → |*E*⟩. Due to the formation of |±⟩, the absorption spectra are split (Rabi-splitting) when coupled to a cavity as shown in Figure 24c, with the lower (upper) energy peak corresponding to the lower (upper) polariton. Figure 24c also shows the increase in Rabi-splitting as more molecules are coupled. Note that the dark states are not seen in the absorption spectra because they are optically dark.

This simplified picture, however, does not reveal the true complexity of the cavity-molecule hybrid systems. The molecular excitations are not truly degenerate in energy (thus the broadening of the absorption spectra in Figure 24b), and their energies fluctuate in time due to their dependence on the nuclear motion. The nuclear motion also induces nonadiabatic transitions between the upper polariton, lower polariton, and dark states, which in turn modify nuclear dynamics. Thus, direct dynamical simulations are an appealing approach to investigating the complex dynamical interplay between photons, molecular vibrations, and electronic degrees of freedom.

Ref (313) implemented a QM/MM excited state molecular dynamics approach to simulate a large ensemble of molecules coupled to a cavity photon mode. Specifically, they implemented the mean-field Ehrenfest approach, where the electronic and photonic degrees of freedom are treated quantum mechanically while the nuclear degrees of freedom are evolved classically. They used the Tavis-Cummings Hamiltonian (see eq 12), obtaining the energies and transition dipole matrix element for each individual molecule on a separate CPU/GPU in parallel, thus allowing them to perform large-scale excited state molecular dynamics inside an optical cavity.

Using the same on-the-fly quantum dynamics approach, ref (126) investigated the relaxation of strongly coupled molecule-cavity systems. In this work, the authors study ensembles of rhodamine molecules coupled to a single radiation mode. They find that the nonadiabatic transitions between the dark states, upper, and lower polaritons prolong the relaxation process of the excited molecule-cavity hybrid system. In an empty cavity, when the system is prepared in the |1⟩ (1 photon in the cavity) state, the cavity quickly relaxes to the vacuum states due to cavity loss (see details in Section 4.7). When the cavity-molecule hybrid system is prepared in the |±⟩ states, the photoemission rate is controlled by both the cavity loss (as they have significant photonic character) as well as the nonadiabatic transitions to the dark-state manifold. This is because the lower/upper polariton population is transiently transferred to the dark-state manifold, which does not have photonic contributions, thus suppressing cavity loss. This effect is shown in Figure 24d. After initial excitation to the lower polariton (pink solid line), fast relaxation to the ground state is observed at very short times. Then at ∼30 fs, a rise in the dark state population is observed and consequently, the relaxation to the ground state is suppressed marked with the ground state population rising at a slower rate at longer times, for the reasons mentioned before. This is in line with experimental works that show cavity-molecule hybrid systems having a much longer lifetime than the bare cavity.^129,483^

As mentioned in Section 2.6.2, a realistic description of the cavity radiation must account for photon dispersion. Ref (138) considers the photon dispersion and uses the generalized Tavis-Cummings model to simulate the photoexcited dynamics in an ensemble of molecules coupled to a distribution of cavity modes. The polariton dispersion is shown in Figure 24e which presents the absorption (or visibility) spectra of the multimolecule multicavity setup computed as^484^
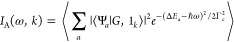
208where |Ψ_*a*_⟩ are the polariton states that are the eigenstate of the polariton Hamiltonian  where *T̂*_**R**_ is the nuclear kinetic energy operator, Δ*E*_*a*_ = *E*_*a*_ – *E*_*G*_ with *E*_*G*_ as the ground state energy of the light–matter hybrid system, Γ_c_ is a broadening parameter that accounts for various sources of dissipation such as cavity loss, and ⟨···⟩ represents the average over different nuclear configurations. The authors find that an initial excitation to the upper polariton branch quickly decays to the dark states which then transfer population to the lower polariton. This relaxation process is reflected in the photoluminescence (PL) spectra shown in Figure 24f. This is because unlike the absorption spectra in Figure 24e, the PL spectra depend on the populations of the polariton states and can be computed as
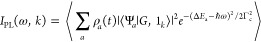
209where *ρ*_*a*_(*t*) is the steady-state (nonequilibrium photodriven condition) population of the *a*_th_ polariton state, |Ψ_*a*_⟩, at a delay time *t* after photoexcitation.

**Figure 25:**
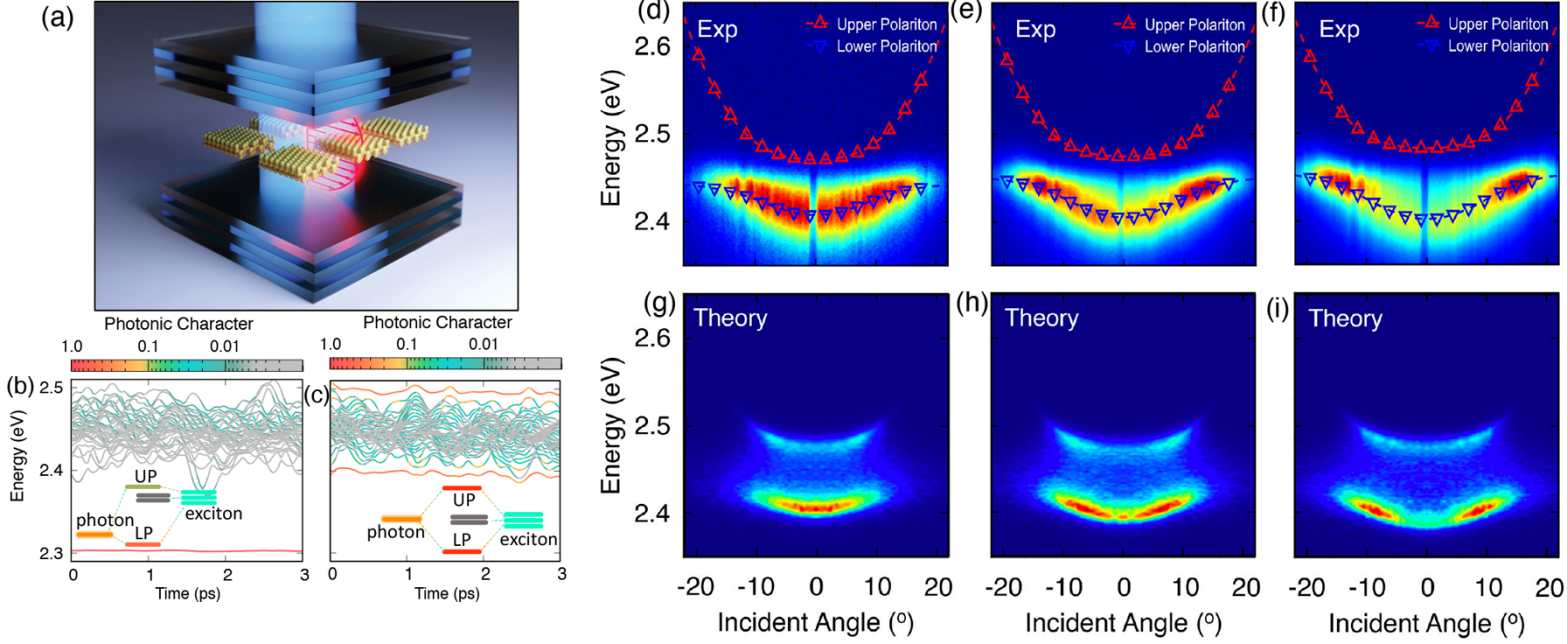
Quantum dynamics of nanoplatelets coupled to a cavity. (a) Schematic illustration of nanoplatelets coupled to a cavity mode. (b,c) Time-dependent polaritonic energies for a representative trajectory obtained with numerical simulation with a large detuning (b) and a small detuning (c). (d–i) Photoluminescence spectra obtained at increasing detunings from left to right experimentally (d–f) and theoretically (g–i) using direct quantum dynamics simulations. Reproduced with permission from ref (40). Copyright 2021 American Chemical Society.

Ref (40) simulated the PL spectra using a similar approach and analyzed the relaxation process from the upper polariton to the lower polariton through the dark state in an ensemble of nanoplatelets coupled to cavity radiation (as schematically illustrated in Figure 25a). This combined theoretical and experimental study demonstrates that at small exciton-photon detunings, the phonon-assisted nonadiabatic transitions lead to the depletion of the upper polariton population and the transfer of population to the lower polariton branch. The PL spectra obtained experimentally and theoretically are shown in Figure 25d-f and Figure 25g–i, respectively.

Figure 25b,c presents time-dependent polariton eigenenergies that fluctuate due to the evolution of phonons (in the mixed-quantum classical picture). In Figure 25b the photon frequency is much lower (off-resonant) than the molecular excitation. As a result, there is no substantial population transfer from the upper polaritons and the dark states (both of which are primarily excitonic) to the lower polariton (primarily photonic). As a consequence of this, the lower polariton, despite having a large photonic character, does not show up in the PL spectra since it does not get populated. At the same time even though the upper polariton and the dark states are substantially populated, they appear dark in the PL spectra due to negligible photonic character.

Figure 25c, shows the time-dependent polariton eigenenergies when photon frequency is close to the molecular excitation (∼ resonant). As the upper polariton is energetically close to the dark states, nonadiabatic transitions lead to the transfer of population from the upper polariton to the dark states. In the same way, the lower polariton gets populated by dark states through nonadiabatic transitions. Thus, at low detunings (or at resonance), the PL intensity congregates at the lower polariton as a result of both significant population and photonic character. Thus, in summary, this work^40^ concludes that the congregation of the PL intensity results from an interplay among phonon-mediated nonadiabatic transitions between polaritons, cavity loss, and the angle-dependent photonic character of the polariton branches. The resulting angular resolved PL spectra with various detuning (at zero angle) obtained experimentally and through direct quantum dynamics simulations are presented in Figure 25d–f and g–i, respectively.

In Figure 25d–f and g–i the Rabi-splittings are nearly the same while the detunings at zero angle Δ*E* are varied. Figure 25d–f (and Figures 25g–i) correspond to Δ*E* = −15.7 meV, Δ*E* = −29.6 meV, and Δ*E* = −34.6 meV, respectively. Overall, it can be observed that the congregation of the PL spectra directly depends on Δ*E*. At low Δ*E* the congregation of PL on the lower polariton is observed at low angles since the resonant condition is met at those angles, at which significant nonadiabatic transitions take place (Figure 25d and g). Similarly, at higher Δ*E* the resonant condition is met at a higher angle, and as a result, the congregation of PL on the lower polariton is observed at higher angles (Figure 25e,f and h,i). The theoretical simulations capture this qualitative trend Figure 25h,I thus verifying our theoretical understanding.

**Figure 26:**
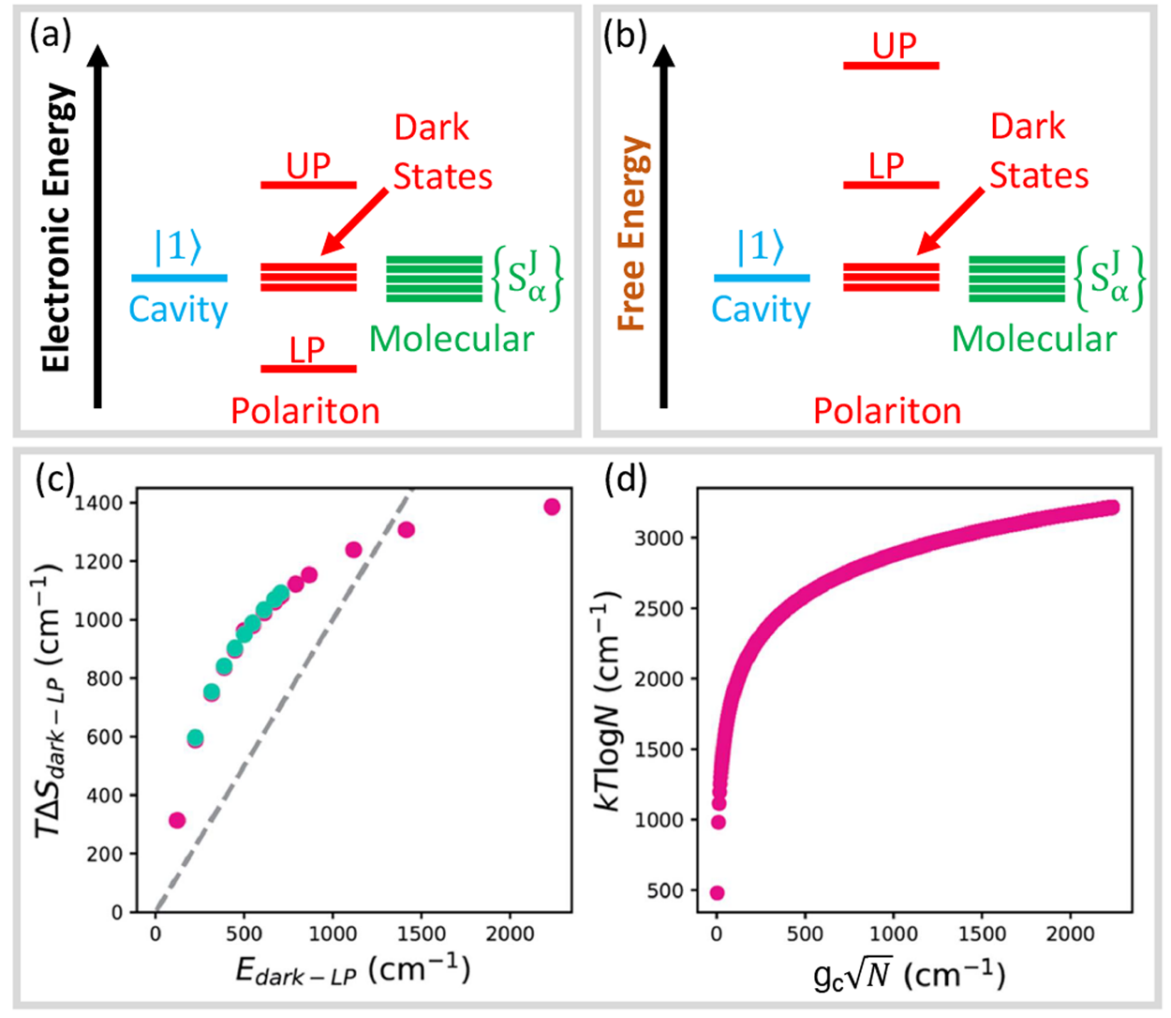
Entropy reordering theory. (a,b) Polaritonic states ordered according to their energy (a) and free-energy energy (b). Note that LP lies above dark states in (b) due to entropic contribution. (c) Entropic difference between dark states and the lower polariton at two different light–matter couplings, *g*_c_ = 50 cm^–1^ (red dots) and 75 cm^–1^ (cyan dots). Scaling of entropic contribution (*k*_B_*T* log *N* roughly estimates maximum entropy of dark states) to the dark state free energy as a function of collective light–matter coupling (equivalently *N*). Adapted from ref (485) with permissions. Copyright 2020 American Chemical Society.

The population transfers among polariton states in the works mentioned above are rationalized by considering their relative energetic ordering such that population dynamics flow downhill, which is reminiscent of Kasha’s law.^486^ Based on this picture, for a typical energetic ordering of polaritonic states shown in Figure 26a, we anticipate a relatively small upper polariton population and a relatively large lower polariton population. Ref (485) points out that this picture of energetic downhill population dynamics could be misleading as it ignores the entropic contribution which could make a dominating contribution to the free energy and dictate long-time populations. For example, while lower polaritons can lie energetically well below the dark states, the entropy of the lower polariton (that is in a delocalized superposition state) is much smaller than that of the localized (localized especially when considering disorder) dark states. Thus, the total free-energy *F* = *E* – *T* · *S*, with temperature *T*, energy *E* and entropy *S* may reorder polaritonic states as shown in Figure 26a,b.

To gain an intuitive understanding of this entropic reordering of polaritons,^485^ consider the single excited subspace spanning the *N* excitonic states {|*E*_*J*_, 0⟩} of the molecular subsystems that is coupling to a cavity excitation |*G*, 1⟩ (one photon in the cavity). As explained before, such as in Figure 24a and in Section 1.2, each of the |*E*_*J*_, 0⟩ states are coupled to the |*G*, 1⟩ state through the light–matter coupling, *g*_c_, and this leads to the formation of a lower polariton, upper polariton, and *N* – 1 dark-states. In reality, these polaritonic states also interact with their environment. For example, the molecular excitation {|*E*_*J*_, 0⟩} at site *J* is also interacting with some local dissipative environment, which will cause static disorder of the excitonic energies {*E*_*J*_}. To account for the interaction with the local environment, the authors^485,487^ sample {*E*_*J*_} from a random Gaussian distribution. For each realization of {*E*_*J*_}, the corresponding polaritonic eigenstate |Ψ_*a*_⟩ can be computed as |Ψ_*a*_⟩ = ∑_*j*_c_*j*_^*a*^|Φ_*j*_⟩, where |Φ_*j*_⟩ ∈ {|*E*_*J*_, 0⟩, |*G*, 1⟩} and *c*_*j*_^*a*^ = ⟨Φ_*j*_|Ψ_*a*_⟩, with the corresponding density matrix |Ψ_*a*_⟩⟨Ψ_*a*_| = ∑_*i,j*_ (*c*_*i*_^*a*^)*c_*j*_^*a*^|Φ_*j*_⟩⟨Φ_*i*_ |. The authors compute the average density matrix *ρ̂_a_* for the *a*_th_ polaritonic eigenstate averaged over random realizations of {*E*_*J*_} as
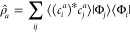
210where ⟨···⟩ denotes the average over the random realizations. With this, the von Neumann entropy *S*_*a*_ for the average *a*_th_ polaritonic state can be computed as
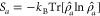
211

When computing the free energy associated with each polaritonic state, it is possible to have the “lower” polariton lying above the dark states because of its lower entropic contribution to its free energy as schematically depicted in Figure 26b. Figure 26c presents numerical results for *g*_c_ = 50 and 75 cm^–1^ represented by red and cyan dots respectively, with *N* = 2000 and a Gaussian disorder with a standard deviation of 25 cm^–1^ at *T* = 300 K. In both cases, a substantial number of dark states lie above the dashed solid line that is represented as *E*_Dark_ – *E*_LP_ = *T*(*S*_Dark_ – *S*_LP_). The reordering between dark states and the lower polariton occurs for *T*(*S*_Dark_ – *S*_LP_) > *E*_Dark_ – *E*_LP_. Therefore, such dark states lie below the “lower” polariton when considering free energy.

The size-scaling of this effect is semiquantitatively investigated in Figure 26d. Note that  while the maximum entropy of the dark states is approximately *k*_B_ log *N*, for large *N*. From Figure 26d it is evident that, for small *N*, the entropic contribution could dominate the free-energy ordering of polaritons, while at large *N* the energy gap between the lower polariton and the dark states will dominate. This can be also verified analytically by simply considering the ratio
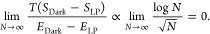


Overall, due to entropic contribution to the free energy, the lower polariton is more reactive to population transfer processes to higher-energy states (i.e., dark states) than it is generally anticipated when only considering their energetic ordering.^485^

Coupling to the cavity can also enhance excitation energy transfer and lead to faster energy transport, especially in materials. Using a generalized Tavis-Cummings Hamiltonian, ref (152) simulates the transport properties of organic crystals when coupled to plasmonic nanoparticle arrays as illustrated in Figure 24g. They find that the propagation length when coupling to a cavity is significantly larger than outside the cavity. Their simulations suggest that nonadiabatic transitions in combination with cavity decay dominate the transport mechanism and set an upper limit to the distance over which energy can be transported.

Ref (141) investigates ballistic transport in exciton-polaritons by tuning the polariton-phonon through light–matter interactions. The polariton-phonon coupling can be modified because the exciton couples to the phonon but the cavity excitation does not, and as a result, the phonon coupling strength directly depends on the excitonic character of the polariton which can be modulated by detuning. They find that the ballistic motion of polariton propagation can be observed even at high exciton content (∼25% excitonic) but with a reduced group velocity, as seen in experiments.^488,489^ Their quantum dynamics simulations indicate that the source of this group velocity rescaling originates from a transient localization process induced by the moderately weak interactions to phonon.^141^

### 6.2 Polariton Photochemistry in the Collective Regime

In this section, we will review theoretical works that propose the modification of photochemical reactivity in the collective regime. This is relevant for the present experimental setups^3,38,255^ where the individual light–matter coupling remains vanishingly small but the collective Rabi-splitting is substantial due to the scaling by  with *N* as the number of molecules.

Ref (356) shows that when a mixture of photoreactive molecules and photononreactive molecules is strongly coupled to the same cavity mode, photoexcitation to the lower polariton can be used to enable reactions in the photoreactive molecules. The main idea of this work follows from Kasha’s rule for the molecule-cavity hybrid system^155,313,356^ which suggests that polaritonic excitations relax into the lowest energy state available to the cavity-molecule system. They use this to funnel energy, initially deposited to the lower polariton, to a molecule that can undergo a photochemical reaction to energy levels below the lower polariton. Through direct on-the-fly atomistic simulations, the author shows that collective strong coupling can be utilized to enable reactivity in a few photoreactive molecules embedded in a large ensemble of nonreactive molecules.

**Figure 27:**
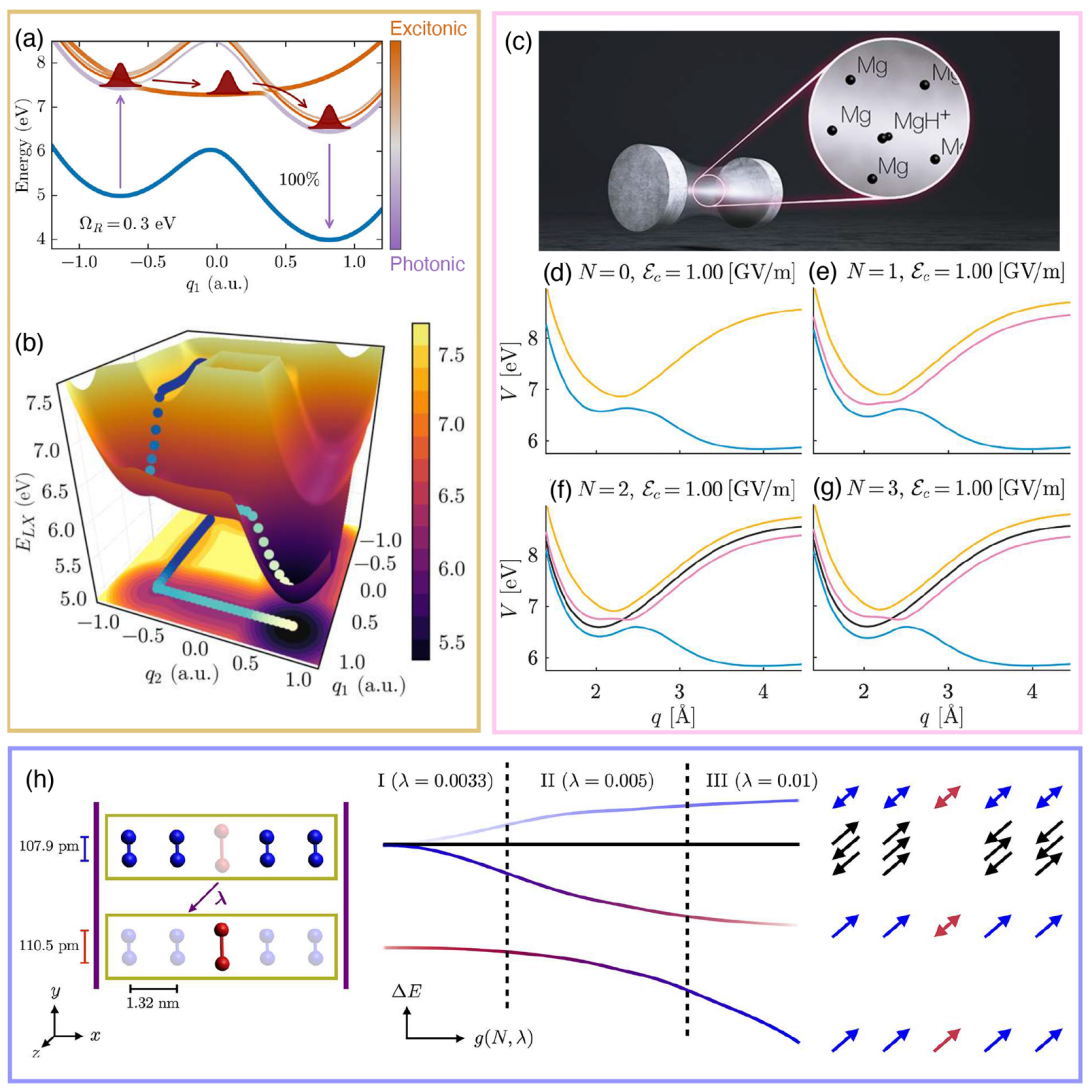
Modification of photochemical reactivity in the collective regime. (a) Polariton potential energy surface of one molecule coupled to a cavity mode along a molecular reaction coordinate with polaritonic states color with by photonic and excitonic character. (b) Polaritonic potential energy surface for two molecules coupled to one cavity mode. (c) Schematic illustration of a single MgH^+^ molecule and an ensemble of Mg atoms coupled to a cavity mode. (d–g) Polaritonic potentials along the dissociation coordinate of MgH^+^ with (d) 0, (e) 1, (f) 2, (g) 3 Mg atoms coupled to a cavity mode. (h) Polaritonic potentials (middle panel) for *N* – 1 identical molecules and one perturbed molecule (schematically illustrated in the left panel) with the character schematically illustrated in the right panel. Panels (a,b) are reproduced from ref (256) with permission. Copyright 2017 American Chemical Society. Panels (c–g) are reproduced from ref (490) with permissions. Copyright 2020 American Chemical Society. Panel (h) is reproduced from ref (238) with permission. Copyright 2021 American Chemical Society.

Ref (256) shows that chemical reactions in an ensemble of molecules can be triggered by a single photon when all molecules are coupled to a cavity photon mode. They represent each molecule with a one-dimensional reaction coordinate resembling an isomerization reaction. The ground state potential (blue solid line in Figure 27a) is characterized by a double-well potential with a large barrier between the left and the right wells corresponding to the product and reactant, respectively. As also explained before (such as in Figure 13e), the excited state potential energy landscape for a single molecule can be modified by coupling to cavity (with a specific photon frequency) such that photoexcitation leads to 100% product which is shown in Figure 27a. In such a scenario, the initially photoexcited molecule emits a photon inside the cavity as it reaches the local minima on the product side (the minima originates from the |*G*, 1⟩ state which is the ground state with 1 photon in the cavity) on the polaritonic potential energy surface. When multiple molecules are present in the cavity, the photon emitted at the end of one molecule reacting (reaching the local minima in the polariton potential energy surface) can be reabsorbed by another molecule, which then can undergo chemical reactivity. This is illustrated in Figure 27b where the polariton potential energy surface for two molecules coupled to a cavity mode is shown. The potential energy surface in Figure 27b reveals that the formation of the two product molecules follows a downhill process, and this photochemical reactivity can be triggered using just one photon. Thus, the quantum yield, defined as the number of products created per photon consumed, goes beyond unity. The same has also been shown beyond two molecules in ref (256). Thus, the photon here acts as a catalyst that is recycled between successive molecules that undergo chemical reactivity.

The mechanism described in ref (256) was confirmed through direct quantum dynamics simulation.^13^ In addition to this, ref (13) showed that instead of molecules directly emitting and absorbing a photon, molecular excitation can be exchanged when cavity photon frequency is off-resonant. In this case, the molecules can exchange a virtual photon, which may be protected from cavity loss and still lead to a quantum yield of more than 1.

Ref (490) investigates how collectively coupling an ensemble of Mg atoms to cavity radiation can modify chemical reactivity in a molecule (MgH^+^) also coupled to cavity radiation (as schematically shown in Figure 27c). Two relevant molecular electronic states and two atomic electronic states are considered in this work. The work assumes a Tavis-Cummings Hamiltonian and focuses on the single excited subspace spanning either a molecular or an atomic excitation or 1 photon in the cavity. The upper and lower polariton potential energy surfaces formed for a single MgH^+^ molecule coupled to a cavity mode along the dissociation coordinate *q* is shown in Figure 27d. When an atom is also coupled, a middle polariton is formed, as shown in Figure 27e (pink solid line). In the resonant situation when the bare molecular, photonic, and atomic transitions are degenerate (at some molecular nuclear configuration), the scenario reduces to what is shown in Figure 24a such that the middle polariton corresponds to a dark state (a superposition of the molecular and atomic excitation). Note that this middle polariton is not dark for any other nuclear configurations. When more atoms are added, new degenerate dark states are formed, which are shown in Figure 27f,g (black solid line). Regardless of *q*, light–matter coupling, or the additional number of atoms, these dark states remain decoupled from the rest of the polaritons and thus have no effect on the reactivity of the molecule. When only a single molecule is coupled due to the formation of the light–matter avoided crossing (Figure 27d between blue and yellow curve) the dissociation is suppressed and the molecule is photostabilized. This work finds that this stability, however, cannot be further enhanced with a large number of atoms *N* ≳ 10 coupled to the cavity. For a small number of atoms, *N* ≲ 10, the stability of the molecule may be enhanced. The authors report constructive and destructive interference at the avoided crossings which prevent molecular dissociation and leads to molecular stability. It is worth mentioning, as this analysis indicates, that collective cavity coupling can only affect a molecular excited state potential energy surface to a limited extent.

Overall, one of the main conundrums of modifying chemical reactivity in a cavity is that while the collective coupling of an ensemble of molecules to a radiation mode and the resulting collective Rabi-splitting is a global phenomenon (involving all molecules spatially spread inside the cavity), a chemical reaction is a local phenomenon in that only one molecule undergoes chemical reactivity at a time which is largely dictated by the potential energy surface of the single molecule. Thus, whether or not the collective coupling to all molecules also translates to a local modification of the potential energy surface of a single molecule remains an open question. Ref (238) has attempted to shed light on this issue.

Ref (238). uses an ab initio QEDFT (see details in Section 3.2.3) approach to investigate a chain of nitrogen dimers within a cavity. They find that collectively coupling all nitrogen dimers (with the same nuclear configuration) can modify the potential energy surface for small perturbations along the dissociation coordinate of one molecule in the vicinity of the uniform (such that all molecules have identical nuclear configuration) configuration. The effect of a small perturbation to one molecular nuclear configuration on the collective coupling is illustrated in Figure 27h. For identical molecules with uniform nuclear configurations, the collective cavity coupling gives *N* – 1 dark states and 2 polaritonic (upper and lower) bright states (as shown in Figures 1d and 24a). For one molecule perturbed, as schematically shown in Figure 27h (left panel), an additional polariton state appears as molecular excitation on one molecule (the perturbed one) is off-resonant to the molecular excitations on the rest of the molecules or cavity photon frequency. To understand this consider the rest of the *N* – 1 molecules collectively coupled to a cavity to form upper polariton, lower polariton, and *N* – 2 degenerate dark states, which corresponds to the higher energy levels in Figure 27h. The perturbed molecular excitation (lying energetically lower) then weakly couples to the upper and lower polaritons. As a result, there are four types of light–matter states, in ascending order of energy they are, (a) an upper polariton composed of *N* – 1 (unperturbed) molecular excitation, the cavity excitation (1 photon in the cavity), and of a relatively tiny fraction of the perturbed molecular excitation, (b) a set of *N* – 2 dark states composed of only *N* – 1 (unperturbed) molecular excitations, (c) a middle polariton with a similar composition as the upper polariton except for a relatively higher contribution (still tiny) from the perturbed molecular excitation, and (d) the lower polariton predominantly composed of the perturbed molecular excitation with a relatively low component of the other molecular and cavity excitations. It is the modification of the lower polariton that will lead to a modification of local chemical reactivity. The authors report, for the few molecules coupled to a cavity considered in their study, this lower polariton can indeed be modified by collective coupling to the rest of the molecules. However, the extent of the modification of this lower polariton is also limited by the light–matter coupling of a single molecule. Thus, when a single molecular coupling to the cavity is vanishingly small, such modification of a single molecular potential is unlikely regardless of how strongly the rest of the molecules are coupled to the cavity.

In a related work, ref (426) investigated collective effects in a Fabry–Perot-type electromagnetic environment, driven by classical electromagnetic fields following the previous development in ref (491). Interestingly, ref (426) shows that the short-time product population dynamics of a model proton transfer reaction (described by a time-dependent potential energy surface) has a nontrivial oscillatory dependence on the number of molecules collectively coupled to the cavity. However, it is unclear if the collective effect observed in the short-time coherent dynamics in this work necessarily translates into the modification of (incoherent) chemical kinetics that occurs at much longer time scales observed in the VSC experiments.

In conclusion, despite many interesting theoretical proposals, modifying chemical reactivity through collective light–matter coupling remains a challenging task. There are several ongoing efforts, both experimental and theoretical, that are focused on clarifying what photochemical reactions can be controlled through collective light–matter coupling and, in such cases, what mechanisms allow this to occur in spite of the minuscule coupling of individual molecules to the cavity. Regardless, the experimental and theoretical works up to this point have demonstrated the potential for many molecule photochemical reactions to be controlled through light–matter coupling and have set the stage for future works to modify photochemistry in the collective coupling regime.

### 6.3 Polariton-Mediated Charge Transfer in the Collective Coupling Regime

In this section, we will review works that have proposed possible ways to modify photoexcited electron transfer reactions in the collective regime. Typical photoexcited electron transfer reactions occur between an optically bright donor state to an optically dark acceptor.^492−496^ As expected, the bright donor states have a large transition dipole (from the ground state) while the dark acceptors have a negligible transition dipole. This asymmetry can be exploited in a cavity, as cavity radiation only couples to optically bright states (here the donor state), allowing us to tune such chemical reactivity.^8,12,379^

An optical cavity can modify photoexcited electron transfer through a wide range of mechanisms. To appreciate this, consider the polariton states |±⟩ at **resonance** given by
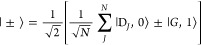
212where |D_*J*_, 0⟩ is the state where the *J*_th_ molecule is in its donor excited state while the rest of the molecules are in their ground states with 0 photons in the cavity and |*G*, 1⟩ represents the molecules in their ground state with 1 photon in the cavity.

First, note the cavity-mediated electronic coupling between |±⟩ and |**A**_*J*_,0⟩ state is ⟨**A**_*J*_,0|*Ĥ*_pl_|±⟩. Here only the |**D**_*J*_,0⟩ component in the |±⟩ is coupled to the |**A**_*J*_,0⟩ state through *Ĥ*_en_. The general expression of the effective electronic coupling is
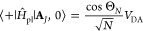
213a
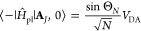
213bwith the mixing angle Θ_*N*_ (see under eq 14). Using these cavity-modified quantities, the charge transfer rate from | ± ⟩ to all possible final states {|**A**_*J*_,0⟩} is expressed as
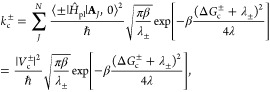
214where to arrive at the second line, we have explicitly evaluated the sum in the first line equation (which are *N* identical terms) and used the expression of the coupling (in eq 213a). Here Δ*G*_c_^±^ and λ_±_ are the polariton-mediated driving force and reorganization energy (between |± ⟩ and |**A**_*J*_, 0⟩) respectively, and the following effective electronic coupling
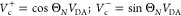
215where Θ_*N*_ is the mixing angle defined under eq 14. The cavity QED process can thus mediate the charge transfer process by modifying the driving force Δ*G*_c_^±^, the reorganization energy λ_c_^±^, and effective electronic coupling *V*_c_^±^. These quantities, and consequently the ET dynamics, can be tuned by changing the photon frequency ω_c_, as well as the light–matter coupling strength *ℏg*_c_. Thus, coupling molecules to the cavity opens up new possibilities to control ET kinetics by using fundamental properties of quantum light–matter interaction.

**Figure 28:**
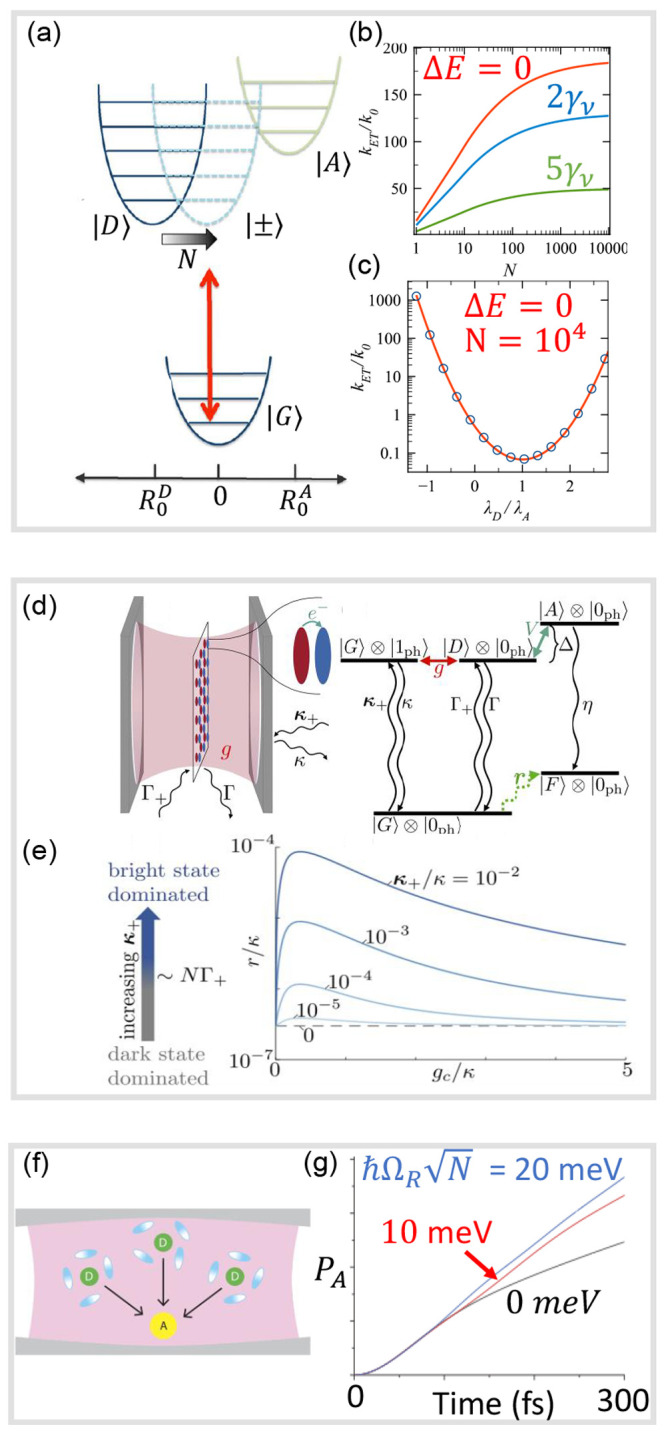
Modifying charge transfer reactions in the collective regime. (a) Schematic of the ground |*G*⟩, donor |D⟩, acceptor |A⟩, and polariton |±⟩ potentials. (b,c) Charge transfer rate as a function of (b) the number of molecules *N* at various differences in vibrational energy Δ*E* = *ω*_*DA*_ – (*m*_*D*_ – *m*_*A*_)*ω*_*ν*_ = 0 (red), 2*γ*_*ν*_ (blue), and 5*γ*_*ν*_, for  and (c) as a function of the donor energy shift *λ*_*D*_/*λ*_*A*_ with  and Δ*E* = 0. Here, *γ*_*ν*_ = 0.01*ω*_*ν*_ is the vibrational line-width, *ω*_*ν*_ is the vibrational frequency with *k*_B_*T* = 0.1*ℏω*_*ν*_. (d) Schematic illustration of a Fabry–Perot cavity depicting electron transfer in the collective regime. (e) Electron transfer rate as a function of light–matter coupling for various cavity loss rates κ. (f) Schematic of multiple donor (D) species coupled to an acceptor (A) inside a Fabry–Perot cavity. (g) The probability of ET from a donor (D) to acceptor (A) as a function of time for a various number of molecules: 0 (black), 10, (red), and 20 (blue) meV. Panels (a–c) were adapted from ref (8) with permissions. Copyright 2016 American Institute of Physics. Panels (d,e) were adapted from ref (379) with permissions. Copyright 2021 American Institute of Physics. Panels (f,g) were adapted from ref (497) with permissions. Copyright 2021 American Institute of Physics.

Ref (8) analyzes the effect of collective coupling on the modification of such excited state electron transfer reactions via the modification of reorganization energy with the key results shown in Figure 28a–c. The bare donor and acceptor states are displaced along the nuclear coordinate (along vibrational DOF) and thus have substantial reorganization energy relative to the ground state (Figure 28a). In this example, the donor state minima *R*_0_^D^ < 0 and the acceptor state minima *R*_0_^A^ > 0 are shifted in opposite directions with respect to the ground state minima (set as the origin). Thus, the reorganization energy between the donor and acceptor states is λ_D_ + λ_A_– ω_D_^2^R_0_^D^R_0_^A^ (for ω_D_ = ω_A_) where  is the of the donor reorganization energy relative to the ground state with ω_D_ as the donor-well frequency, and  is the acceptor reorganization energy relative to the ground state with ω_A_ as the acceptor well frequency. When there are *N* donor–acceptor pairs whose ground-donor transition is coupled to a cavity, the resulting polariton states are superpositions of the donor states with 0 photons in the cavity and the and the ground state with 1 photon in the cavity.

In particular, consider the polariton states  at resonance, where |D_*J*_, 0⟩ is the state where the *J*_th_ molecule is in its donor excited state while the rest of the molecules are in their ground states. The consequence of the  factor in front of the donor states in eq 212 is that the reorganization energy of these polariton states, in the strong coupling limit, is proportional to the donor reorganization energy times 1/*N*. This can be seen as a shift of the polariton parabolas toward the ground state configuration (which is closer to the acceptor state) as *N* increases (Figure 28a). In the limit of large *N*, this polariton reorganization energy goes to 0, which is known as polaron decoupling. Note that this effect also applies to the donor dark states, but does not apply to the acceptor states, which are uncoupled from the cavity in this example and thus retain their original reorganization energy relative to the ground state.

The consequences of polaron decoupling are demonstrated in Figure 28b,c. The reduction of the reorganization energies of the polariton and dark states relative to the ground state changes the reorganization energy of these states relative to the acceptor states, thus directly impacting the Marcus transfer rate. Figure 28b shows the effect of increasing the number of molecules while maintaining the same Rabi splitting. In this case, the increase in *N*, and consequential decrease in *λ*_*D*_, cause an increase in the cavity-modified ET rate relative to the rate outside the cavity. However, this increased relative rate eventually plateaus at large *N* since *λ*_*D*_ nears its limit of 0. The effect of the ratio of *λ*_*D*_ to *λ*_*A*_ in the large *N* limit is examined in Figure 28c. Depending on the relative magnitude and sign of the reorganization energies, *λ*_*D*_ versus *λ*_*A*_, the many-molecule cavity may experience an increased or decreased rate of electron transfer, with the largest rate increases seen at very large relative *λ*_*D*_ values. This demonstrates that light–matter coupling can have a very system-dependent effect on the rate due to polaron decoupling.

The polariton-mediated charge transfer dynamics can also be affected by cavity loss, particularly in relation to the strength of laser driving as shown in Figure 28d,e, adapted from ref (379). In this work, a Lindblad driving/decay model was constructed to describe charge transfer in a driven and lossy cavity (Figure 28d). Both the cavity loss rate κ and laser driving to the |*G*,1⟩ state κ_+_ were independently varied to determine their effects on the charge transfer rate inside the cavity. Even for laser driving rates orders of magnitude smaller than the loss rate, an increase of charge transfer rate relative to outside the cavity was observed (Figure 28e) which grew for larger relative driving strengths. These rates also varied as a function of the strength of the light–matter coupling relative to the cavity loss.

The collectivity of light–matter coupling can also facilitate a different arrangement of charge transfer reaction, a so-called “super-reaction” as shown in Figure 28f,g, adapted from ref (498). In this reaction, several donor molecules are coupled to a single acceptor molecule (Figure 28f). This arrangement allows charge from any of the donors to transfer to the acceptor molecule, which allows for an increase in rate as *N* becomes larger. In particular, the donor–acceptor coupling in the matter Hamiltonian has the form
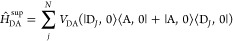
216and the coupling between the upper polariton and the acceptor state at resonance is
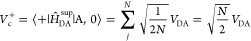
217such that the coupling strength between the upper polariton and the acceptor increases as . This is in stark contrast to the case when only individual pairs of donor and acceptor molecules are coupled and the coupling strength scales as  (see eq 213a). Additionally, the effective reorganization energy in the large Rabi splitting regime between the upper polariton and the acceptor is λ_+*A*_ = *Nλ* thus the reorganization energy of the polariton states scales as *N*. The rate constant at resonance is thus
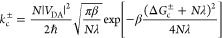
218

The super-reaction rate thus has a  dependence in its prefactor as well as an *N* dependence in the shoulder of the negative exponential that scales as *e*^–*N*^ when *Nλ* ≫ Δ*G*_c_^±^. The consequence of these scalings is that there exists some optimal value of *N* that maximizes the rate constant in eq 218 before the *e*^–*N*^ scaling kills the rate for larger *N*. The main principle behind why cavities can enhance super-reaction systems is that the protection of coherence between the donor molecules is especially important to maximize transfer rates to the acceptor molecule. Coupling to the cavity increases the coherence between these donor molecules by encouraging delocalized polariton and dark state formation. This ultimately leads to an increase in the acceptor population versus outside the cavity (Figure 28g).

Ref (499) investigates the possibility of modifying free charge carrier generation as in a system composed of oligothiophene donors and fullerene acceptors when coupling to the cavity. They model the oligothiophene as a chain containing *N* Frankel excitation sites (one electron and one hole is located at a site) |*XT*_*J*_⟩ ≡ |*D*_*J*_^*e*^⟩⊗|*D*_*J*_^*h*^⟩, with *e* and *h* representing an electron and a hole, whose nearest neighbors are coupled (|*XT*_*J*_⟩ is coupled to |*XT*_*J*__+1_⟩). They treat the fullerene molecules by an effective, coarse-grained supermolecule, such that there is one acceptor state |*A*_0_^*e*^⟩ which is localized on the fullerene supermolecule. As a result, there exists *N* charge transfer states |*CT*_*J*_⟩ = |*A*_0_^*e*^⟩⊗|*D*_*J*_^*h*^⟩, that is electron localized on the fullerene (supermolecule) and a hole localized on the *J*th site on the oligothiophene, which couples to its neighboring |*CT*_*J*__+1_⟩ state (similar to the |*XT*_*J*_⟩). In their analysis, they restrict themselves within the single excited subspace such that when considering the cavity they are considering the subspace spanning {|*XT*_*J*_, 0⟩, |*CT*_*J*_, 0⟩, |*G*, 1⟩}, where |*G*, 1⟩ is ground state of the matter with 1 photon in the cavity. Due to the light–matter interactions, each |*XT*_*J*_, 0⟩ couples to the |*G*, 1⟩} state. Finally, |*XT*_0_⟩ is coupled only to the |*CT*_1_⟩ state as the fullerenes are assumed to be spatially close to the *i* = 1 site of the oligothiophene.

The main idea of this work is to use the collective coupling of |*G*, 1⟩ to the {|XT_*J*_, 0⟩} states to enhance free charge carrier generation. Due to the collective Rabi-splitting that scales as  where *N* (see Figure 1d) is the number of sites, a lower polariton, upper polariton, and *N* – 1 dark states are formed. Because the lower and upper polaritons are energetically shifted, they can be brought closer or further away from the {|CT_*J*_, 0⟩} states thereby modifying the free charge carrier generation. They show that by such secondary hybridization, that is between the lower polariton and the |CT_1_, 0⟩, the free charge carrier generation is enhanced, as these two states are energetically brought closer through collective light–matter coupling. However, they also show that when considering cavity loss, the generation of free charge carrier is actually suppressed as the lower polariton has significant photonic character. Overall, they find that free charge carrier generation can be enhanced at short time scales (shorter compared to the cavity lifetime) but is suppressed due cavity loss at longer times.

### 6.4 Collective Effects in VSC-Modified Reactivities

The VSC experiments^4,17,37,41,130^ happen intrinsically in the collective coupling regime, where there are a large number of molecules (often >10^10^) coupled to the cavity modes and the light–matter coupling strength for each molecule is relatively small. Unfortunately, most theoretical works are restricted to the one molecule limit, which requires a nonphysical light–matter coupling strength or an extremely small cavity size.^14,85^ However, dealing with a model system with many molecules coupled to a cavity is challenging for both direct MD simulations and theoretical derivations. There have been quite a few attempts on explaining the mysterious collective effects, but more theoretical work is needed to provide a satisfactory answer.

The VSC Rabi frequency in eq 195 is only valid for the single-molecule case. The result can be generalized for *N* identical molecules {*R*_*J*_} coupling to *q̂*_c_. The light–matter Hamiltonian for N molecules coupled to one cavity photon mode (in the single excited subspace) is given by,
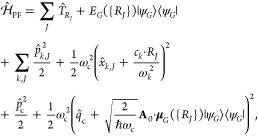
219where |*ψ*_*G*_⟩⟨*ψ*_*G*_| is the ground state of the matter with *E*_*G*_({*R*_*J*_}) as the ground state potential energy surface and **μ**_*G*_ ({*R*_*J*_}) is the ground state permanent dipole. For noninteracting molecules, we have  and **μ**_*G*_ ({*R*_*J*_}) ≈ *N***μ**_0_ + *∑*_*J*_**μ**_0_*R*_*J*_. Note the term *N***μ**_0_ can be removed by the translation  using a displacement operator for *q*_c_. With this simplification, the expression of the collective Rabi-splitting for the many-molecule case can be obtained by defining a collective molecular coordinate  which couples to the *q̂*_c_ with a collective coupling scaled by . At the resonant condition of ω_c_ = ω_0_, the Rabi splitting *ℏ*Ω_*R*_ in the collective coupling regime can be expressed as^85,431,441^
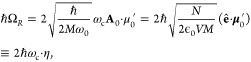
220where *N* is the total number of molecules coupled to the cavity mode, and the collective normalized coupling strength η characterizes the light–matter coupling strength. Setting *N* = 1 will go back to the single-molecule case. Note that the above relation between Ω_*R*_ and η only holds under the linear approximation of the dipole operator, and it breaks down for ultrastrong coupling (USC) regime and beyond when η > 0.1.^47^

While the scaling of the Rabi-splitting with the number of molecules *N* is well understood theoretically and verified experimentally, it is not clear how VSC modification of chemical reactivity could depend on *N*. Currently, there is no cohesive theory that fully explains the range of phenomena experimentally observed for VSC reactions in the collective regime. However, many groups have made important and notable advances to this field that hopefully further elucidate the problem at hand and inspire future advances in the field. With that in mind, the rest of this section discusses many of these creative theoretical advances in VSC, reviewing the methods, results, and drawbacks of each one of these theories.

Campos-Gonzalez-Angulo and Yuen-Zhou^440^ performed a normal-mode analysis of a model system where molecules were isotropically distributed and coupled to the same cavity mode. The Hamiltonian of the model system is shown in eq 221 as
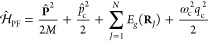
221
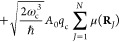
222where *M* is the mass of the molecule, *N* is the total number of molecules, *E*_*g*_(**R**_*J*_) is the ground-state potential energy surface of the *J*-th molecule, *A*_0_ characterizes the strength of the light–matter coupling strength, ϵ is the polarization vector of the cavity field, and μ(**R**_*J*_) is the dipole moment of the *J*-th molecule. When one molecule is in the transition state, the Hamiltonian can be rewritten in an effective 3-mode expression , where **x** = {*R*_‡_, *R*_B_, *q*_c_} represents the coordinates of the reactive molecule, the collective bright mode, and the photon mode. To compute the normal-mode frequencies, the 3-mode Hessian matrix is written as,
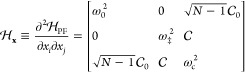
223where ω_0_ is the reactant frequency, ω_‡_ is the barrier frequency, μ_0_’ is the slope of the permanent dipole at the reactant well, μ_‡_’ is the slope of the permanent dipole at the transition state, ⟨···⟩ denotes the ensemble average, and  represents the coupling between the cavity mode and the collective bright mode. Here, we have introduced  which characterizes the light–matter coupling strength. Clearly, the coupling strength between the reactive molecule and the cavity mode is limited by  (single-molecule coupling strength). As a result, the reaction rate will not depend on the number of molecules. The same conclusion is drawn when using the Pollak-Grabert-Hänggi theory that extends the MTST to the energy diffusion-limited regime.^500^ Then the normal-mode frequencies are used to compute *κ*_*N*_, which is the ratio between the rate constant of *N* molecules inside the cavity and the TST rate of one molecule outside the cavity,
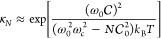
224

As the denominator scales with respect to the number of molecules *N*, when *N* is large, *κ*_*N*_ → 1. In other words, when *N* is large (*N* = 10^9^ in the original work), the reaction rate has no obvious dependence on the coupling strength or the scale of the dipole moment. Note that the single-molecule limit of such theory is equivalent to what was presented in ref (85) when considering the dipole self-energy term.

Galego and co-workers^441^ performed classical molecular dynamics simulations to explore a system of many molecules distributed around a sphere nanoparticle, where the permanent dipoles of molecules are aligned along the direction of the field of the sphere’s *z*-oriented dipole mode. The simulation results show that both the dipole-sphere interaction (between the molecules and the nanosphere) and the dipole–dipole interaction (between the molecules) have positive contributions to the potential energy barrier of the whole system, so that the barrier increases almost linearly with respect to the number of molecules coupled to the sphere. Consequently, the TST rate will decrease exponentially due to the monotonic increase of the reaction barrier. Even though the authors find rate suppression in the *perfectly aligned* case, the frequency dependence (resonant effect) is obviously missing.

**Figure 29:**
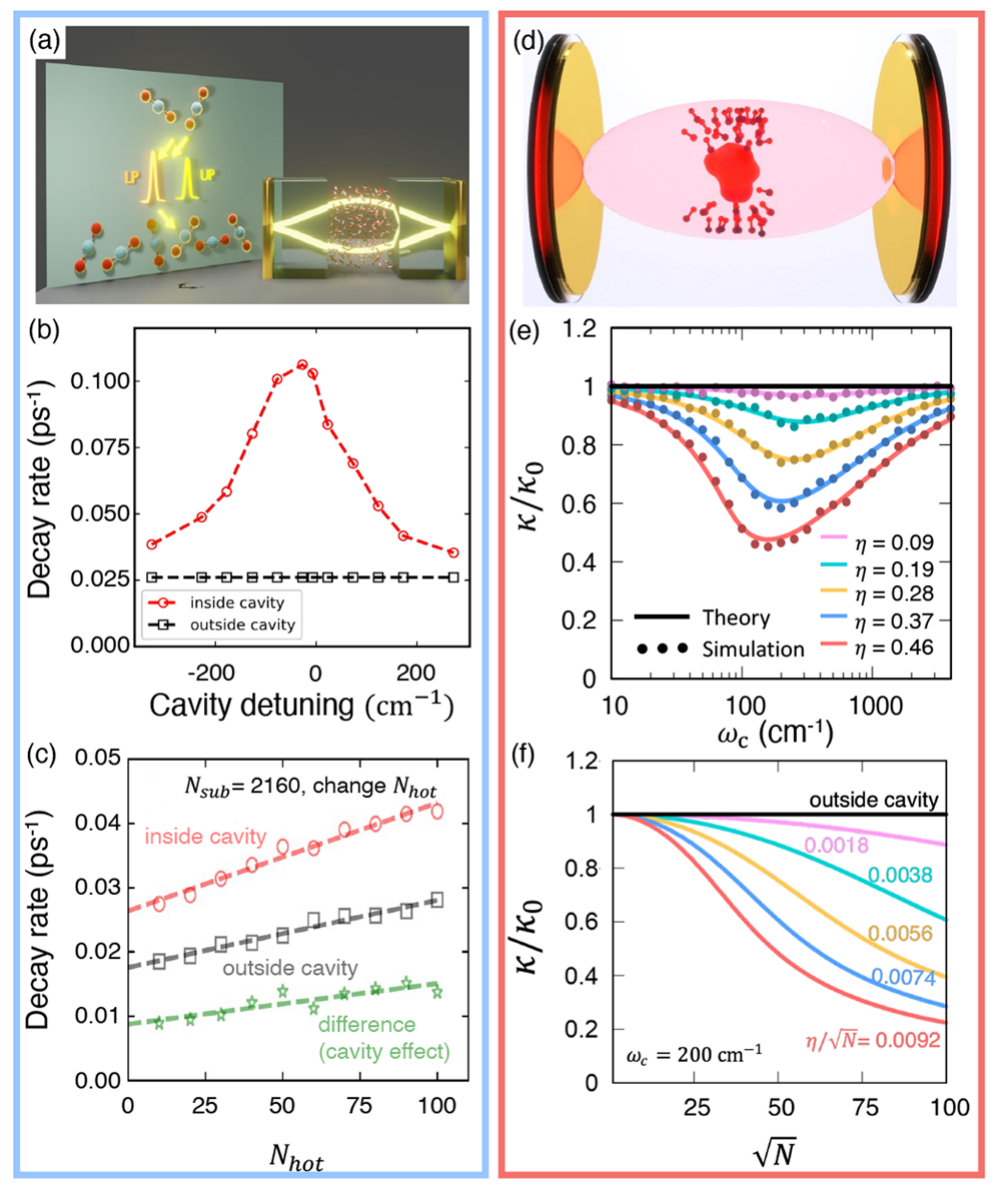
Cavity modification of ground state kinetics in the collective coupling regime. (a) A schematic illustration showing some “hot” CO_2_ molecules (with higher thermal energy) surrounded by a thermal bath of other CO_2_ molecules which are at room temperature. All these molecules collectively couple to a cavity mode. (b) Fitted vibrational energy relaxation rates as a function of the cavity mode frequency. The enhancement of energy dissipation is in resonance with the cavity frequency. (c) While the number of CO_2_ molecules in the thermal bath (*N*_*sub*_) is fixed, increasing the number of hot molecules (*N*_*hot*_) enhances thermal dissipation both inside and outside the cavity. However, the enhancement inside the cavity increases faster with respect to *N*_*hot*_. (d) A rendering shows a model system where a reactive molecule is coupled to some solvent molecules that are coupled to the cavity mode. (e) Fixing the total number of solvent molecules (*N* = 2,500), increasing per-molecule light–matter coupling will further suppress the reaction rate. Note that the suppression is in resonance with the cavity frequency. (f) Reaction rate as a function of the total number of solvent molecules (*N*) at different fixed per-molecule light–matter coupling strengths (shown as the numbers). The reaction rate decreases monotonically in all cases. Panels (a–c) are adapted with permission from ref (501). Copyright 2021 Wiley-VCH. Panels (d–f) are reproduced with permission from ref (116). Copyright 2022 American Institute of Physics.

Nitzan and co-workers^501^ used classical molecular dynamics to simulate a model system with many CO_2_ molecules coupled to a cavity mode shown in Figure 29a. The strong coupling is formed between the cavity mode and the C–O bond stretching mode in the CO_2_ molecules. A fraction of the molecules are “hot”, which are thermally activated and have higher kinetic energy. The rest CO_2_ molecules are at room temperature, which are called “thermal” molecules and act like a thermal bath to dissipate excess energies from the “hot” molecules. Figure 29b shows that inside the cavity the fitted vibrational relaxation rates are much larger than the rates outside the cavity. This shows that polaritons can facilitate the intermolecular vibrational energy transfer between the hot CO_2_ molecules and the thermal bath. This effect is especially strong at the resonant condition where the cavity frequency is close to the C–O bond stretching frequency. Figure 29c shows that while the total number of molecules (*N*_*sub*_) is fixed, increasing the number of “hot” molecules results in faster energy dissipation both inside and outside the cavity. However, the increase of decay rate is faster inside the cavity, so the difference between the two rates increases as well, so this cavity-enhanced energy transfer depends on the Rabi splitting and scales with the number of hot molecules. Although polaritons are always transiently excited and able to mediate the energy transfer, the modification on the average relaxation rates becomes negligible when the total number of CO_2_ molecules exceeds a certain number (*N*_*sub*_ > 10^4^ as reported in the work).

In ref (116), the authors developed a model system, shown in Figure 29d, where a reactive molecule couples to many solvent molecules and these solvent molecules then couple to the cavity mode. The model Hamiltonian is written as
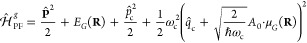
225where *E*_*g*_(**R**) is modeled as
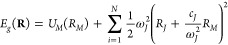
226

The solute molecule is modeled as a double-well potential *U*_*M*_ (*R*_*M*_) = *aR*_*M*_^4^ - *bR*_*M*_^2^. At the top of the barrier . Further, the total dipole of the system is *μ*_*G*_ (**R**) = ∑_*J* = 1_^*N*^*μ*_*J*_ (*R*_*J*_) ≈ ∑_*J*_μ_J_’ *R*_*J*_ (where *μ*_*J*_′ = *dμ*_*G*_ (**R**)/*dR*_*J*_), and we assume that *μ*_*M*_(*R*_*M*_) = 0. Here, *c*_*J*_ is the reactant-solvent coupling constant and *ω*_*J*_ is the solvent frequency. For simplicity, we assume that the solvent molecules are identical, such that *c*_*J*_ = *c*_s_, *ω*_*J*_ = ω_s_ and *μ*_*J*_′ = *μ*_*s*_′. Note that these solvent molecules are aligned anisotropically around the reactive molecule. Similar to the previous work in the single-molecule limit,^85^ the GH theory can be applied to this system to study the reaction rate suppression due to the cavity mode. However, unlike in the single molecule limit, the suppression observed here will also depend on the solvent frequency ω_s_ (in addition to the barrier frequency ω_‡_) and the total number of solvent molecules *N*. At the dividing surface *R*_*M*_ = *R*_*M*_^‡^, the Hessian matrix in the 3-mode **x** subspace is shown in eq 227,
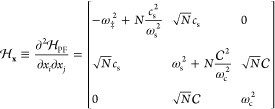
227where **x** = {*R*_*M*_^‡^,*R*_B_,*q*_c_} represents the coordinates of the reactive molecule, the collective bright mode, and the photon mode. ω_‡_ is the barrier frequency of the reactive molecule and . Note the interesting structural difference between eq 227 and eq 223. In eq 227, while both the off-diagonal coupling terms scale by , in eq 223 only one off-diagonal term scales by . The presence of this additional  in eq 227, which appears due to intermolecular interactions (solvent–solute interactions) is the origin of the collective “resonant” suppression shown in Figure 29e. This also indicates that such intermolecular interactions might be one of the missing pieces for solving the mystery of VSC in the collective coupling regime.

The normal-mode frequencies can be obtained by solving eq 227 and the transmission coefficient κ can be computed by plugging these normal-mode frequencies into eq 202. Figure 29e shows the trend of κ/κ_0_, where κ is the transmission coefficient inside the cavity and κ_0_ is the transmission coefficient outside the cavity, concerning different light–matter coupling strengths. When the total number of solvent molecules is fixed (*N* = 2500), the reaction rate is suppressed at all tested coupling strengths, and there is a clear resonant structure for the cavity frequency. The rate profile, similar to its single molecular counterpart^85^ presented in Figure 21b, shows a much broader cavity photon frequency dependency than is observed in experiments. However, note that the *resonant* photon frequency (where the highest cavity modification is observed) is related to ω_s_ as well as the barrier frequency ω_‡_. This is in contrast to the single molecule scenario where the *resonant* photon frequency did not depend on any (stable) molecular vibrational frequency. Further, it demonstrates the collective behavior, as one increase the number of solvent DOF, the VSC modification increases.. Figure 29f shows the cavity-modified reaction rate with respect to the number of solvent molecules, while the cavity frequency is fixed at ω_c_ = 200 cm^–1^. At a certain per-molecule light–matter coupling strength, increasing the number of molecules will further suppress the reaction rate. Additionally, the authors also explored the effects of cavity loss and found that cavity loss can further enhance the dissipation capability of the cavity mode, which will lead to more suppression of the reaction rate.^116^ Note that the setup of this model system is not directly related to the experimental setups shown in Figure 19a. Here, *N* denotes the number of solvent DOF (which are also collectively coupled to the cavity) directly coupling to the reactive molecule, while the experiments in Figure 19a suggest the reactivity depends on the number of reactive molecules (or their concentration in Figure 20b) collectively coupled to the cavity. Nevertheless, there are VSC experiments that directly couple cavity mode to the solvent DOF, whereas the solvents are then coupled to a solute molecule that undergoes reactions. In Figure 19c, the rate constant is enhanced when the cavity mode is collectively coupled to the solvents, which are also coupled to the reactive molecules. In a very recent experiment of VSC modified Urethane Addition Reaction,^420^ it was also found that when collectively coupling the solvent DOF with the cavity mode (where the solvent also interacts with the reactive molecule), the rate constant is suppressed, which is in favor to the theoretical results proposed here. We should emphasize that by no means does this theoretical work provides the ultimate answer to the mysteries of the VSC modification of the reactivities. We envision that this theoretical work brings us one step closer to finally resolving the mysteries of VSC enabled chemistry demonstrated in recent experiments^4,17,18,41,131,432^ by demonstrating both the collective coupling effect and the cavity frequency dependent modification of the rate constant.

**Figure 30:**
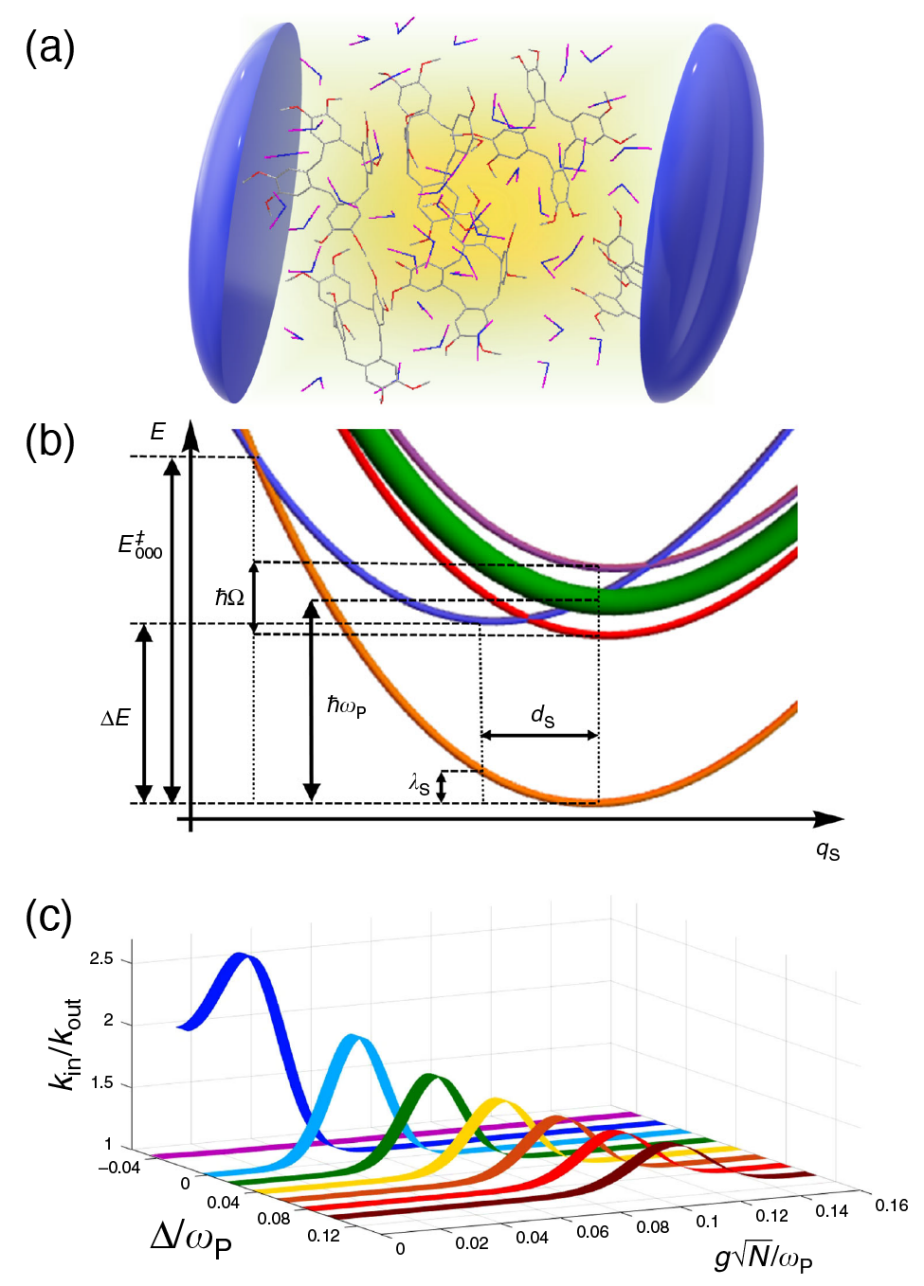
Cavity modification of nonadiabatic electron transfer reaction through vibrational strong coupling. (a) Schematic illustration of an ensemble of molecules placed inside an optical cavity. (b) Vibro-polariton potential energy surfaces of the molecule-cavity hybrid system. (c) Chemical rate constant as a function of cavity detuning Δ and total light–matter coupling strength . Reproduced from ref (45) under the CC BY license.

Finally, ref (45) investigated the VSC effect using a model that couples radiation modes to the vibrational degrees of freedom in nonadiabatic electron transfer reaction. The authors in that work considered an ensemble of molecules placed inside an optical cavity (schematically shown in Figure 30a) with quantized radiation described by a single cavity mode. Each molecule has a reactant (donor) |*R*_*J*_⟩ and a product |*P*_*J*_⟩ electronic state (with *J* as the index for the molecule) and they are coupled to a high-frequency molecular vibration such that the electron transfer rate (reactant to product) constant is computed using the MLJ theory. The cavity excitation is assumed to be coupled to the vibrational excitation but only on the product such that the light–matter coupling term read *g*∑_*J*_*â*_c_^†^*â*_*i*_ |*P*_*J*_⟩⟨*P*_*J*_ |+*h.c*. where *g*_c_ is the light–matter coupling strength. In other words, a product state with no vibrational excitation and one photon in the cavity _c_^†^|*P*_*J*_⟩⊗|0⟩ (where *â*_c_^†^ is the cavity photon creation operator) is coupled to the product state with a vibrational excitation and no photons in the cavity *â*_*J*_^†^|*P*_*J*_⟩⊗|0⟩ where |0⟩ represents the vacuum state of the cavity and the molecular vibrations. For *N* identical molecular vibrations, only one collective bright vibration, representing delocalized vibrational excitation over all molecules, can be shown to hybridize strongly to the cavity excitation, such that the light–matter coupling can be written as . where . At the same time, *N* – 1 dark vibrational excitations remain uncoupled from the cavity photon mode. The resulting hybrid vibro-polaritonic states are schematically illustrated in Figure 30b.

Figure 30b presents the vibro-polariton energies along a reaction coordinate *q*_*s*_. Here the driving force between the reactant state (blue solid line) |*R*_*J*_⟩ and the product state (orange solid line) |*P*_*J*_⟩ is in the Marcus inverted regime. Due to the light–matter coupling between *â*_*B*_ and *â*_c_ two light–matter hybrid states, lower and upper polariton states, are formed which are indicated as red and violet solid lines. The relative driving force between the reactant state and the lower polariton thus depends on the light–matter coupling  where *N* is the number of products. Thus, the chemical rate increases as more products are formed. Meanwhile, the relative driving force between the reactant state and (*N* – 1) excited vibrational dark states (green solid line) remains the same as the uncoupled case. The chemical rate modification as a function of the light–matter coupling  and the detuning Δ = ω_c_ – *ω*_*p*_, where *ω*_*p*_ is the frequency of the vibrational mode on the product state, is shown in Figure 30c. The bell-shaped rate curves are because as the Rabi splitting increases, the activation energy of the lower polariton decreases, thus making this channel dominant.^45^ The authors find a parameter range where, despite the vastly greater number of dark-state channels (*N* – 1) than polaritonic ones, the latter controls the reaction’s kinetics due to their lower activation energies.^45^

Using a similar model system, ref (498) showed that such nonadiabatic ground state electron transfer reactions can also be suppressed in addition to being enhanced as shown in ref (45). The authors in ref (498) point out the two main factors in modifying such reactions: (i) through the modification of the driving forces due to the shifts of the energy levels induced by the light–matter coupling and (ii) through the modification of the Franck–Condon factors that rescale the diabatic coupling. They find that when the cavity coupling for the reactant and product states differ significantly from each other (as was the case in ref (45)) the cavity coupling leads to an increase in chemical rate. On the other hand, when cavity coupling for the reactant and product states are similar in magnitude the modification of the Franck–Condon factors leads to suppression of chemical kinetics, especially at ultrastrong vibrational coupling regime.

Meanwhile, ref (145) points out that a realistic cavity contains a distribution of cavity modes and not just *k*_*x*_ = 0 mode (see Figure 4). When considering the full polariton dispersion the authors find a negligible effect in the VSC regime for nonadiabatic electron transfer rate for the type of model system studied in ref (45). Specifically, while ref (45) implicitly assumes that the density of states consists of three delta functions which are at the lower and upper polariton and the dark states, ref (145) generalizes their approach to a continuous density of states of polaritons and dark states. By doing so they find that the overall chemical reaction rate is proportional to an energy integral rather than a sum over three discrete contributions. The net cavity modification of chemical rate under such circumstances is negligible. This work also illustrates the importance of studying cavity-mediated chemical reactions beyond a single cavity mode.

Overall, despite many theoretical efforts, a clear theoretical explanation of the experimentally observed modifications of ground-state chemical reactivity is unavailable. However, these studies will undoubtedly inspire future research that may 1 day solve the mysteries of cavity-modified ground-state chemical reactivity.

## 7 Conclusions and Future Directions

As the experimental demonstrations of molecular cavity QED in the strong and ultrastrong coupling regimes become more frequent and accessible to the broader community, there is a need for the development of new theoretical tools that can accurately and efficiently describe such complex light–matter interactions found in experiments. This review summarizes some of these exciting theoretical advances in polariton chemistry, showcasing methods ranging from improvements in the fundamental framework and description of these hybrid systems to the computational challenges, techniques, and applications spanning from modifying reactivity in the ground state to understanding spectral signatures of excited state photochemistry.

In Section 2, we discussed the rigorous theoretical background of molecular cavity QED. We first reviewed the basic theory of the molecular Hamiltonian (Section 2.1) and quantum electrodynamics (Section 2.2). Section2.3 further reviews different forms of the QED Hamiltonians under different gauges and provides a clear connection among them through gauge transformations. Even though the theory of QED goes back to the midtwentieth century, we discussed recent advances made in resolving gauge ambiguities to describe interactions between light and matter. In particular, Section 2.4 highlighted several possible causes of such ambiguities and their resolutions, which enable consistent physics regardless of the chosen gauge. In Section 2.5, we then connected the most rigorous QED Hamiltonian with various approximate Hamiltonians commonly used in the quantum optics community, which can be achieved through intuitive arguments and simple mathematical approximations of the rigorous Hamiltonian. Finally, in Section 2.6 we discussed light–matter interactions between many molecules and many cavity modes inside a Fabry–Pérot cavity, which is one of the most experimentally relevant setups.

In Section 3, we discussed the recent progress of ab initio polariton chemistry calculations, where one aims to solve polariton eigenvalue problems with real molecular systems. Particularly, we reviewed two approaches for performing these calculations: the parametrized QED (Section 3.1) approach and the self-consistent QED (Section 3.2) approach. Along with a brief overview and direct comparison of the two methods (Section 3.3.3), in Section 3.3, we showcased recent works that have implemented these approaches and demonstrated their ability to calculate chemically relevant properties (Section 3.3.3) in both the excited (Section 3.3.3) and ground polaritonic states (Section 3.3.3).

In the second half of this review, we discussed theoretical and computational applications that use the approaches outlined in the previous sections. Experiments have shown that, by tuning the cavity photon frequency and light–matter coupling between the quantized cavity photons and electronic transitions, photochemical reactions can be controlled inside an optical cavity. In Section 4, we revealed how through excited state nonadiabatic polariton dynamics simulations, theorists, inspired by experiments, have discovered new ways of modifying and enabling photochemical reactivity by exploiting quantum light–matter interactions. In Section 4.1, we briefly outlined some of the quantum dynamics approaches used for simulating polariton dynamics exactly and approximately. Then, in Section 4.2, we introduced intuitive schemes and possible approaches for modifying and manipulating photochemistry with the readily available theoretical tools from cavity QED. Following this, in Section 4.3, we showed how ab initio on-the-fly simulations can validate these schemes toward modifying photochemical reactivity in real molecular systems, thereby revealing previously unknown basic principles of how polaritons can be used to manipulate excited state features and dynamical properties. Further, in Section 4.4 we show that the same ideas can be applied in the modification of photoinduced charge transfer reactions. We then reviewed works in Section 4.5 that demonstrate the possibility of introducing new conical intersections through light–matter interactions and their impacts on excited state processes. In Section 4.6, we showed that the choice of the initially prepared quantum state of the cavity photon can also be used to directly control photochemistry. We concluded this section with a discussion in Section 4.7 on the important role of cavity loss in these excited state processes and illustrated how the nonideal nature of real experiments (e.g., partially transparent mirrors) can inhibit or enhance cavity control of photochemistry.

If the cavity resonance is instead tuned to the vibrational (i.e., instead of electronic) transitions in the molecule, referred to as the vibrational strong coupling regime, enhancement *and* suppression of ground state chemical reactions have been experimentally observed. We presented a few recently proposed theoretical explanations of this (largely unresolved) phenomenon in Section 5. Within this section, we first introduced a model Hamiltonian in Section 5.1 for a single molecule coupled to a cavity radiation mode and showed in Section 5.2 why simple one-dimensional classical transition state theory (TST) fails to predict any modification to the chemical reactivity when coupling to the cavity for this model. In Section 5.3 and 5.4, we further developed the model and showed how Grote–Hynes (GH) rate theory (or the multidimensional TST), which also treats all degrees of freedom (DOFs) classically, predicts a suppression of chemical reactivity albeit much broader than what is observed in experiments, as well as it predicts the rate suppression when the cavity frequency matches the reaction barrier frequency (which has not been observed by any experiments). Nevertheless, the GH theory provides a conceptually simple idea–the so-called solvent caging effect, where the cavity radiation mode acts as a non-Markovian solvent DOF – to explain the cavity-mediated suppression. Within this section, we also showed that approximate quantum corrections to the GH theory tend to depart further from experimental observations while exact quantum dynamics simulations make predictions much closer to experiments and depict similar features in the chemical rate that are sharply peaked at resonance conditions. In Section 5.5 we showed that the cavity photonic mode, which was shown to act as a solvent DOF in Section 5.4, can also enhance the chemical reactivity when solvent-molecule interactions are weak and can be understood using the Pollak-Grabert-Hänggi rate (PGH) rate theory, which classically treats all degrees of freedoms. Again, we show that the direct quantum dynamics result (at the single molecule limit) shows a sharp resonant cavity modification (enhancement) of chemical reactivity, which is visually similar to the experiments. Finally, to conclude this section, we described in Section 5.6 how the cavity can also modify thermally activated nonadiabatic electron transfer reactions. One major deficiency of many of these theoretical works is that they operate in the single molecule limit, whereas the experiments operate in the collective coupling regime with a large ensemble of molecules simultaneously coupled to the cavity radiation. To this end, in Section 6, we reviewed theoretical works that operate in the collective coupling regime.

Overall, with the recent new capabilities demonstrated in experiments, there has been a recent push to rigorously simulate polariton systems in the strong coupling regime. This has led to a number of theoretical innovations that start to explain and predict these experimental results. However, there are still many mysteries to solve as the systems get increasingly more complex with more molecules and cavity modes.

From the theoretical perspective, the single-molecule case has made significant progress due to the relative numerical simplicity of the simulations compared to highly expensive many-mode (with many Fock states) and many-molecule (with many electronic levels) simulations that have yet to be fully explored. From the experimental perspective, single-molecule spectroscopy in plasmonic cavities is extremely challenging and has not been widely achieved; however, the results stemming from such simple hybrid systems will afford a much greater leap forward in understanding.

The theoretical understanding of how cavities can control photochemical reactions in the collective coupling regime has also seen significant progress, particularly for polariton-mediated electron transfer reactions. Important experimental work remains to confirm the collective coupling mechanisms proposed by theorists and to further demonstrate changes in photochemical reactivity in the collective coupling regime for a wider variety of reactions.

Despite the recent progress discussed in Section 6, we still do not clearly understand the mechanisms of collective vibrational strong coupling and their modification of reactivities, or the available mechanisms that can take advantage of the collective coupling of forming polariton that changes photochemistry reactivities. In Section 6.1, we review works that elucidate how collective coupling can modify photophysical properties, such as energy/carrier transport, population dynamics, and linear and nonlinear spectroscopy. On the other hand, in Sections 6.3 and 6.2, we show how photochemical reactivity may be modified by collective effects. Finally, we discuss the mysteries of modifying chemical reactivity in the vibrational strong coupling in Section 6.4 and illustrate a few theoretical works that have attempted to address this issue. This cutting-edge research has many opportunities for both theorists and experimentalists to contribute and discover new physics. In this manner, much work is needed from both sides to demystify these collective effects and unlock their potential applications.

The purpose of this review was to provide fundamental knowledge for the readers in the emerging field of polariton chemistry. Through the examination of the recent literature, this review aimed to provide, in a single location, much of the current working theoretical knowledge of polariton chemistry for the continued efforts of both the chemistry and quantum optics communities to actively participate in this exciting new research direction. Hopefully, this work can inspire the discovery of new principles and mechanisms of chemical reactions that take advantage of intrinsic quantum light–matter interactions and facilitate a quantum leap in chemistry.
